# New Benchmark in DNA-Based Asymmetric Catalysis: Prevalence of Modified DNA/RNA Hybrid Systems

**DOI:** 10.1021/jacsau.2c00271

**Published:** 2022-08-02

**Authors:** Nicolas Duchemin, Sidonie Aubert, João V. de Souza, Lucas Bethge, Stefan Vonhoff, Agnieszka K. Bronowska, Michael Smietana, Stellios Arseniyadis

**Affiliations:** † Queen Mary University of London, Department of Chemistry, Mile End Road, London E1 4NS, United Kingdom; ‡ NOXXON Pharma AG, Max-Dohrn-Strasse 8-10, Berlin 10589, Germany; § Chemistry−School of Natural and Environmental Sciences, Newcastle University, Newcastle NE1 7RU, United Kingdom; ⊥ Institut des Biomolécules Max Mousseron, Université de Montpellier, CNRS, ENSCM, 1919 Route de Mende, Montpellier 34095, France

**Keywords:** DNA catalysis, asymmetric catalysis, artificial metalloenzyme, DNA−RNA hybrid, Friedel−Crafts alkylation, Michael additions

## Abstract

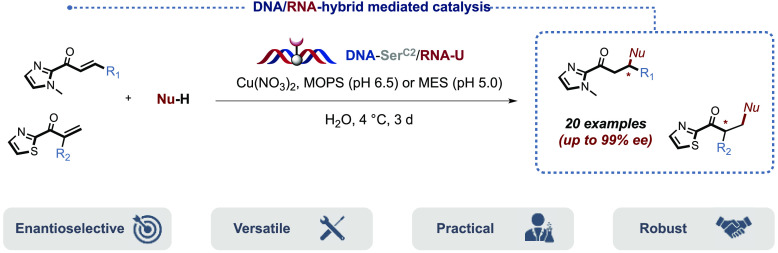

By harnessing the chirality of the DNA double helix, chemists have been able to obtain new, reliable, selective, and environmentally friendly biohybrid catalytic systems with tailor-made functions. Nonetheless, despite all the advances made throughout the years in the field of DNA-based asymmetric catalysis, many challenges still remain to be faced, in particular when it comes to designing a “universal” catalyst with broad reactivity and unprecedented selectivity. Rational design and rounds of selection have allowed us to approach this goal. We report here the development of a DNA/RNA hybrid catalytic system featuring a covalently attached bipyridine ligand, which exhibits unmatched levels of selectivity throughout the current DNA toolbox and opens new avenues in asymmetric catalysis.

## Introduction

Biohybrid catalysis has recently emerged as a powerful tool offering new perspectives in synthetic organic chemistry and beyond.^1,2^ Nonetheless, despite Nature’s vast repertoire of enzymatic transformations, many reactions still lack catalysts capable of promoting them in a highly selective and efficient manner. Inspired by the fascinating microenvironments provided by natural enzymes, considerable progress has been made over the years to design artificial catalytic systems capable of mirroring enzymatic active sites and achieving high catalytic capacity and substrate selectivity. A highly attractive feature of these new types of catalysts is that both the biopolymer scaffold and the transition-metal catalyst can be optimized independently by genetic, evolutionary, and/or synthetic methods. Following the successful development of metalloenzymes,^3^ DNA hybrids have recently been recognized as valuable alternatives.^4^ Indeed, since the pioneering work of Roelfes and Feringa,^5^ who were the first to combine the chirality of the double helical structure of st-DNA with catalytically active metallic cofactors to catalyze a Diels–Alder cycloaddition,^6^ the concept of DNA-based asymmetric catalysis (DAC) has been applied to an increasing number of enantioselective carbon–carbon and carbon–heteroatom bond-forming reactions, including Friedel–Crafts alkylations,^7^ Michael additions,^8^*syn*-hydrations,^9^ cyclopropanations,^10^ fluorinations,^11^ the hydrolytic kinetic resolution of epoxides,^12^ the hydroamination of nitrostyrene,^13^ the photocatalyzed [2 + 2] cycloadditions,^14^ and more recently, the inverse electron-demand hetero-Diels–Alder.^15^ Despite these achievements, the desire to tailor new DNA-based catalytic systems with improved efficiency, selectivity, and versatility remains an everlasting goal. We report here our efforts toward the development of a DNA/RNA hybrid catalytic system obtained through rational design and rounds of selection, which displays unprecedented levels of selectivity throughout the current DAC repertoire.

The challenge in DNA-based asymmetric catalysis is to be able to perform a reaction in the close vicinity of the helix, as it provides the necessary chiral microenvironment to induce enantioselectivity. To achieve this goal, two catalyst-anchoring strategies have been devised; the first one relies on a supramolecular approach, where the catalyst is bound to a DNA-specific ligand such as an intercalator or a minor groove binder, whereas the second one relies on a covalent approach where the catalyst is attached directly onto the DNA backbone. As a result, the supramolecular assembly is relatively trivial to implement; however the ligands usually do not exhibit strong base-pairing affinity, which results in a heterogeneous mixture of catalysts. In contrast, the covalent attachment allows a precise positioning of the metallic cofactor within the DNA framework, which warrants a more straightforward rationalization of the results. Several groups have followed this route and have incorporated various chelating moieties such as bipyridines,^16^ phosphines,^17^ dienes,^18^ crown ethers,^19^ salens,^20^ and imidazoles;^21^ however, this strategy lacks modularity as well as practicality, as it relies either on the postmodification of an oligonucleotide (ON) or on the direct incorporation of a modified phosphoramidite. In this context, our group recently contributed to the effort by evaluating the influence of the groove (major vs minor) on the selectivity of the challenging Cu^II^-catalyzed Friedel–Crafts alkylation/enantioselective protonation of pro-chiral acyl thiazoles in water.^22^ By fine-tuning the chiral microenvironment around the ligand cofactor, we were able to develop a much more general method that displayed unprecedented levels of selectivity on a broader range of substrates. Together, these results highlight the huge potential of the covalent anchoring mode in the development of DNA-based catalysts.

Following this initial incursion into the use of covalently attached DNA-based catalysts,^22^ our goal rapidly merged into rationally designing a novel biohybrid catalyst that would exhibit high levels of enantioselectivity on the broadest range of transformations. To achieve this goal, we opted for an SAR-type approach aided by model studies to predict and ultimately unveil the optimum catalytic system. This rational approach allows a precise study of the positioning of the ligand within the double helix and provides structural insights on the various parameters that govern the selectivity.

## Results and Discussion

We initiated this study by first focusing on the development of a general method to build ON conjugates where one finely optimized sequence could be easily coupled to any metallic cofactor through a unique anchoring point to generate a “universal” toolbox. To achieve this goal, we opted for the use of TFA-protected l- and d-serinol phosphoramidites to allow postfunctionalization on the primary amine upon cleavage of the *N*-protecting group (Table 1).^23^ As for the design of the optimum DNA sequence, we took on board the observations made by Roelfes and Feringa who reported the strong influence of small self-complementary DNA sequences on both the enantioselectivity and the reaction rate.^15^ They notably observed that 12-mers containing G-tracts lead to the highest levels of selectivity. With this in mind, we started our screening with 5′-GCCAGC**S**_**L/D**_GACCG-3′ (**ODN1**), where **S**_**L/D**_ corresponds to either (l)-Ser (**S**_**L**_) or (d)-Ser (**S**_**D**_). Additionally, two alternative model sequences were used: the AT-rich 5′-GTAGAT**S**_**L**_AGTAG-3′ (**ODN2**) and the hybrid 5′-GTATGA**S**_**L**_CACTG-3′ (**ODN3**).

**Table 1:**
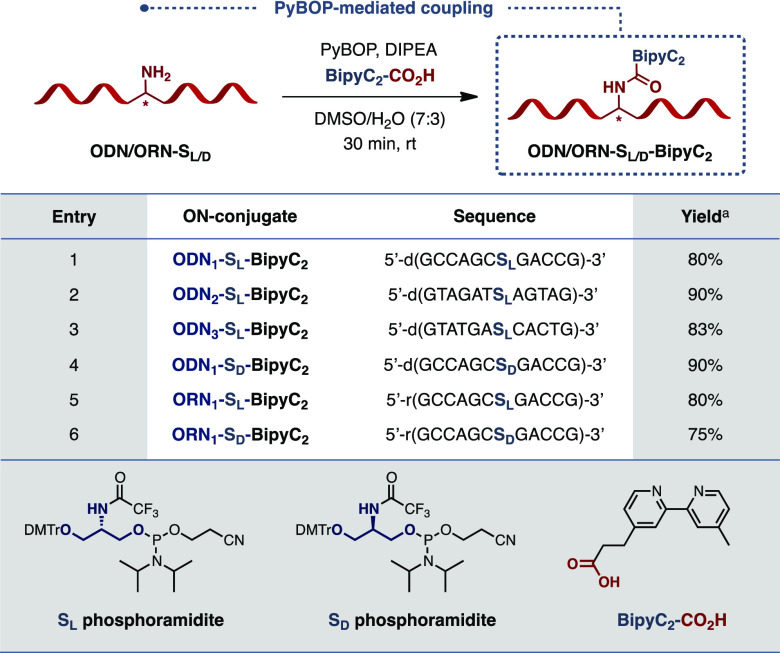
PyBOP-Mediated Coupling of BipyC_2_–CO_2_H with S_L/D_-Modified Oligonucleotides

a Isolated yield. [PyBOP = benzotriazol-1-yl-oxytripyrrolidino-phosphonium hexafluorophosphate].

To prepare these sequences and ultimately have a straightforward access to a wide range of ON conjugates, we started by developing an efficient method for the coupling of Ser(NH_2_)-oligonucleotides with CO_2_H-containing ligands. Seitz and co-workers previously used *N*-hydrosuccinimic ester as coupling partner, but their method required a large excess (100 equiv.) of the reagent and long reaction times.^23^ Moreover, the activated esters needed to be synthesized and purified prior to the coupling. To circumvent these drawbacks, we envisaged the use of PyBOP.^24^ Indeed, this phosphonium coupling reagent generates stable intermediates when coupled to carboxylic acids and operates well in aqueous media.^25^ A first set of reactions was therefore performed using **Bipy-C**_**2**_-**COOH** as a model bipyridine-based carboxylic acid and our lead serinol-modified DNA (**ODN1**) (see the Supporting Information for full details). Interestingly, when running the reaction with as little as 4 equiv. of carboxylic acid and 24 equiv. of Hunig’s base in a 7:3 DMSO/H_2_O mixture for 30 min, we were able to isolate the desired coupled product in 80% yield (Table 1, entry 1).

Encouraged by this result, we applied these conditions to our Ser(NH_2_)-modified DNA and RNA sequences. Once again, excellent coupling efficiencies were obtained with yields ranging from 75 to 90% (Table 1, entries 2–6). It is worth pointing out that the l- or d-nature of the serinol moiety did not impact the efficacy of the coupling. Moreover, the reactions produced no side products, rendering the method particularly attractive as an alternative to all the existing postsynthetic coupling methods reported so far. Most importantly, this new strategy allows the *in situ* activation and coupling of virtually any carboxylic acid-containing compound with both DNA and RNA sequences, thus providing a particularly useful handle to incorporate virtually any catalyst onto the ON framework; a necessary feature en route to developing our ON-based catalyst toolbox.

With this initial set of bipyridine-modified ON in hand, we next turned our attention to the evaluation of their catalytic activity. We thus selected two sequences bearing the model bipyridine modification on the central serinol unit, **ODN1** and **ORN1** (5′-GCCAGC**S**_**L**_GACCG-3′), and paired them with complementary DNA and RNA strands containing a propyl linker (noted **C3**) facing the serinol modification and incorporated using a phosphoramidite derived from 1,3-propanediol. As a benchmark reaction we chose the asymmetric Cu^II^-catalyzed Friedel–Crafts alkylation between α,β-unsaturated-2-acyl imidazole^26^**1a** and 5-methoxyindole **2a** (Table 2). Interestingly, all the reactions led to full or quasi-full conversion of the starting acyl imidazole and all four duplexes favored the formation of the same major enantiomer (Table 2, entries 1–4).

**Table 2:**
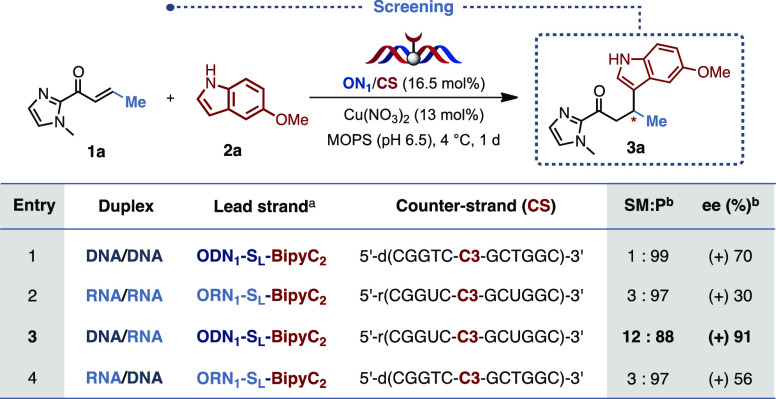
Initial Screening

a ODN_1_–S_L_ = 5′-d(GCCAGCS_L_GACCG)-3′, ORN1-S_L_ = 5′-r(GCCAGCSLGACCG)-3′.

b Determined by chiral HPLC.

As a general trend, the nature of the oligonucleotide strongly influenced the enantioselectivity. Indeed, while the efficacy of the double stranded DNA and RNA oligonucleotides appeared relatively consistent with the previous reports,^7^ the levels of enantioselectivity achieved with the hybrid duplexes were particularly striking. Notably, the use of the DNA/RNA double strand allowed a remarkable increase in selectivity to up to 91% ee (Table 2, entry 3), clearly outmatching the ones obtained with our DNA or RNA duplexes (Table 2, entries 1 and 2). As for the RNA/DNA duplex, the selectivity induced, albeit moderate, was still markedly superior to the corresponding RNA duplex (Table 2, entry 4). Although these results confirmed the superiority of the DNA/RNA hybrids, they were rather unexpected considering the precedent reported in the literature.^6b^ Nonetheless, this difference in selectivity can reasonably be associated with the covalent anchoring itself and/or to the structure adopted by the different helices and in particular to the width and depth of the corresponding duplexes. Indeed, while DNA duplexes adopt a B-type helix structure with 10 base pairs per helix rotation, it has been demonstrated that DNA/RNA hybrid duplexes share the A-form parameters leading to a narrower and deeper major groove and a wider and shallower minor groove.^27^ This, however, prompted yet another question regarding the difference in selectivity observed with the RNA duplex compared to the DNA/RNA hybrids, as they both adopt an A-type conformation. Nonetheless, as DNA/RNA hybrids exhibit a significantly larger bend compared to both DNA and RNA duplexes, we hypothesized that the greater bend allows the covalently attached ligand to be positioned in a more favored chiral microenvironment.

**Figure 1:**
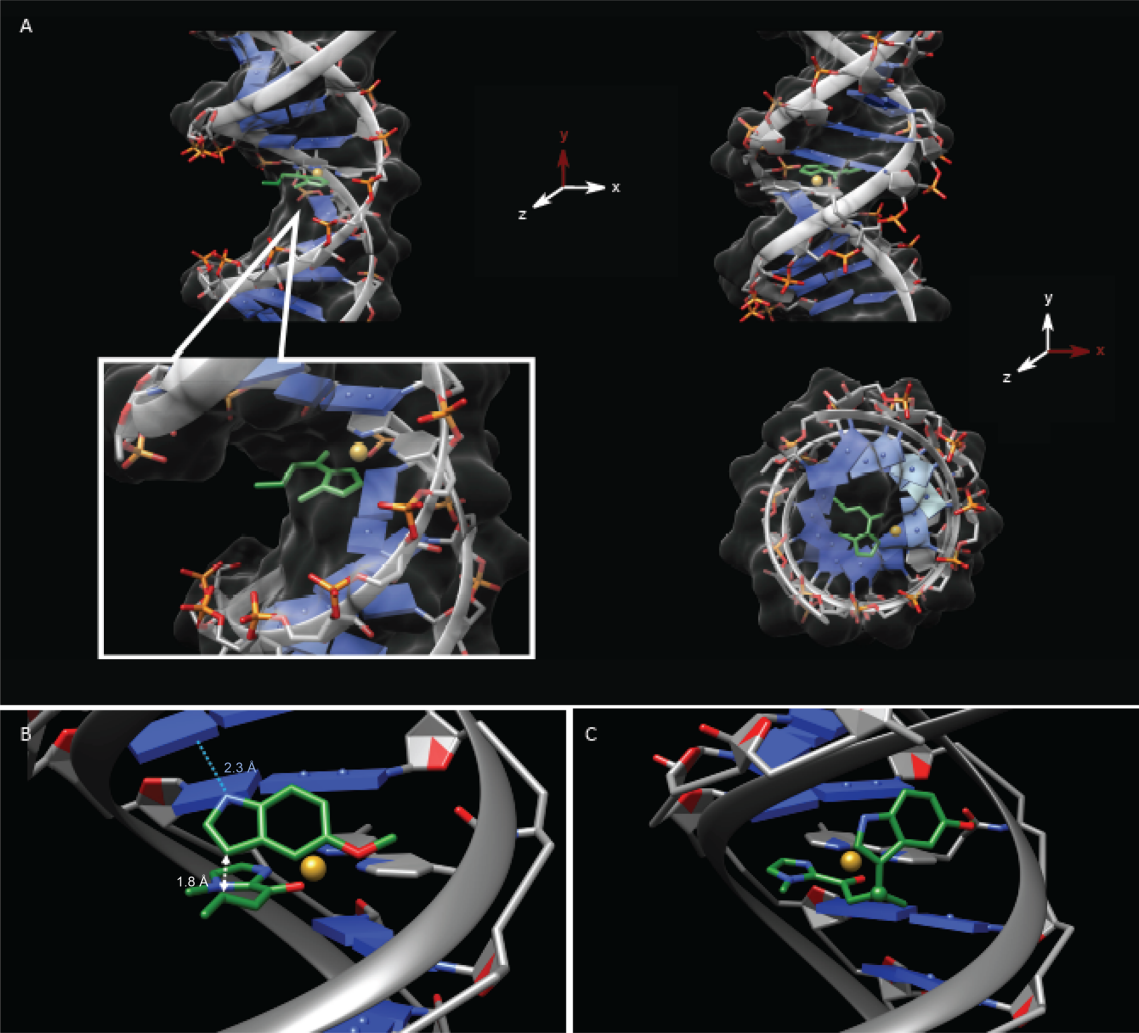
Atomistic model of the DNA/RNA duplex, **ODN1-S**_**L**_**-BipyC**_**2**_, obtained via a molecular mechanical approach. (A) Overall 3D structure of the DNA/RNA complex with the bipyridine, and the α,β-unsaturated-2-acyl imidazole (green) and the copper atom (orange); the inset panel focuses on the binding site. (B) Docked configuration for the acyl imidazole and the 5-methoxyindole. The atoms involved in the reaction are highlighted by the white arrow (distance 1.8 Å) and the hydrogen bond between the 5-methoxyindole NH and uracil U9 is highlighted by the blue dashed line. (C) Predicted configuration of the product, shown in green.

To gain a better understanding of the key parameters that are at stake, we decided to take an approach guided by structure-based modeling and simulation. Our all-atom molecular dynamics (MD) simulations showed that **BypiC**_**2**_ intercalated between the adjacent bases when bound to the duplex. With this in mind, we next evaluated which orientation the ligand was more likely to adopt. Two options were possible; one wherein the interaction with the copper occurs in the major groove (the stacked conformation), and one wherein the interaction with the copper occurs in the minor groove (the flipped conformation). Our molecular mechanical calculations not only showed that the stacked conformation was favored due to stabilizing π–π interactions, they also unveiled a pocket within the major groove of the duplex (Figure 1A). In contrast, the calculations made on the flipped conformation showed it did not generate a binding pocket spacious enough to bind the copper and was therefore discarded. The use of molecular docking in conjunction with molecular-mechanical energy calculations determined that the *R* enantiomer was more likely to be formed because of a stabilizing H-bond with uracil U9 on the RNA counter-strand (Figure 1B, C and Figure S1). Interestingly, this interaction was not observed in the DNA/DNA and RNA/DNA systems.

We also assessed reaction energetics by performing single-point calculations on the system using quantum mechanical calculations with continuum solvation. The results obtained on both the favored and the unfavored configuration of the reactant bound to the catalyst support the formation of the *R* enantiomer (Δ*E* = 31 kcal/mol) in line with the molecular-mechanical results (see Table S1 for more details).

Another important parameter was the intrinsic dynamics of C8, which appeared to be crucial for the formation of the catalytic pocket (see Figures S2–S5 for more details). Indeed, the hydrogen bond between the NH and O4 is only formed when C8 moves away from the 5-methoxyindole, resulting in a steric clash between the ligand and the N4 of C5, which is not observed with the RNA/RNA duplex.

As the model study showed the superiority of the DNA/RNA duplex over the other systems, we decided to make several structural modifications to fine-tune the catalytic pocket. Although the modification of the linker’s length was relatively innocuous in the case of the l-serinol (Table 3, entries 1–3), the use of d-serinol led to particularly striking discrepancies and showed that the flexibility of the linker could be beneficial for the accommodation of the ligand within the double helix (Table 3, entries 4–6). We investigated the effect of the linker on the architecture of the catalytic site and found that the size of the spacer affected the solvent-accessible surface area (SASA), the electrostatic (Coulombic) potential of the catalytic site, and the planarity of the bipyridine ring system (see Figure S6 for more details). Indeed, the shorter linker (**BipyC**_**0**_) has an amide bond that exhibits a trans conformation, which reduces the negatively charged electrostatic potential “patch”. This affects the binding pose and interaction energy of the imidazole and is likely contributing to the improved conversion rate observed compared to the bipyridines bearing a longer linker (**BipyC**_**2**_ and **BipyC**_**3**_).

**Table 3:**
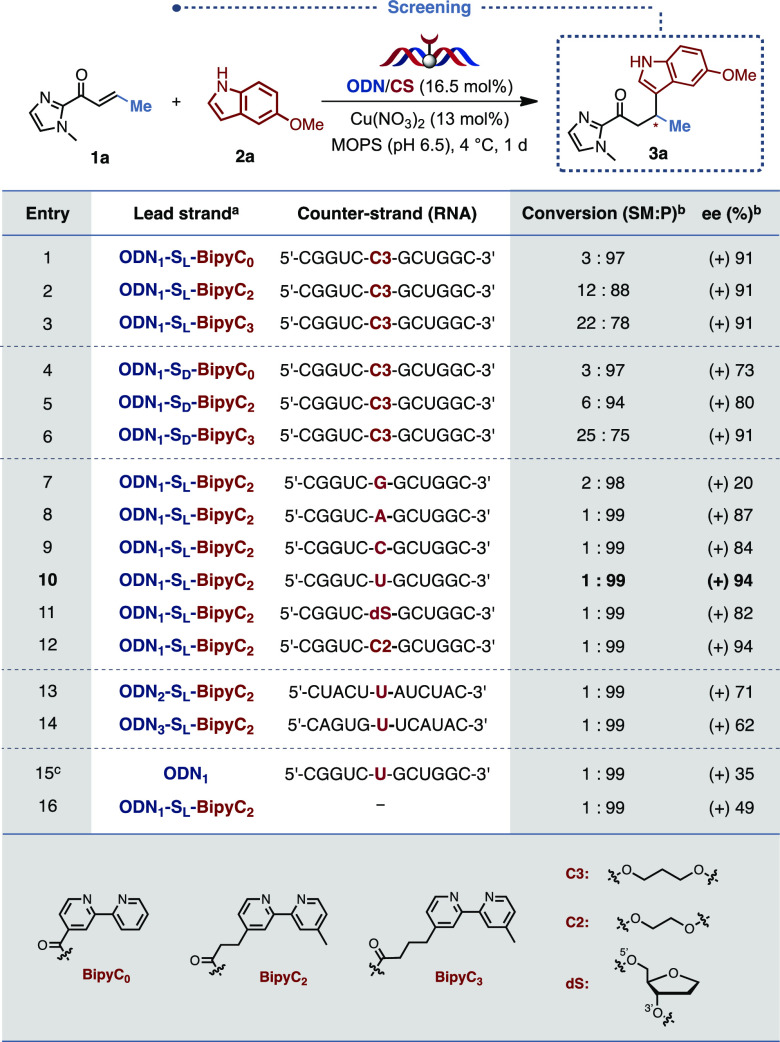
Complementary Screening

a ODN_1_–S_L/D_ = 5′-d(GCCAGCS_L/D_GACCG)-3′, ODN_2_–S_L_ = 5′-d(GTAGATS_L_AGTAG)-3′, ODN_3_–S_L_ = 5′-d(GTATGAS_L_CACTG)-3′.

b Determined by chiral HPLC.

c Cu(II)/dmbpy was used as catalyst (13 mol%).

The influence of the base facing the bipyridine ligand was also evaluated. Indeed, our simulations showed that this parameter was crucial, as a potential interaction between the indole and the facing base could lead to a preorganization of the substrate within the catalytic pocket and result in an improved face-discrimination. Interestingly, **G** appeared to have a detrimental effect on the ee value (Table 3, entry 7), whereas all the other canonical bases led to improved selectivities, with up to 91% ee obtained with the **C3** spacer (Table 3, entry 2) and an unprecedented 94% ee with **U** (Table 3, entry 10). A similar result was obtained with the ethyl linker **C2** (Table 3, entry 12), yet we opted to pursue our study with the canonical and much cheaper base **U**. Most importantly, upon docking the substrates within the simulated active site, we observed that 5-methoxyindole **2a** could bind to the uridine carbonyl *via* H-bonding. Our model also explains the unusual drop in selectivity observed in Table 3, entry 7. Indeed, **G** present on the counter-strand is stabilized by an intramolecular H-bond between N2 of the guanosine and OP2 of the backbone. This stabilizing interaction changes the architecture of the binding site and favors the approach of the 5-methoxyindole from the “bottom” rather from the “top” side, leading to the formation of the *S*-enantiomer (see Figure S7 for more details). A sequence-dependence screening confirmed that the GC-rich sequence gave the best selectivities compared to the AT/U-rich sequence (Table 3, entries 13 and 14). A control experiment using a nonmodified sequence and 4,4′-dimethylbipyridine (Table 3, entry 15) confirmed the superiority of the covalent approach when catalysis is performed with small sequences. Finally, the absence of a complementary strand does not prevent the reaction from happening but leads to a lower selectivity (Table 3, entry 16).

Melting temperatures (*T*_m_) and Δ*H*_s_ were also measured to potentially correlate the variations in selectivity observed with the stability of the duplexes. Interestingly, the unmodified DNA/DNA (*T*_m_ = 38.3 °C) and DNA/RNA duplexes (*T*_m_ = 45.1 °C) exhibited a remarkably lower *T*_m_ and Δ*H* than the serinol-modified sequences (*T*_m_ > 51.4 °C, Table S1). This difference in *T*_m_ between the DNA/DNA and the DNA/RNA duplexes can be explained by the secondary structure of the duplexes. Indeed, the A-form duplex adopted by the DNA/RNA double strand is, as we previously stated, tighter and more condensed than the B-form, which generally leads to higher *T*_m_. Nonetheless, upon addition of 4,4′-dimethylbipyridine, the *T*_m_ did not drastically evolve, whereas an increase of the Δ*H* was observed, showing a clear destabilization of the duplex formation dynamics. In contrast, the serinol-modified sequences exhibit a higher *T*_m_ and Δ*H*, which seems to indicate a remarkable stabilization of the corresponding duplexes by the bipyridine conjugation. This is all the more remarkable that these modified sequences also lead to considerably higher enantioselectivities in comparison to the unmodified ones, therefore establishing an interesting correlation between the inherent stability of the duplexes and the selectivity observed. However, it is also important noting that the various serinol-modified ON conjugates induce different selectivities, which clearly shows that other parameters also come into play. For example, the serinol modification and the facing base, which can both bind to the copper, can potentially confer to the formed duplex a higher stability and a lower flexibility. Although such parameters cannot be properly visualized here, the increased Δ*H* for the uridine-containing sequence might be an illustration of these different interactions.

**Figure 2:**
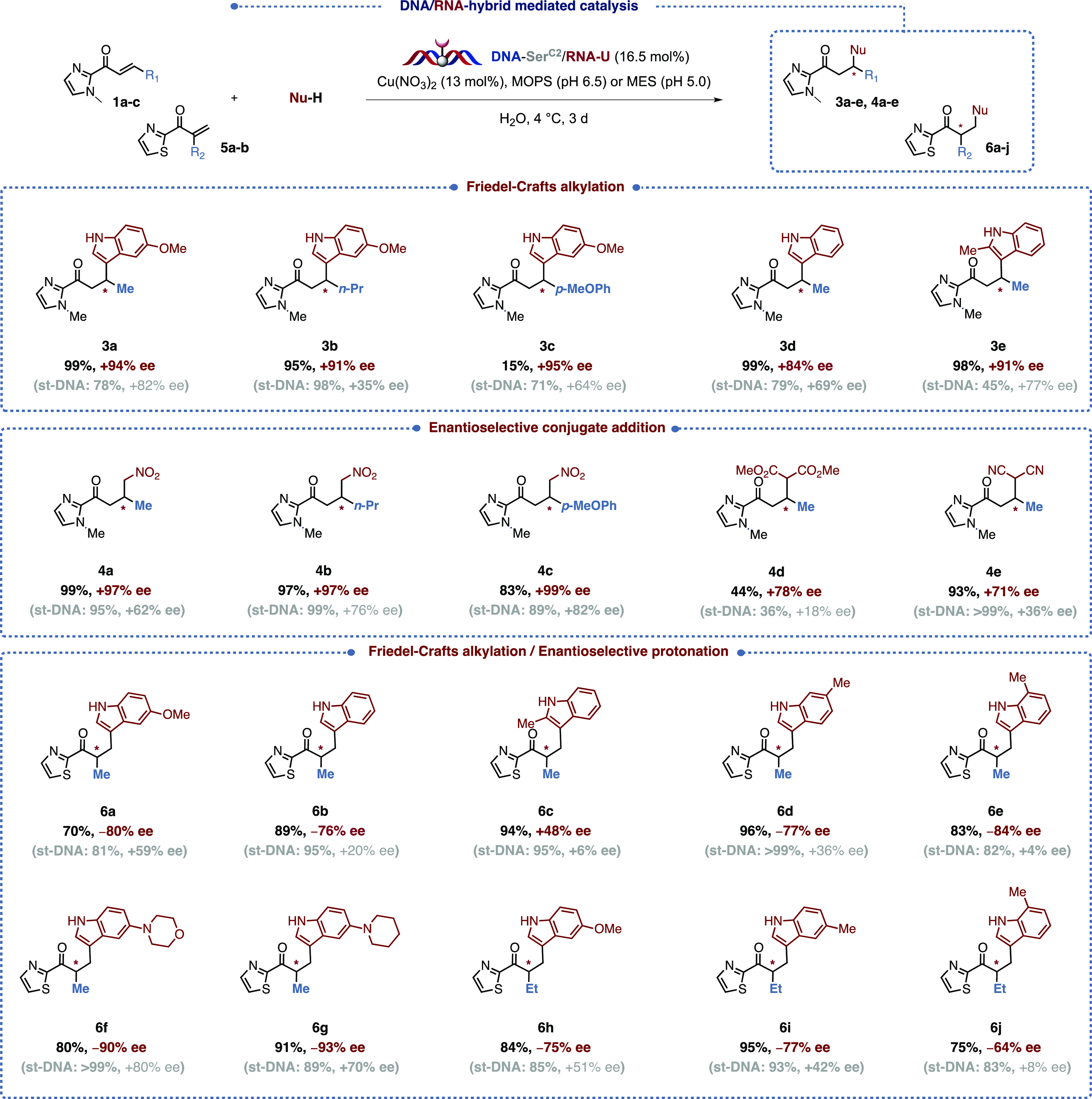
DNA/RNA hybrid-mediated catalysis: scope.

To prove the versatility of our DNA/RNA hybrid catalyst and compare its efficacy, we decided to test it on a wider range of transformations (Figure 2). We naturally turned our attention toward the key reactions reported in the field, namely, the Friedel–Crafts alkylation, the conjugate addition of dimethyl malonate, malonitrile and nitromethane, and the sequential Friedel–Crafts alkylation/asymmetric protonation. All the reactions were run in a buffered medium (MOPS buffer pH 6.5 or MES buffer pH 5.0) using 16.5 mol% double-stranded ON and 13 mol% Cu(NO_3_)_2_. All the results were compared to the ones obtained using st-DNA and the Cu^II^-dmbpy complex under otherwise identical conditions.

To our delight, the DNA-RNA duplex appeared to be much more selective independently of the reaction chosen. In the Friedel–Crafts alkylations (**3a**–**e**), for example, our ON-conjugate clearly outperformed st-DNA, with ee values ranging from 84 to 95% when the latter induced selectivities ranging from 35 to 82%. A similar trend was also observed in the case of the conjugate additions of nitromethane (**4a**–**c**, 97–99% ee *vs* 62−82% ee), dimethyl malonate (**4d**, 78% ee *vs* 18% ee), and malonitrile (**4e**, 71% ee *vs* 36% ee). The results were even more striking in the Friedel–Crafts alkylation/asymmetric protonation of α,β-unsaturated-2-acyl thiazoles in water, a reaction that is particularly challenging as it relies on the selective protonation of the transient enolate intermediate that is generated upon addition of the nucleophile. Moreover, the small size of the proton and the potential racemization issues that can occur under thermodynamic control are two factors that can plummet the selectivity as well.^28^ However, biohybrid catalysts are remarkably appropriate to carry out this type of transformation as naturally occurring macromolecules such as DNA possess a well-organized network of water molecules that could, if properly oriented, be the source of proton this transformation requires. Roelfes and co-workers^29^ and later our group^22^ managed to get moderate to good levels of selectivity using a supramolecular and a covalent approach, respectively, however the present DNA/RNA ON-conjugate strategy clearly outperformed the previous methods affording excellent yields and unprecedented levels of selectivity throughout (up to 93% ee). As a matter of fact, our DNA/RNA hybrid also happens to be more effective than the LmrR-based artificial metalloenzyme recently reported in the literature.^30^ Beyond the high ees, we also observed an inversion of the selectivity in almost all cases, indicating an alternative binding mode to the metallic cofactor. This trend was not observed in the other transformations, which leads us to believe that this phenomenon arises from the very specific and efficient chiral network of water molecules surrounding the catalytic site. The best selectivities were obtained with the morpholine and the piperidine-substituted derivatives **6f** (90% ee) and **6g** (93% ee), a trend that was also observed by Roelfes and co-workers and which seems to arise from the binding of the indole with the oligonucleotide as showcased in our previous simulation.

## Concluding Remarks

During the past decade, the use of DNA as a chiral inducer has met growing success. The concept has been applied to a wide variety of reactions affording high levels of enantioselectivity. However, the major use of the supramolecular assembly strategy has demonstrated several limitations, whereas the use of ON-conjugates stood out as an efficient alternative allowing a precise positioning of the metallic cofactor within the sequence. Using TFA-protected serinol phosphoramidites and PyBOP as an activating reagent, we were able to develop an effective method for the rapid generation of libraries of catalytically active ON-conjugates. These modified oligonucleotides were obtained in high yields and without the need of large excess of reactant. The rational design of the newly synthesized hybrid catalysts, followed by their screening, allowed us to uncover the huge potential of DNA/RNA chimeras yielding unprecedented levels of enantioselectivity throughout the DAC repertoire. More importantly, the use of serinol-modified sequences legitimated to envision a bright future for biohybrid catalysis with the possibility to expand the repertoire of DNA-based asymmetric catalysis.

## Supporting Information Available

Details of molecular simulations protocol, synthetic procedures, and characterization data (PDF)
Molecular dynamics simulations of DNA/DNA (AVI)
Molecular dynamics simulations of DNA/RNA (AVI)
Molecular dynamics simulations of RNA/DNA (AVI)
Molecular dynamics simulations of RNA/RNA (AVI)

## Supplementary Material

au2c00271_si_001.pdf

au2c00271_si_002.avi

au2c00271_si_003.avi

au2c00271_si_004.avi

au2c00271_si_005.avi
