# Supplementary material for: New Benchmark in DNA-Based Asymmetric Catalysis: Prevalence of Modified DNA/RNA Hybrid Systems
Source: JACS Au. 2022 Aug 2;2(8):1910–7. doi: 10.1021/jacsau.2c00271 (PMC9400053; doi:10.1021/jacsau.2c00271)
Supplement: Supplementary file 1 — au2c00271_si_001.pdf [file au2c00271_si_001.pdf]

A new benchmark in DNA-based asymmetric catalysis: Prevalence of modified DNA/RNA hybrid systems

Nicolas Duchemin,[†,‡] Sidonie Aubert,[†] João V. de Souza,[f] Lucas Bethge,[‡] Stefan Vonhoff,[‡] Agnieszka K. Bronowska,[f] Michael Smietana,[,§] and Stellios Arseniyadis*[,†]*

[†]*Queen Mary University of London, Department of Chemistry, Mile End Road, E1 4NS, London, United Kingdom*

[f] Chemistry – School of Natural and Environmental Sciences, Newcastle University, NE1 7RU Newcastle, *United Kingdom*

[‡] *NOXXON Pharma AG, Max-Dohrn-Strasse 8-10, 10589 Berlin, Germany*

[§]*Institut des Biomolécules Max Mousseron, Université de Montpellier, CNRS, ENSCM, 1919 Route de Mende, 34095 Montpellier, France*

Supporting Information

Table of contents

I. GENERAL CONSIDERATIONS S3

II. A NEW COVALENT APPROACH BASED ON SERINOL-DERIVED BASE SURROGATE S5

A. PREPARATION OF THE D-SERINOL PHOSPHORAMIDITE S7

B. PREPARATION OF THE L-SERINOL PHOSPHORAMIDITE S11

C. PREPARATION OF BIPYRIDINE DERIVATIVES S15

D. SERINOL-MODIFIED OLIGONUCLEOTIDE SYNTHESIS AND COUPLING REACTIONS S19

III. PREPARATION OF STOCK SOLUTIONS S29

IV. DNA-CATALYSED REACTIONS: FRIEDEL-CRAFTS REACTIONS, MICHAEL ADDITIONS, AND SEQUENTIAL FRIEDEL-CRAFTS ALKYLATION/ENANTIOSELECTIVE PROTONATION S30

A. PREPARATION OF STARTING MATERIALS S30

B. PREPARATION OF RACEMIC PRODUCTS S42

C. DNA-CATALYSED REACTIONS WITH SERINOL-MODIFIED OLIGONUCLEOTIDES AND ST-DNA S44

D. DESCRIPTION OF PRODUCTS S48

V. COMPUTATIONAL MOLECULAR MODELING .... SERREUR ! SIGNET NON DÉFINI.

I. General considerations

Reactions were conducted under a positive pressure of dry nitrogen or argon in oven-dried or flame-dried glassware, and at ambient room temperature, unless specified otherwise. Anhydrous solvents were either obtained from commercial sources or dried with a MBRAUN Solvent Purification System SPS-800. Petroleum ether refers to the 40-60 °C boiling fraction. Commercially available chemicals were used as purchased from Sigma Aldrich, Alfa Aesar, Fluorochem or Acros Organics. **DNA-catalysed experiments** were conducted in 500 µL Eppendorf® safe-lock tubes placed in a VWR Thermal Shake *lite* in a cold room, at 4 °C. **Evaporations** were carried out on Büchi rotavapors under reduced pressure with a bath temperature of 35-40 °C using a dry ice condenser. Further drying of pure compounds was ensured by exposure to high vacuum for a few hours. **Analytical TLC** was performed with Merck silica gel plates, pre-coated with silica gel 60 F254 (0.2 mm). Visualisation was effected by quenching of UV fluorescence (λ_{max} = 254 nm or 360 nm) and by staining with *p*-anisaldehyde, potassium permanganate or vanillin TLC stain solutions, followed by heating. **Flash column chromatography** employed VWR (230–400 mesh) silica gel. **Preparative TLC** was performed with Analtech silica gel plates, pre-coated with silica gel 60 F254 (0.5 or 1.0 mm). **NMR spectra** were recorded at 298 K using a Bruker AVANCE 400 spectrometer. ^{1}H NMR spectra were recorded at 400 MHz and residual solvent peaks were used as an internal reference ($CHCl_3$ δ 7.26, Methanol-d_4 δ 3.31, DMSO-d_6 δ 2.50, Acetone-d_6 δ 2.05). Data are reported as follows: chemical shift in ppm, multiplicity (s = singlet, br s = broad singlet, d = doublet, t = triplet, q = quartet, m = multiplet or overlap of non equivalent resonances), coupling constants, and integration. ^{13}C NMR spectra were recorded at 101 MHz and residual solvent peaks were used as an internal reference ($CHCl_3$ δ 77.16, methanol-d_4 δ 49.00, DMSO-d_6 δ 39.52, acetone-d_6 δ 29.08). Data are reported as follows: chemical shift in ppm, multiplicity deduced from DEPT experiments (CH_3, CH_2, CH and C_q). ^{31}P NMR spectra were recorded at 162 MHz. ^{19}F NMR spectra were recorded at 377 MHz. **IR spectra** were recorded on a Perkin Elmer Spectrum 65 FT-IR spectrometer and are reported in frequency of absorption at the peak maximum (cm^{-1}). **Melting points** were determined in open glass capillaries and are uncorrected. **Low resolution mass spectra** were recorded on an Agilent 1100 series LC-MS (with a 6310 ion trap) under electrospray ionisation (ESI). **High resolution mass spectra** were recorded on a Waters SYNAPT G2-Si High

Definition Mass Spectrometry system equipped with an Acquity UPLC BEH C18 column (2.1 x 50 mm; 130 Å) using a solvent gradient (0->100% Acetonitrile in Water + 0.1% Formic acid) in positive electrospray ionisation (ESI+) mode. The instrument was tuned using a Leucin Enkephalin mix to optimum resolution and signal intensity and was calibrated using a Waters Major Mix IMS/ToF in a range of m/z 50-1200. **High-pressure liquid chromatography** analysis was carried out on an Agilent 1100 system, using a mixture of *n*-hexane and isopropyl alcohol with Chiralpak columns (250 x 4.6 mm; 5 µm). The sign before the ee's values is arbitrary evaluated. **The syntheses of the oligonucleotides** were performed on solid support using an automated DNA synthesiser (Applied Biosystems 394 or Äkta oligopilot plus 10). Crude oligonucleotides were purified by ion exchange chromatography. Pure oligonucleotides were analysed by RP-HPLC, LC-MS and lyophilised from water. **LC-MS** were recorded on Water 2796 HPLC / Micromass triple quad MS using either set up 1 (Waters UPLC BEH C18 column, 1x50mm, 1.7µm particles. Buffer A: 100 mM HFIP, 10 µM EDTA, 10mM TEA in water. Buffer B: 100 mM HFIP, 10 µM EDTA, 10 mM TEA in methanol. Gradient: 0 to 30% B in 4 min) or set up 2 (Phenomenex Gemini C6 Phenyl column, 2 x 50mm, 3 µm particles, 110A. Buffer A: 100 mM HFIP, 10 µM EDTA, 10 mM TEA in water. Buffer B: 100 mM HFIP, 10 µM EDTA, 10 mM TEA in methanol. Gradient: 0 to 100% B in 6 min). **RP-HPLC** were recorded on Water 2796 HPLC using a Waters UPLC BEH C18 column, 2.1 x 30 mm, 1.7 µm particles, 130A. Buffer A: 100 mM TEAA in water. Buffer B: 100mM TEAA in 95% acetonitrile. Gradient: 0 to 60% B in 5 min. **λ Quantitation of DNAs and T_m experiments** were performed on a Varian Cary 300 Bio λ/Visible by measuring absorbance at 260 nm. Oligonucleotides were diluted in 20 mM cacodylate buffer with 100 mM NaCl for T_m experiments.

II. A new covalent approach based on Serinol-derived base surrogate

General procedure A: Methylation of serine

Acetyl chloride (2.70 equiv.) was added dropwise to cold methanol (C = 0.67 M), charged in an oven-dried 500 mL round-bottom flask under an argon atmosphere. After 10 min, D-serine (1.00 equiv.) was added in one portion to the solution and the reaction mixture was refluxed overnight. After completion of the reaction (monitored by TLC), the solvent was removed under reduced pressure to afford the product, used without purification.

General procedure B: Trifluoroacetylation of amine

An oven-dried round-bottom flask under an argon atmosphere was charged with a solution of serine methyl ester hydrochloride (1.00 equiv.) in methanol (C = 0.32 M), followed by the successive additions of triethylamine (2.50 equiv.) and ethyl trifluoroacetate (1.70 equiv.). The resulting mixture was stirred at room temperature until completion of the reaction (monitored by TLC) and then concentrated under reduced pressure. The crude residue was eventually purified by flash column chromatography on silica gel to afford the desired product.

General procedure C: Tritylation of alcohol

An oven-dried round-bottom flask under an argon atmosphere was charged with *N*-(2,2,2-trifluoroacetyl)-serine methyl ester (1.00 equiv.) and pyridine (C = 0.53 M), followed by a solution of 4,4'-dimethoxytrityl chloride (20.1 g, 59.4 mmol, 1.10 equiv.) in pyridine (C = 0.50 M) through an addition funnel. The resulting mixture was stirred overnight at room temperature until completion of the reaction (monitored by TLC) and then concentrated under reduced pressure. The crude was diluted in a saturated aqueous solution of $NaHCO_3$ and extracted with ethyl acetate (two times). The combined organic phases were then washed with a saturated aqueous solution of NaCl, dried over anhydrous $MgSO_4$ and evaporated under reduced pressure. The crude mixture was eventually loaded on Celite® to be purified by flash column chromatography on silica gel to afford the desired product.

General procedure D: Reduction

An oven-dried round-bottom flask under an argon atmosphere was charged with a solution of *N*-(2,2,2-trifluoroacetyl)-*O*-dimethoxytritylated-serine methyl ester (1.00 equiv.) in absolute ethanol (C = 0.25 M), followed by the subsequent additions of lithium chloride

(12.0 equiv.) and sodium borohydride (12.0 equiv.) in portions, at 0 °C. The solution was stirred at 0 °C for 2 h and then at room temperature until completion of the reaction (monitored by TLC). The mixture was slowly quenched by adding it onto an ice bath and then extracted with ethyl acetate. The combined organic phases were washed twice with a saturated aqueous solution of $NaHCO_3$, dried over anhydrous $MgSO_4$, gravity filtered and concentrated under reduced pressure. The crude mixture was eventually purified by flash column chromatography on silica gel to afford the desired product.

General procedure E: Trifluoroacetylation of amine after reduction

An oven-dried round-bottom flask under an argon atmosphere was charged with a solution of *O*-dimethoxytritylated-serinol (1.00 equiv.) in methanol (C = 0.10 M), followed by the successive additions of triethylamine (1.10 equiv.) and ethyl trifluoroacetate (1.10 equiv.). The reaction mixture was stirred at room temperature until completion of the reaction (monitored by TLC) and then concentrated under reduced pressure. The crude mixture was dissolved in diethyl ether, washed twice with water and then twice with a saturated aqueous solution of NaCl, dried over anhydrous $MgSO_4$ and evaporated under reduced pressure to afford the desired compound. The product was used without further purification.

General procedure F: Phosphoramidite synthesis

An oven-dried round-bottom flask under an argon atmosphere was charged with *N*-(2,2,2-trifluoroacetyl)-*O*-dimethoxytritylated-serinol (1.50 g, 3.07 mmol, 1.00 equiv.), *N,N*-diisopropylethylamine (2.00 equiv.) and dichloromethane (31.0 mL). The reaction mixture was stirred at room temperature until complete dissolution of the solid, followed by the dropwise addition of 2-cyanoethyl *N,N*-diisopropyl-chlorophosphoramidite (1.50 equiv.). The mixture was stirred at room temperature until completion of the reaction (monitored by TLC), and quenched by addition of a saturated aqueous solution of $NaHCO_3$. The organic phase was washed twice with the saturated aqueous solution of $NaHCO_3$, followed by a saturated aqueous solution of NaCl. The resulting organic phase was dried over anhydrous $MgSO_4$, gravity filtered and concentrated under reduced pressure. The crude mixture was eventually purified by flash column chromatography with silica gel to afford the desired product.

A. Preparation of the D-serinol phosphoramidite

D-Serine methyl ester hydrochloride (i)

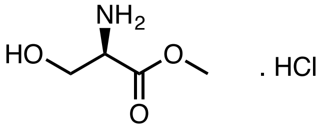

MW (g/mol): 155.58 **Molecular formula:** $C_4H_{10}ClNO_3$

Synthesised according general procedure A using D-serine (15.0 g, 143 mmol, 1.00 equiv.) The desired D-serine methyl ester hydrochloride was obtained as a white solid (22.2 g, 143 mmol, quantitative yield) and used without purification. The spectroscopic data of the product were identical with those reported in the literature.[1]

^{1}H NMR (400 MHz, Methanol-*d*$_4$) δ 4.15 (t, *J* = 4.0 Hz, 1H), 4.05-3.92 (m, 2H), 3.85 (s, 3H).

^{13}C NMR (101 MHz, Methanol-*d*$_4$) δ 169.4, 60.7, 56.1, 53.7.

***N*-(2,2,2-Trifluoroacetyl)-D-serine methyl ester (ii)**

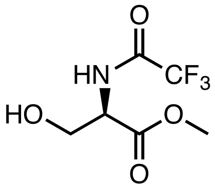

MW (g/mol): 215.13 **Molecular formula:** $C_6H_8F_3NO_4$

Synthesised according general procedure B using D-serine methyl ester hydrochloride **i** (15.0 g, 96.4 mmol, 1.00 equiv.). The desired product was obtained as yellow oil (17.6 g, 81.9 mmol, 85% yield) after purification by flash column chromatography on silica gel (Petroleum ether/Ethyl acetate, from 7:3 to 1:1). The spectroscopic data of the product were identical with those reported in the literature.[2,3]

^{1}H NMR (400 MHz, CDCl$_3$) δ 7.41 (br s, 1H, -*NH*), 4.68 (m, 1H), 4.10 (dd, *J* = 11.4, 3.3 Hz, 1H), 3.95 (dd, *J* = 11.4, 3.3 Hz, 1H), 3.82 (s, 3H).

^{13}C NMR (101 MHz, CDCl$_3$) δ 169.6, 157.4 (q, *J* = 38.0 Hz), 115.7 (q, *J* = 287.4 Hz, -*CF$_3$*), 62.2, 54.8, 53.3.

^{19}F NMR (377 MHz, CDCl$_3$) δ –75.3.

***N*-(2,2,2-Trifluoroacetyl)-*O*-dimethoxytritylated-D-serine methyl ester (iii)**

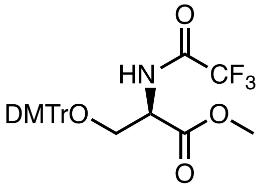

MW (g/mol): 517.50 **Molecular formula:** $C_{27}H_{26}F_3NO_6$

Synthesised according general procedure C using compound **ii** (11.4 g, 53.0 mmol, 1.00 equiv.). The desired product was obtained as a pale yellow oil (27.4 g, 53.0 mmol, quantitative yield) after loading on Celite® and purification by flash column chromatography on silica gel (Petroleum ether/Ethyl acetate, from 90:10 to 85:15 + 1% triethylamine). The spectroscopic data of the product were identical with those reported in the literature.[3,4]

^{1}H NMR (400 MHz, Acetone-*d*$_6$) δ 7.47-7.40 (m, 2H), 7.35-7.28 (m, 7H), 6.91-6.85 (m, 4H), 4.82 (m, 1H), 3.79 (s, 6H), 3.75 (s, 3H), 3.54 (m, 2H).

^{13}C NMR (101 MHz, Acetone-*d*$_6$) δ 169.9, 159.7 (2C), 157.3 (q, *J* = 37.0 Hz), 145.7, 136.3 (2C), 130.9 (4C), 128.8 (2C), 128.6 (2C), 127.7, 113.9 (4C), 113.2 (q, *J* = 229.0 Hz, -*CF$_3$*), 87.1 , 63.3, 55.5 (2C), 54.2, 52.9.

^{19}F NMR (377 MHz, Acetone-*d*$_6$) δ –76.1.

***O*-Dimethoxytritylated-D-serinol (iv)**

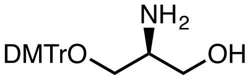

MW (g/mol): 393.48 **Molecular formula:** $C_{24}H_{27}NO_4$

Synthesised according general procedure D using compound **iii** (26.4 g, 50.1 mmol, 1.00 equiv.). The desired product was obtained as white foam (15.0 g, 38.0 mmol, 76% yield) after purification by flash column chromatography on silica gel (Dichloromethane/Methanol, from 10:0 to 9:1 + 1% triethylamine). The spectroscopic data of the product were identical with those reported in the literature.[2,3]

^{1}H NMR (400 MHz, Acetone-d_6) δ 7.48-7.44 (m, 2H), 7.38-7.27 (m, 6H), 7.23 (m, 1H), 6.93-6.86 (m, 4H), 3.88 (m, 1H), 3.78 (s, 6H), 3.61 (m, 1H), 3.51 (m, 1H), 3.25 (dd, *J* = 9.1, 4.7 Hz, 1H), 3.12 (dd, *J* = 9.1, 6.1 Hz, 1H).

^{13}C NMR (101 MHz, Acetone-d_6) δ 159.6 (2C), 146.2, 137.0, 136.9, 130.9 (4C), 129.0 (2C), 128.6 (2C), 127.5, 113.9 (4C), 86.6, 68.6, 65.0, 58.8, 55.5 (2C).

_N_-(2,2,2-Trifluoroacetyl)-_O_-dimethoxytritylated-D-serinol (v)

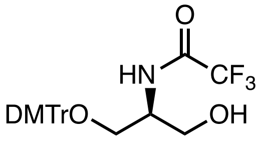

MW (g/mol): 489.49 **Molecular formula:** $C_{26}H_{26}F_3NO_5$

Synthesised according general procedure E using D-serinol derivative **iv** (2.47 g, 6.28 mmol, 1.00 equiv.). The desired product was obtained as a colourless oil (2.64 g, 5.40 mmol, 86% yield) and used without further purification. The spectroscopic data of the product were identical with those reported in the literature.[3,5]

^{1}H NMR (400 MHz, Acetone-d_6) δ 7.50-7.42 (m, 2H), 7.34-7.27 (m, 6H), 7.21 (m, 1H), 6.90-6.84 (m, 4H), 4.27 (m, 1H), 4.03 (s, 1H), 3.78 (s, 6H), 3.73 (m, 1H), 3.31 (m, 1H), 3.23 (m, 1H).

^{13}C NMR (101 MHz, Acetone-d_6) δ 159.6 (2C), 157.3 (q, *J* = 37.0 Hz), 146.1, 136.8 (2C), 130.9 (4C), 128.9 (2C), 128.6 (2C), 127.5, 113.9 (4C), 113.2 (q, *J* = 229.0 Hz, -CF_3), 86.9, 64.4, 63.2, 55.5 (2C), 53.7.

^{19}F NMR (377 MHz, Acetone-d_6) δ –76.2.

_N_-(2,2,2-Trifluoroacetyl)-_O_-dimethoxytritylated-D-serinol-(2-cyanoethyl)-diisopropylphosphoramidite (vi)

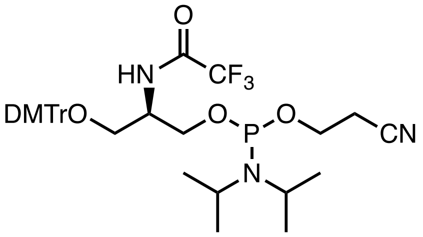

MW (g/mol): 689.71 **Molecular formula:** $C_{35}H_{43}F_3N_3O_6P$

Synthesised according general procedure F using compound **v** (1.50 g, 3.07 mmol, 1.00 equiv.). The desired product was obtained as a colourless oil (1.29 g, 1.87 mmol, 61% yield) after purification by flash column chromatography with silica gel (_n_-Hexane/Diethyl ether, 6:1 + 0.5% triethylamine). The spectroscopic data of the product were identical with those reported in the literature.[3]

^{1}H NMR (400 MHz, $CDCl_3$) δ 7.42-7.36 (m, 2H), 7.31-7.27 (m, 6H), 7.22 (m, 1H), 6.89-6.78 (m, 4H), 4.29 (m, 1H), 3.91 (m, 1H), 3.79 (br s, 7H), 3.76-3.67 (m, 2H), 3.61-3.47 (m, 2H), 3.39 (m, 1H), 3.22 (m, 1H), 2.62-2.48 (m, 2H), 1.17 (dd, _J_ = 6.8, 2.2 Hz, 6H), 1.11 (dd, _J_ = 10.2, 6.8 Hz, 6H).

^{13}C NMR (101 MHz, $CDCl_3$) δ 158.8 (2C), 157.3 (q, _J_ = 37.0 Hz), 144.6, 135.8, 135.6, 130.1 (4C), 128.1 (2C), 128.0 (2C), 127.1, 117.8, 113.4 (4C), 113.2 (q, _J_ = 229.0 Hz, -CF_3), 86.5, 61.3 (2C), 58.3, 55.4 (2C), 50.8, 43.4, 43.3, 24.8 (2C), 24.7 (2C), 21.8.

^{19}F NMR (377 MHz, $CDCl_3$) δ –76.1, –76.2 (two diastereoisomers).

^{31}P NMR (162 MHz, $CDCl_3$) δ 149.5, 147.5 (two diastereoisomers).

B. Preparation of the L-serinol phosphoramidite

L-Serine Methyl Ester Hydrochloride (vii)

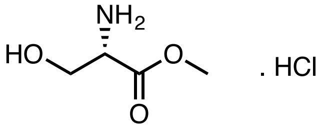

MW (g/mol): 155.58 **Molecular formula:** $C_4H_{10}ClNO_3$

Synthesised according general procedure A using L-serine (25.0 g, 238 mmol, 1.00 equiv.). The desired L-serine methyl ester hydrochloride was obtained as a white solid (37.0 g, 238 mmol, quantitative yield) and used without purification. The spectroscopic data of the product were identical with those reported in the literature.[1]

^{1}H NMR (400 MHz, Methanol-*d*$_4$) δ 4.15 (t, *J* = 3.9 Hz, 1H), 4.07-3.87 (m, 2H), 3.85 (s, 3H).

^{13}C NMR (101 MHz, Methanol-*d*$_4$) δ 169.4, 60.7, 56.1, 53.7.

***N*-(2,2,2-Trifluoroacetyl)-L-serine methyl ester (viii)**

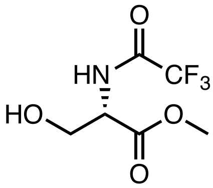

MW (g/mol): 215.13 **Molecular formula:** $C_6H_8F_3NO_4$

Synthesised according general procedure B using L-serine methyl ester hydrochloride **vii** (25.0 g, 161 mmol, 1.00 equiv.). The desired product was obtained as yellow oil (34.6 g, 161 mmol, quantitative yield) after purification by flash column chromatography on silica gel (Petroleum ether/Ethyl acetate, from 7:3 to 1:1). The spectroscopic data of the product were identical with those reported in the literature.[2,3]

^{1}H NMR (400 MHz, CDCl$_3$) δ 7.49 (br s, 1H, -*NH*), 4.66 (m, 1H), 4.08 (dd, *J* = 11.5, 3.4 Hz, 1H), 3.94 (dd, *J* = 11.5, 3.3 Hz, 1H), 3.81 (s, 3H), 2.90 (br s, 1H, -*OH*).

^{13}C NMR (101 MHz, CDCl$_3$) δ 169.7, 157.5 (q, *J* = 38.0 Hz), 115.7 (q, *J* = 287.2 Hz, -*CF$_3$*), 62.1, 54.8, 53.3.

^{19}F NMR (377 MHz, CDCl$_3$) δ –75.9.

_N_-(2,2,2-Trifluoroacetyl)-_O_-dimethoxytritylated-L-serine methyl ester (ix)

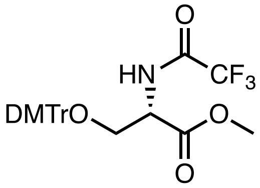

MW (g/mol): 517.50 **Molecular formula:** $C_{27}H_{26}F_3NO_6$

Synthesised according general procedure C using compound **viii** (11.6 g, 54.0 mmol, 1.00 equiv.). The desired product was obtained as a pale yellow oil (27.7 g, 53.5 mmol, 99% yield) after loading on Celite® and purification by flash column chromatography on silica gel (Petroleum ether/Ethyl acetate, from 90:10 to 85:15 + 1% triethylamine). The spectroscopic data of the product were identical with those reported in the literature.[3,4]

^{1}H NMR (400 MHz, Acetone-d_6) δ 7.47-7.39 (m, 2H), 7.33-7.28 (m, 6H), 7.24 (m, 1H) 6.91-6.86 (m, 4H), 4.81 (m, 1H), 3.79 (s, 6H), 3.75 (s, 3H), 3.61-3.47 (m, 2H).

^{13}C NMR (101 MHz, Acetone-d_6) δ 169.9, 159.8 (2C), 157.3 (q, *J* = 37.0 Hz), 145.7, 136.3 (2C), 130.9 (4C), 128.8 (2C), 128.6 (2C), 127.7, 114.0 (4C), 113.2 (q, *J* = 229.0 Hz, -CF_3), 87.1, 63.3, 55.5 (2C), 54.2, 52.9.

^{19}F NMR (377 MHz, Acetone-d_6) δ –76.2.

_O_-Dimethoxytritylated-L-serinol (x)

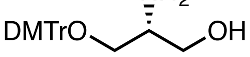

MW (g/mol): 393.48 **Molecular formula:** $C_{24}H_{27}NO_4$

Synthesised according general procedure D using compound **ix** (25.9 g, 50.0 mmol, 1.00 equiv.). The desired product was obtained as white foam (13.2 g, 33.5 mmol, 67% yield) after purification by flash column chromatography on silica gel (Dichloromethane/Methanol, from 10:0 to 9:1 + 1% triethylamine). The spectroscopic data of the product were identical with those reported in the literature.[2,3]

¹H NMR (400 MHz, Acetone-*d*$_6$) δ 7.48-7.45 (m, 2H), 7.34-7.30 (m, 6H), 7.22 (m, 1H), 6.90-6.87 (m, 4H), 3.89 (t_{app}, *J* = 7.2 Hz, 1H), 3.78 (s, 6H), 3.61 (m, 1H), 3.51 (t_{app}, *J* = 7.2 Hz, 1H), 3.26 (dd, *J* = 9.2, 4.7 Hz, 1H), 3.12 (dd, *J* = 9.2, 6.0 Hz, 1H).

^{13}C NMR (101 MHz, Acetone-*d*$_6$) δ 158.6 (2C), 145.2, 136.0, 135.9, 129.9 (4C), 127.9 (2C), 127.6 (2C), 126.5, 112.9 (4C), 85.6, 67.6, 63.9, 57.7, 54.5 (2C).

***N*-(2,2,2-Trifluoroacetyl)-*O*-dimethoxytritylated-L-serinol (xi)**

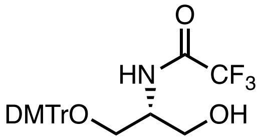

MW (g/mol): 489.49 **Molecular formula:** $C_{26}H_{26}F_3NO_5$

Synthesised according general procedure E using compound **x** (6.00 g, 15.3 mmol, 1.00 equiv.). The desired product was obtained as a colourless oil (7.11 g, 14.5 mmol, 95% yield) after purification by flash column chromatography with silica gel (*n*-Hexane/Diethyl ether, 6:1 + 0.5% triethylamine). The spectroscopic data of the product were identical with those reported in the literature.[3]

¹H NMR (400 MHz, Acetone-*d*$_6$) δ 7.48-7.43 (m, 2H), 7.35-7.27 (m, 6H), 7.22 (m, 1H), 6.90-6.84 (m, 4H), 4.27 (m, 1H), 4.05 (q, *J* = 7.1 Hz, 1H), 3.79 (s+m, 7H), 3.31 (dd, *J* = 9.3, 5.3 Hz, 1H), 3.24 (dd, *J* = 9.3, 6.2 Hz, 1H).

^{13}C NMR (101 MHz, Acetone-*d*$_6$) δ 159.6 (2C), 157.3 (q, *J* = 37.0 Hz), 146.1, 136.8 (2C), 130.9 (4C), 128.9 (2C), 128.6 (2C), 127.5, 113.9 (4C), 113.2 (q, *J* = 229.0 Hz, -*CF*$_3$), 86.9, 63.1, 61.7, 55.5 (2C), 53.7.

^{19}F NMR (377 MHz, Acetone-*d*$_6$) δ –76.3.

N-(2,2,2-Trifluoroacetyl)-O-dimethoxytritylated-L-serinol-(2-cyanoethyl)-diisopropylphosphoramidite (xii)

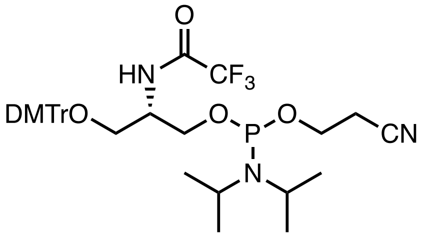

MW (g/mol): 689.71 **Molecular formula:** $C_{35}H_{43}F_3N_3O_6P$

Synthesised according general procedure F using compound **xi** (1.50 g, 3.07 mmol, 1.00 equiv.). The desired product was obtained as a colourless oil (1.27 g, 1.84 mmol, 60% yield) after purification by flash column chromatography with silica gel (*n*-Hexane/Diethyl ether, 6:1 + 0.5% triethylamine). The spectroscopic data of the product were identical with those reported in the literature.[3]

^{1}H NMR (400 MHz, CDCl$_3$) δ 7.42-7.36 (m, 2H), 7.31-7.27 (m, 6H), 7.22 (m, 1H), 6.86-6.80 (m, 4H), 4.29 (m, 1H), 3.91 (m, 1H), 3.79 (br s, 7H), 3.76-3.67 (m, 2H), 3.61-3.47 (m, 2H), 3.39 (m, 1H), 3.22 (m, 1H), 2.62-2.48 (m, 2H), 1.17 (dd, *J* = 6.8, 2.2 Hz, 6H), 1.11 (dd, *J* = 10.2, 6.8 Hz, 6H).

^{13}C NMR (101 MHz, CDCl$_3$) δ 158.8 (2C), 157.3 (q, *J* = 37.0 Hz), 144.6, 135.8, 135.6, 130.1 (4C), 128.1 (2C), 128.0 (2C), 127.1, 117.8, 113.4 (4C), 113.2 (q, *J* = 229.0 Hz, -*CF$_3$*), 86.5, 61.3 (2C), 58.3, 55.4 (2C), 50.8, 43.4, 43.3, 24.8 (2C), 24.7 (2C), 21.8.

^{19}F NMR (377 MHz, CDCl$_3$) δ –75.5, –75.6 (two diastereoisomers).

^{31}P NMR (162 MHz, CDCl$_3$) δ 147.9, 147.8 (two diastereoisomers).

C. Preparation of bipyridine derivatives

Ethyl 3-(4'-methyl-[2,2'-bipyridin]-4-yl)propanoate (xiii)

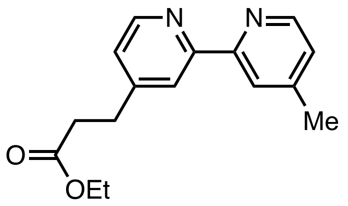

MW (g/mol): 270.33 **Molecular formula:** $C_{16}H_{18}N_2O_2$

An oven-dried round-bottomed flask was charged with lithium diisopropylamide solution (5.42 mL, 1.00 M in THF/hexanes) and cooled down to –78 °C. A solution of 4,4'-dimethyl-2,2'-bipyridine (1.00 g, 5.42 mmol, 1.00 equiv.) in dry THF (40.0 mL) was added dropwise and the reaction mixture was stirred at –78 °C for two hours. The resulting solution was cannulated dropwise to a solution of ethyl bromoacetate (0.69 mL, 6.23 mmol, 1.20 equiv.) in dry THF (10.0 mL) at –78 °C, allowed to warm up at room temperature and stirred overnight. The resulting mixture was filtrated and concentrated under reduced pressure. The crude was purified by flash column chromatography over silica gel (PE/Ethyl acetate/Et_3N = 85:10:5). The desired product was obtained as a colourless oil (741 mg, 2.74 mmol, 53% yield). The spectroscopic data of the product were identical with those reported in the literature.[6]

^{1}H NMR (400 MHz, CDCl$_3$) δ 8.53 (ddd, *J* = 16.4, 5.0, 1.25 Hz, 2H), 8.23 (d, *J* = 17.1 Hz, 2H), 7.13 (ddd, *J* = 14.3, 5.0 Hz, 2H), 4.12 (qd, *J* = 7.2, 1.4 Hz, 2H), 3.04-3.01 (m, 2H), 2.70 (td, *J* = 7.7, 1.2 Hz, 2H), 2.42 (s, 3H), 1.22 (td, *J* = 7.2, 1.5 Hz, 3H).

^{13}C NMR (101 MHz, CDCl$_3$) δ 172.3, 156.4, 155.8, 150.6, 149.2, 149.9, 148.2, 124.7, 123.7, 122.0, 121.1, 60.6, 34.6, 30.4, 21.2, 14.2.

3-(4'-Methyl-[2,2'-bipyridin]-4-yl)propanoic acid (xiv)

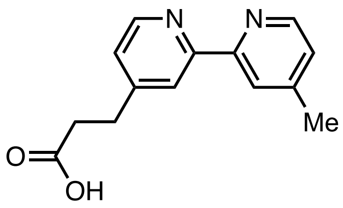

MW (g/mol): 242.28 **Molecular formula:** $C_{14}H_{14}N_2O_2$

A 50 mL round-bottomed flask was charged with ethyl 3-(4'-methyl-[2,2'-bipyridin]-4-yl)propanoate **xiii** (300 mg, 1.11 mmol, 1.00 equiv.) dissolved in THF (3.50 mL) and methanol (0.50 mL). A 2N NaOH aqueous solution (2.20 mL) was then added stirred at room temperature for two hours and at reflux overnight. The resulting mixture was concentrated under reduced pressure, dissolved in H_2O (2.00 mL) and extracted with ethyl acetate (2 x 2.00 mL). The resulting aqueous layer was acidified while cooling at 0 °C using a 25% HCl solution. The formed precipitate was collected by centrifugation, washed with water and lyophilized. The title compound was obtained as a white powder (256 mg, 1.05 mmol, 95% yield). The spectroscopic data of the product were identical with those reported in the literature.[6]

^{1}H NMR (400 MHz, DMSO-*d*$_6$) δ 12.20 (br s, 1H), 8.54 (ddd, *J* = 8.4, 4.9, 0.8 Hz, 2H), 8.24 (ddd, *J* = 11.3, 1.8, 0.9 Hz, 2H), 7.33-7.27 (m, *J* = 5.0, 2H), 7.27 (dd, *J* = 5.0, 1.6 Hz, 1H), 2.94 (t, *J* = 7.4 Hz, 2H), 2.64 (t, *J* = 7.4 Hz, 2H), 2.41 (s, 3H).

^{13}C NMR (101 MHz, DMSO-*d*$_6$) δ 174.4, 167.1, 155.1, 152.1, 148.9, 147.9, 124.8, 124.1, 121.2, 120.4, 35.9, 30.8, 22.14, 20.7.

Ethyl 4-(4'-methyl-[2,2'-bipyridin]-4-yl)butanoate (xv)

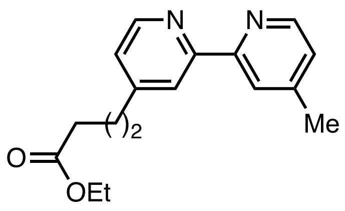

MW (g/mol): 284.36 **Molecular formula:** $C_{17}H_{20}N_2O_2$

An oven-dried round-bottomed flask was charged with lithium diisopropylamide solution (5.42 mL, 1.00 M in THF/hexanes) and cooled down to –78 °C. A solution of 4,4'-dimethyl-2,2'-bipyridine (1.00 g, 5.42 mmol, 1.00 equiv.) in dry THF (40.0 mL) was added dropwise and the reaction mixture was stirred at –78 °C for two hours. The resulting solution was cannulated dropwise to a solution of ethyl bromopropionate (0.80 mL, 6.23 mmol, 1.2 equiv.) in dry THF (10.0 mL) at –78 °C, allowed to warm up at room temperature and stirred overnight. The resulting mixture was filtrated and concentrated under reduced pressure. The crude was purified by flash column chromatography over silica gel (Petroleum ether/Ethyl acetate/Et_3N = 85:10:5). The desired product was obtained as a colourless oil (292 mg, 1.02 mmol, 19 % yield). The spectroscopic data of the product were identical with those reported in the literature.[7]

^{1}H NMR (400 MHz, $CDCl_3$) δ 8.55 (dd, *J* = 14.0, 5.0 Hz, 2H), 8.24-8.22 (m, 2H), 7.15-7.12 (m, 2H), 4.12 (q, *J* = 7.1 Hz, 2H), 2.77-2.73 (m, 2H), 2.44 (s, 3H), 2.35 (t, *J*=7.4 Hz, 2H), 2.08-2.02 (m, 2H), 1.25 (t, *J* = 7.1 Hz, 3H).

^{13}C NMR (101 MHz, $CDCl_3$) δ 173.1, 156.2, 156.0, 151.4, 149.1, 149.0, 148.1, 124.7, 123.9, 122.0, 121.3, 60.4, 34.7, 33.6, 30.4, 25.5, 21.2, 14.2.

4-(4'-Methyl-[2,2'-bipyridin]-4-yl)butanoic acid (xvi)

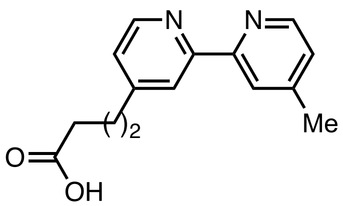

MW (g/mol): 256.31 **Molecular formula:** $C_{15}H_{16}N_2O_2$

A 50 mL round-bottomed flask was charged with ethyl 4-(4'-methyl-[2,2'-bipyridin]-4-yl)butanoate **xv** (100 mg, 0.36 mmol, 1.00 equiv.) dissolved in THF (1.2 mL) and methanol (0.20 mL). A 2N NaOH aqueous solution (0.70 mL) was then added stirred at room temperature for two hours and at reflux overnight. The resulting mixture was concentrated under reduced pressure, dissolved in H_2O (0.60 mL) and extracted with ethyl acetate (2 x 1.00 mL). The resulting aqueous layer was acidified while cooling at 0 °C using a 25% HCl solution. The formed precipitate was collected by centrifugation, washed with water and lyophilized. The title compound was obtained as a white powder (82.0 mg, 0.32 mmol, 88% yield). The spectroscopic data of the product were identical with those reported in the literature.[7]

^{1}H NMR (400 MHz, DMSO-*d*$_6$) δ 12.16 (br s, 1H), 8.54 (dd, *J* = 10.3, 4.9 Hz, 2H), 8.23 (s, 2H), 7.28 (td, *J* = 4.8, 1.6 Hz, 2H), 2.71 (t, *J* = 7.7 Hz, 2H), 2.41 (s, 3H), 2.26 (t, *J* = 7.3 Hz, 2H), 1.90-1.82 (m, 2H).

^{13}C NMR (101 MHz, DMSO-*d*$_6$) δ 174.6, 155.8, 155.6, 152.1, 149.6, 149.4, 148.4, 125.4, 124.6, 121.7, 120.9, 34.3, 33.5, 25.7, 21.2.

D. Serinol-modified oligonucleotide synthesis and coupling reactions

Representative procedure for oligonucleotide synthesis

Oligonucleotide synthesis was performed on solid support, using commercially available $O^{5'}$-dimethoxytrityl-2'-deoxyribonucleoside $O^{3'}$-phosphoramidites. Previously synthesised serinol phosphoramidites were incorporated with 5 min time coupling (0.10 M) to synthetise the desired 12-mer sequences (DMT-off synthesis): 5'-GCCAGC$\mathbf{S_{D/L}}$GACCG-3', 5'-GTAGAT$\mathbf{S_{D/L}}$AGTAG-3' and 5'-GTATGA$\mathbf{S_{D/L}}$CACTG-3'. After synthesis of these modified oligonucleotides, CPG beads were washed with a 0.20 M solution of sodium bisulfite (3 x 1.00 mL), water (3 x 2.00 mL), anhydrous acetonitrile (3 × 2.00 mL) and then dried by flushing with nitrogen. Ammonia (20% in H_2O, 1.00 mL/mol oligonucleotide) was added to the solid-supported oligonucleotide (3 µmol) to cleave the support overnight at 40 °C, followed by filtration of the beads. The crude was desalted and purified by RP chromatography or used without purification for coupling reactions.

HPLC and Mass analysis

Entry	Name	Sequence (5' to 3')	Calcd	Found
**1**	ODN1-S_L	GCCAGC$\mathbf{S_L}$GACCG	3479.3	3479.2
**2**	ODN1-S_D	GCCAGC$\mathbf{S_D}$GACCG	3479.3	3479.3
**3**	ORN1-S_L	r(GCCAGC$\mathbf{S_L}$GACCG)	3655.2	3655.2
**4**	ORN1-S_D	r(GCCAGC$\mathbf{S_D}$GACCG)	3655.2	3655.4
**5**	cs-ODN1-C3	CGGTC**C3**GCTGGC	3486.2	3486.4
**6**	cs-ORN1-C3	r(CGGTC**C3**GCTGGC)	3635.2	3635.6
**7**	cs-ORN1-G	r(CGGUC**G**GCUGGC)	3842.3	3842.6
**8**	cs-ORN1-A	r(CGGUC**A**GCUGGC)	3825.3	3825.1
**9**	cs-ORN1-C	r(CGGUC**C**GCUGGC)	3801.3	3801.4
**10**	cs-ORN1-U	r(CGGUC**U**GCUGGC)	3805.3	3805.1
**11**	cs-ORN1-dS	r(CGGUC**dS**GCUGGC)	3676.2	3675.8
**12**	cs-ORN1-C2	r(CGGUC**C2**GCUGGC)	3620.2	3620.0
**13**	cs-ORN2-U	r(CUACU**U**AUCUAC)	3676.2	3676.5
**14**	cs-ORN3-U	r(CAGUG**U**UCAGAC)	3587.2	3587.4

ODN1-S_L

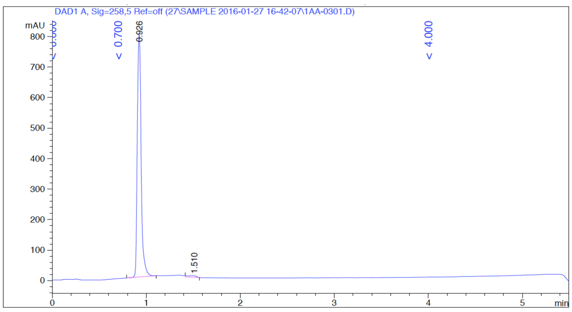

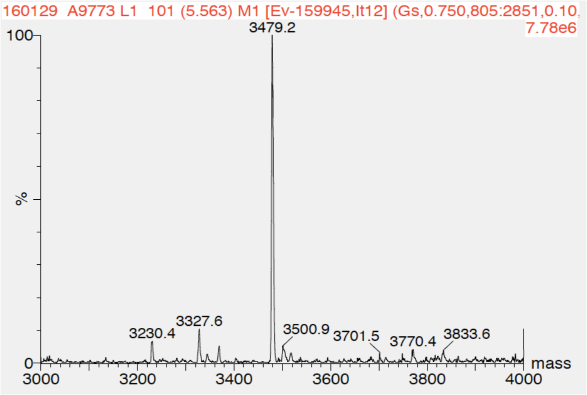

ODN1-S_D

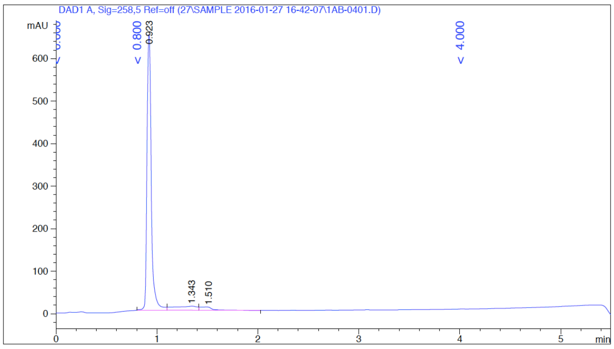

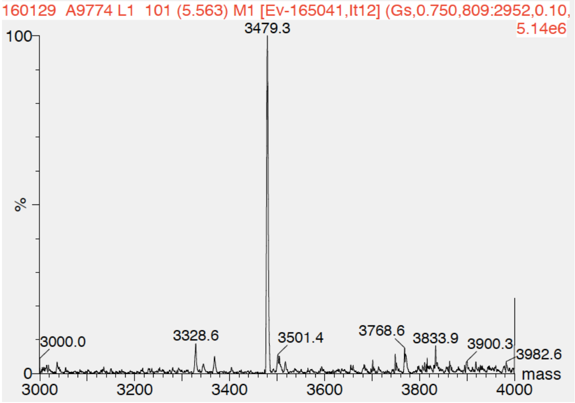

ORN1-S_L

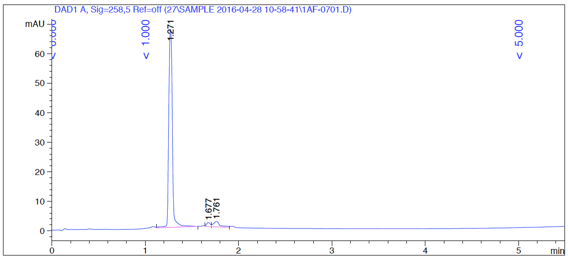

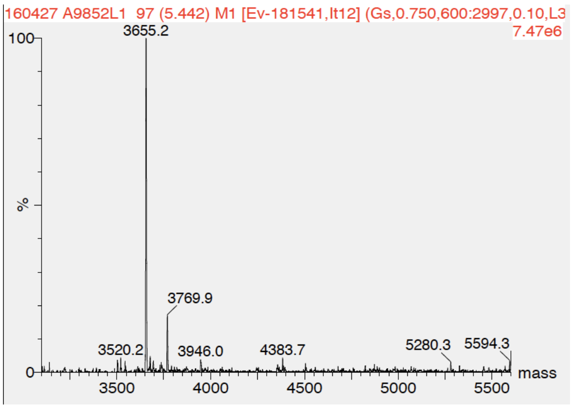

ORN1-S$_D$

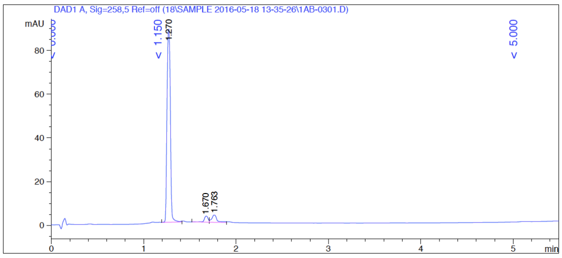

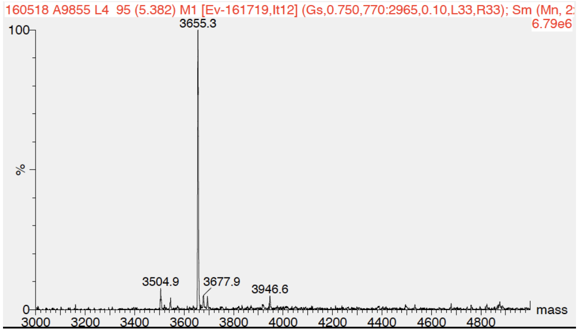

cs-ODN1-C3

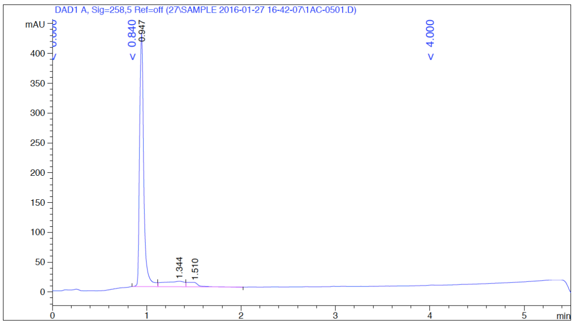

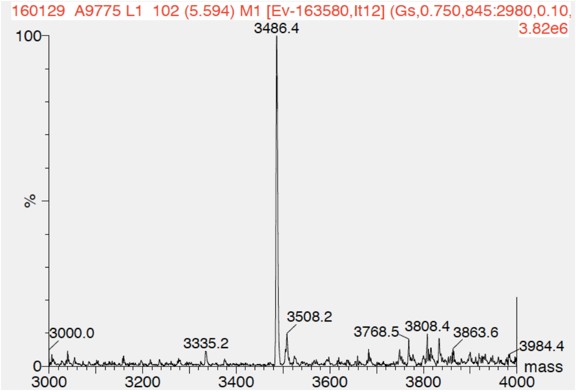

cs-ORN1-C3

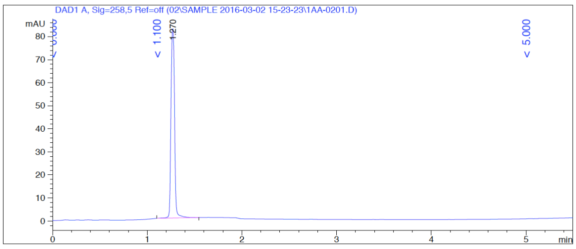

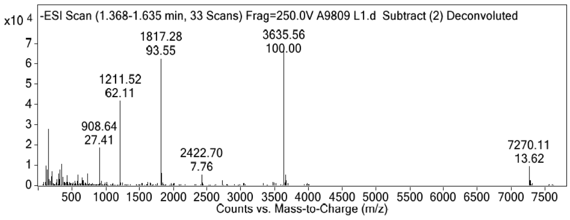

cs-ORN1-G

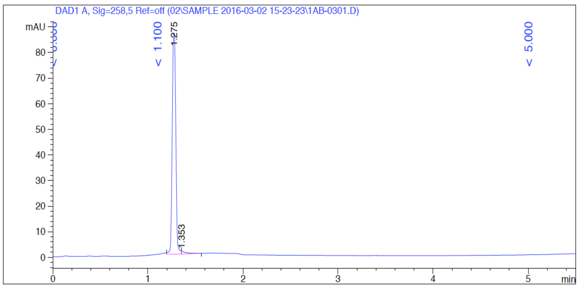

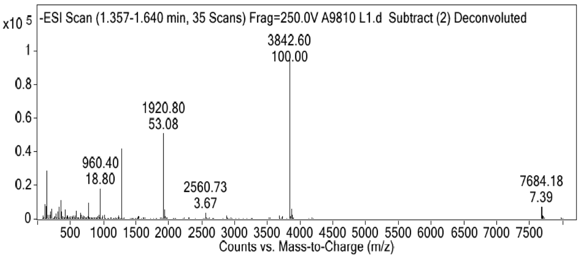

cs-ORN1-A

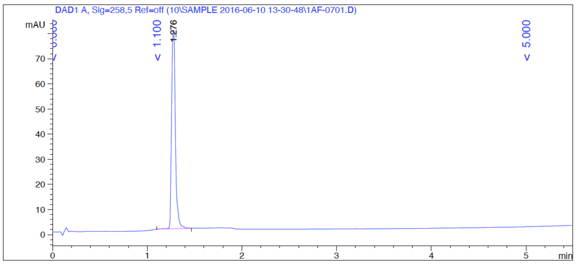

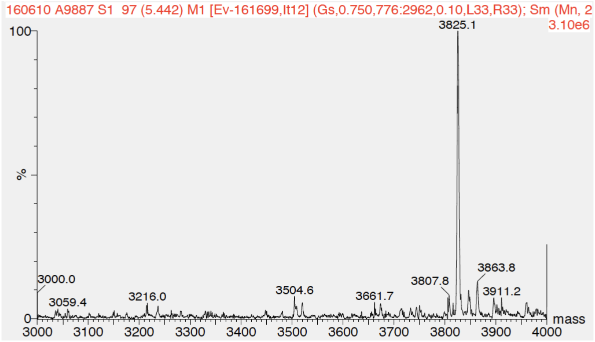

cs-ORN1-C

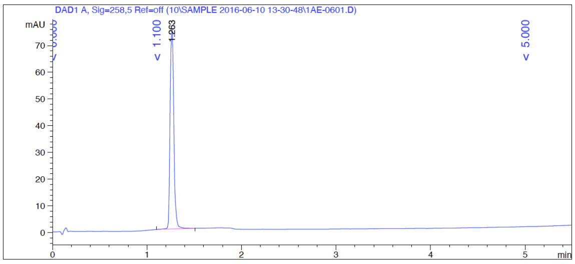

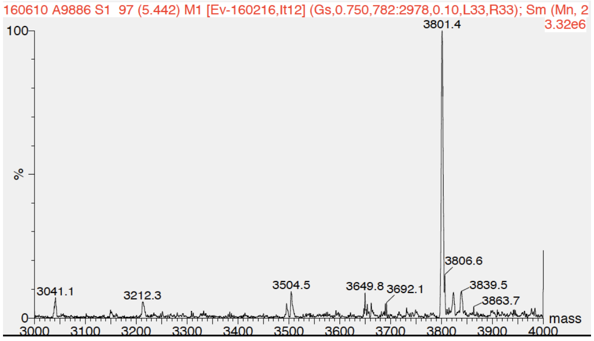

cs-ORN1-U

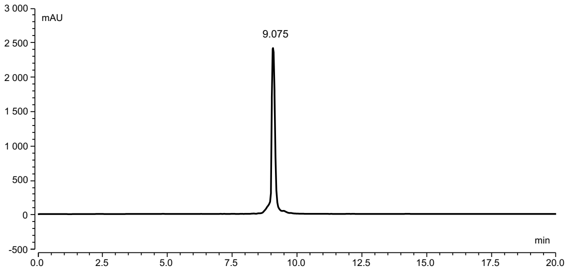

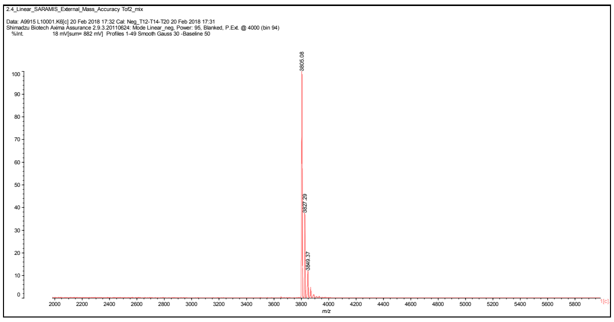

cs-ORN1-dS

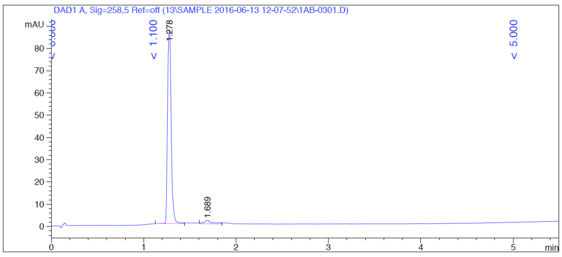

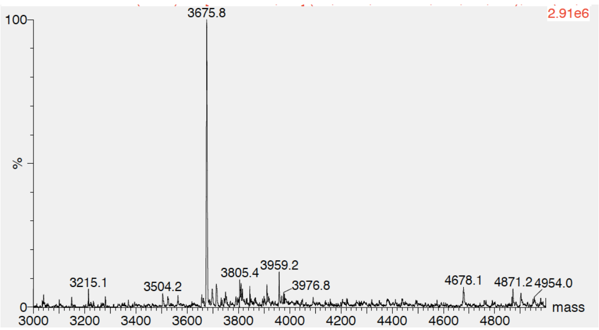

cs-ORN1-C2

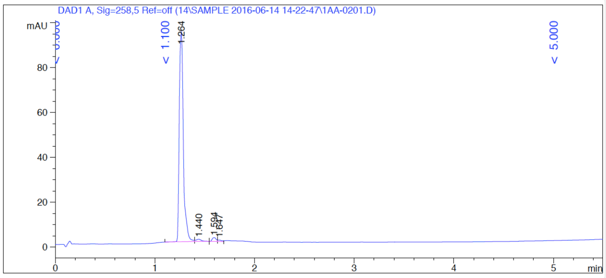

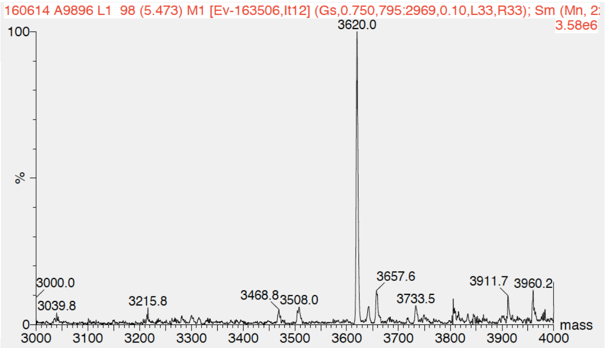

cs-ORN2-U

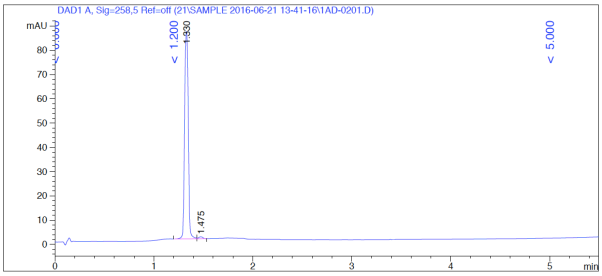

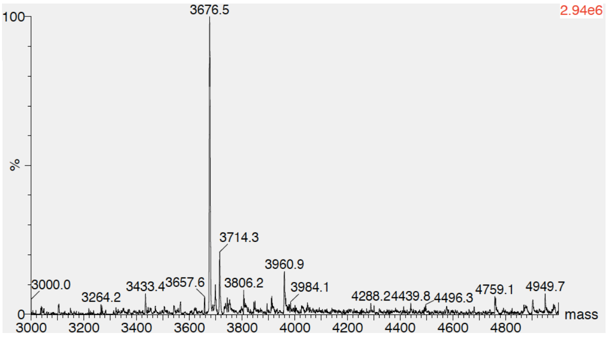

cs-ORN3-U

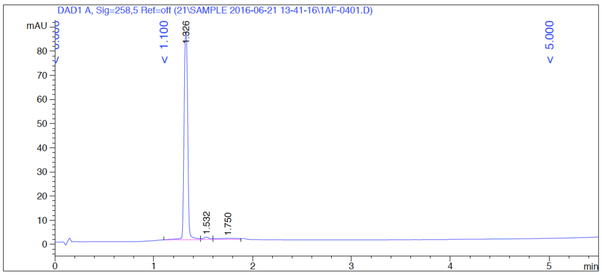

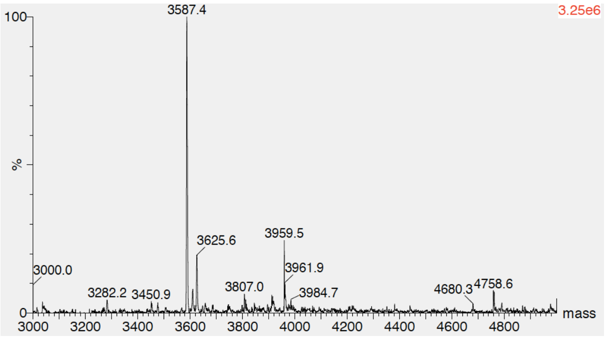

General procedure for PyBOP-mediated conjugation of R-COOH on serinol-modified

The amino-modified oligonucleotide (0.131 µmol, 1.00 equiv.) was dissolved in a H_2O/DMSO mixture (1:2, V_{tot} = 150 µL) to a typical 80 OD/mL and DIPEA (2.50% of V_{tot}, 3.75 µL) was added after cooling down to room temperature. In a separate reaction vessel, pre-activation of R-COOH was conducted by reacting the carboxylic acid component (0.524 µmol, 4.00 equiv. per amino function in the amino-modified precursor oligonucleotide) with PyBOP (10.0 µL from a stock solution C = 534 mM in DMSO, 0.524 µmol, 4.00 equiv.) in presence of DIPEA (11.0 µL from a stock solution C = 2.86 M in DMSO, 3.14 µmol, 24.0 equiv.). The resulting solution was stirred for 1 min and added to the amino-modified ON solution. When RP HPLC showed complete conversion, the reaction mixture was diluted with cold acetone (1.00 mL) and an aqueous solution of sodium acetate (C = 3.00 M, 20.0 µL) was added. After mixing the solution, an additional amount of cold acetone (1.00 mL) was added. The resulting white precipitate was spun down by centrifugation and separated from the supernatant. After centrifugation, the supernatant was removed and the remaining solvent was removed through speed vacuum. The crude was finally dissolved in H_2O and purified, desalted by RP chromatography.

HPLC and Mass analysis

Entry	Name	Sequence (5' to 3')	Calcd	Found
**1**	ODN1-S_L-BipyC$_2$	GCCAGC**S_L(BipyC$_2$)**GACCG	3703.5	3703.7
**2**	ODN2-S_L-BipyC$_2$	GTAGAT**S_L(BipyC$_2$)**AGTAG	3797.6	3796.9
**3**	ODN3-S_L-BipyC$_2$	GTATGA**S_L(BipyC$_2$)**CACTG	3733.6	3736.0
**4**	ODN1-S_D-BipyC$_2$	GCCAGC**S_D(BipyC$_2$)**GACCG	3703.5	3703.7
**5**	ORN1-S_L-BipyC$_2$	r(GCCAGC**S_L(BipyC$_2$)**GACCG)	3880.5	3880.7
**6**	ORN1-S_D-BipyC$_2$	r(GCCAGC**S_D(BipyC$_2$)**GACCG)	3880.5	3880.0
**7**	ODN1-S_L-BipyC$_0$	GCCAGC**S_L(BipyC$_0$)**GACCG	3661.4	3661.7
**8**	ODN1-S_L-BipyC$_3$	GCCAGC**S_L(BipyC$_3$)**GACCG	3717.5	3717.7
**9**	ODN1-S_D-BipyC$_0$	GCCAGC**S_D(BipyC$_0$)**GACCG	3661.4	3661.7
**10**	ODN1-S_D-BipyC$_3$	GCCAGC**S_D(BipyC$_3$)**GACCG	3717.5	3717.7

ODN1-S_L-BipyC$_2$

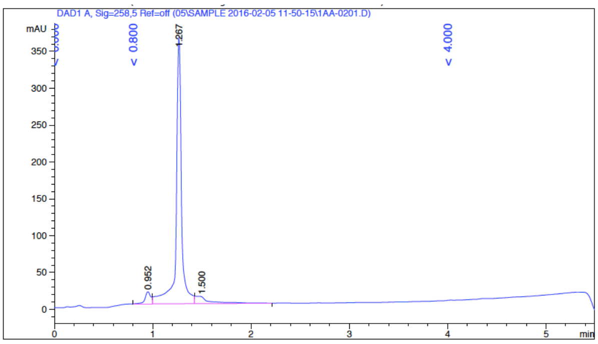

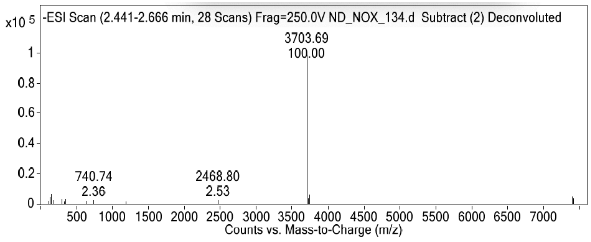

ODN2-S_L-BipyC$_2$

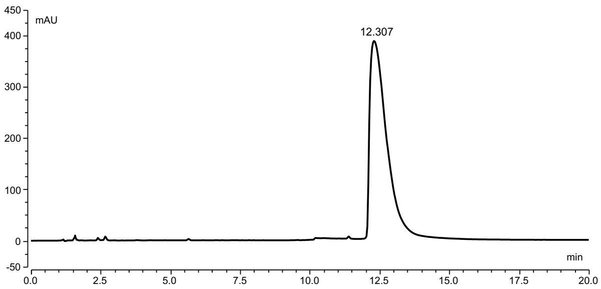

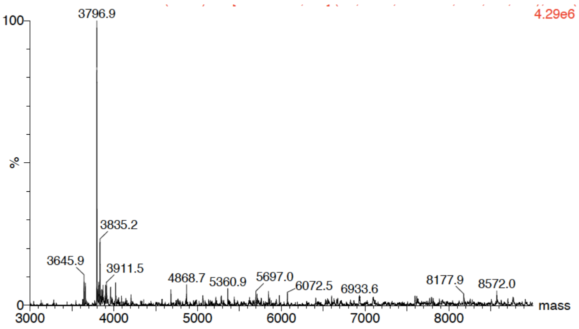

ODN3-S_L-BipyC$_2$

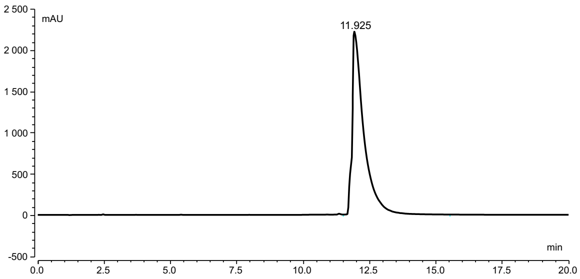

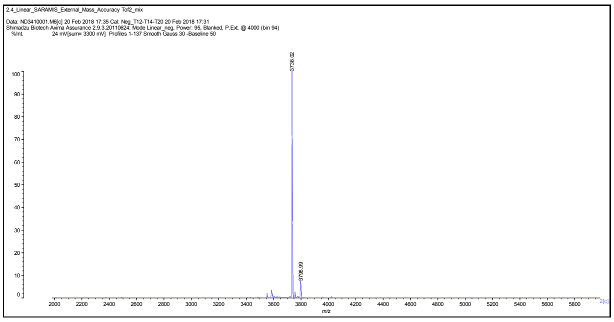

ODN1-S_D-BipyC$_2$

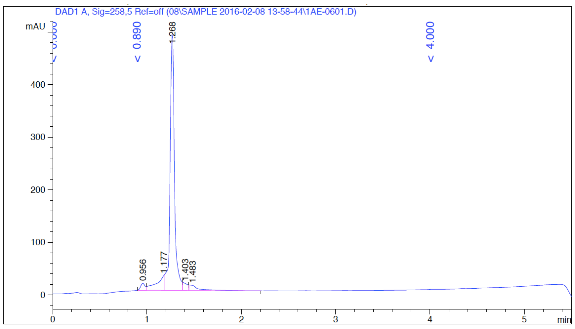

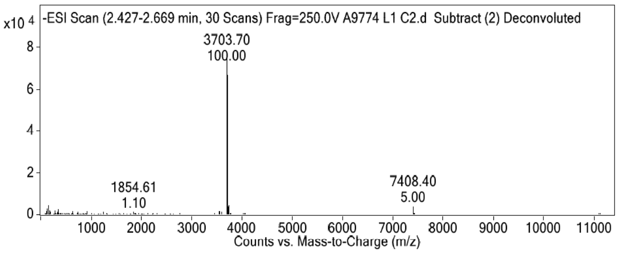

ORN1-S_L-BipyC_2

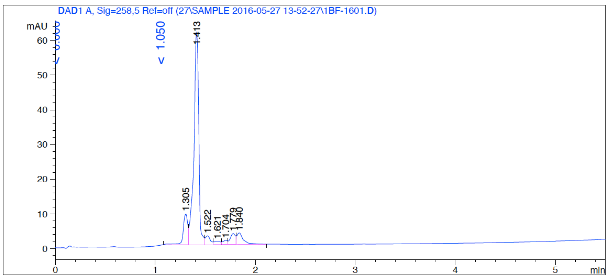

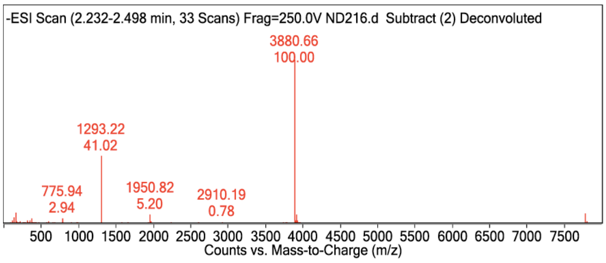

ORN1-S_D-BipyC_2

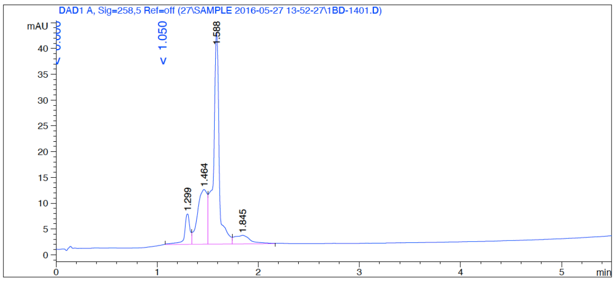

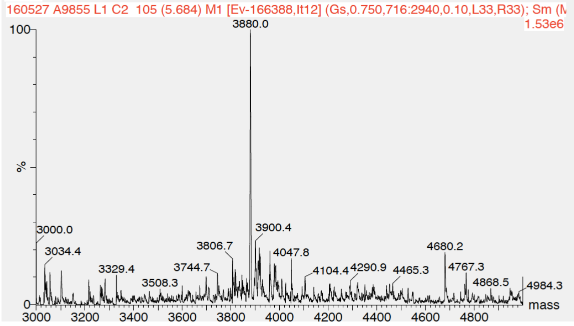

ODN1-S_L-BipyC_0

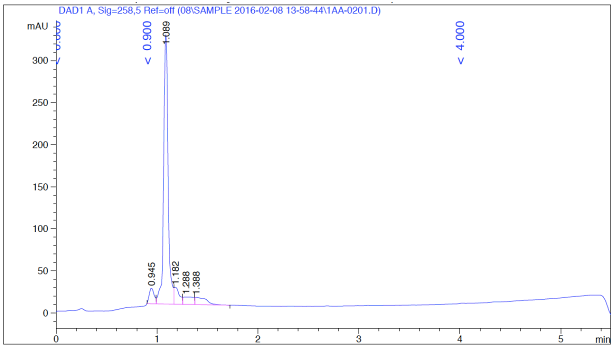

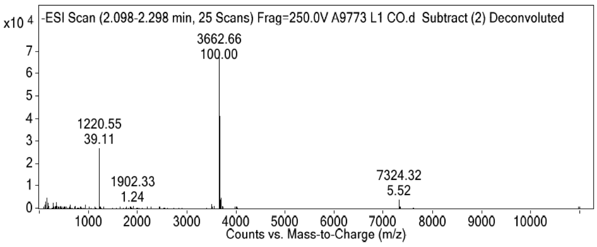

ODN1-S_L-BipyC_3

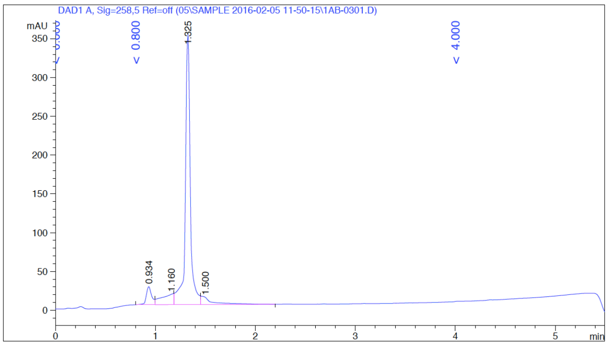

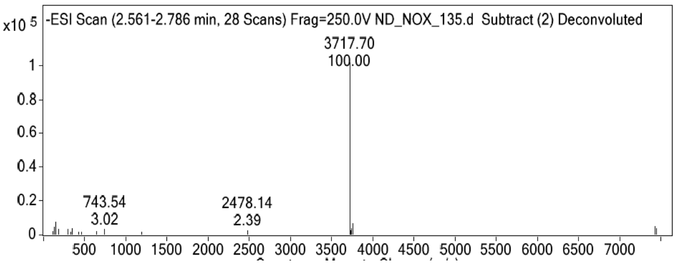

ODN1-S_D-BipyC$_0$

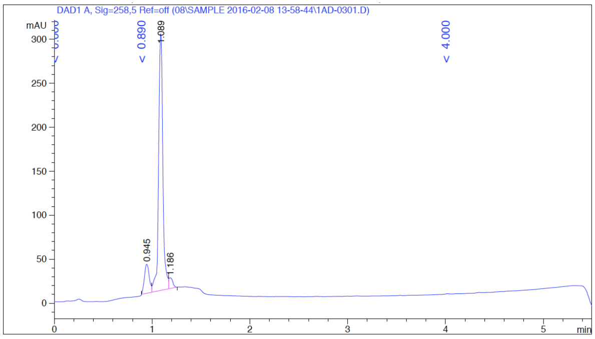

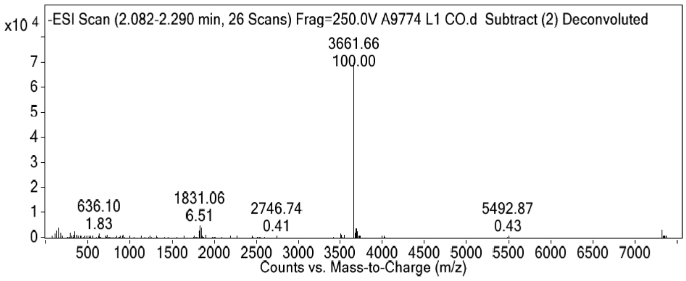

ODN1-S_D-BipyC$_3$

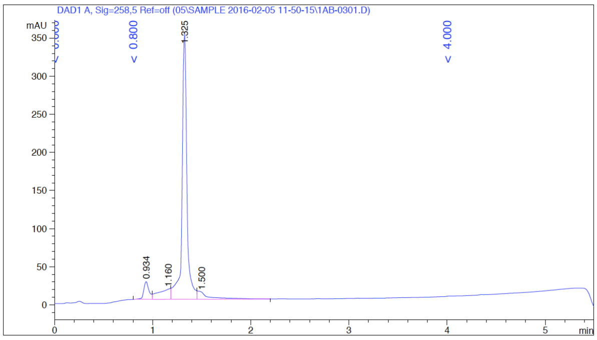

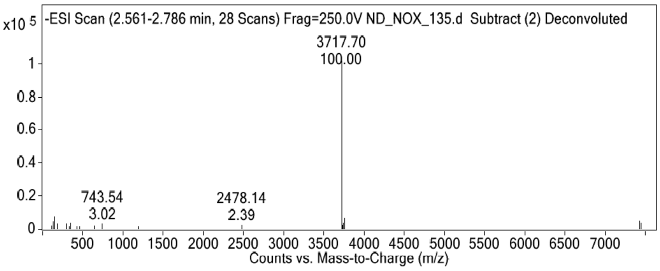

Melting temperatures

Duplex abbreviations	Sequence compositions
DNA/DNA-C3	5'-GCCAGC-**C**-GACCG-3'<br>5'-CGGTC-**C3 spacer**-GCTGGC-3'
DNA/RNA-C3	5'-GCCAGC-**C**-GACCG-3'<br>5'-CGGUC-**C3 spacer**-GCUGGC-3'
DNA-SerC2/DNA-C3	5'-GCCAGC-**(L)-Ser-bipyC2**-GACCG-3'<br>5'-CGGTC-**C3 spacer**-GCTGGC-3'
DNA-SerC2/RNA-C3	5'-GCCAGC-**(L)-Ser-bipyC2**-GACCG-3'<br>5'-CGGUC-**C3 spacer**-GCUGGC-3'
DNA-SerC2/RNA-U	5'-GCCAGC-**(L)-Ser-bipyC2**-GACCG-3'<br>5'-CGGUC-**U**-GCUGGC-3'

Table S1 – Melting temperatures of selected duplexes.

Entry	Duplex abbreviations	T_m (°C)[a]
1	DNA/DNA-C3	38.3
2	DNA/DNA-C3 **+ dmpby**	39.3
3	DNA/RNA-C3	45.1
4	DNA/RNA-C3 **+ dmpby**	44.4
5	**DNA-Ser^{C2}/DNA-C3**	53.2
6	**DNA-Ser^{C2}/RNA-C3**	53.0
7	**DNA-Ser^{C2}/RNA-U**	51.4

[a]Melting temperatures were obtained from the maxima of the first derivatives of the melting curve (A_{260} *vs.* temperature).

III. Preparation of stock solutions

Preparation of stock solutions of st-DNA.

st-DNA solutions were prepared by dissolving commercial st-DNA from Sigma Aldrich (reported to be about 2,000 base-pairs long) in Milli-Q H_2O to a concentration of 4.50 mg/mL (6.90 mM concentration relative to base pairs) at most two weeks before use.

Preparation of a 200 mM stock solution of MES buffer (pH 5.0).

3-(*N*-Morpholino)ethanesulfonic acid (2.13 g, 10.0 mmol, MW = 195.23 g/mol) was dissolved in Milli-Q H_2O (40.0 mL). The pH was adjusted to 5.0 using an aqueous solution of NaOH (C = 0.10 M, MW = 39.40 g/mol). To the resulting solution was added a given volume of MilliQ H_2O to obtain a 200 mM concentration.

Representative procedure for the preparation of other 200 mM stock solutions of buffer.

200 mM stock solutions of MOPS buffer were prepared by dissolving 3-(*N*-morpholino)propanesulfonic acid (1.05 g, MW = 209.26 g/mol) in Milli-Q H_2O (10.0 mL). The pH was adjusted as desired using an aqueous solution of KOH (C = 0.10 M, MW = 56.11 g/mol). To the resulting solution was added a given volume of MilliQ H_2O to obtain a 200 mM concentration.

Preparation of a 0.90 mM $Cu(NO_3)_2$.dmbpy stock solution.

$Cu(NO_3)_2 \cdot 3H_2O$ (4.80 mg, 0.02 mmol, 1.00 equiv.) and 4,4'-dimethyl-2,2'-bipyridyl (dmbpy, 4.40 mg, 0.024 mmol, 1.20 equiv.) were dissolved in 22.2 mL of Milli-Q H_2O. The mixture was stirred at room temperature for 5 h and used without purification.

Preparation of a 1.00 mM $Cu(NO_3)_2$ stock solution.

$Cu(NO_3)_2 \cdot 3H_2O$ (4.80 mg, 0.02 mmol, 1.00 equiv.) was dissolved in 20.0 mL of Milli-Q H_2O. The mixture was stirred at room temperature for 1 h and used the next day.

IV. DNA-catalysed reactions: Friedel-Crafts reactions, Michael additions, and sequential Friedel-Crafts alkylation/enantioselective protonation

A. Preparation of starting materials

General procedure I: Addition of 1-methylimidazole to carboxylic acids

The α,β-unsaturated substrates were synthesised *via* a modification of the procedure originally reported by Evans and co-workers.[8] An oven-dried round-bottom flask under an argon atmosphere was charged with *N*-methyl imidazole (2.40 equiv.) and dry THF (C = 0.50 M). The solution was cooled to −78 °C for 15 min, then a commercial solution of *n*-BuLi (C = 2.50 M in *n*-hexane, 2.40 equiv.) was added dropwise over 10 min. The mixture was warmed to room temperature and stirred for 30 min, then cooled back to −78 °C. The desired carboxylic acid (1.00 equiv.) in dry THF (C = 1.10 M) was added dropwise over a 10-min period. The resulting solution was stirred at −78 °C for 15 min, then warmed to room temperature and stirred for additional 2 h. After completion of the reaction (monitored by TLC), the reaction was quenched with a saturated aqueous solution of $NaHCO_3$ and the aqueous phase was extracted with ethyl acetate (three times). The combined organic layers were washed with a saturated solution of NaCl (two times), dried over anhydrous $MgSO_4$ and evaporated under reduced pressure. The crude mixture was eventually purified by flash column chromatography on silica gel to afford the desired product.

General procedure J: Aldol

The α,β-unsaturated substrates were synthesised *via* a modification of the procedure originally reported by Scheidt and co-workers.[9] An oven-dried round-bottom flask under an argon atmosphere was charged with 1-(1-methyl-1*H*-imidazol-2-yl)ethan-1-one (1.00 equiv.) and ethanol (C = 0.50 M). The desired aldehyde (1.00 equiv.) and a catalytic amount of KOH (0.20 equiv.) dissolved in a minimal amount of ethanol were added to the solution and the resulting reaction mixture was stirred at room temperature until completion of the reaction (monitored by TLC). The reaction was quenched with H_2O and the aqueous phase was extracted with ethyl acetate (three times). The combined organic layers were washed with a saturated aqueous solution of NaCl (two times), dried over anhydrous $MgSO_4$, gravity filtered and concentrated under reduced pressure. The crude product was eventually purified by flash column chromatography over silica gel to afford the desired product.

General procedure K: Addition of 2-(trimethysilyl)triazole on aldehyde

An oven-dried microwave vial under an argon atmosphere was charged with 2-(trimethysilyl)triazole (1.60 mL, 10.0 mmol, 1.00 equiv.) and the desired aldehyde (1.00 equiv.). The resulting solution was stirred at room temperature for 4 h. The mixture was then diluted in THF (200 mL) and a commercial solution of tetrabutylammonium fluoride solution (1.00 M in THF, 10.0 mL, 10.0 mmol, 1.00 equiv.) was added. After additional stirring for 1 h, the solvent was removed under vacuum, and then the crude was dissolved in ethyl acetate and washed with a saturated aqueous solution of $NaHCO_3$. The crude product was eventually purified by flash column chromatography on silica gel to afford the desired product.

General procedure L: MnO_2 oxidation of alcohol

An oven-dried 10 mL round-bottom flask under an argon atmosphere was charged with the desired alcohol (1.00 equiv.) and dichloromethane (C = 0.18 M) followed by MnO_2 (10.0 equiv.). The mixture was stirred at room temperature for 1.5 h. After completion of the reaction (monitored by TLC), the crude was filtered over Celite® and the solvent was removed under vacuum. The crude product was used on the same day without further purification.

Generation procedure M: Preparation of indoles

An oven-dried microwave vial under an argon atmosphere was charged with $Pd_2(dba)_3$ (0.01 equiv.), 2-diclyclohexylphosphino-2'-(*N,N*-dimethylamino)biphenyl (0.024 equiv.) and 5-bromoindole (196 mg, 1.00 mmol, 1.00 equiv.) in THF (1.00 mL). A commercial solution of LiHMDS (C = 1.00 M in THF, 2.20 equiv.) and the desired amine (1.20 equiv.) were slowly added to the solution, stirred at 65 °C overnight. After cooling down at room temperature, an aqueous solution of HCl (C = 1.00 M, 2.00 mL) was added and the mixture was stirred for additional 15 min. The crude was then quenched with a saturated aqueous solution of $NaHCO_3$, and extracted with ethyl acetate (three times). The combined organic phases were washed with a saturated aqueous solution of NaCl, dried over anhydrous $MgSO_4$, gravity filtered and concentrated under vacuum. The crude was eventually purified by flash column chromatography on silica gel to afford the desired product.

1-(1-Methyl-1*H*-imidazol-2-yl)ethan-1-one (xvii)

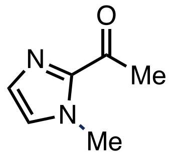

MW (g/mol): 124.06 **Molecular formula:** $C_6H_8N_2O$

An oven-dried 250 mL round-bottom flask under an argon atmosphere was charged with 1-methylimidazole (9.00 g, 8.80 mL, 110 mmol, 1.10 equiv.) and dry THF (120 mL). The solution was cooled to 0 °C in an ice bath for 15 min, then a commercial solution of *n*-BuLi (C = 2.50 M in *n*-hexane, 42.0 mL, 110 mmol, 1.10 equiv.) was added dropwise over 15 min. The mixture was stirred at 0 °C for additional 15 min, then cannulated into a solution of 4-acetylmorpholine (11.6 mL, 100 mmol, 1.00 equiv.) in dry THF (100 mL) at –78 °C. The reaction mixture was then stirred at –78 °C for 1 h and quenched with an aqueous solution of HCl (C = 1.00 M, 10.0 mL), stirred for 5 min and diluted with a saturated aqueous solution of $NaHCO_3$ (30.0 mL) and a saturated aqueous solution of NaCl (15.0 mL). The aqueous phase was extracted with ethyl acetate and the combined organic layers were dried over anhydrous $MgSO_4$, gravity filtered and concentrated under reduced pressure. The crude was eventually purified by flash column chromatography on silica gel (Ethyl acetate/Petroleum ether, 1:1) to provide the title compound as a colourless oil (9.68 g, 78.0 mmol, 78% yield). The spectroscopic data of the product were identical with those reported in the literature.[10]

^{1}H NMR (400 MHz, $CDCl_3$) δ 7.10 (s, 1H), 6.99 (s, 1H), 3.97 (s, 3H), 2.63 (s, 3H).

^{13}C NMR (101 MHz, $CDCl_3$) δ 190.2, 142.9, 128.7, 126.7, 35.9, 26.8.

(*E*)-1-(1-Methyl-1H-imidazol-2-yl)but-2-en-1-one (1a)

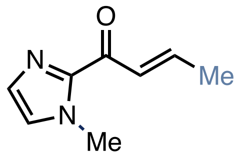

MW (g/mol): 150.18 **Molecular formula:** $C_8H_{10}N_2O$

Synthesised according general procedure I using crotonic acid (0.95 g, 11.4 mmol, 1.00 equiv.). The desired product was obtained as a colourless oil (0.91 g, 6.00 mmol, 53% yield) after purification by flash column chromatography on silica gel (Ethyl acetate/Pentane, 40:60). The spectroscopic data of the product were identical with those reported in the literature.[8]

^{1}H NMR (400 MHz, CDCl$_3$) δ 7.36 (dq, *J* = 15.5, 1.8 Hz, 1H), 7.13-7.01 (m, 2H), 6.99 (s, 1H), 3.98 (s, 3H), 2.20-1.58 (m, 3H).

^{13}C NMR (101 MHz, CDCl$_3$) δ 180.5, 143.7, 143.5, 129.0, 127.7, 126.9, 36.1, 18.3.

(*E*)-1-(1-Methyl-1*H*-imidazol-2-yl)hex-2-en-1-one (1b)

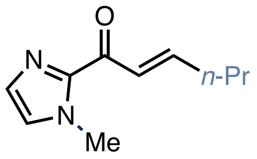

MW (g/mol): 178.24 **Molecular formula:** $C_{10}H_{14}N_2O$

Synthesised according general procedure I using (*E*)-2-hexenoic acid (2.60 g, 22.8 mmol, 1.00 equiv.). The desired product was obtained as a colourless oil (1.50 g, 8.43 mmol, 37% yield) after purification by flash column chromatography on silica gel (Diethyl ether/Petroleum ether, 40:60). The spectroscopic data of the product were identical with those reported in the literature.[8]

^{1}H NMR (400 MHz, CDCl$_3$) δ 7.38 (dt, *J* = 15.7, 1.4 Hz, 1H), 7.19-7.05 (m, 2H), 7.01 (s, 1H), 4.02 (s, 3H), 2.35-2.19 (m, 2H), 1.61-1.45 (m, 2H), 0.94 (t, *J* = 7.4 Hz, 3H).

^{13}C NMR (101 MHz, CDCl$_3$) δ 180.7, 148.6, 143.8, 129.1, 127.0, 126.4, 36.2, 34.7, 21.5, 13.7.

(*E*)-3-(4-Methoxyphenyl)-1-(1-methyl-1*H*-imidazol-2-yl)prop-2-en-1-one (1c)

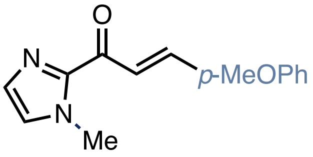

MW (g/mol): 242.28 **Molecular formula:** $C_{14}H_{14}N_2O_2$

Synthesised according general procedure J using 1-(1-methyl-1*H*-imidazol-2-yl)ethan-1-one (1.30 g, 10.5 mmol, 1.00 equiv.). The desired product was obtained as a yellow solid (1.30 g, 5.36 mmol, 51% yield) after purification by flash column chromatography over silica gel (Diethyl ether/Pentane, 90:10). The spectroscopic data of the product were identical with those reported in the literature.[9]

^{1}H NMR (400 MHz, CDCl$_3$) δ 7.93 (d, *J* = 16.0 Hz, 1H), 7.77 (d, *J* = 16.0 Hz, 1H), 7.62 (d, *J* = 8.8 Hz, 2H), 7.18 (s, 1H), 7.03 (s, 1H), 6.89 (d, *J* = 8.8 Hz, 2H), 4.05 (s, 3H), 3.81 (s, 3H).

^{13}C NMR (101 MHz, CDCl$_3$) δ 180.4, 161.5, 144.0, 143.1, 130.4 (2C), 129.0, 127.6, 127.0 , 120.4, 114.2 (2C), 55.3, 36.3.

(*E*)-1-(1-Methyl-1*H*-imidazol-2-yl)-3-phenylprop-2-en-1-one (1d)

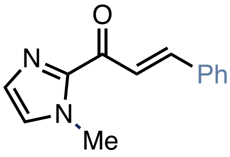

MW (g/mol): 212.25 **Molecular formula:** $C_{13}H_{12}N_2O$

To a solution of 1-(1-methyl-1H-imidazol-2-yl)ethan-1-one **xiii** (667 mg, 5.37 mmol, 1.00 equiv.) in ethanol (10 mL) were added the benzaldehyde (545 µL, 5.37 mmol, 1.00 equiv.) followed by lithium hydroxide (141 mg, 5.91 mmol, 1.10 equiv.). The resulting reaction mixture was stirred overnight. Upon completion, the reaction was quenched by addition of water (10.0 mL). The resulting aqueous mixture was extracted with ethyl acetate (3 x 20.0 mL) and combined organic layers were dried over anhydrous $MgSO_4$ and concentrated under reduced pressure. The crude was purified by flash column

chromatography over silica gel (Petroleum ether/Diethyl ether = 2:8). The desired product was obtained as a yellow solid (856 mg, 4.03 mmol, 75% yield). The spectroscopic data of the product were identical with those reported in the literature.[10]

^{1}H NMR (400 MHz, $CDCl_3$) δ 8.08 (d, *J* = 16.0 Hz, 1H), 7.83 (d, *J* = 16.0 Hz, 1H), 7.73-7.67 (m, 2H), 7.44-7.36 (m, 3H), 7.22 (d, *J* = 0.9 Hz, 1H), 7.08 (d, *J* = 0.9 Hz, 1H), 4.10 (s, 3H).

^{13}C NMR (101 MHz, $CDCl_3$) δ 180.7, 144.2, 143.5, 135.1, 130.6, 129.5, 129.0 (2C), 128.9 (2C), 127.4, 123.0, 36.5.

2-Methyl-1-(thiazol-2-yl)prop-2-en-1-ol (xviii)

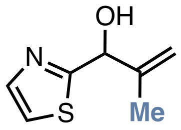

MW (g/mol): 155.22 **Molecular formula:** C_7H_9NOS

Synthesised according general procedure K using methacrolein (0.82 mL, 10.0 mmol, 1.00 equiv.). The desired product was obtained as yellow oil (776 mg, 5.00 mmol, 50% yield) after purification by flash column chromatography over silica gel (Petroleum ether/Ethyl acetate, from 100:0 to 80:20). The spectroscopic data of the product were identical with those reported in the literature.[11]

^{1}H NMR (400 MHz, $CDCl_3$) δ 7.75 (d, *J* = 3.3 Hz, 1H), 7.34 (d, *J* = 3.3 Hz, 1H), 5.47 (s, 1H), 5.26 (m, 1H), 5.07 (m, 1H), 3.25 (br s, 1H, -*OH*), 1.73 (s, 3H).

^{13}C NMR (101 MHz, $CDCl_3$) δ 173.0, 145.2, 142.0, 119.9, 114.3, 75.9, 17.4.

2-Methylene-1-(thiazol-2-yl)butan-1-ol (xix)

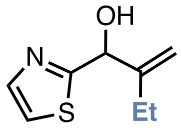

MW (g/mol): 169.24 **Molecular formula:** $C_8H_{11}NOS$

Synthesised according general procedure K using 2-ethylacrolein (0.98 mL, 10.0 mmol, 1.00 equiv.). The desired product was obtained as yellow oil (542 mg, 3.20 mmol, 32% yield) after purification by flash column chromatography over silica gel (Petroleum ether/Ethyl acetate, from 100:0 to 75:25).

^{1}H NMR (400 MHz, CDCl$_3$) δ 7.73 (d, *J* = 3.2 Hz, 1H), 7.32 (d, *J* = 3.2 Hz, 1H), 5.49 (d, *J* = 3.1 Hz, 1H), 5.30 (m, 1H), 5.07 (m, 1H), 3.22 (s, 1H, *-OH*), 2.17 (m, 1H), 1.97 (m, 1H), 1.04 (t, *J* = 7.4 Hz, 3H).

^{13}C NMR (101 MHz, CDCl$_3$) δ 173.2, 151.2, 142.3, 119.8, 111.8, 75.8, 23.7, 12.1.

HRMS (ESI): m/z calcd for $C_8H_{12}NOS$, $[M+H]^+$: 170.0634, found: 170.0637.

IR (neat): 3237, 2967, 1505, 1136, 1043, 907, 724 cm^{-1}.

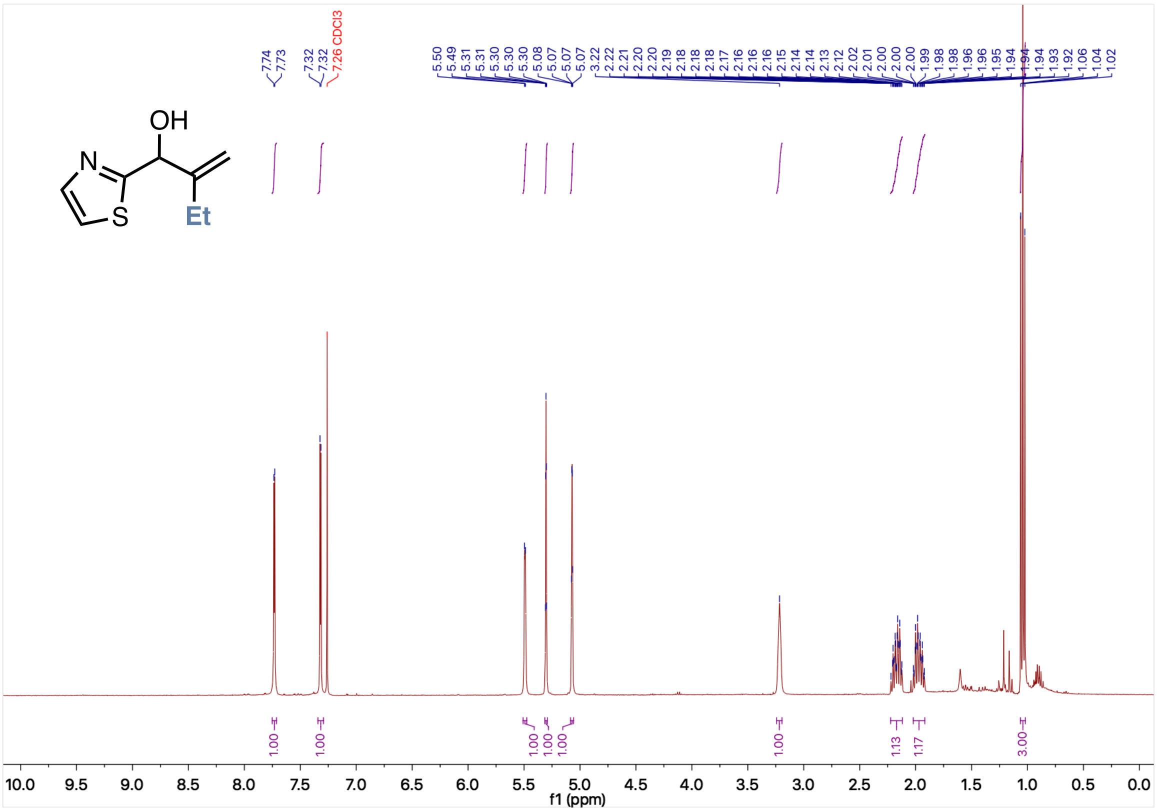

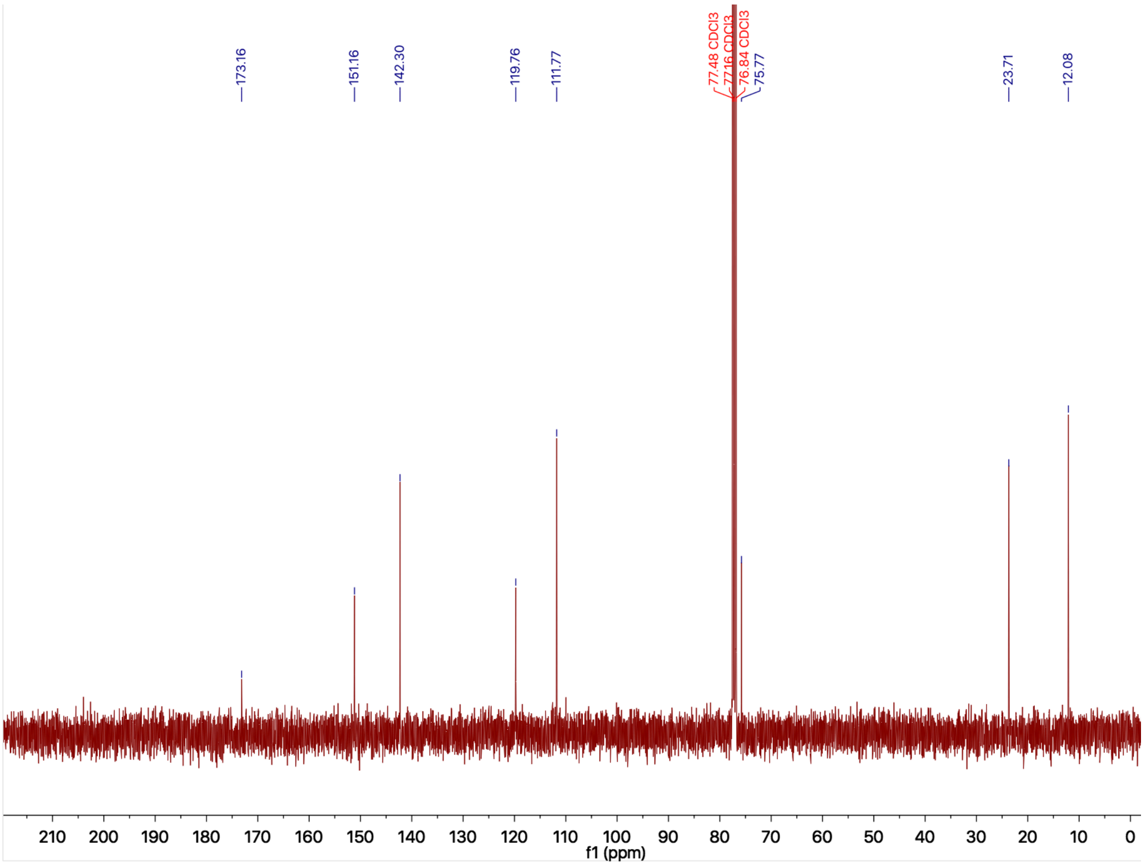

2-Methyl-1-(thiazol-2-yl)prop-2-en-1-one (1e)

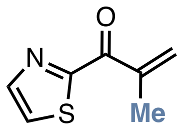

MW (g/mol): 153.20 **Molecular formula:** C_7H_7NOS

Synthesised according general procedure L using alcohol **xviii** (100 mg, 0.64 mmol, 1.00 equiv.). The desired product was obtained as a colourless oil (85.0 mg, 0.56 mmol, 87% yield) and was used on the same day without further purification. The spectroscopic data of the product were identical with those reported in the literature.[11]

^{1}H NMR (400 MHz, CDCl₃) δ 7.99 (d, *J* = 3.1 Hz, 1H), 7.64 (d, *J* = 3.1 Hz, 1H), 6.93 (m, 1H), 6.14 (m, 1H), 2.09 (s, 3H).

^{13}C NMR (101 MHz, CDCl₃) δ 185.6, 167.7, 144.6, 141.8, 131.4, 125.8, 18.9.

2-Methylene-1-(thiazol-2-yl)butan-1-one (1f)

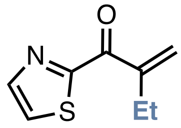

MW (g/mol): 167.23 **Molecular formula:** C_8H_9NOS

Synthesised according general procedure L using alcohol **xix** (60.0 mg, 0.35 mmol, 1.00 equiv.). The desired product was obtained as a colourless oil (53.0 mg, 0.32 mmol, 90% yield) and was used on the same day without further purification.

^{1}H NMR (400 MHz, $CDCl_3$) δ 8.00 (d, *J* = 3.1 Hz, 1H), 7.64 (d, *J* = 3.1 Hz, 1H), 6.86 (m, 1H), 6.09 (m, 1H), 2.54-2.48 (m, 2H), 1.14 (t, *J* = 7.4 Hz, 3H).

^{13}C NMR (101 MHz, $CDCl_3$) δ 185.9, 168.0, 147.6, 144.6, 129.1, 125.8, 25.1, 18.9.

HRMS (ESI): m/z calcd for $C_8H_{10}NOS$, $[M+H]^+$: 168.0478, found: 168.0471.

IR (neat): 1767, 1323, 1142, 901 cm^{-1}.

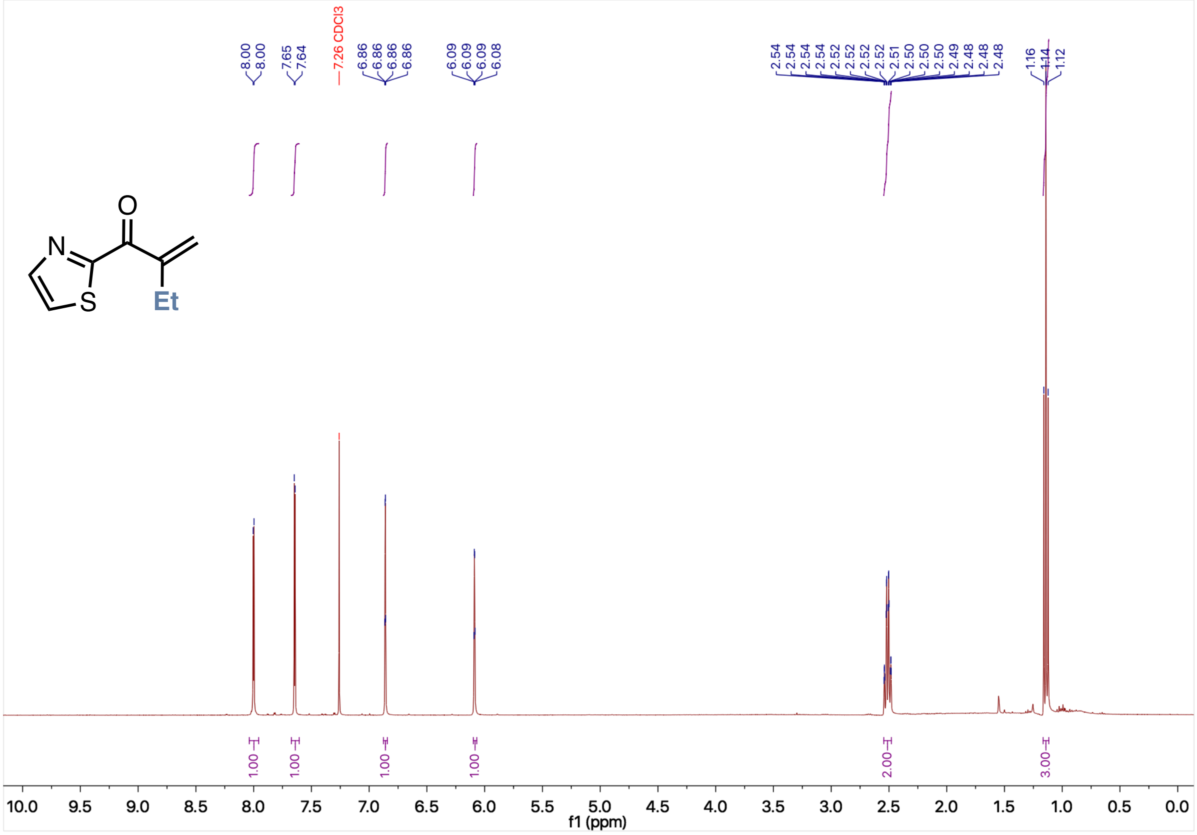
8.00
8.00
7.65
7.64
7.26 CDCl3
6.86
6.86
6.86
6.86
6.09
6.09
6.09
6.08
2.54
2.54
2.54
2.54
2.52
2.52
2.52
2.52
2.51
2.50
2.50
2.50
2.49
2.48
2.48
2.48
1.16
1.14
1.12
O
N
S
Et
1.00
1.00
1.00
1.00
2.00
3.00
10.0 9.5 9.0 8.5 8.0 7.5 7.0 6.5 6.0 5.5 5.0 4.5 4.0 3.5 3.0 2.5 2.0 1.5 1.0 0.5 0.0
f1 (ppm)

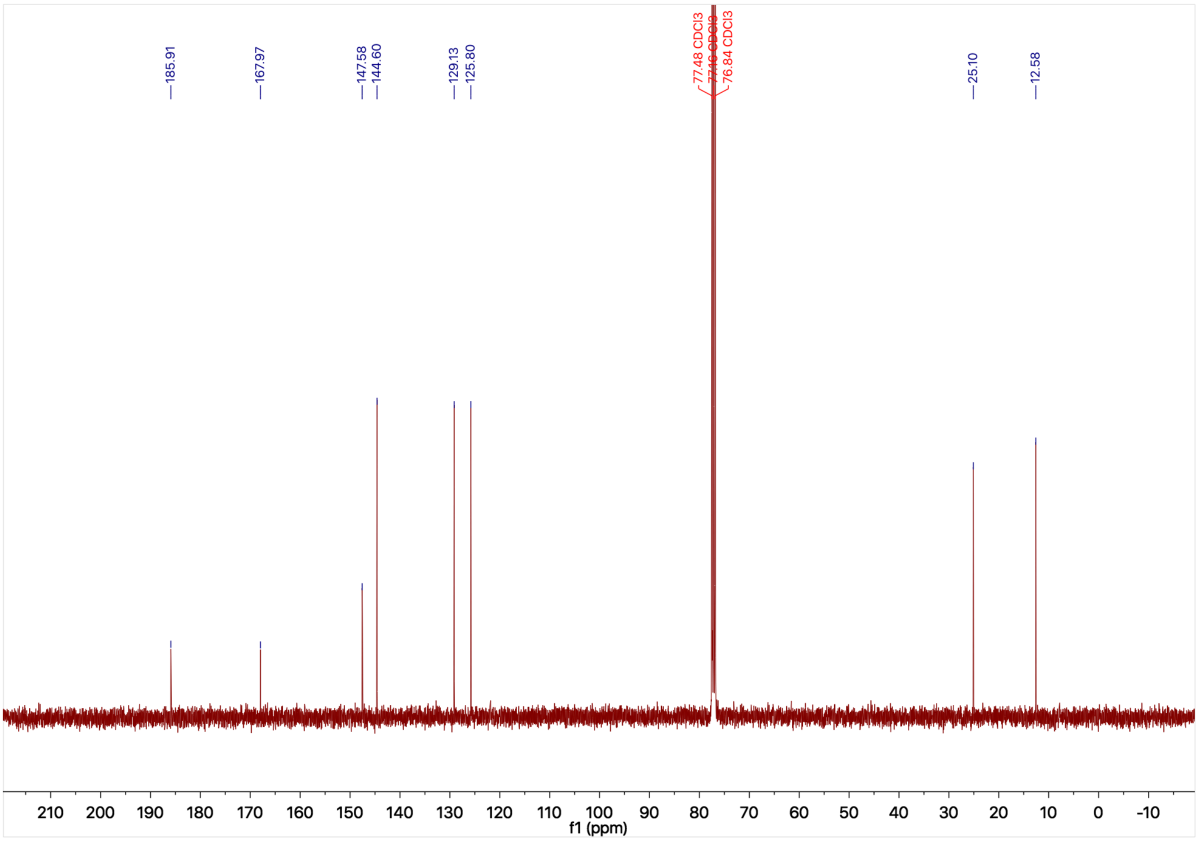
185.91
167.97
147.58
144.60
129.13
125.80
77.48 CDCl3
77.16 CDCl3
76.84 CDCl3
25.10
12.58
210 200 190 180 170 160 150 140 130 120 110 100 90 80 70 60 50 40 30 20 10 0 -10
f1 (ppm)

4-(1*H*-Indol-5-yl)morpholine (xx)

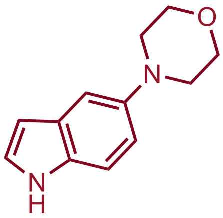

MW (g/mol): 202.26 **Molecular formula:** $C_{12}H_{14}N_2O$

Synthesised according general procedure M using morpholine (105 µL, 1.20 mmol, 1.20 equiv.). The desired product was obtained as a brown oil (142 mg, 0.70 mmol, 70% yield) after purification by flash column chromatography over silica gel (Petroleum ether/Ethyl acetate, from 100:0 to 125:75). The spectroscopic data of the product were identical with those reported in the literature.[11]

^{1}H NMR (400 MHz, $CDCl_3$) δ 8.15 (br s, 1H, -*NH*), 7.30 (d, *J* = 8.8 Hz, 1H), 7.19 (d, *J* = 2.4 Hz, 1H), 7.16 (t, *J* = 2.5 Hz, 1H), 6.97 (dd, *J* = 8.8, 2.5 Hz, 1H), 6.49 (m, 1H), 3.96-3.89 (m, 4H), 3.19-3.12 (m, 4H).

^{13}C NMR (101 MHz, $CDCl_3$) δ 146.0, 131.6, 128.5, 124.9, 115.4, 111.7, 107.6, 102.5, 67.4, 52.2.

5-(Piperidin-1-yl)-1*H*-indole (xxi)

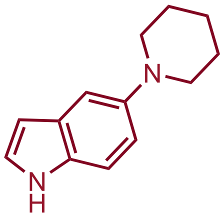

MW (g/mol): 200.29 **Molecular formula:** $C_{13}H_{16}N_2$

Synthesised according general procedure M using piperidine (119 µL, 1.20 mmol, 1.20 equiv.). The desired product was obtained as a brown oil (150 mg, 0.75 mmol, 75% yield) after purification by flash column chromatography over silica gel (Petroleum ether/Ethyl acetate, from 100:0 to 125:75). The spectroscopic data of the product were identical with those reported in the literature.[11]

^{1}H NMR (400 MHz, $CDCl_3$) δ 8.22 (br s, 1H, -*NH*), 7.27-7.20 (m, 2H), 7.12 (t, *J* = 2.8 Hz, 1H), 7.02 (dd, *J* = 8.8, 2.3 Hz, 1H), 6.47 (t, *J* = 2.5 Hz, 1H), 3.23-3.03 (m, 4H), 1.81 (q, *J* = 5.7 Hz, 4H), 1.59 (m, 2H).

^{13}C NMR (101 MHz, $CDCl_3$) δ 147.2, 131.5, 128.4, 124.6, 116.7, 111.5, 108.3, 102.4, 53.7, 26.5, 24.4.

B. Preparation of racemic products

Racemic Friedel-Crafts alkylations with indole

An oven-dried 25 mL round-bottom flask was charged with $Cu(NO_3)_2{\cdot}3H_2O$ (0.10 equiv.) and 4,4’-dimethyl-2,2’-bipyridyl (dmbpy, 0.12 equiv.) and acetonitrile (5.00 mL). The mixture was stirred at room temperature for 10 min then the α,β-unsaturated substrate (0.35 mmol, 1.00 equiv.) and indole (1.50 equiv.) were added. The solution was stirred at room temperature until completion of the reaction (monitored by TLC). The reaction was diluted with H_2O (10.0 mL) and extracted with diethyl ether (three times). The combined organic layers were dried over anhydrous $MgSO_4$, gravity filtered and concentrated under reduced pressure. The crude product was eventually purified by flash column chromatography over silica gel.

Racemic Michael additions of dimethyl malonate

An oven-dried 10 mL round-bottom flask was charged with $Cu(NO_3)_2{\cdot}3H_2O$ (0.20 equiv.), 4,4’-dimethyl-2,2’-bipyridyl (dmbpy, 0.22 equiv.) and acetonitrile (1.00 mL) or DMF (1.00 mL). The mixture was stirred at room temperature for 10 min then the α,β-unsaturated substrate (0.33 mmol, 1.00 equiv.) and dimethyl malonate (20.0 equiv.) were added. The solution was stirred at room temperature until completion of the reaction (monitored by TLC). The reaction was diluted with H_2O (10.0 mL) and extracted with diethyl ether (three times). The combined organic layers dried over anhydrous $MgSO_4$, gravity filtered and concentrated under reduced pressure. The crude product was eventually purified by flash column chromatography over silica gel.

Racemic Michael additions of nitromethane/malononitrile/*tert*-butyl cyanoacetate

An oven-dried 10 mL round-bottom flask was charged K_2CO_3 (5.00 equiv.), nitromethane/malononitrile/*tert*-butyl cyanoacetate (5.00 equiv.) and DMF (3.00 mL). The mixture was stirred at room temperature for 10 min then the α,β-unsaturated substrate (0.33 mmol, 1.00 equiv.) was added. The solution was stirred at room temperature until completion of the reaction (monitored by TLC). The reaction was diluted with H_2O (10.0 mL) and extracted with diethyl ether (three times). The combined organic layers dried over anhydrous $MgSO_4$, gravity filtered and concentrated under reduced pressure. The crude product was eventually purified by flash column chromatography over silica gel.

Racemic sequential Friedel-Crafts alkylations/protonation

An oven-dried microwave vial under an argon atmosphere was charged with $Cu(NO_3)_2 \cdot 3H_2O$ (0.10 equiv.) and 4,4'-dimethyl-2,2'-bipyridyl (dmbpy, 0.12 equiv.) in acetonitrile (2.00 mL). The mixture was stirred for 10 min and a solution of enone **1e** or **1f** (0.26 mmol, 1.00 equiv.) and the desired indole (1.50 equiv.) in acetonitrile (2.00 mL) was added. The solution was stirred at room temperature for 3 d. Solvent was removed under vacuum and the crude was purified by flash column chromatography on silica gel (*n*-Hexane/Ethyl acetate) to give the desired compound. In all cases, yields were low (not reported) and purifications were not trivial.

C. DNA-catalysed reactions with serinol-modified oligonucleotides and st-DNA

Duplex abbreviations	Sequence compositions
DNA-SerC2/RNA-U	5'-GCCAGC-**(L)-Ser**-bipyC2-GACCG-3'<br>5'-CGGUC-**U**-GCUGGC-3'
DNA-SerC2/RNA-C3	5'-GCCAGC-**(L)-Ser**-bipyC2-GACCG-3'<br>5'-CGGUC-**C3**-GCUGGC-3'

General procedure N: Enantioselective Friedel-Crafts reactions and Michael additions (nitromethane, malononitrile and *tert*-butyl cyanoacetate) - covalent approach with serinol-modified oligonucleotides DNA-SerC2/RNA-U

Strand solution **DNA**-SerC2 (31.0 µL, stock solution 536 nmol/mL in Milli-Q H_2O, 0.165 equiv.), counter strand solution **RNA-U** (41.4 µL, stock solution 481 nmol/mL in Milli-Q H_2O, 0.198 equiv.) and Milli-Q H_2O (4.60 µL) were successively added to a 500 µL Eppendorf® safe-lock tube. The reaction mixture was briefly mixed, heated at 90 °C for 5 min and allowed to cool down at room temperature. MOPS buffer solution (10.0 µL, stock solution 200 mM in Milli-Q H_2O, pH 6.5, 1/10 V_{tot}) and $Cu(NO_3)_2$ solution (13.0 µL, stock solution 1.00 mM in Milli-Q H_2O, 0.13 equiv.) were then added. The resulting mixture was stirred at 19 °C for 20 min and frozen at –20 °C for 30 min. The Eppendorf® safe-lock tube was brought into the cold room and the desired enone (2.00 µL, stock solution 0.05 M in DMSO, 0.10 µmol, 1.00 equiv.) was added and indole (2.00 µL, 0.25 M in DMSO, 0.50 µmol, 5.00 equiv.) **OR** nitromethane (5.40 µL, 100 µmol, 1000 equiv.) **OR** malononitrile (10.0 µL, 1.00 M in DMSO, 10.0 µmol, 100 equiv.) **OR** *tert*-butyl cyanoacetate (1.40 µL, 10.0 µmol, 100 equiv.) was finally added. The resulting solution was stirred at 4 °C for 3 d in a thermoshaker placed in a cold room. The mixture was then transferred into 1.5 mL Eppendorf® safe-lock tube. The initial Eppendorf® safe-lock tube was rinsed with H_2O (100 µL) and diethyl ether (100 µL). Additional diethyl ether (400 µL) was finally added. After stirring, the tube was placed in dry ice to freeze H_2O. The etheral phase was poured into a new 1.5 mL Eppendorf® safe-lock tube. The process was repeated twice (2 x 500 µL diethyl ether). After evaporation of diethyl ether, the sample was dissolved in *i*-PrOH (2 x 50 µL), transferred in HPLC mini vials and injected in HPLC to measure conversion and ee.

General procedure O: Enantioselective Michael additions of dimethyl malonate - covalent approach with serinol-modified oligonucleotides DNA-SerC2/RNA-U

Strand solution **DNA-SerC2** (31.0 µL, stock solution 536 nmol/mL in Milli-Q H_2O, 0.165 equiv.), counter strand solution **RNA-U** (41.4 µL, stock solution 481 nmol/mL in Milli-Q H_2O, 0.198 equiv.) and Milli-Q H_2O (4.60 µL) were successively added to a 500 µL Eppendorf® safe-lock tube. The reaction mixture was briefly mixed, heated at 90 °C for 5 min and allowed to cool down at room temperature. MOPS buffer solution (10.0 µL, stock solution 200 mM in Milli-Q H_2O, pH 7.9, 1/10 V_{tot}) and $Cu(NO_3)_2$ solution (13.0 µL, stock solution 1.00 mM in Milli-Q H_2O, 0.13 equiv.) were then added. The resulting mixture was stirred at 19 °C for 20 min and frozen at –20 °C for 30 min. The Eppendorf® safe-lock tube was brought into the cold room and the desired enone (2.00 µL, stock solution 0.05 M in DMSO, 0.10 µmol, 1.00 equiv.) and dimethyl malonate (1.20 µL, 10.0 µmol, 100 equiv.) were finally added. The resulting solution was stirred at 4 °C for 3 d in a thermoshaker placed in a cold room. The same work-up used in general procedure N was followed.

General procedure P: Sequential Friedel-Crafts alkylation/enantioselective protonation - covalent approach with serinol-modified oligonucleotides DNA-SerC2/RNA-U

Strand solution **DNA-SerC2** (31.0 µL, stock solution 536 nmol/mL in Milli-Q H_2O, 0.165 equiv.), counter strand solution **RNA-U** (41.4 µL, stock solution 481 nmol/mL in Milli-Q H_2O, 0.198 equiv.) and Milli-Q H_2O (4.60 µL) were successively added to a 500 µL Eppendorf® safe-lock tube. The reaction mixture was briefly mixed, heated at 90 °C for 5 min and allowed to cool down at room temperature. MES buffer solution (10.0 µL, stock solution 200 mM in Milli-Q H_2O, pH 5.0, 1/10 V_{tot}) and $Cu(NO_3)_2$ solution (13.0 µL, stock solution 1.00 mM in Milli-Q H_2O, 0.13 equiv.) were then added. The resulting mixture was stirred at 19 °C for 20 min and frozen at –20 °C for 30 min. The desired enone (2.00 µL, stock solution 0.05 M in DMSO, 0.10 µmol, 1.00 equiv.) was added and the mixture was frozen again at –20 °C for 30 min. Indole (2.00 µL, 0.05 M in DMSO, 0.10 µmol, 1.00 equiv.) was finally added in a cold room. The resulting solution was stirred at 4 °C for 1 d in a thermoshaker placed in a cold room. The same work-up used in general procedure N was followed.

General procedure Q: Enantioselective Friedel-Crafts reactions and Michael additions (nitromethane, malononitrile and *tert*-butyl cyanoacetate) - supramolecular approach with st-DNA

st-DNA solution (15.0 µL, stock solution 4.50 mg/mL or 6.92 mM (bp) in Milli-Q H_2O, 1.00 M final concentration) and Milli-Q H_2O (61.0 µL) were successively added to a 500 µL Eppendorf® safe-lock tube. The reaction mixture was briefly mixed. MOPS buffer solution (10.0 µL, stock solution 200 mM in Milli-Q H_2O, pH 6.5, 1/10 V_{tot}) and $Cu(NO_3)_2$.dmbpy solution (14.4 µL, stock solution 0.90 mM in Milli-Q H_2O prepared 24 h in advance, 0.13 equiv.) were then added. The resulting mixture was stirred at 19 °C for 20 min and frozen at −20 °C for 30 min. The Eppendorf® safe-lock tube was brought into the cold room and the desired enone (2.00 µL, stock solution 0.05 M in DMSO, 0.10 µmol, 1.00 equiv.) was added. Indole (2.00 µL, 0.25 M in DMSO, 0.50 µmol, 5.00 equiv.) **OR** nitromethane (5.40 µL, 100 µmol, 1000 equiv.) **OR** malononitrile (10.0 µL, 1.00 M in DMSO, 10.0 µmol, 100 equiv.) **OR** *tert*-butyl cyanoacetate (1.40 µL, 10.0 µmol, 100 equiv.) was finally added in a cold room. The resulting solution was stirred at 4 °C for 3 d in a thermoshaker placed in a cold room. The same work-up used in general procedure N was followed.

General procedure R: Enantioselective Michael additions of dimethyl malonate - supramolecular approach with st-DNA

st-DNA solution (15.0 µL, stock solution 4.50 mg/mL or 6.92 mM (bp) in Milli-Q H_2O, 1.00 M final concentration) and Milli-Q H_2O (61.0 µL) were successively added to a 500 µL Eppendorf® safe-lock tube. The reaction mixture was briefly mixed. MOPS buffer solution (10.0 µL, stock solution 200 mM in Milli-Q H_2O, pH 7.9, 1/10 V_{tot}) and $Cu(NO_3)_2$.dmbpy solution (14.4 µL, stock solution 0.90 mM in Milli-Q H_2O prepared 24 h in advance, 0.13 equiv.) were then added. The resulting mixture was stirred at 19 °C for 20 min and frozen at −20 °C for 30 min. The desired enone (2.00 µL, stock solution 0.05 M in DMSO, 0.10 µmol, 1.00 equiv.) was added and the mixture was frozen again at −20 °C for 30 min. Dimethyl malonate (1.20 µL, 10.0 µmol, 100 equiv.) was finally added in a cold room. The resulting solution was stirred at 4 °C for 3 d in a thermoshaker placed in a cold room. The same work-up used in general procedure N was followed.

General procedure S: Sequential Friedel-Crafts alkylation/enantioselective protonation - supramolecular approach with st-DNA

st-DNA solution (15.0 µL, stock solution 4.50 mg/mL or 6.92 mM (bp) in Milli-Q H_2O, 1.00 M final concentration) and Milli-Q H_2O (61.0 µL) were successively added to a 500 µL Eppendorf® safe-lock tube. The reaction mixture was briefly mixed. MES buffer solution (10.0 µL, stock solution 200 mM in Milli-Q H_2O, pH 5.0, 1/10 V_{tot}) and $Cu(NO_3)_2$.dmbpy solution (14.4 µL, stock solution 0.90 mM in Milli-Q H_2O prepared 24 h in advance, 0.13 equiv.) were then added. The resulting mixture was stirred at 19 °C for 20 min and frozen at −20 °C for 30 min. The desired enone (2.00 µL, stock solution 0.05 M in DMSO, 0.10 µmol, 1.00 equiv.) was added and the mixture was frozen again at −20 °C for 30 min. Indole (2.00 µL, 0.05 M in DMSO, 0.10 µmol, 1.00 equiv.) was finally added in a cold room. The resulting solution was stirred at 4 °C for 1 d in a thermoshaker placed in a cold room. The same work-up used in general procedure N was followed.

D. Description of products

3-(5-Methoxy-1H-indol-3-yl)-1-(1-methyl-1H-imidazol-2-yl)butan-1-one (3a)

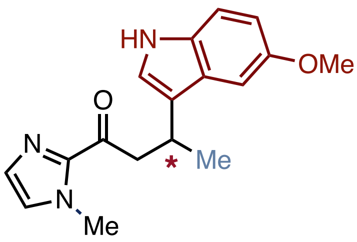

MW (g/mol): 297.35 **Molecular formula:** $C_{17}H_{19}N_3O_2$

Synthesised according racemic procedure using **1a**. The desired product was obtained as a brown solid (95.0 mg, 0.32 mmol, 91% yield) after purification by flash column chromatography over silica gel (Ethyl acetate/Pentane, 50:50). The spectroscopic data of the product were identical with those reported in the literature.[12]

^{1}H NMR (400 MHz, CDCl$_3$) δ 8.18 (br s, 1H, -*NH*), 7.20 (d, *J* = 8.8 Hz, 1H), 7.17-7.15 (m, 2H), 7.02 (d, *J* = 2.5 Hz, 1H), 7.00 (br s, 1H), 6.82 (dd, *J* = 8.8, 2.5 Hz, 1H), 3.94 (s, 3H), 3.86 (s, 3H), 3.77 (m, 1H), 3.58 (dd, *J* = 15.6, 6.5 Hz, 1H), 3.40 (dd, *J* = 15.6, 8.8 Hz, 1H), 1.40 (d, *J* = 6.5 Hz, 3H).

^{13}C NMR (101 MHz, CDCl$_3$) δ 192.3, 153.7, 143.3, 131.5, 128.8, 126.9, 126.8, 121.1, 120.9, 112.1, 111.7, 101.0, 55.9, 46.8, 36.2, 27.1, 21.4.

HPLC: Chiralpak IA column, T = 20 °C, *n*-Hexane/*i*-PrOH = 90:10, 1 mL/min, λ = 254 nm.

Entry	General procedure	Sequences	Conversion (%)[a]	ee (%)[a]
**1**	N	**DNA-SerC2/RNA-U**	99	+94
**2**	Q	st-DNA	78[b]	+82

[a]Determined by HPLC. The sign before the ee's values is arbitrary evaluated. [b] Isolated yield.

Racemic Chiralpak IA column, T = 20 °C, *n*-Hexane/*i*-PrOH = 90:10, 1 mL/min, λ = 254 nm, t_R = 38.652 min and t_R = 41.236 min.

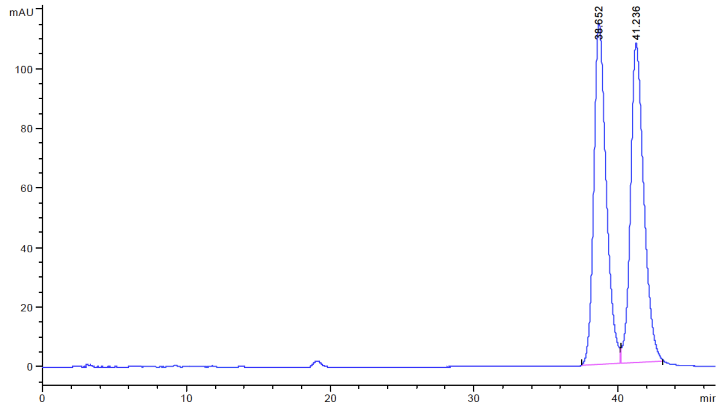

Peak	Retention Time	Rel. Area
	min	%
1	38,652	49,9684
2	41,236	50,0316

Starting material 1a Chiralpak IA column, T = 20 °C, *n*-Hexane/*i*-PrOH = 90:10, 1 mL/min, λ = 254 nm, t_R = 7.377 min.

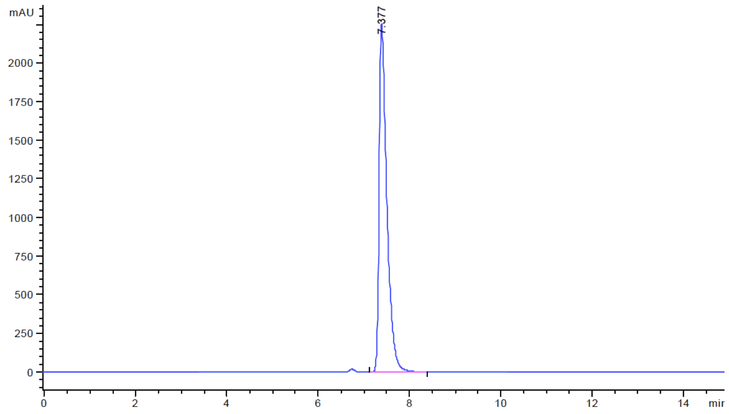

Indole Chiralpak IA column, T = 20 °C, *n*-Hexane/*i*-PrOH = 90:10, 1 mL/min, λ = 254 nm, t_R = 6.334 min

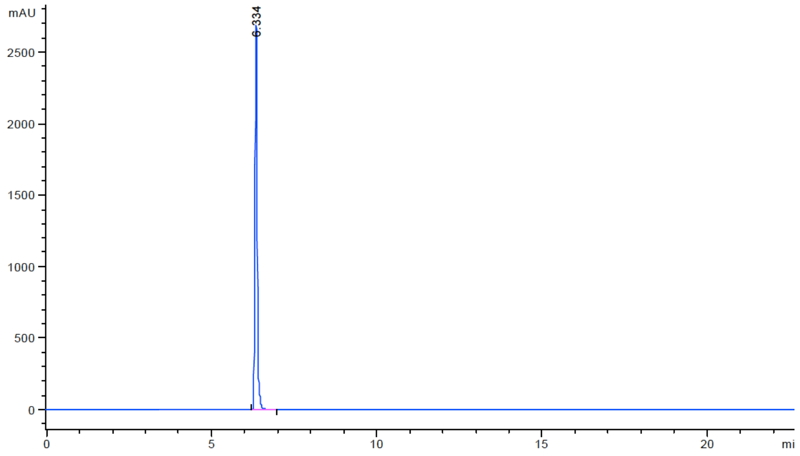

Syn-hydration product Chiralpak IA column, T = 20 °C, *n*-Hexane/*i*-PrOH = 90:10, 1 mL/min, λ = 254 nm, t_R = 16.804 min

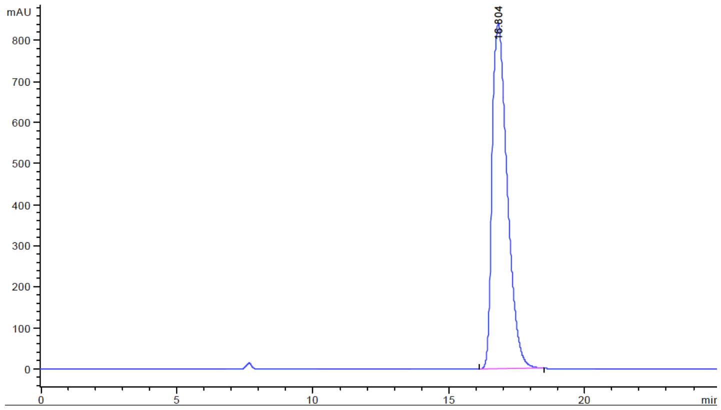

Following the general procedure N. HPLC analysis of the crude residue indicated a ratio **1a/3a** of 1:99 and an enantiomeric excess of (+) 95 [Chiralpak IA column, T = 20 °C, *n*-Hexane/*i*-PrOH = 90:10, 1 mL/min, λ = 254 nm t_R = 38.571 min and t_R = 41.178 min].

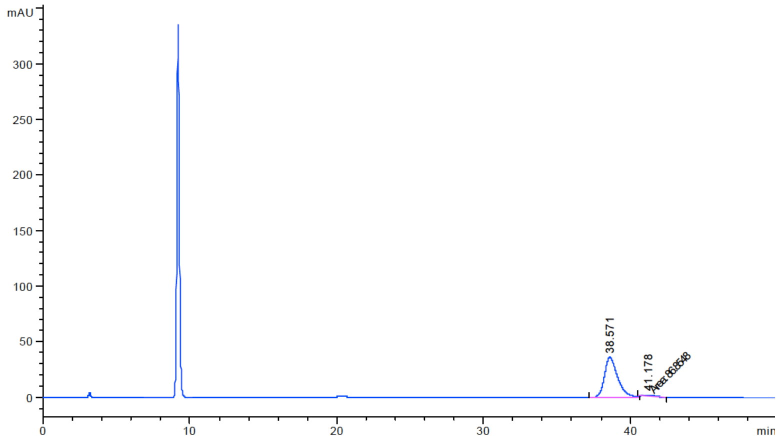

Peak	Retention Time	Rel. Area
	min	%
1	38,571	97,4434
2	41,178	2,5566

3-(5-Methoxy-1*H*-indol-3-yl)-1-(1-methyl-1*H*-imidazol-2-yl)hexan-1-one (3b)

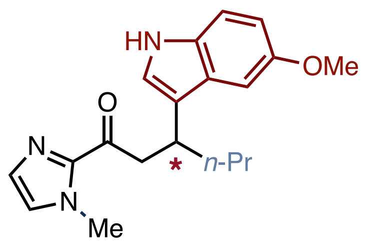

MW (g/mol): 325.41 **Molecular formula:** $C_{19}H_{23}N_3O_2$

Synthesised according racemic procedure using **1b**. The desired product was obtained as a brown oil (77.0 mg, 0.24 mmol, 68% yield) after purification by flash column chromatography over silica gel (Ethyl acetate/Pentane, 50:50). The spectroscopic data of the product were identical with those reported in the literature.[13]

^{1}H NMR (400 MHz, $CDCl_3$) δ 8.41 (br s, 1H, -*NH*), 7.16 (d, *J* = 8.8 Hz, 1H), 7.12 (d, *J* = 0.9 Hz, 1H), 7.07 (d, *J* = 2.4 Hz, 1H), 6.96 (d, *J* = 2.4 Hz, 1H), 6.94 (d, *J* = 0.9 Hz, 1H), 6.78 (dd, *J* = 8.8, 2.4 Hz, 1H), 3.83 (s, 6H), 3.69 (m, 1H), 3.51 (d, *J* = 7.3 Hz, 2H), 1.78-1.69 (m, 2H), 1.37-1.19 (m, 2H), 0.86 (t, *J* = 7.3 Hz, 3H).

^{13}C NMR (101 MHz, $CDCl_3$) δ 192.3, 153.7, 143.4, 131.5, 128.7, 127.5, 126.8, 121.9, 119.3, 111.8, 111.7, 101.2, 55.9, 45.7, 38.5, 36.0, 32.1, 20.6, 14.1.

HPLC: Chiralpak IC column, T = 20 °C, *n*-Hexane/*i*-PrOH = 90:10, 1 mL/min, λ = 254 nm.

Entry	General procedure	Sequences	Conversion (%)[a]	ee (%)[a]
**1**	N	**DNA-SerC2/RNA-U**	95	+97
**2**	R	st-DNA	98	+35

[a]Determined by HPLC. The sign before the ee's values is arbitrary evaluated.

Racemic Chiralpak IC column, T = 20 °C, *n*-Hexane/*i*-PrOH = 90:10, 1 mL/min, λ = 254 nm, t_R = 31.164 min and t_R = 33.703 min.

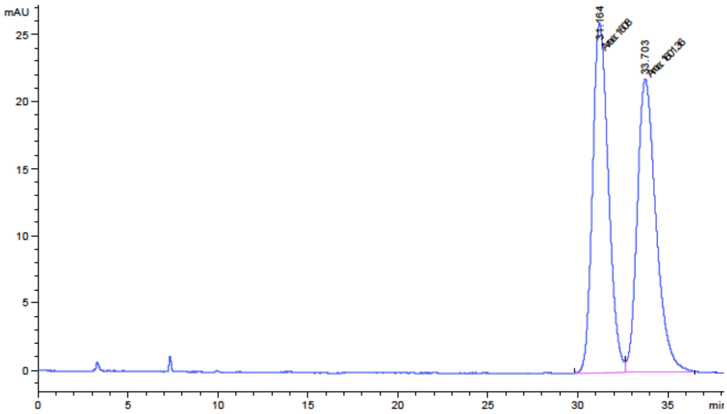

Peak	Retention Time	Rel. Area
	min	%
1	31,164	50,1034
2	33,703	49,8966

Starting material 1b Chiralpak IC column, T = 20 °C, *n*-Hexane/*i*-PrOH = 90:10, 1 mL/min, λ = 254 nm, t_R = 36.647 min

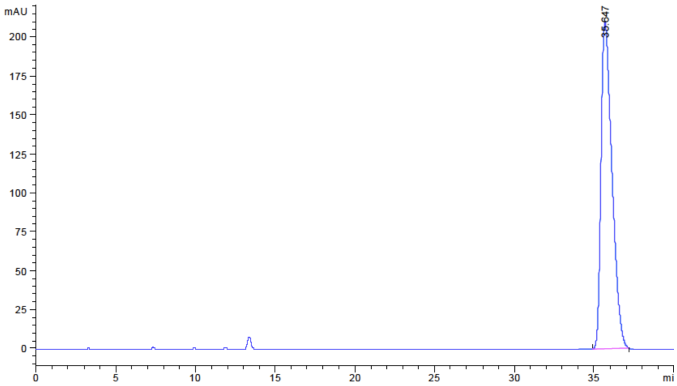

Indole Chiralpak IC column, T = 20 °C, *n*-Hexane/*i*-PrOH = 90:10, 1 mL/min, λ = 254 nm, t_R = 8.922 min.

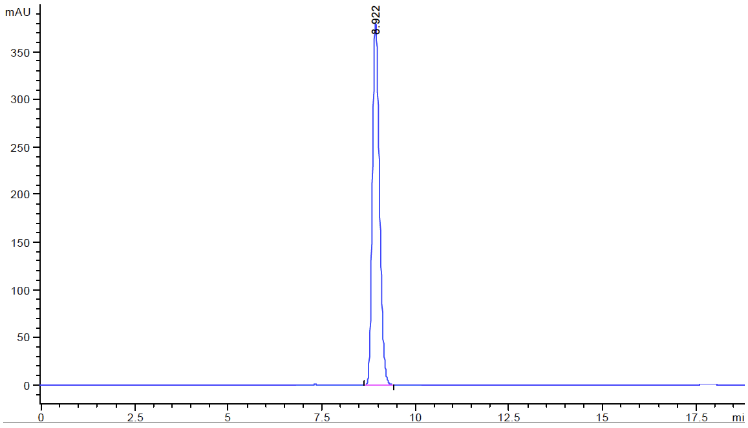

Following the general procedure N. HPLC analysis of the crude residue indicated a ratio **1b**/**3b** of 5:95 and an enantiomeric excess of (+) 97% [Chiralpak IC column, T = 20 °C, *n*-Hexane/*i*-PrOH = 90:10, 1 mL/min, λ = 254 nm, t_R = 28.968 min and t_R = 32.700 min].

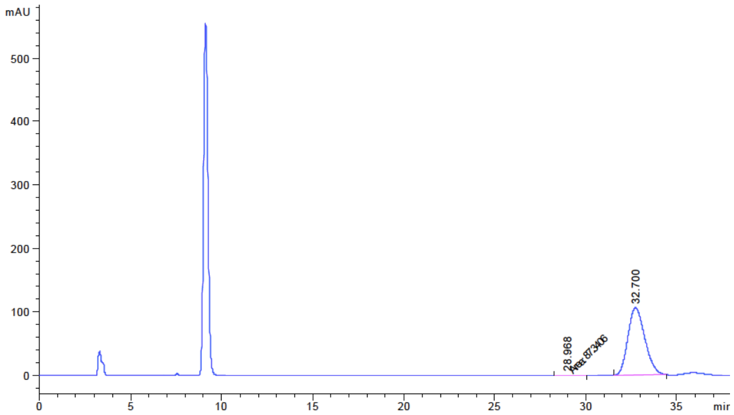

Peak	Retention Time	Rel. Area
	min	%
1	28,968	1,2357
2	32,7	98,7643

3-(5-Methoxy-1*H*-indol-3-yl)-3-(4-methoxyphenyl)-1-(1-methyl-1*H*-imidazol-2-yl)propan-1-one (3c)

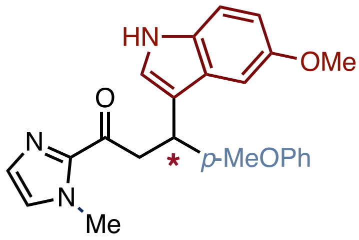

MW (g/mol): 389.46 **Molecular formula:** $C_{23}H_{23}N_3O_3$

Synthesised according racemic procedure using **1c**. The desired product was obtained as a brown oil (77.0 mg, 0.24 mmol, 68% yield) after purification by flash column chromatography over silica gel (Ethyl acetate/Pentane, 50:50). The spectroscopic data of the product were identical with those reported in the literature.[12]

^{1}H NMR (400 MHz, $CDCl_3$) δ 8.81 (br s, 1H, -*NH*), 7.28-7.24 (m, 2H), 7.16 (s, 1H), 7.13 (d, *J* = 8.8 Hz, 1H), 7.01 (d, *J* = 2.4 Hz, 1H), 6.97-6.90 (m, 2H and H_{Ar}), 6.80-6.72 (m, 3H and H_{Ar}), 4.97 (t, *J* = 7.6 Hz, 1H), 3.98-3.83 (m, 2H), 3.86 (s, 3H), 3.76 (s, 3H), 3.71 (s, 3H).

^{13}C NMR (101 MHz, $CDCl_3$) δ 191.2, 157.8, 153.7, 143.2, 136.4, 131.6, 128.8 (2C), 128.7, 127.2, 126.9, 122.1, 119.5, 113.7 (2C), 112.1, 111.6, 101.5, 55.8, 55.2, 45.5, 37.4, 36.2.

HPLC: Chiralpak IA column, T = 20 °C, *n*-Hexane/*i*-PrOH = 80:20, 1 mL/min, λ = 254 nm.

Entry	General procedure	Sequences	Conversion (%)[a]	ee (%)[a]
**1**	N	**DNA-SerC2/RNA-U**	27	+90
**2**	Q	st-DNA	71	+64

[a]Determined by HPLC. The sign before the ee's values is arbitrary evaluated. [b] Isolated yield.

Racemic Chiralpak IA column, T = 20 °C, *n*-Hexane/*i*-PrOH = 80:20, 1 mL/min, λ = 254 nm, t_R = 29.634 min and t_R = 46.848 min.

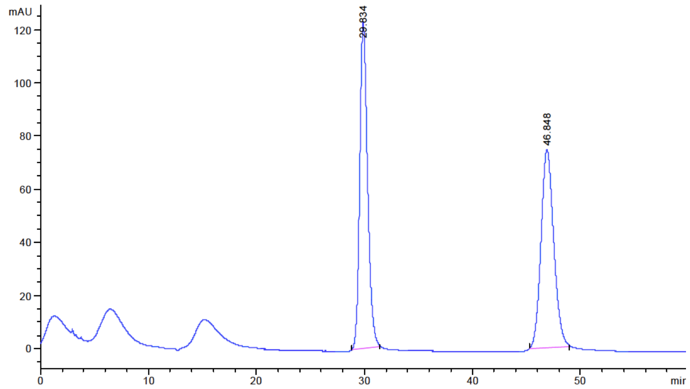

Peak	Retention Time	Rel. Area
	min	%
1	29,834	50,3148
2	46,848	49,6852

Starting material 1c Chiralpak IA column, T = 20 °C, *n*-Hexane/*i*-PrOH = 80:20, 1 mL/min, λ = 254 nm, t_R = 11.039 min.

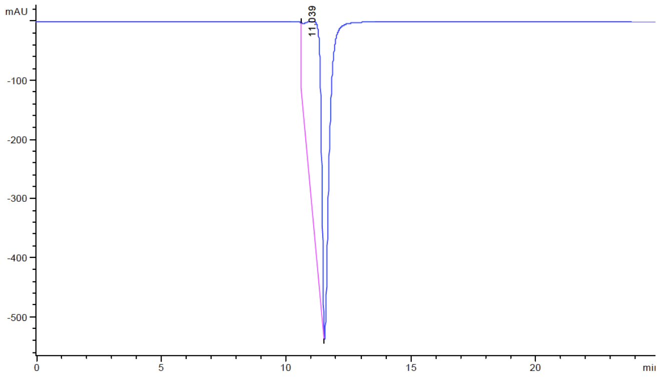

Indole Chiralpak IA column, T = 20 °C, *n*-Hexane/*i*-PrOH = 80:20, 1 mL/min, λ = 254 nm, t_R = 8.922 min.

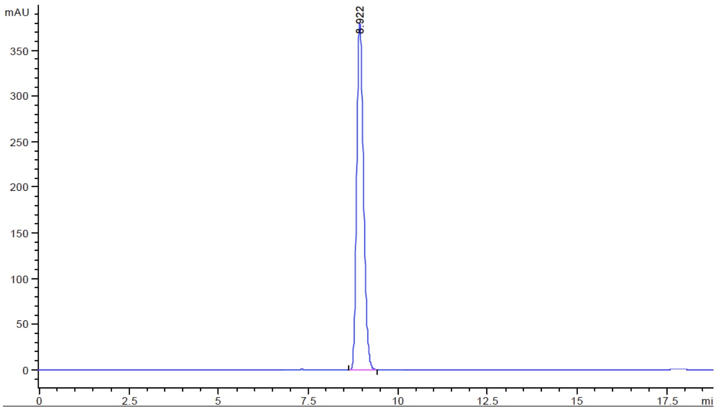

Following the general procedure N. HPLC analysis of the crude residue indicated a ratio **1c**/**3c** of 73:27 and an enantiomeric excess of (+) 90% [Chiralpak IA column, T = 20 °C, *n*-Hexane/*i*-PrOH = 80:20, 1 mL/min, λ = 254 nm t_R = 29.070 min and t_R = 42.778 min].

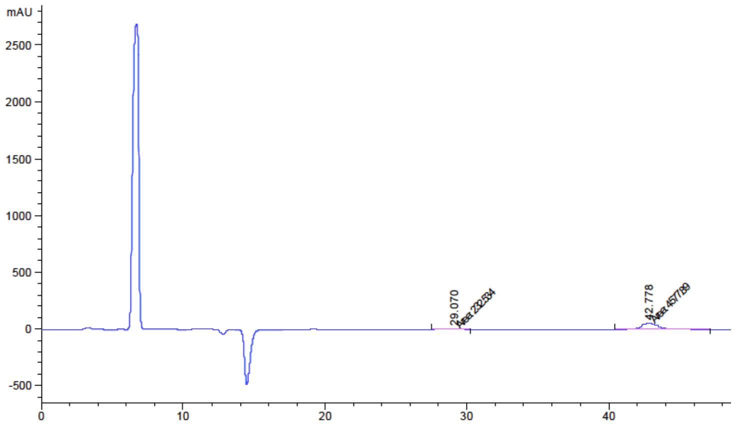

Peak	Retention Time	Rel. Area
	min	%
1	29,07	4,834
2	42,778	95,166

3-(1*H*-indol-3-yl)-1-(1-methyl-1*H*-imidazol-2-yl)butan-1-one (3d)

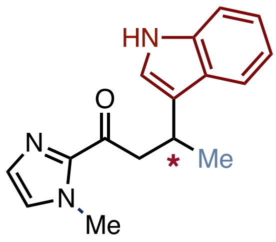

MW (g/mol): 267.33 **Molecular formula:** $C_{16}H_{17}N_3O$

Synthesised according racemic procedure using **1a**. The desired product was obtained as a pale yellow oil (85.0 mg, 0.32 mmol, 92% yield) after purification by flash column chromatography over silica gel. The spectroscopic data of the product were identical with those reported in the literature.[12]

^{1}H NMR (400 MHz, CDCl$_3$) δ 8.18 (br s, 1H), 7.67 (dd, *J* = 7.4, 1.9 Hz, 1H), 7.32 (dd, *J* = 8.2, 0.9 Hz, 1H), 7.16-714 (m, 2H), 7.09 (ddd, *J* = 8.0, 7.1, 1.1 Hz, 1H), 7.04 (d, *J* = 2.1 Hz, 1H), 6.99 (s, 1H), 3.93 (s, 3H), 3.85 (m, 1H), 3.57 (dd, *J* = 15.8, 6.4 Hz, 1H), 3.46 (dd, *J* = 15.8, 8.2 Hz, 1H), 1.42 (d, *J* = 6.9 Hz, 3H).

^{13}C NMR (101 MHz, CDCl$_3$) δ 192.4, 143.3, 136.4, 128.9, 126.9, 126.6, 121.9, 121.5, 120.2, 119.3, 119.1, 111.1, 46.7, 36.2, 27.2, 22.7, 14.1.

HPLC: Chiralpak ID column, T = 30 °C, n-Hexane/i-PrOH = 90:10, 1 mL/min, λ = 280 nm.

Entry	General procedure	Sequences	Conversion (%)[a]	ee (%)[a]
**1**	N	**DNA-SerC2/RNA-U**	>99	+88
**2**	Q	st-DNA	79[b]	+72

[a]Determined by HPLC. The sign before the ee’s values is arbitrary evaluated. [b] Isolated yield.

Racemic Chiralpak ID column, T = 30 °C, n-Hexane/i-PrOH = 90:10, 1 mL/min, λ = 280 nm, t_R = 21.755 min, t_R = 26.275 min.

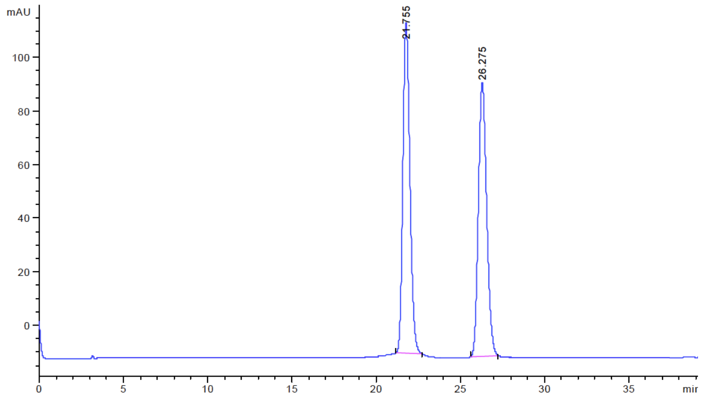

Peak	Retention Time	Rel. Area
	min	%
1	21,755	50,4013
2	26,275	49,5987

Starting material 1a Chiralpak ID column, T = 30 °C, n-Hexane/i-PrOH = 90:10, 1 mL/min, λ = 280 nm, t_R = 12.619 min.

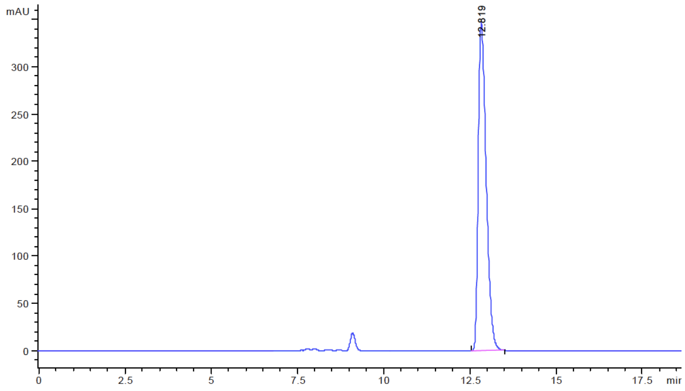

Indole Chiralpak ID column, T = 30 °C, n-Hexane/i-PrOH = 90:10, 1 mL/min, λ = 280 nm, t_R = 4.375 min.

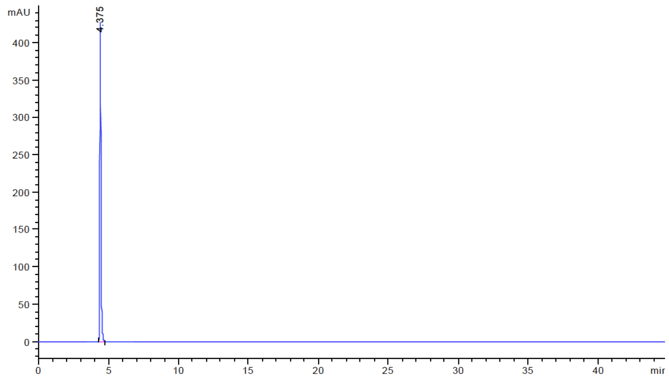

Following the general procedure N. HPLC analysis of the crude residue indicated a ratio **1a/3d** of 1:99 and an enantiomeric excess of (+) 88% [Chiralpak ID column, T = 30 °C, n-Hexane/i-PrOH = 90:10, 1 mL/min, λ = 280 nm, t_R = 22.627 min and t_R = 26.886 min].

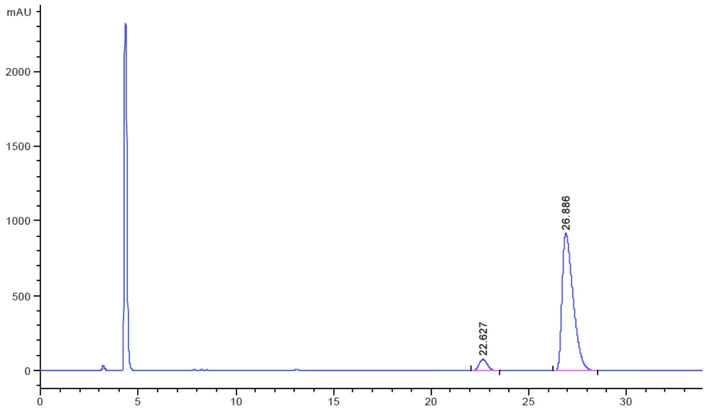

Peak	Retention Time	Rel. Area
	min	%
1	22,627	6,143
2	26,886	93,857

1-(1-Methyl-1*H*-imidazol-2-yl)-3-(2-methyl-1*H*-indol-3-yl)butan-1-one (3e)

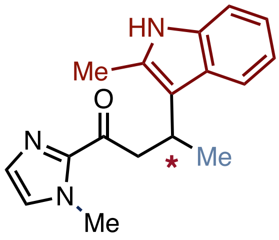

MW (g/mol): 281.36 **Molecular formula:** $C_{17}H_{19}N_3O$

Synthesised according racemic procedure using **1a**. The desired product was obtained as a dark yellow oil (82.0 mg, 0.29 mmol, 82% yield) after purification by flash column chromatography over silica gel. The spectroscopic data of the product were identical with those reported in the literature.[12]

^{1}H NMR (400 MHz, $CDCl_3$) δ 7.78 (br s, 1H), 7.65 (dd, *J* = 8.1, 6.5 Hz, 1H), 7.20 (m, 1H), 7.10 (d, *J* = 0.8 Hz, 1H), 7.10-7.00 (m, 2H), 6.93 (s, 1H), 3.85 (s, 3H), 3.79 (dt, *J* = 14.1, 7.1 Hz, 1H), 3.64 (dd, *J* = 13.2, 4.0 Hz, 1H), 3.64 (dd, *J* = 13.2, 4.9 Hz, 1H), 2.95 (s, 3H), 1.47 (d, *J* = 7.1 Hz, 3H).

^{13}C NMR (101 MHz, $CDCl_3$) δ 192.3, 143.3, 135.3, 130.4, 128.8, 127.4, 126.6, 120.5, 119.2, 118.9, 115.4, 110.2, 46.2, 36.0, 27.1, 21.1, 12.2.

HPLC: Chiralpak IA column, T = 30 °C, *n*-Hexane/*i*-PrOH = 90:10, 1 mL/min, λ = 280 nm.

Entry	General procedure	Sequences	Conversion (%)[a]	ee (%)[a]
**1**	N	**DNA-SerC2/RNA-U**	98	+91
**2**	Q	st-DNA	45[b]	+31

[a]Determined by HPLC. The sign before the ee's values is arbitrary evaluated. [b] Isolated yield.

Racemic Chiralpak IA column, T = 30 °C, *n*-Hexane/*i*-PrOH = 90:10, 1 mL/min, λ = 280 nm, t_R = 18.359 min, t_R = 21.862 min.

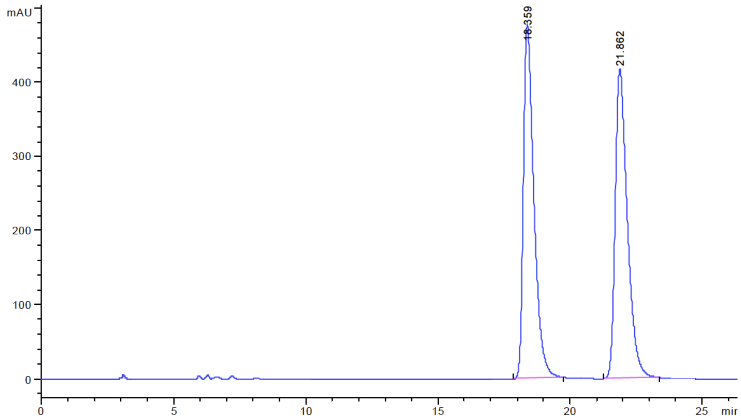

Peak	Retention Time	Rel. Area
	min	%
1	18,359	49,9743
2	21,862	50,0257

Starting material 1a Chiralpak IA column, T = 30 °C, *n*-Hexane/*i*-PrOH = 90:10, 1 mL/min, λ = 280 nm, t_R = 7.377 min.

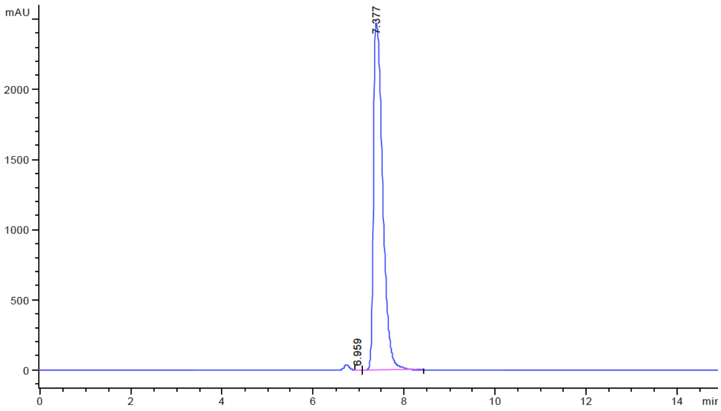

Indole Chiralpak IA column, T = 30 °C, *n*-Hexane/*i*-PrOH = 90:10, 1 mL/min, λ = 280 nm, t_R = 5.755 min.

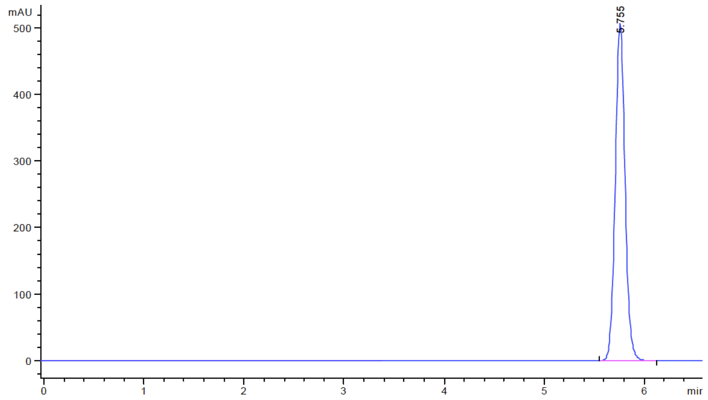

Following the general procedure N. HPLC analysis of the crude residue indicated a ratio **1a/3e** of 2:98 and an enantiomeric excess of (+) 91% [Chiralpak IA column, T = 30 °C, *n*-Hexane/*i*-PrOH = 90:10, 1 mL/min, λ = 280 nm, t_R = 17.836 min and t_R = 21.739 min].

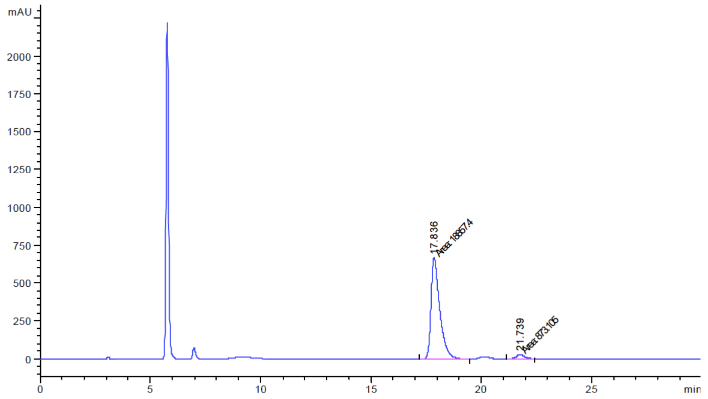

Peak	Retention Time	Rel. Area
	min	%
1	17,836	95,5748
2	21,739	4,4252

1-(1-Methyl-1*H*-imidazol-2-yl)-3-(1-methyl-1*H*-indol-3-yl)butan-1-one (3f)

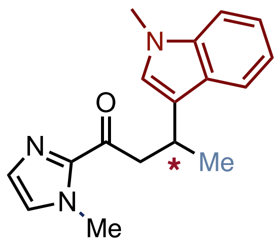

MW (g/mol): 281.36 **Molecular formula:** $C_{17}H_{19}N_3O$

Synthesised according racemic procedure using **1a**. The desired product was obtained as a brown oil (65.0 mg, 0.23 mmol, 66% yield) after purification by flash column chromatography over silica gel. The spectroscopic data of the product were identical with those reported in the literature.[12]

^{1}H NMR (400 MHz, CDCl$_3$) δ 7.65 (m, 1H), 7.26 (m, 1H), 7.19 (m, 1H), 7.14 (d, J = 7.2 Hz, 1H), 7.07 (ddd, *J* = 8.0, 6.9, 1.1 Hz, 1H), 6.99 (s, 1H), 6.93 (s, 1H), 3.92 (s, 3H), 3.84 (dd, *J* = 14.2, 7.0 Hz, 1H), 3.72 (s, 3H), 3.56 (dd, *J* = 15.8, 6.5 Hz, 1H), 3.45 (dd, *J* = 15.8, 8.0 Hz, 1H), 1.42 (d, J = 6.9 Hz, 1H).

^{13}C NMR (101 MHz, CDCl$_3$) δ 192.4, 143.4, 137.1, 128.9, 127.0, 126.8, 125.1, 121.4, 120.0, 119.4, 118.5, 109.1, 46.9, 36.1, 32.6, 27.2, 21.9.

HPLC: Chiralpak IA column, T = 30 °C, *n*-Hexane/*i*-PrOH = 93:7, 1 mL/min, λ = 280 nm.

Entry	General procedure	Sequences	Conversion (%)[a]	ee (%)[a]
**1**	N	**DNA-SerC2/RNA-U**	76	+54
**2**	Q	st-DNA	68[b]	+81

[a]Determined by HPLC. The sign before the ee's values is arbitrary evaluated. [b] Isolated yield.

Racemic Chiralpak IA column, T = 30 °C, *n*-Hexane/*i*-PrOH = 93:7, 1 mL/min, λ = 280 nm, t_R = 12.436 min, t_R = 14.989 min.

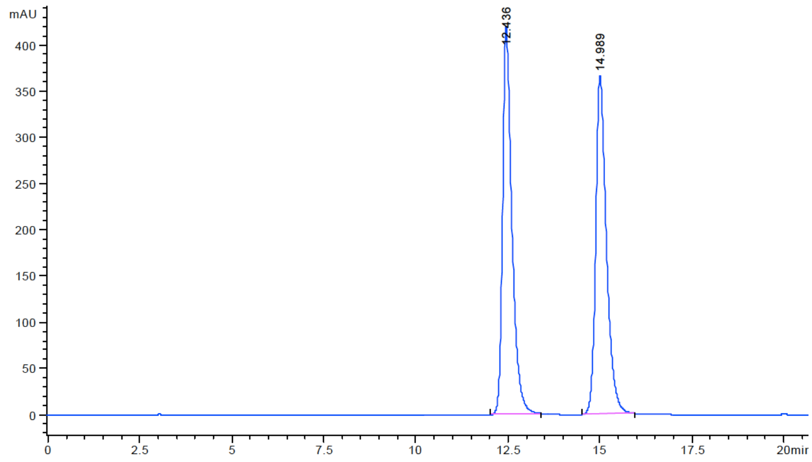

Peak	Retention Time	Rel. Area
	min	%
1	12,436	49,9749
2	14,989	50,0251

Starting material 1a Chiralpak IA column, T = 30 °C, *n*-Hexane/*i*-PrOH = 93:7, 1 mL/min, λ = 280 nm, t_R = 8.064 min.

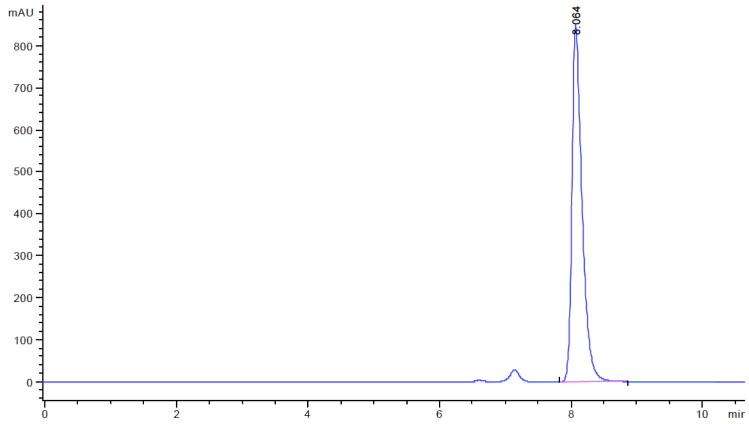

Indole Chiralpak IA column, T = 30 °C, *n*-Hexane/*i*-PrOH = 93:7, 1 mL/min, λ = 280 nm, t_R = 3.984 min.

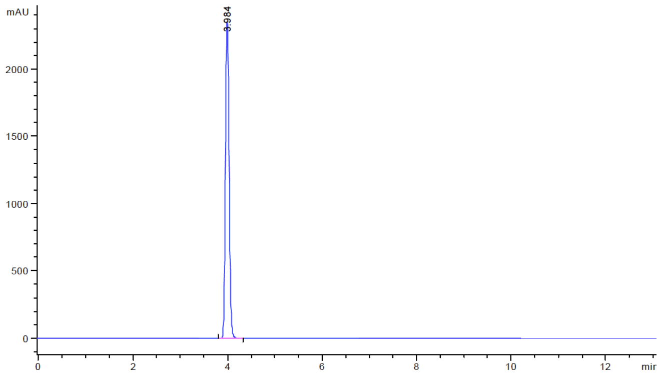

Following the general procedure N. HPLC analysis of the crude residue indicated a ratio **1a/3f** of 24:76 and an enantiomeric excess of (+) 54% [Chiralpak IA column, T = 30 °C, *n*-Hexane/*i*-PrOH = 93:7, 1 mL/min, λ = 280 nm, t_R = 12.688 min and t_R = 15.455 min].

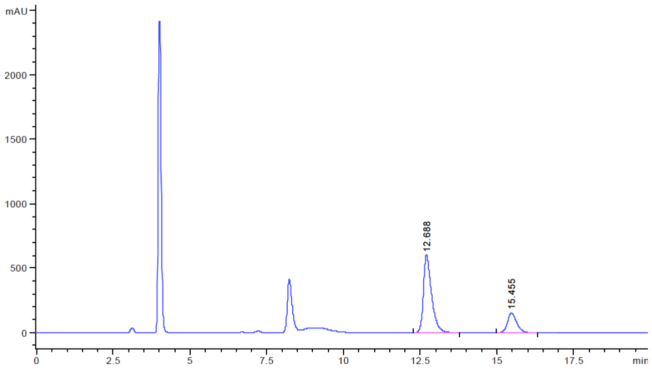

Peak	Retention Time	Rel. Area
	min	%
1	12,688	77,1955
2	15,455	22,8045

(1-(1-Methyl-1*H*-imidazol-2-yl)-3-(1-methyl-1*H*-indol-3-yl)-3-phenylpropan-1-one (3g)

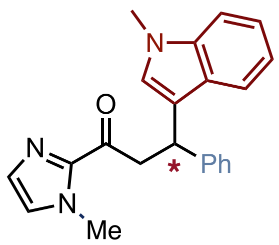

MW (g/mol): 343.43 **Molecular formula:** $C_{22}H_{21}N_3O$

Synthesised according racemic procedure using **1d**. The desired product was obtained as a pale yellow solid (93.0 mg, 0.27 mmol, 77% yield) after purification by flash column chromatography over silica gel. The spectroscopic data of the product were identical with those reported in the literature.[12]

^{1}H NMR (400 MHz, CDCl$_3$) δ 7.50 (d, *J* = 8.0 Hz, 1H), 7.41 (d, *J* = 16.0 Hz, 1H), 7.27-7.23 (m, 3H), 7.20-7.12 (m, 3H), 7.01 (m, 1H), 7.01 (s, 1H), 6.96 (s, 1H), 7.08 (d, *J* = 0.9 Hz, 1H), 5.07 (t, *J* = 7.6 Hz, 1H), 4.10 (s, 3H), 4.02 (dd, *J* = 16.5, 7.5 Hz, 1H), 3.90-3.84 (m, 4H), 3.71 (s, 3H),

^{13}C NMR (101 MHz, CDCl$_3$) δ 191.1, 144.7, 143.3, 137.2, 129.0, 128.3, 127.9, 126.9, 126.3, 126.1, 121.6, 119.6, 118.8, 117.9, 109.1, 45.5, 38.1, 36.1, 32.7.

HPLC: Chiralpak IA column, T = 25 °C, *n*-Hexane/*i*-PrOH = 92:8, 1 mL/min, λ = 280 nm.

Entry	General procedure	Sequences	Conversion (%)[a]	ee (%)[a]
**1**	N	**DNA-SerC2/RNA-U**	79	+73
**2**	Q	st-DNA	87[b]	+75

[a]Determined by HPLC. The sign before the ee's values is arbitrary evaluated. [b] Isolated yield.

Racemic Chiralpak IA column, T = 25 °C, *n*-Hexane/*i*-PrOH = 92:8, 1 mL/min, λ = 280 nm, t_R = 20.36 min, t_R = 21.63 min.

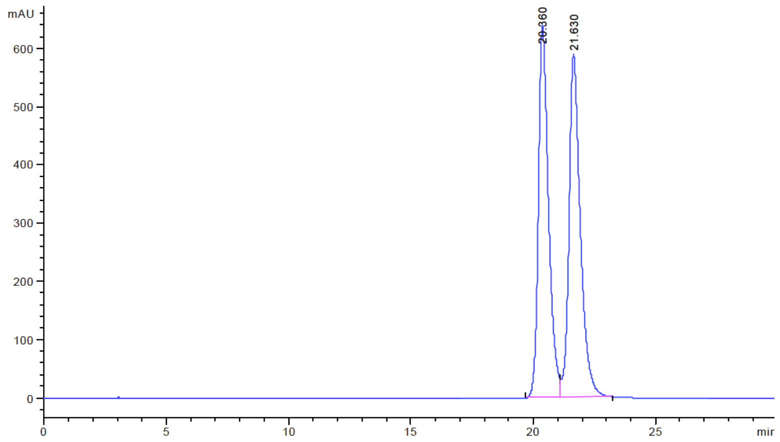

Peak	Retention Time	Rel. Area
	min	%
1	20,36	49,3242
2	21,63	50,6758

Starting material 1d Chiralpak IA column, T = 25 °C, *n*-Hexane/*i*-PrOH = 92:8, 1 mL/min, λ = 280 nm, t_R = 14.083 min.

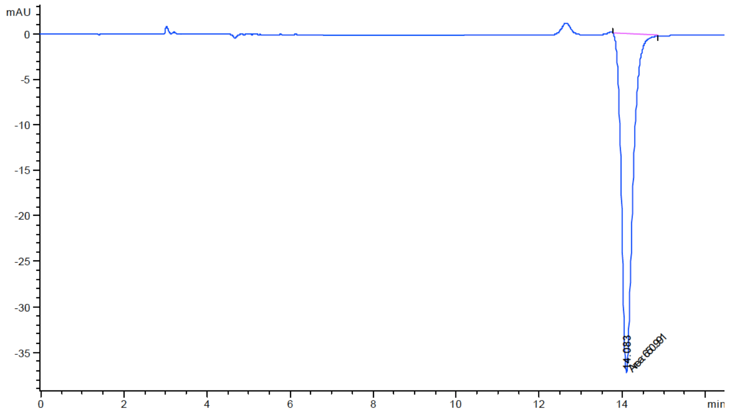

Indole Chiralpak IA column, T = 25 °C, *n*-Hexane/*i*-PrOH = 92:8, 1 mL/min, λ = 280 nm, t_R = 3.998 min.

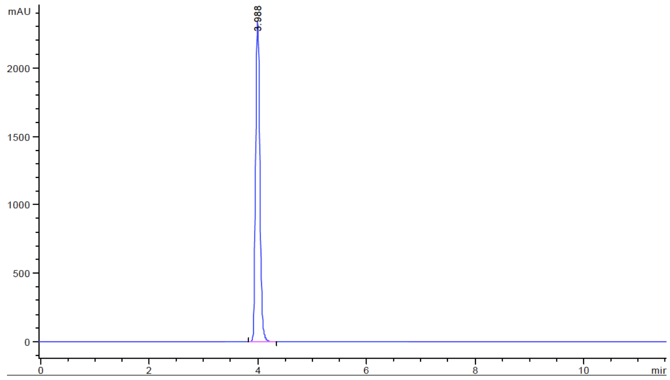

Following the general procedure N. HPLC analysis of the crude residue indicated a ratio **1d/3g** of 21:79 and an enantiomeric excess of (+) 73% [Chiralpak IA column, T = 25 °C, *n*-Hexane/*i*-PrOH = 92:8, 1 mL/min, λ = 280 nm, t_R = 20.205 min and t_R = 21.706 min].

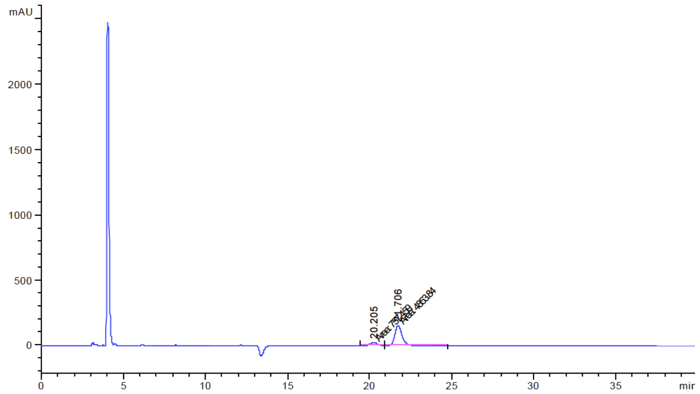

Peak	Retention Time	Rel. Area
	min	%
1	20,205	13,4988
2	21,706	86,5012

3-Methyl-1-(1-methyl-1*H*-imidazol-2-yl)-4-nitrobutan-1-one (4a)

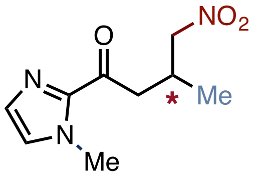

MW (g/mol): 211.22 **Molecular formula:** $C_9H_{13}N_3O_3$

Synthesised according racemic procedure using **1a**. The desired product was obtained as a brown solid (45.0 mg, 0.21 mmol, 65% yield) after purification by flash column chromatography over silica gel (Ethyl acetate/Petroleum ether, 50:50). The spectroscopic data of the product were identical with those reported in the literature.[14]

^{1}H NMR (400 MHz, CDCl$_3$) δ 7.11 (br s, 1H), 7.04 (br s, 1H), 4.50 (dd, *J* =12.0, 5.8 Hz, 1H), 4.32 (dd, *J* = 12.0, 7.8 Hz, 1H), 3.98 (s, 3H), 3.29-3.12 (m, 2H), 2.96 (m, 1H), 1.11 (d, *J* = 6.8 Hz, 3H).

^{13}C NMR (101 MHz, CDCl$_3$) δ 190.3, 142.9, 129.4, 127.5, 80.8, 42.4, 36.3, 29.0, 17.8.

HPLC: Chiralpak IC column, T = 20 °C, *n*-Hexane/*i*-PrOH = 90:10, 1 mL/min, λ = 254 nm.

Entry	General procedure	Sequences	Conversion (%)[a]	ee (%)[a]
**1**	N	**DNA-SerC2/RNA-U**	99	+97
**2**	Q	st-DNA	95	+62

[a]Determined by HPLC. The sign before the ee's values is arbitrary evaluated.

Racemic Chiralpak IC column, T = 20 °C, *n*-Hexane/*i*-PrOH = 90:10, 1 mL/min, λ = 254 nm, t_R = 20.725 min and t_R = 25.419 min.

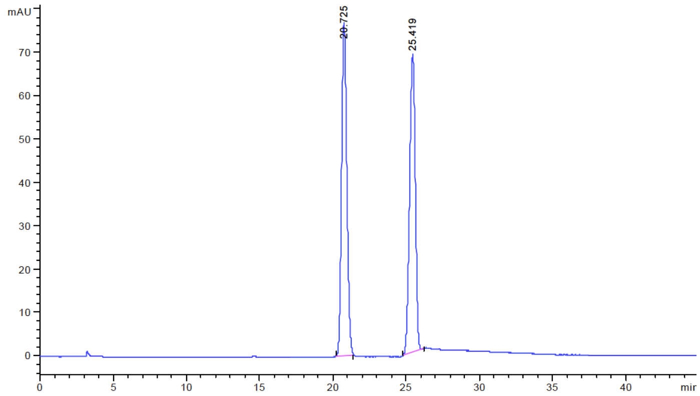

Peak	Retention Time	Rel. Area
	min	%
1	20,725	50,563
2	25,419	49,437

Starting material 1a Chiralpak IC column, T = 20 °C, *n*-Hexane/*i*-PrOH = 90:10, 1 mL/min, λ = 254 nm, t_R = 32.430 min.

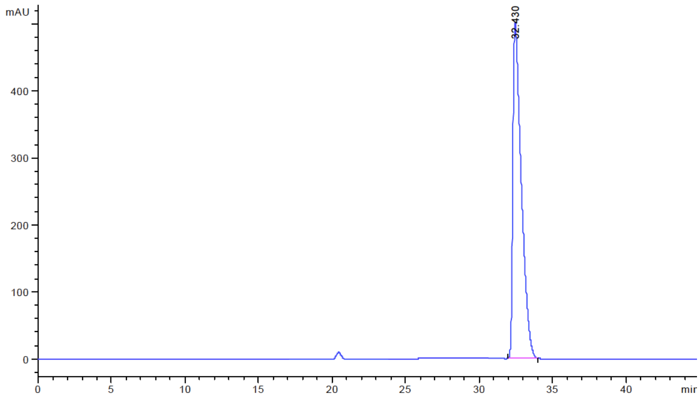

Syn-hydration product Chiralpak IC column, T = 20 °C, *n*-Hexane/*i*-PrOH = 90:10, 1 mL/min, λ = 254 nm, t_R = 37.175 min.

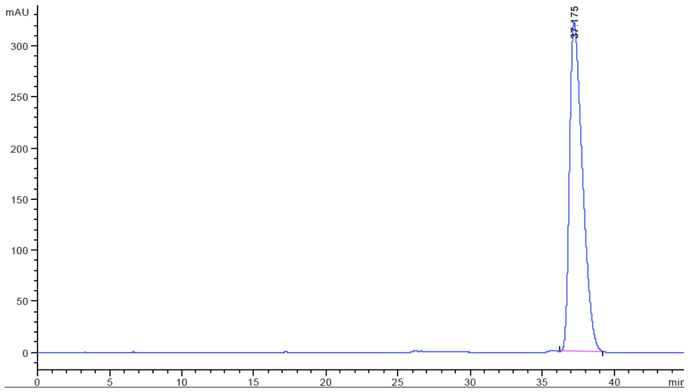

Following the general procedure N. HPLC analysis of the crude residue indicated a ratio **1a**/**4a** of 1:99 and an enantiomeric excess of (+) 97% [Chiralpak IC column, T = 20 °C, *n*-Hexane/*i*-PrOH = 90:10, 1 mL/min, λ = 254 nm, t_R = 19.891 min and t_R = 24.111 min.].

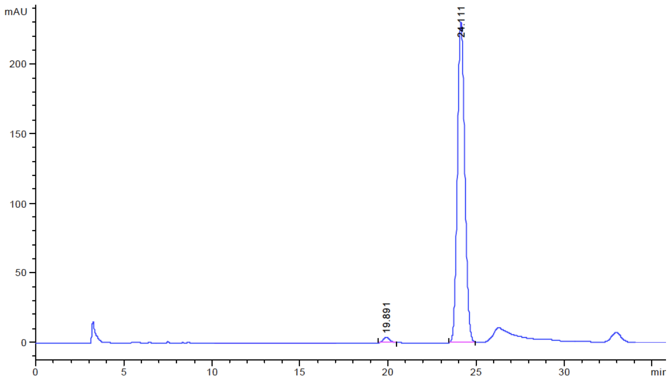

Peak	Retention Time	Rel. Area
	min	%
1	19,891	1,4971
2	24,111	98,5029

1-(1-Methyl-1*H*-imidazol-2-yl)-3-(nitromethyl)hexan-1-one (4b)

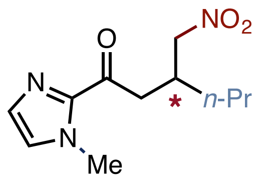

MW (g/mol): 239.28 **Molecular formula:** $C_{11}H_{17}N_3O_3$

Synthesised according racemic procedure using **1b**. The desired product was obtained as a brown oil (57.0 mg, 0.24 mmol, 72% yield) after purification by flash column chromatography over silica gel (Ethyl acetate/Petroleum ether, 50:50).

^{1}H NMR (400 MHz, $CDCl_3$) δ 7.11 (d, J = 1.0 Hz, 1H), 7.03 (d, J = 1.0 Hz, 1H), 4.50 (dd, J = 12.2, 6.2 Hz, 1H), 4.42 (dd, J =12.2, 6.7 Hz, 1H), 3.98 (s, 3H), 3.29 (dd, J =17.5, 5.6 Hz, 1H), 3.19 (dd, J =17.5, 7.8 Hz, 1H), 2.84 (m, 1H), 1.46-1.33 (m, 4H), 0.90 (t, J = 0.9 Hz, 3H).

^{13}C NMR (101 MHz, $CDCl_3$) δ 190.6, 142.8, 129.2, 127.3, 79.0, 40.3, 36.1, 34.0, 33.2, 19.6, 13.9.

HRMS (ESI): m/z calcd for $C_{11}H_{18}N_3O_3$, $[M+H]^+$: 240.1348, found: 240.1358.

IR (neat): 2961, 1674, 1547, 1408, 918, 778 cm^{-1}.

HPLC: Chiralpak IC column, T = 20 °C, *n*-Hexane/*i*-PrOH = 90:10, 1 mL/min, λ = 254 nm.

Entry	General procedure	Sequences	Conversion (%)[a]	ee (%)[a]
**1**	N	**DNA-SerC2/RNA-U**	97	+97
**2**	Q	st-DNA	99	+76

[a]Determined by HPLC. The sign before the ee's values is arbitrary evaluated.

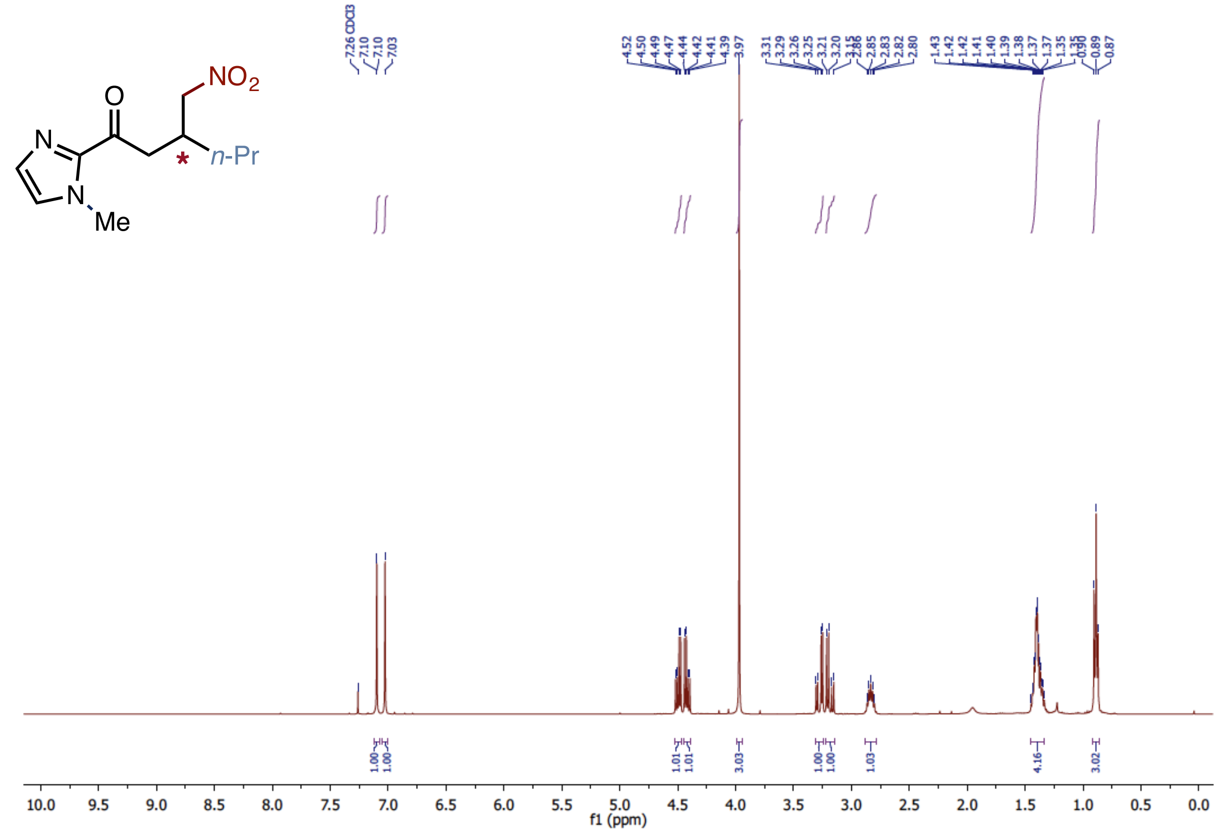
O
NO_2
N
N
Me
*
n-Pr
f1 (ppm)

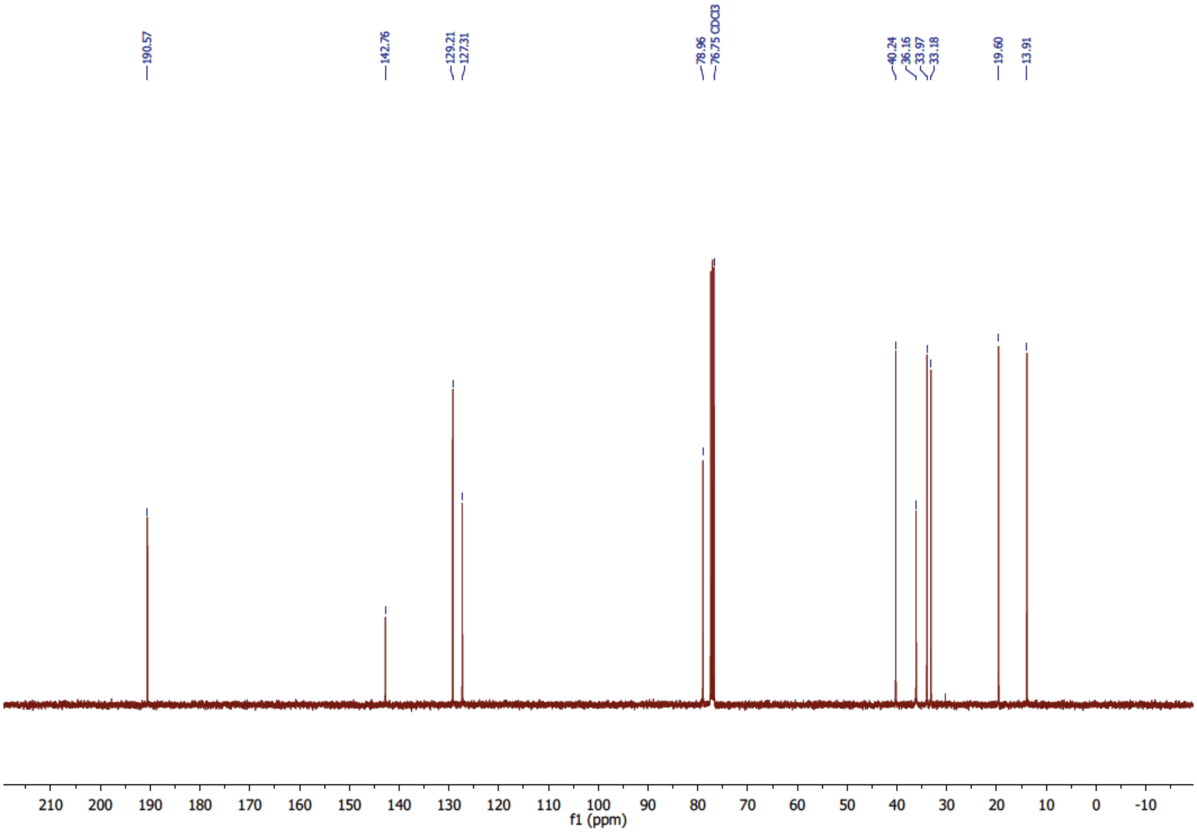
f1 (ppm)

Racemic Chiralpak IC column, T = 20 °C, *n*-Hexane/*i*-PrOH = 90:10, 1 mL/min, λ = 254 nm, t_R = 17.281 min and t_R = 22.258 min.

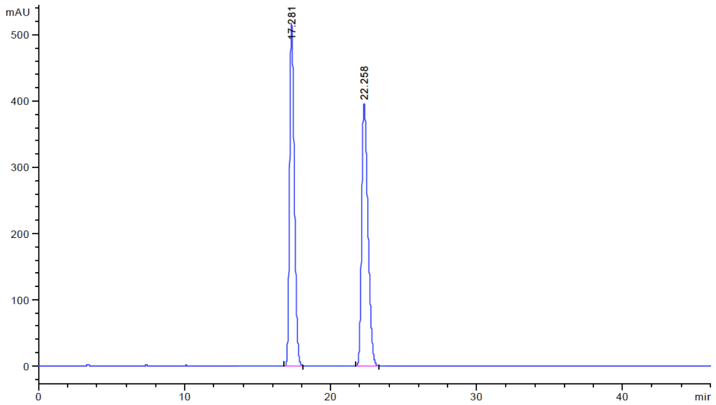

Peak	Retention Time	Rel. Area
	min	%
1	17,281	49,9246
2	22,258	50,0754

Starting material Chiralpak IC column, T = 20 °C, *n*-Hexane/*i*-PrOH = 90:10, 1 mL/min, λ = 254 nm, t_R = 35.647 min.

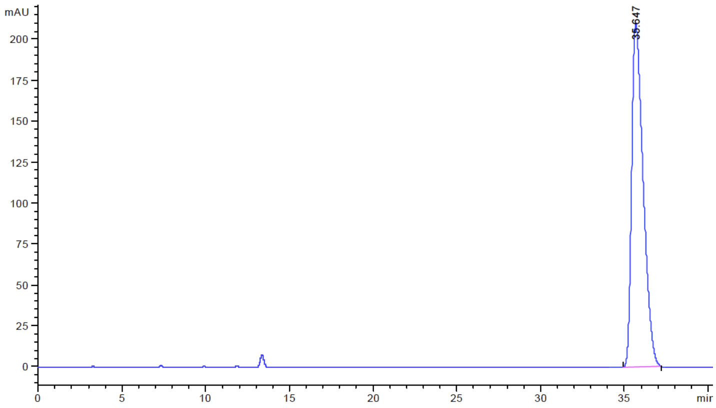

Following the general procedure N. HPLC analysis of the crude residue indicated a ratio **1b/4b** of 1:99 and an enantiomeric excess of (+) 97% [Chiralpak IC column, T = 20 °C, *n*-Hexane/*i*-PrOH = 90:10, 1 mL/min, λ = 254 nm, t_R = 18.013 min and t_R = 23.392 min.].

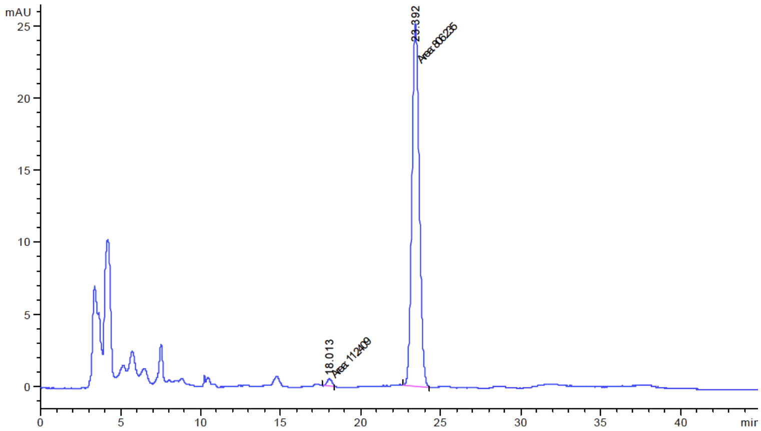

Peak	Retention Time	Rel. Area
	min	%
1	18,013	1,3751
2	23,392	98,6249

3-(4-Methoxyphenyl)-1-(1-methyl-1*H*-imidazol-2-yl)-4-nitrobutan-1-one (4c)

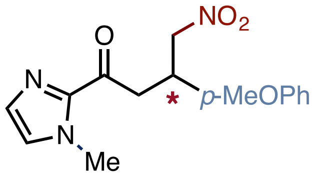

MW (g/mol): 303.32 **Molecular formula:** $C_{15}H_{17}N_3O_4$

Synthesised according racemic procedure using **1c**. The desired product was obtained as a brown solid (54.0 mg, 0.18 mmol, 54% yield) after purification by flash column chromatography over silica gel (Ethyl acetate/Petroleum ether, 50:50). The spectroscopic data of the product were identical with those reported in the literature.[14]

^{1}H NMR (400 MHz, CDCl$_3$) δ 7.23-7.19 (m, 2H), 7.13 (br s, 1H), 7.02 (br s, 1H), 6.85-6.81 (m, 2H), 4.70 (dd, *J* = 12.3, 6.7 Hz, 1H), 4.59 (dd, *J* = 12.3, 8.4 Hz, 1H), 4.15 (m, 1H), 3.94 (s, 3H), 3.76 (s, 3H), 3.71 (m, 1H), 3.48 (m, 1H).

^{13}C NMR (101 MHz, CDCl$_3$) δ 189.4, 159.1, 142.8, 131.0, 129.4, 128.7 (2C), 127.5, 114.4 (2C), 80.3, 55.4, 42.1, 38.8, 36.2.

HPLC: Chiralpak IA column, T = 20 °C, *n*-Hexane/*i*-PrOH = 80:20, 1 mL/min, λ = 254 nm.

Entry	General procedure	Sequences	Conversion (%)[a]	ee (%)[a]
**1**	N	**DNA-SerC2/RNA-U**	83	+97
**2**	Q	st-DNA	89	+82

[a]Determined by HPLC. The sign before the ee's values is arbitrary evaluated.

Racemic Chiralpak IA column, T = 20 °C, *n*-Hexane/*i*-PrOH = 80:20, 1 mL/min, λ = 254 nm, t_R = 16.777 min and t_R = 19.625 min.

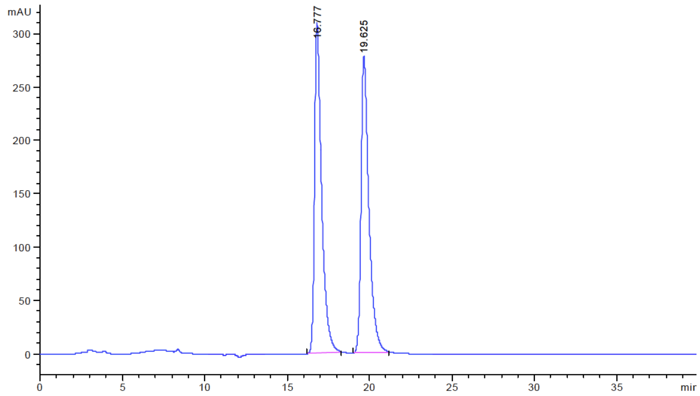

Peak	Retention Time	Rel. Area
	min	%
1	16,777	50,1227
2	19,625	49,8773

Starting material Chiralpak IA column, T = 20 °C, *n*-Hexane/*i*-PrOH = 80:20, 1 mL/min, λ = 254 nm, t_R = 13.224 min.

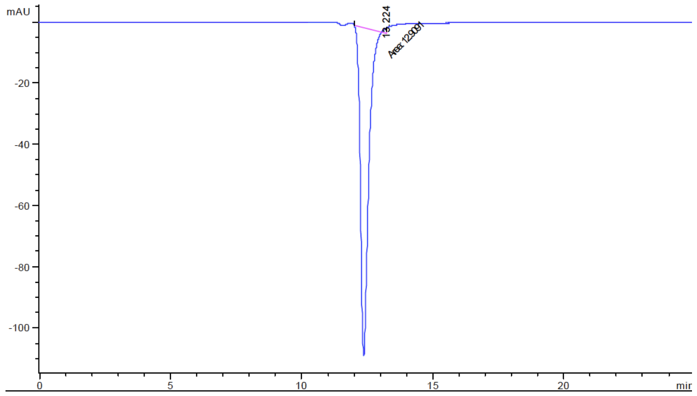

Following the general procedure N. HPLC analysis of the crude residue indicated a ratio **1c**/**4c** of 17:83 and an enantiomeric excess of (+) 97% [Chiralpak IA column, T = 20 °C, *n*-Hexane/*i*-PrOH = 80:20, 1 mL/min, λ = 254 nm, t_R = 15.795 min and t_R = 18.086 min.].

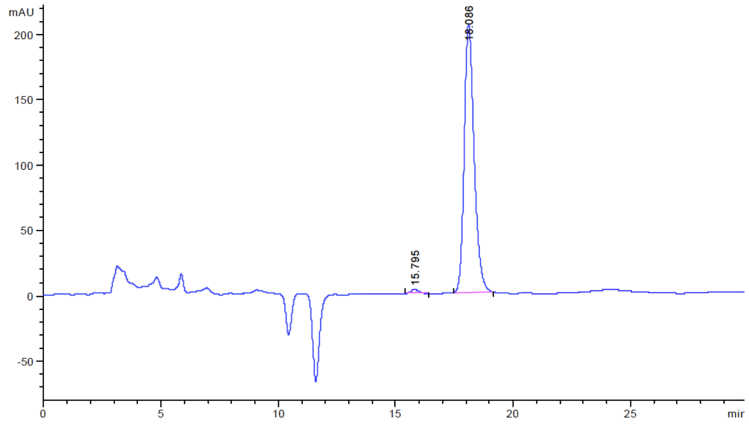

Peak	Retention Time	Rel. Area
	min	%
1	15,795	1,3125
2	18,086	98,6875

Dimethyl 2-(4-(1-methyl-1H-imidazol-2-yl)-4-oxobutan-2-yl)malonate (4d)

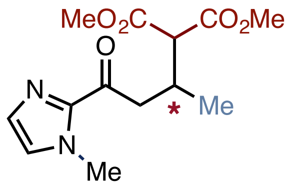

MW (g/mol): 282.30 **Molecular formula:** $C_{13}H_{18}N_2O_5$

Synthesised according racemic procedure using **1a**. The desired product was obtained as a brown solid (60.0 mg, 0.21 mmol, 64% yield) after purification by flash column chromatography over silica gel (Ethyl acetate/Petroleum ether, 50:50). The spectroscopic data of the product were identical with those reported in the literature.[14]

^{1}H NMR (400 MHz, $CDCl_3$) δ 7.05 (br s, 1H), 6.96 (br s, 1H), 3.93 (s, 3H), 3.66 (s, 3H), 3.65 (s, 3H), 3.45 (d, *J* = 7.4 Hz, 1H), 3.17 (d, *J* = 6.6 Hz, 2H), 2.89 (m, 1H), 1.01 (d, *J* = 6.8 Hz, 3H).

^{13}C NMR (101 MHz, $CDCl_3$) δ 191.1, 169.0, 168.9, 143.0, 129.0, 127.0, 56.2, 52.4, 52.3, 43.1, 36.2, 29.3, 17.7.

HPLC: Chiralpak IC column, T = 20 °C, *n*-Hexane/*i*-PrOH = 86:14, 0.8 mL/min, λ = 254 nm.

Entry	General procedure	Sequences	Conversion (%)[a]	ee (%)[a]
**1**	O	**DNA-SerC2/RNA-U**	44	+78
**2**	Q	st-DNA	36	+18

[a]Determined by HPLC. The sign before the ee's values is arbitrary evaluated.

Racemic Chiralpak IC column, T = 20 °C, *n*-Hexane/*i*-PrOH = 86:14, 0.8 mL/min, λ = 254 nm, t_R = 50.261 min and t_R = 65.954 min.

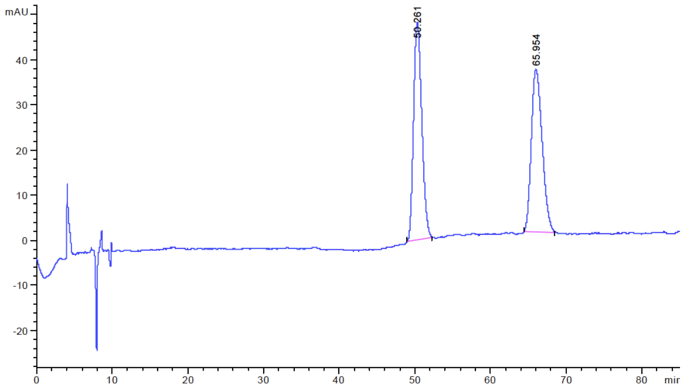

Peak	Retention Time	Rel. Area
	min	%
1	50,261	50,3518
2	65,954	49,6482

Starting material 1a Chiralpak IC column, T = 20 °C, *n*-Hexane/*i*-PrOH = 86:14, 0.8 mL/min, λ = 254 nm, t_R = 22.810 min.

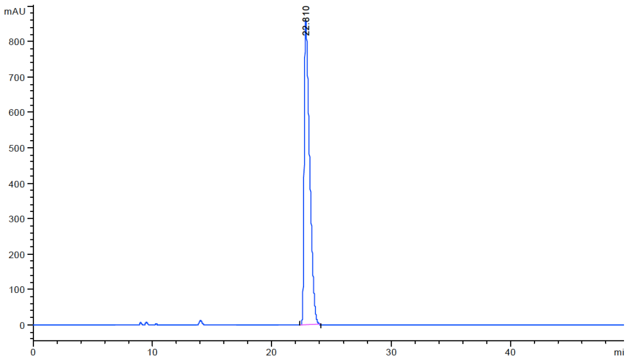

Syn-hydration product Chiralpak IC column, T = 20 °C, *n*-Hexane/*i*-PrOH = 86:14, 0.8 mL/min, λ = 254 nm, t_R = 33.029 min.

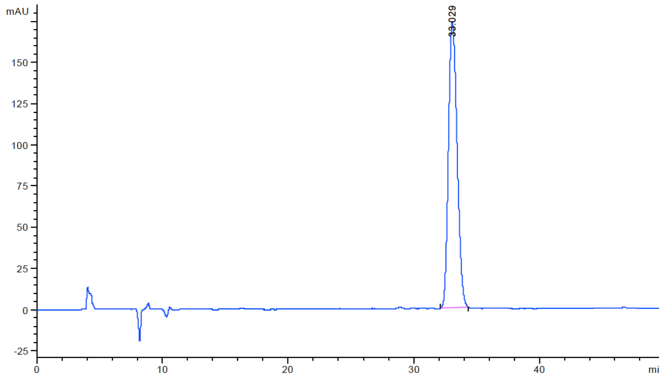

Following the general procedure O. HPLC analysis of the crude residue indicated a ratio **1a**/syn-hydration/**4d** of 11:46:44 and an enantiomeric excess of (+) 78% [Chiralpak IC column, T = 20 °C, *n*-Hexane/*i*-PrOH = 86:14, 0.8 mL/min, λ = 254 nm, t_R = 57.038 min and t_R = 73.671 min.].

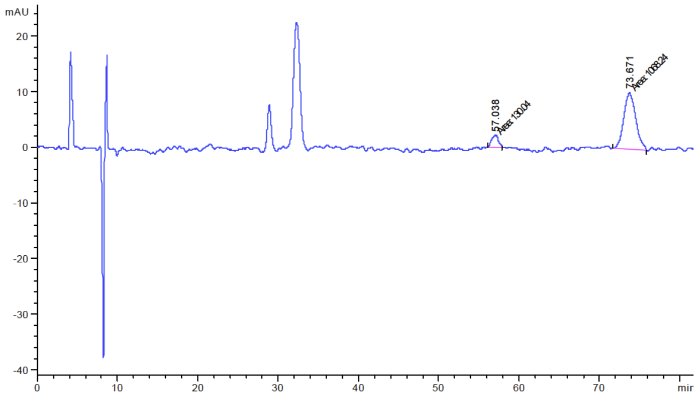

Peak	Retention Time	Rel. Area
	min	%
1	57,038	10,8522
2	73,671	89,1478

Following the general procedure Q. HPLC analysis of the crude residue indicated a ratio **1a**/syn-hydration/**4d** of 4:60:36 and an enantiomeric excess of (+) 18% [Chiralpak IC column, T = 20 °C, *n*-Hexane/*i*-PrOH = 86:14, 0.8 mL/min, λ = 254 nm, t_R = 63.335 min and t_R = 84.864 min.].

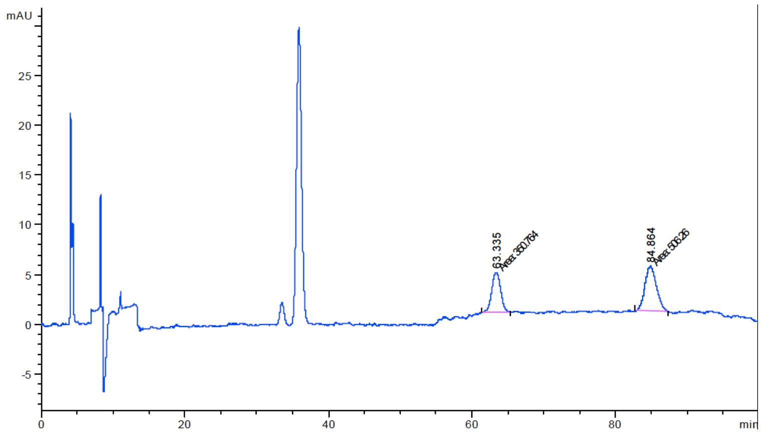

Peak	Retention Time	Rel. Area
	min	%
1	63,335	40,9241
2	84,864	49,0719

2-(4-(1-Methyl-1*H*-imidazol-2-yl)-4-oxobutan-2-yl)malononitrile (4e)

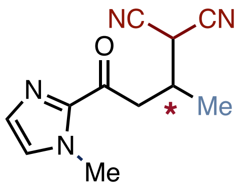

MW (g/mol): 216.24 **Molecular formula:** $C_{11}H_{12}N_4O$

Synthesised according racemic procedure using **1a**. The desired product was obtained as a colourless oil (63.0 mg, 0.29 mmol, 88% yield) after purification by flash column chromatography over silica gel (Ethyl acetate/Petroleum ether, 50:50). The spectroscopic data of the product were identical with those reported in the literature.[15]

^{1}H NMR (400 MHz, CDCl$_3$) δ 7.14 (br s, 1H), 7.08 (br s, 1H), 4.36 (d, *J* = 4.8 Hz, 1H), 3.98 (s, 3H), 3.45 (dd, *J* = 18.0, 5.2 Hz, 1H), 3.27 (dd, *J* = 18.0, 8.2 Hz, 1H), 2.79 (m, 1H), 1.35 (d, *J* = 6.8 Hz, 3H).

^{13}C NMR (101 MHz, CDCl$_3$) δ 189.4, 149.3, 129.7, 127.8, 112.5, 111.5, 41.5, 36.2, 31.8, 28.12, 17.7.

HPLC: Chiralpak IC column, T = 20 °C, *n*-Hexane/*i*-PrOH = 85:15, 1 mL/min, λ = 254 nm.

Entry	General procedure	Sequences	Conversion (%)[a]	ee (%)[a]
**1**	N	**DNA-SerC2/RNA-U**	93	+72
**2**	Q	st-DNA	>99	+36

[a]Determined by HPLC. The sign before the ee's values is arbitrary evaluated.

Racemic Chiralpak IC column, T = 20 °C, *n*-Hexane/*i*-PrOH = 85:15, 1 mL/min, λ = 254 nm, t_R = 16.498 min and t_R = 19.026 min.

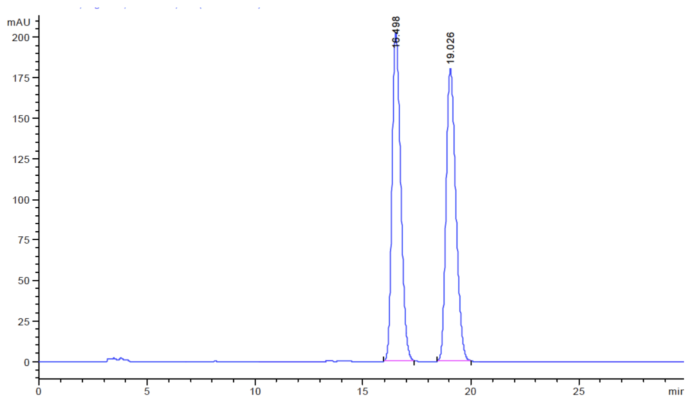

Peak	Retention Time	Rel. Area
	min	%
1	16,498	50,0024
2	19,026	49,9976

Starting material 1a Chiralpak IC column, T = 20 °C, *n*-Hexane/*i*-PrOH = 85:15, 1 mL/min, λ = 254 nm, t_R = 24.240 min.

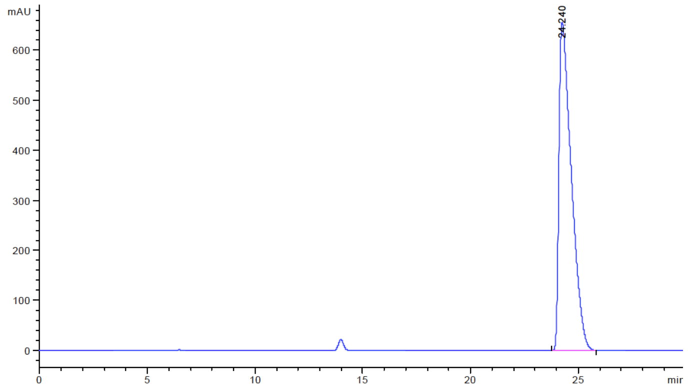

Following the general procedure N. HPLC analysis of the crude residue indicated a ratio **1a/4e** of 7:93 and an enantiomeric excess of (+) 72% [Chiralpak IC column, T = 20 °C, *n*-Hexane/*i*-PrOH = 85:15, 1 mL/min, λ = 254 nm, t_R = 15.518 min and t_R = 17.282 min.].

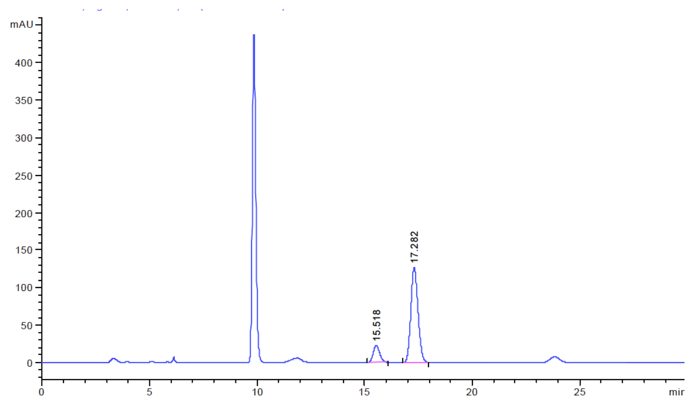

Peak	Retention Time	Rel. Area
	min	%
1	15,518	14,0512
2	17,282	85,9488

2-(1-(4-Methoxyphenyl)-3-(1-methyl-1*H*-imidazol-2-yl)-3-oxopropyl)malononitrile (4f)

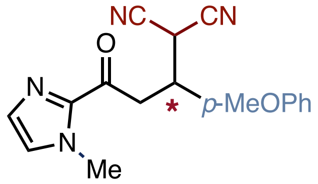

MW (g/mol): 308.34 **Molecular formula:** $C_{17}H_{16}N_4O_2$

Synthesised according racemic procedure using **1c**. The desired product was obtained as a colourless oil (80.0 mg, 0.26 mmol, 79% yield) after purification by flash column chromatography over silica gel (Ethyl acetate/Petroleum ether, 1:2). The spectroscopic data of the product were identical with those reported in the literature.[15]

^{1}H NMR (400 MHz, $CDCl_3$) δ 7.35-7.26 (m, 2H), 7.14 (d, *J* = 0.9 Hz, 1H), 7.04 (d, *J* = 0.9 Hz, 1H), 6.90-6.88 (m, 2H), 4.43 (m, 1H), 3.95-3.84 (m, 2H), 3.93 (s, 3H), 3.77 (s, 3H), 3.71 (m, 1H).

^{13}C NMR (101 MHz, $CDCl_3$) δ 188.7, 159.6, 142.2, 129.7 (2C), 129.3, 128.3, 127.8, 114.5 (2C), 112.1, 111.8, 55.3, 40.8, 40.6, 36.2, 29.6.

HPLC: Chiralpak IA column, T = 20 °C, *n*-Hexane/*i*-PrOH = 80:20, 1 mL/min, λ = 254 nm.

Entry	General procedure	Sequences	Conversion (%)[a]	ee (%)[a]
**1**	N	**DNA-SerC2/RNA-U**	95	+71
**2**	Q	st-DNA	>99	+64

[a]Determined by HPLC. The sign before the ee's values is arbitrary evaluated.

Racemic Chiralpak IA column, T = 20 °C, *n*-Hexane/*i*-PrOH = 80:20, 1 mL/min, λ = 254 nm, t_R = 20.973 min and t_R = 24.046 min.

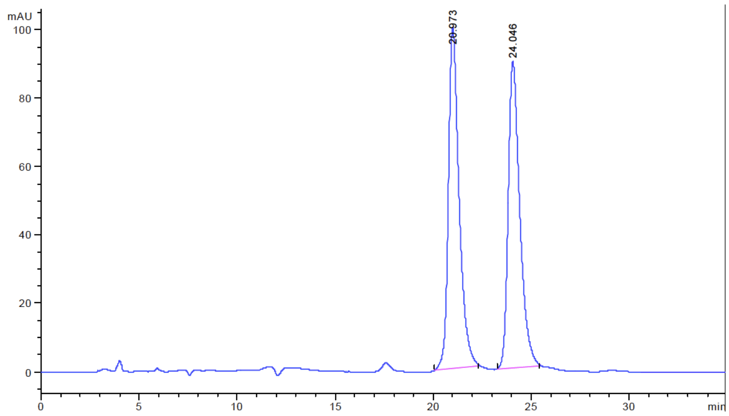

Peak	Retention Time	Rel. Area
	min	%
1	20,973	50,7301
2	24,046	49,2699

Starting material 1c Chiralpak IA column, T = 20 °C, *n*-Hexane/*i*-PrOH = 80:20, 1 mL/min, λ = 254 nm, t_R = 11.526 min.

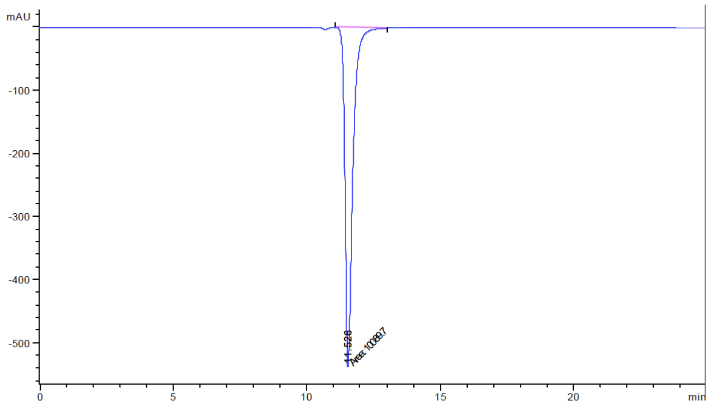

Following the general procedure N. HPLC analysis of the crude residue indicated a ratio **1c**/**4f** of 5:95 and an enantiomeric excess of (+) 71% [Chiralpak IA column, T = 20 °C, *n*-Hexane/*i*-PrOH = 80:20, 1 mL/min, λ = 254 nm, t_R = 20.879 min and t_R = 23.898 min.].

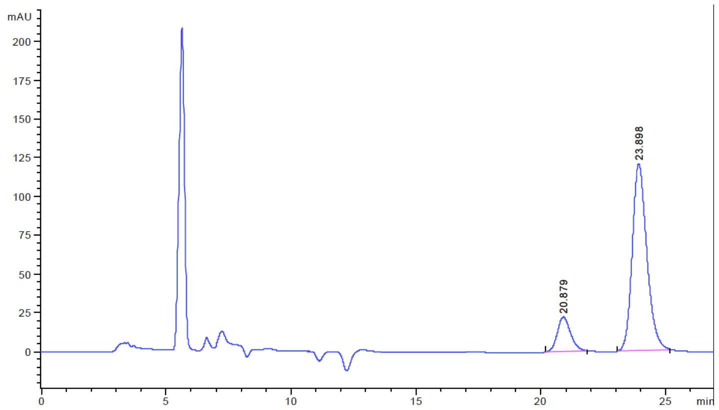

Peak	Retention Time	Rel. Area
	min	%
1	20,879	14,6929
2	23,898	85,3071

***tert*-Butyl 2-cyano-3-(4-methoxyphenyl)-5-(1-methyl-1*H*-imidazol-2-yl)-5-oxo pentanoate (4g)**

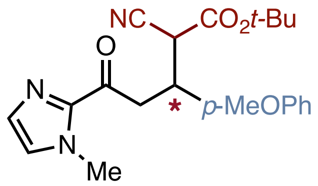

MW (g/mol): 383.45 **Molecular formula:** $C_{21}H_{25}N_3O_4$

Synthesised according racemic procedure using **1c**. The major diastereoisomer was obtained as a brown oil (101 mg, 0.26 mmol, 80% yield) after purification by flash column chromatography over silica gel (Ethyl acetate/Petroleum ether, 30:70).

^{1}H NMR (400 MHz, $CDCl_3$) δ 7.33-7.29 (m, 2H), 7.10 (d, *J* = 0.9 Hz, 1H), 7.00 (br s, 1H), 6.82-6.78 (m, 2H), 4.08 (d, *J* = 5.9 Hz, 1H), 4.00 (m, 1H), 3.92 (s, 3H), 3.79 (dd, *J* = 18.5, 6.3 Hz, 1H), 3.75 (s, 3H), 3.67 (dd, *J* = 18.5, 7.7 Hz, 1H), 1.31 (s, 9H).

^{13}C NMR (101 MHz, $CDCl_3$) δ 189.6, 164.0, 159.2, 142.5, 130.4, 129.4 (2C), 129.2, 127.3, 115.9 (2C), 113.9, 84.0, 55.2, 44.7, 42.3, 39.7, 36.1, 27.7 (3C).

HRMS (ESI): m/z calcd for $C_{21}H_{26}N_3O_4$, $[M+H]^+$: 384.1923, found: 384.1939.

IR (neat): 2980, 1737, 1676, 1516, 1253, 1154, 1035, 838 cm^{-1}.

HPLC: Chiralpak IA column, T = 20 °C, *n*-Hexane/*i*-PrOH = 88:12, 1 mL/min, λ = 254 nm.

Entry	General procedure	Sequences	Conversion (%)[a]	ee (%)[a]
**1**	N	**DNA-SerC2/RNA-U**	50<br>(dr = 2:3)	+79 (minor)<br>+74 (major)
**2**	Q	st-DNA	64<br>(dr = 2:3)	+49 (minor)<br>+50 (major)

[a]Determined by HPLC. The sign before the ee's values is arbitrary evaluated.

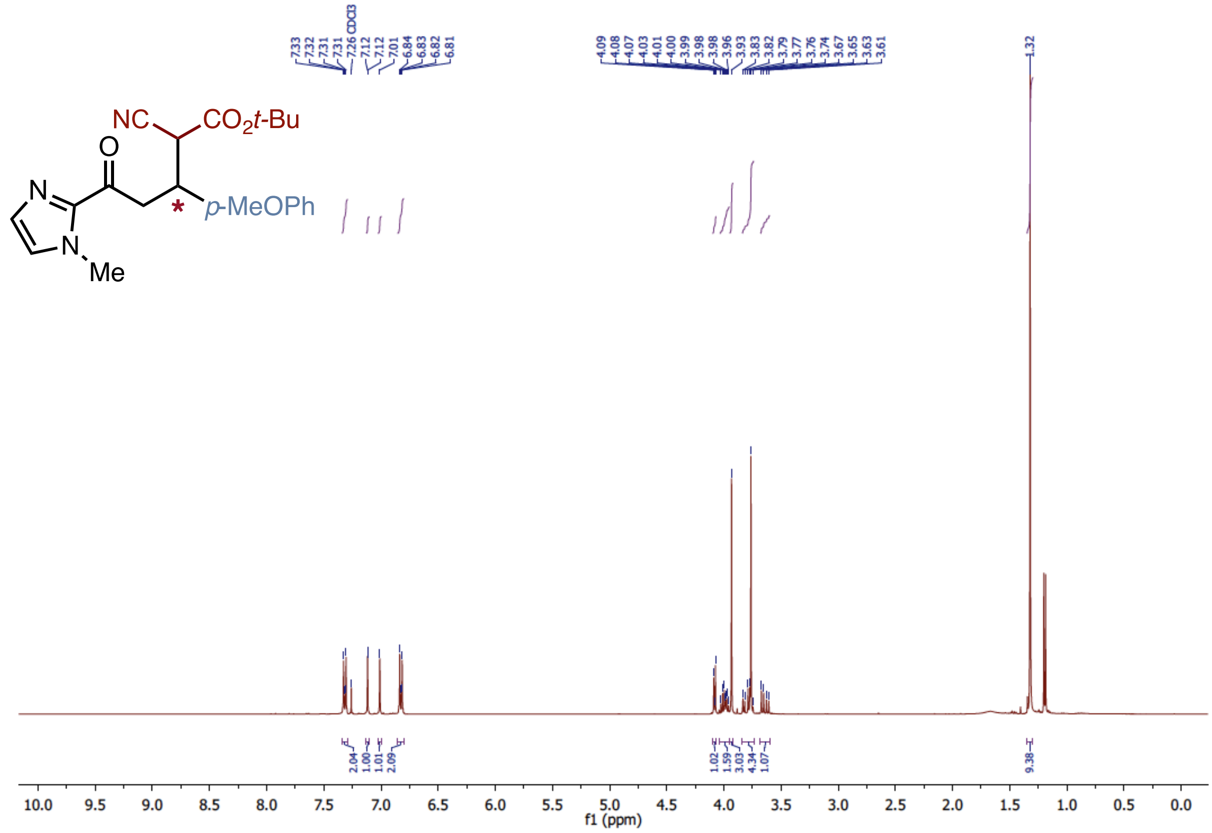
NC
CO2t-Bu
O
N
N
Me
p-MeOPh
7.33 7.32 7.31 7.31 7.26 CDCl3 7.12 7.12 7.01 6.84 6.83 6.82 6.81
4.09 4.08 4.07 4.03 4.01 4.00 3.99 3.98 3.98 3.96 3.93 3.83 3.82 3.79 3.77 3.76 3.74 3.67 3.65 3.63 3.61
1.32
2.04 1.00 1.01 2.09
1.02 1.59 3.03 4.34 1.07
9.38
f1 (ppm)

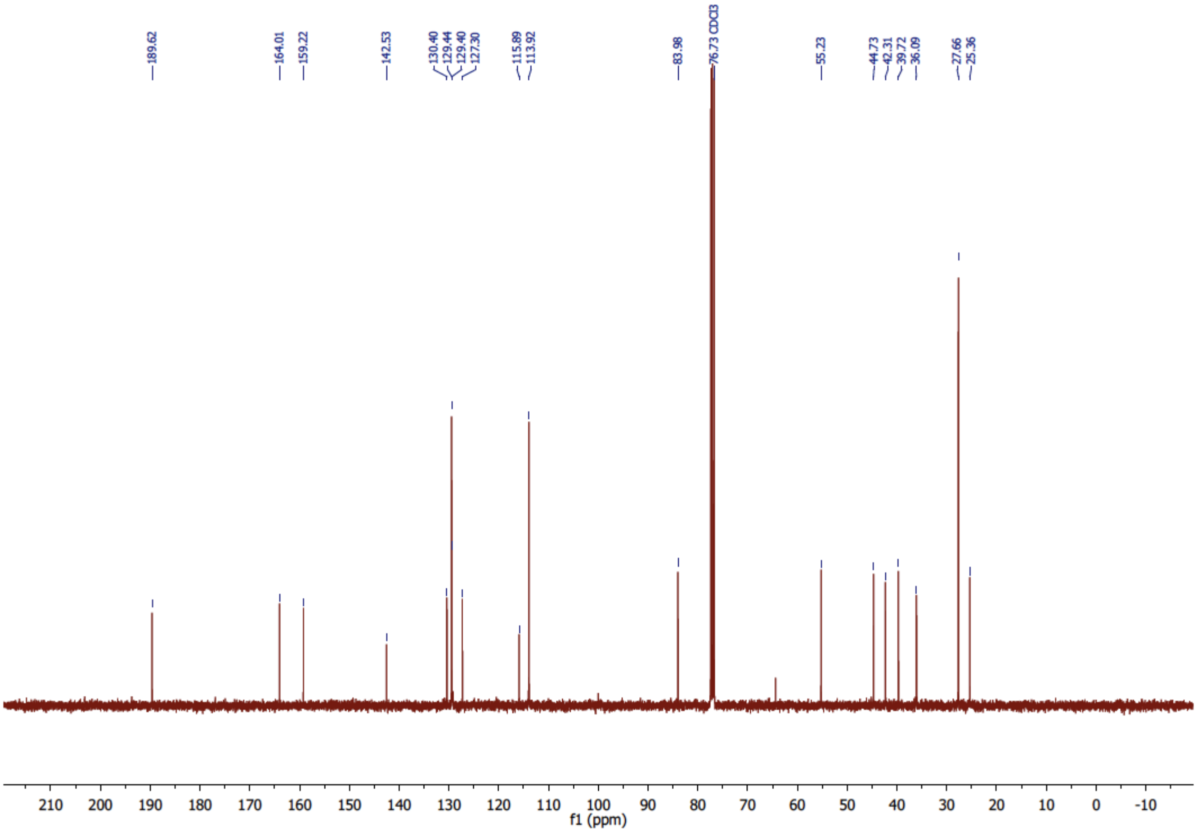
189.62
164.01
159.22
142.53
130.40 129.44 129.40 127.30
115.89 113.92
83.98
76.73 CDCl3
55.23
44.73 42.31 39.72 36.09
27.66 25.36
f1 (ppm)

Racemic Chiralpak IA column, T = 20 °C, *n*-Hexane/*i*-PrOH = 88:12, 1 mL/min, λ = 254 nm, t_R = 26.591 min (diastereoisomer 1), t_R = 28.414 min (diastereoisomer 1), t_R = 42.098 min (diastereoisomer 2) and t_R = 45.120 min (diastereoisomer 2).

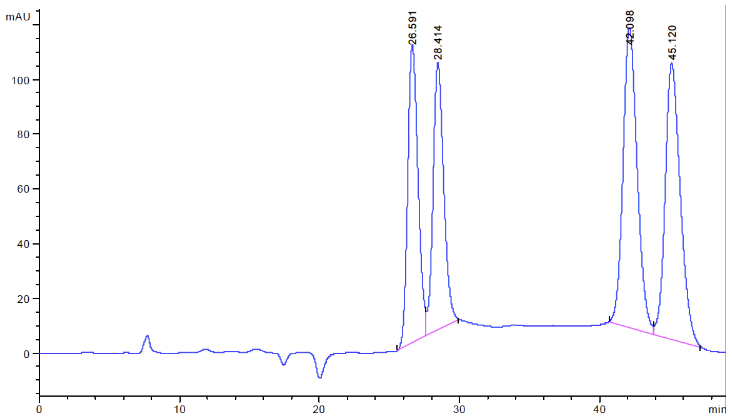

Peak	Retention Time	Rel. Area
	min	%
1	26,591	21,3663
2	28,414	19,8884
3	42,098	29,604
4	45,12	29,0513

Starting material 1c Chiralpak IA column, T = 20 °C, *n*-Hexane/*i*-PrOH = 88:12, 1 mL/min, λ = 254 nm, t_R = 19.892 min.

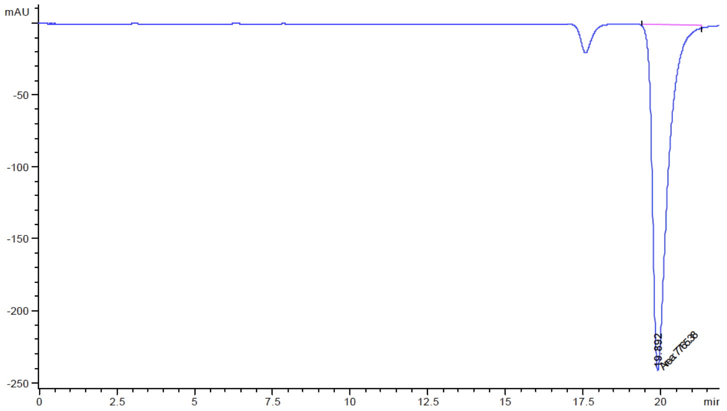

Following the general procedure N. HPLC analysis of the crude residue indicated a ratio **1c**/**4g** of 50:50, a diastereomeric ration of 3:2 an enantiomeric excess of (+) 74% (major diastereoisomer) and (+) 79% (minor diastereoisomer) [Chiralpak IA column, T = 20 °C, *n*-Hexane/*i*-PrOH = 88:12, 1 mL/min, λ = 254 nm, t_R = 27.083 min, t_R = 28.881 min, t_R = 42.917 min, and t_R = 45.890 min.].

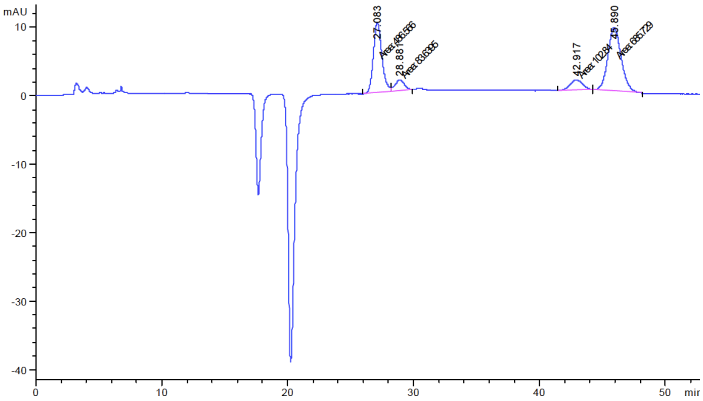

Peak	Retention Time	Rel. Area
	min	%
1	27,082	35,8092
2	28,881	6,1555
3	42,917	7,5686
4	45,89	50,4667

(1-Methyl-1*H*-imidazol-2-yl)(3-methylbicyclo[2.2.1]hept-5-en-2-yl)methanone (5a)

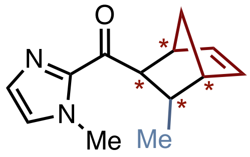

MW (g/mol): 216.28 **Molecular formula:** $C_{13}H_{16}N_2O$

Synthesised according racemic procedure using **1a**. The major *endo* product was obtained as a colourless oil (80.0 mg, 0.37 mmol, 92% yield, *endo*/*exo* 14:1) after purification by flash column chromatography over silica gel (Petroleum ether/Ethyl acetate, 70:30). The spectroscopic data of the product were identical with those reported in the literature.[16]

^{1}H NMR (400 MHz, $CDCl_3$) δ 7.12 (br s, 1H), 6.98 (br s, 1H), 6.32 (dd, *J* = 5.6, 3.5 Hz, 1H), 5.78 (dd, *J* = 5.6, 2.8 Hz, 1H), 3.92 (s, 3H), 3.71 (dd, *J* = 4.5, 3.5 Hz, 1H), 3.34 (br s, 1H), 2.51 (m, 1H), 2.01 (m, 1H), 1.76 (d, *J* = 8.5 Hz, 1H), 1.44 (dd, *J* = 8.5, 1.7 Hz, 1H), 1.15 (d, *J* = 7.0 Hz, 3H).

^{13}C NMR (101 MHz, $CDCl_3$) δ 193.6, 143.5, 138.9, 132.0, 128.9, 126.8, 56.1, 49.9, 49.2, 47.3, 36.4, 36.1, 20.7.

HPLC: Chiralpak IC column, T = 20 °C, *n*-Hexane/*i*-PrOH = 90:10, 1 mL/min, λ = 254 nm. The minor *exo* compound could not be separated.

Entry	General procedure	Sequences	Conversion (%)[a]	ee (%)[a]
**1**	N	**DNA-SerC2/RNA-U**	65<br>*endo*/*exo*<br>92:8	+73 (*endo*)
**2**	P	**DNA-SerC2/RNA-C3**	77<br>*endo*/*exo*<br>91:9	+76 (*endo*)
**2**	Q	st-DNA	*endo*/*exo*<br>>99 :1	+86 (*endo*)

[a]Determined by HPLC. The sign before the ee's values is arbitrary evaluated.

Racemic Chiralpak IC column, T = 20 °C, *n*-Hexane/*i*-PrOH = 90:10, 1 mL/min, λ = 254 nm, t_R = 7.187 min (major *endo*, major enantiomer) and t_R = 7.922 min (major *endo*, minor enantiomer). The minor *exo* compound could not be separated.

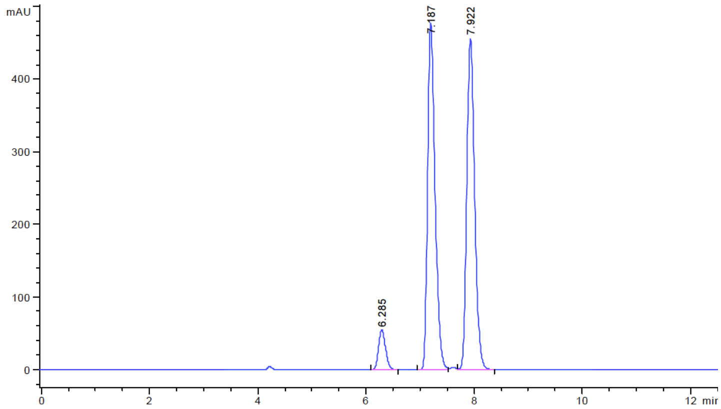

Peak	Retention Time	Rel. Area
	min	%
1	6,285	5,253
2	7,187	47,3617
3	7,922	47,3852

Starting material 1a Chiralpak IC column, T = 20 °C, *n*-Hexane/*i*-PrOH = 90:10, 1 mL/min, λ = 254 nm, t_R = 25.693 min.

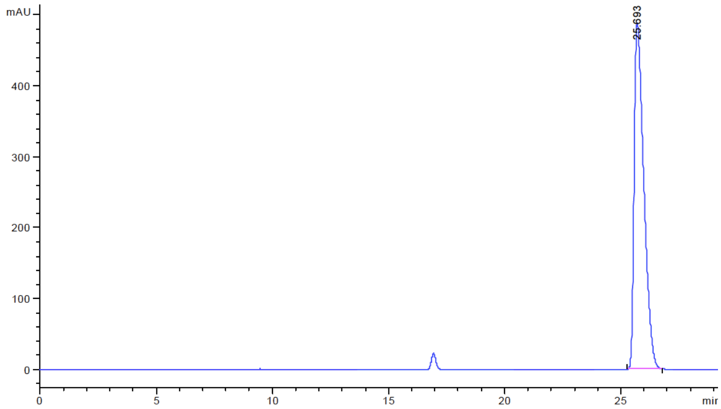

Following the general procedure N. HPLC analysis of the crude residue indicated a ratio **1a/5a** of 35:65 and an enantiomeric excess of (+) 73% (major diastereoisomer) [Chiralpak IC column, T = 20 °C, *n*-Hexane/*i*-PrOH = 90:10, 1 mL/min, λ = 254 nm, t_R = 7.107 min, t_R = 7.633 min].

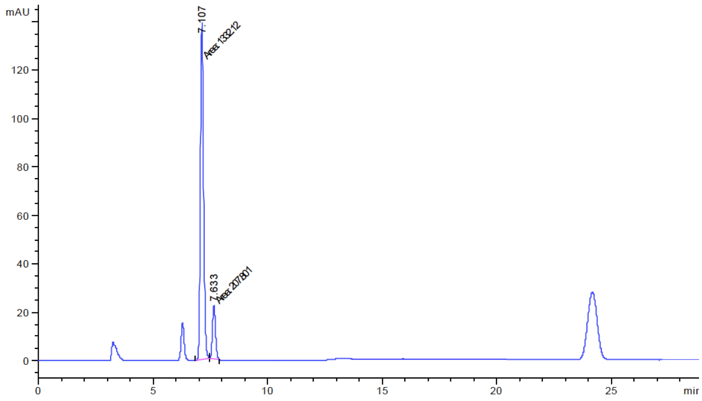

Peak	Retention Time	Rel. Area
	min	%
1	7,107	86,5058
2	7,633	13,4942

Following the general procedure P HPLC analysis of the crude residue indicated a ratio **1a/5a** of 23:77 and an enantiomeric excess of (+) 76% (major diastereoisomer) [Chiralpak IC column, T = 20 °C, *n*-Hexane/*i*-PrOH = 90:10, 1 mL/min, λ = 254 nm, t_R = 7.112 min, t_R = 7.684 min].

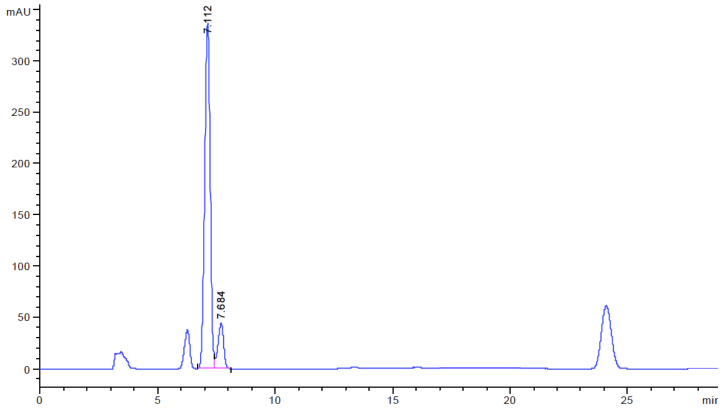

Peak	Retention Time	Rel. Area
	min	%
1	7,112	88,0599
2	7,684	11,9401

(3-(4-Methoxyphenyl)bicyclo[2.2.1]hept-5-en-2-yl)(1-methyl-1*H*-imidazol-2-yl)methanone (5b)

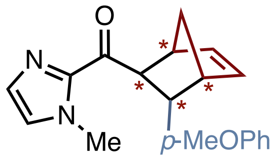

MW (g/mol): 308.38 **Molecular formula:** $C_{19}H_{20}N_2O_2$

Synthesised according racemic procedure using **1c**. The major *endo* product was obtained as a colourless oil (116 mg, 0.38 mmol, 94% yield, *endo*/*exo* 15:1) after purification by flash column chromatography over silica gel (Petroleum ether/Ethyl acetate, 30:70). The spectroscopic data of the product were identical with those reported in the literature.[16]

^{1}H NMR (400 MHz, $CDCl_3$) δ 7.21 (d, *J* = 8.6 Hz, 2H), 7.13 (br s, 1H), 6.98 (br s, 1H), 6.80 (d, *J* = 8.6 Hz, 2H), 6.49 (dd, *J* = 5.5, 3.2 Hz, 1H), 5.87 (dd, *J* = 5.5, 2.7 Hz, 1H), 4.33 (dd, *J* = 5.1, 3.5 Hz, 1H), 3.94 (s, 3H), 3.75 (s, 3H), 3.58 (s, 1H), 3.29 (d, *J* = 4.7 Hz, 1H), 2.97 (br s, 1H), 2.01 (d, *J* = 8.5 Hz, 1H), 1.57 (m, 1H).

^{13}C NMR (101 MHz, $CDCl_3$) δ 192.6, 157.9, 143.2, 139.5, 136.4, 132.7, 129.0, 128.6 (2C), 126.8, 113.9 (2C), 55.3, 55.2, 50.1, 49.5, 48.3, 45.1, 36.3.

HPLC: Chiralpak IC column, T = 20 °C, *n*-Hexane/*i*-PrOH = 90:10, 1 mL/min, λ = 254 nm.

Entry	General procedure	Sequences	Conversion (%)[a]	ee (%)[a]
**1**	N	**DNA-SerC2/RNA-U**	21<br>*endo*/*exo* 85:15	+51 (*endo*)
**2**	P	**DNA-SerC2/RNA-C3**	15<br>*endo*/*exo* 85:15	+56 (*endo*)
**3**	Q	st-DNA	*endo*/*exo* 99:1	+98 (*endo*)

[a]Determined by HPLC. The sign before the ee's values is arbitrary evaluated.

Racemic Chiralpak IC column, T = 20 °C, *n*-Hexane/*i*-PrOH = 90:10, 1 mL/min, λ = 254 nm, t_R = 11.375 min (minor *exo*), t_R = 12.948 min (major *endo*), t_R = 17.337 min (minor *exo*) and t_R = 27.555 min (major *endo*).

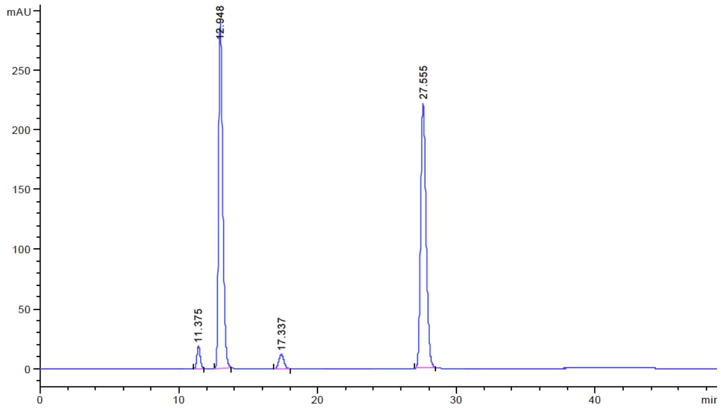

Peak	Retention Time	Rel. Area
	min	%
1	11,375	2,5375
2	12,948	47,4084
3	17,337	2,5451
4	27,555	47,509

Starting material 1c Chiralpak IC column, T = 20 °C, *n*-Hexane/*i*-PrOH = 90:10, 1 mL/min, λ = 254 nm, t_R = 30.438 min.

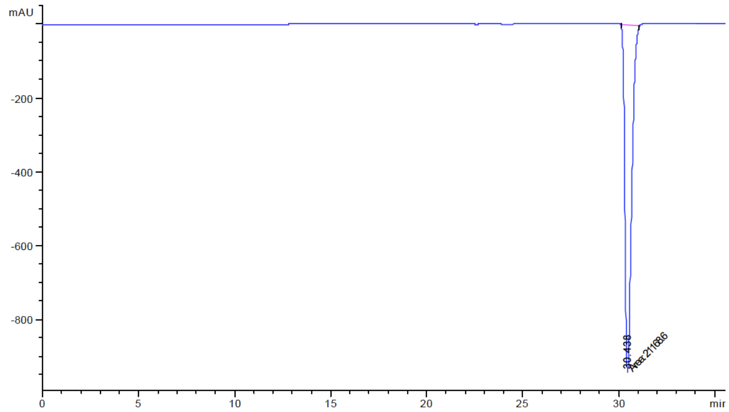

Following the general procedure N. HPLC analysis of the crude residue indicated a ratio **1c**/**5b** of 79:21 and an enantiomeric excess of (+) 51% (major diastereoisomer) [Chiralpak IC column, T = 20 °C, *n*-Hexane/*i*-PrOH = 90:10, 1 mL/min, λ = 254 nm, t_R = 13.045 min, t_R = 27.685 min].

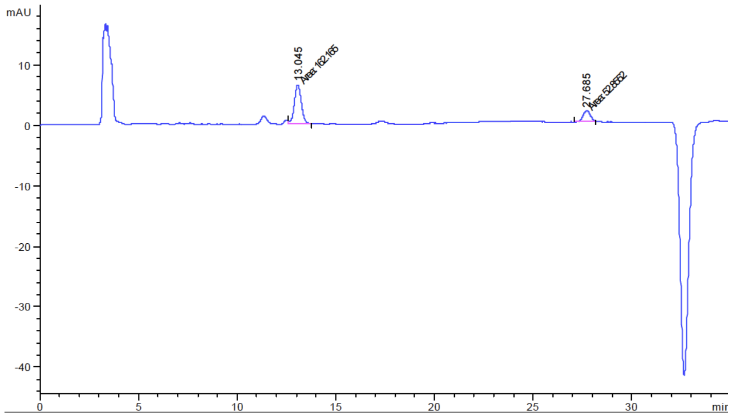

Peak	Retention Time	Rel. Area
	min	%
1	13,045	75,4185
2	27,685	24,5815

Following the general procedure P HPLC analysis of the crude residue indicated a ratio **1b**/**5b** of 85:15 and an enantiomeric excess of (+) 56% (major diastereoisomer) [Chiralpak IC column, T = 20 °C, *n*-Hexane/*i*-PrOH = 90:10, 1 mL/min, λ = 254 nm, t_R = 13.003 min, t_R = 27.630 min].

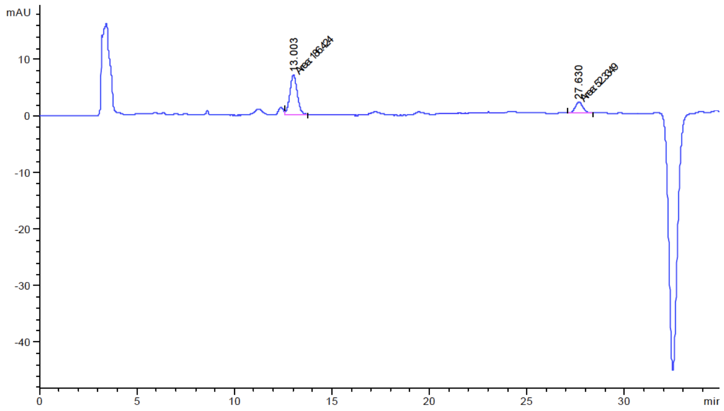

Peak	Retention Time	Rel. Area
	min	%
1	13,003	78,0804
2	27,63	21,9196

2-Methyl-3-(2-methyl-1*H*-indol-3-yl)-1-(thiazol-2-yl)propan-1-one (6a)

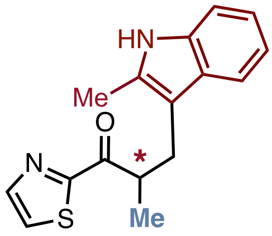

MW (g/mol): 284.38 **Molecular formula:** $C_{16}H_{16}N_2OS$

Synthesised according racemic procedure using **1e**. Isolated as yellow oil. The spectroscopic data of the product were identical with those reported in the literature.[11]

^{1}H NMR (400 MHz, $CDCl_3$) δ 8.00 (d, *J* = 3.0 Hz, 1H), 7.83 (br s, 1H, -*NH*), 7.68 (m, 1H), 7.62 (d, *J* = 3.0 Hz, 1H), 7.23 (m, 1H), 7.17-7.02 (m, 2H), 4.23 (m, 1H), 3.31 (dd, *J* = 14.2, 5.7 Hz, 1H), 2.81 (dd, *J* = 14.2, 8.9 Hz, 1H), 2.39 (s, 3H), 1.25 (d, *J* = 6.9 Hz, 3H).

^{13}C NMR (101 MHz, $CDCl_3$) δ 197.7, 167.0, 144.8, 135.3, 132.2, 128.9, 126.2, 121.0, 119.3, 118.5, 110.2, 109.4, 42.9, 28.2, 16.4, 11.9.

HPLC: Chiralpak IA column, T = 30 °C, *n*-Hexane/*i*-PrOH = 97:3, 1 mL/min, λ = 280 nm.

Entry	General procedure	Sequences	Conversion (%)[a]	ee (%)[a]
**1**	P	**DNA-SerC2/RNA-U**	94	+48
**2**	S	st-DNA	95	+6

[a]Determined by HPLC. The sign before the ee's values is arbitrary evaluated.

Racemic [Chiralpak IA column, T = 30 °C, *n*-Hexane/*i*-PrOH = 97:3, 1 mL/min, λ = 280 nm, t_R = 30.532 min and t_R = 35.839 min].

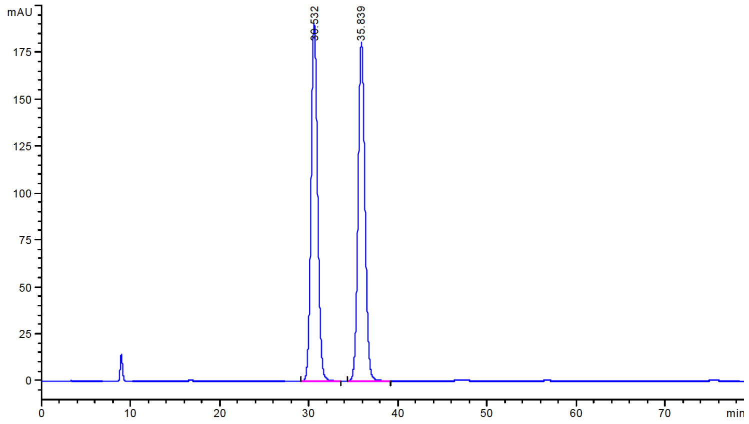

Peak	Retention Time	Rel. Area
	min	%
1	30.532	50.0069
2	35.839	49.9931

Indole [Chiralpak IA column, T = 30 °C, *n*-Hexane/*i*-PrOH = 97:3, 1 mL/min, λ = 280 nm, t_R = 11.919 min].

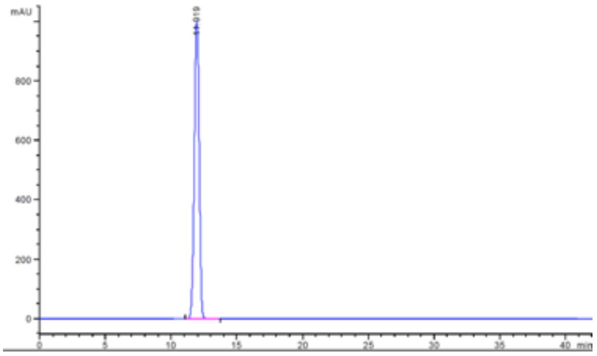

Following the general procedure P. HPLC analysis of the crude residue indicated a ratio indole/**6a** of 6:94 and an enantiomeric excess of (+) 48% [Chiralpak IA column, T = 30 °C, *n*-Hexane/*i*-PrOH = 97:3, 1 mL/min, λ = 280 nm, t_R = 30.079 min and t_R = 35.264 min].

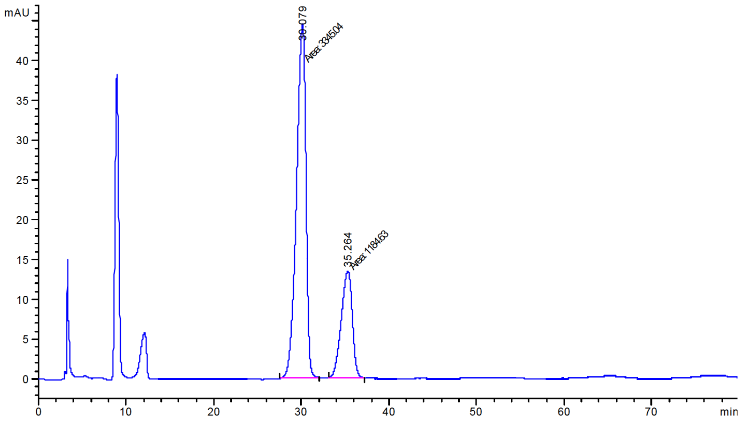

Peak	Retention Time	Rel. Area
	min	%
1	30.079	73.8474
2	35.264	26.1526

Following the general procedure S. HPLC analysis of the crude residue indicated a ratio indole/**6a** of 5:95 and an enantiomeric excess of (+) 6% [Chiralpak IA column, T = 30 °C, *n*-Hexane/*i*-PrOH = 97:3, 1 mL/min, λ = 280 nm, t_R = 30.505 min and t_R = 35.932 min].

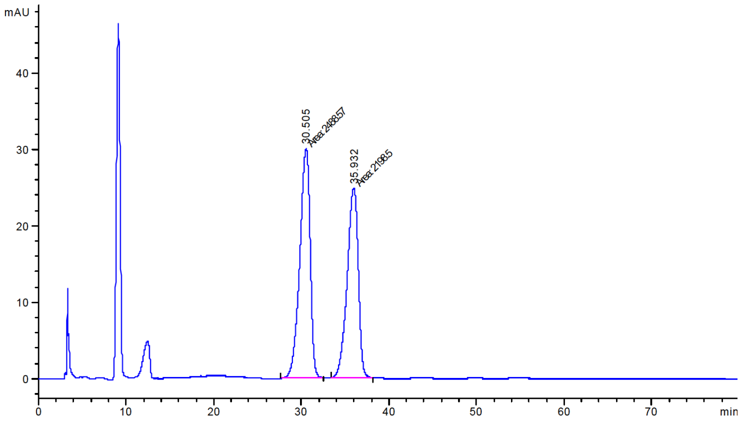

Peak	Retention Time	Rel. Area
	min	%
1	30.505	53.0944
2	35.932	46.9056

2-Methyl-3-(6-methyl-1*H*-indol-3-yl)-1-(thiazol-2-yl)propan-1-one (6b)

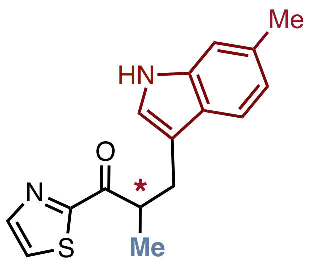

MW (g/mol): 284.38 **Molecular formula:** $C_{16}H_{16}N_2OS$

Synthesised according racemic procedure using **1e**. Isolated as yellow oil.

^{1}H NMR (400 MHz, $CDCl_3$) δ 8.02 (d, *J* = 3.0 Hz, 1H), 7.85 (br s, 1H, -*NH*), 7.63 (d, *J* = 3.0 Hz, 1H), 7.61 (d, *J* = 8.0 Hz, 1H), 7.11 (br s, 1H), 6.98 (dd, *J* = 8.0, 1.4 Hz, 1H), 6.93 (d, *J* = 2.3 Hz, 1H), 4.25 (m, 1H), 3.38 (dd, *J* = 14.3, 6.3 Hz, 1H), 2.88 (dd, *J* = 14.3, 7.9 Hz, 1H), 2.46 (s, 3H), 1.30 (d, *J* = 7.0 Hz, 3H).

^{13}C NMR (101 MHz, $CDCl_3$) δ 197.6, 167.1, 144.8, 136.8, 131.8, 126.3, 125.6, 122.0, 121.2, 118.9, 113.7, 111.1, 42.6, 28.9, 21.8, 16.9.

HRMS (ESI): m/z calcd for $C_{16}H_{17}N_2OS$, $[M+H]^+$: 285. 1061, found: 285.1067.

IR (neat): 3406, 1677, 1453, 1388, 800, 734 cm^{-1}.

HPLC: Chiralpak IB column, T = 30 °C, *n*-Hexane/*i*-PrOH = 92:8, 1 mL/min, λ = 300 nm.

Entry	General procedure	Sequences	Conversion (%)[a]	ee (%)[a]
**1**	P	**DNA-SerC2/RNA-U**	96	–77
**2**	S	st-DNA	99	+36

[a]Determined by HPLC. The sign before the ee's values is arbitrary evaluated.

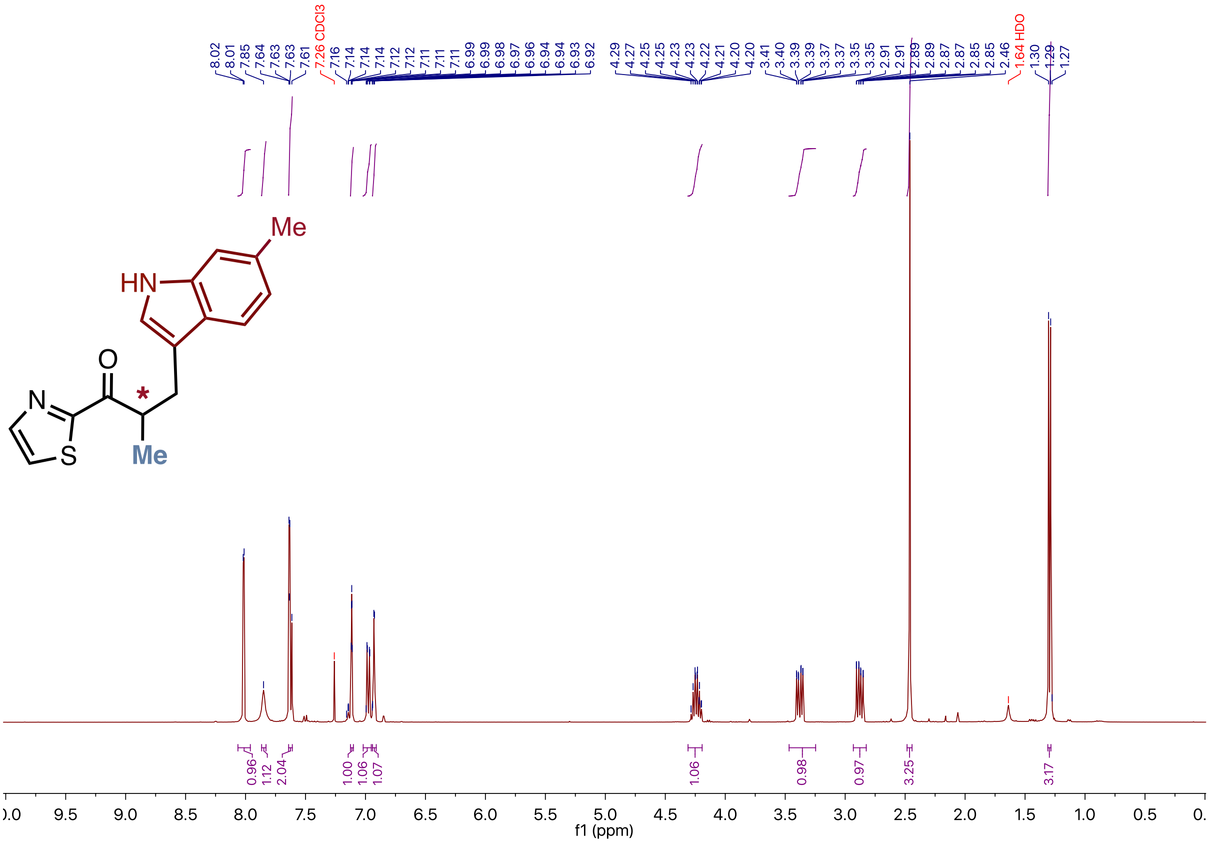
8.02
8.01
7.85
7.64
7.63
7.63
7.61
7.26 CDCl3
7.16
7.14
7.14
7.14
7.12
7.12
7.11
7.11
7.11
6.99
6.99
6.98
6.97
6.96
6.94
6.94
6.93
6.92
4.29
4.27
4.25
4.25
4.23
4.23
4.22
4.21
4.20
4.20
3.41
3.40
3.39
3.39
3.37
3.37
3.35
3.35
2.91
2.91
2.89
2.89
2.87
2.87
2.85
2.85
2.46
1.64 HDO
1.30
1.29
1.27
Me
HN
O
N
S
*
Me
0.96
1.12
2.04
1.00
1.06
1.07
1.06
0.98
0.97
3.25
3.17
f1 (ppm)

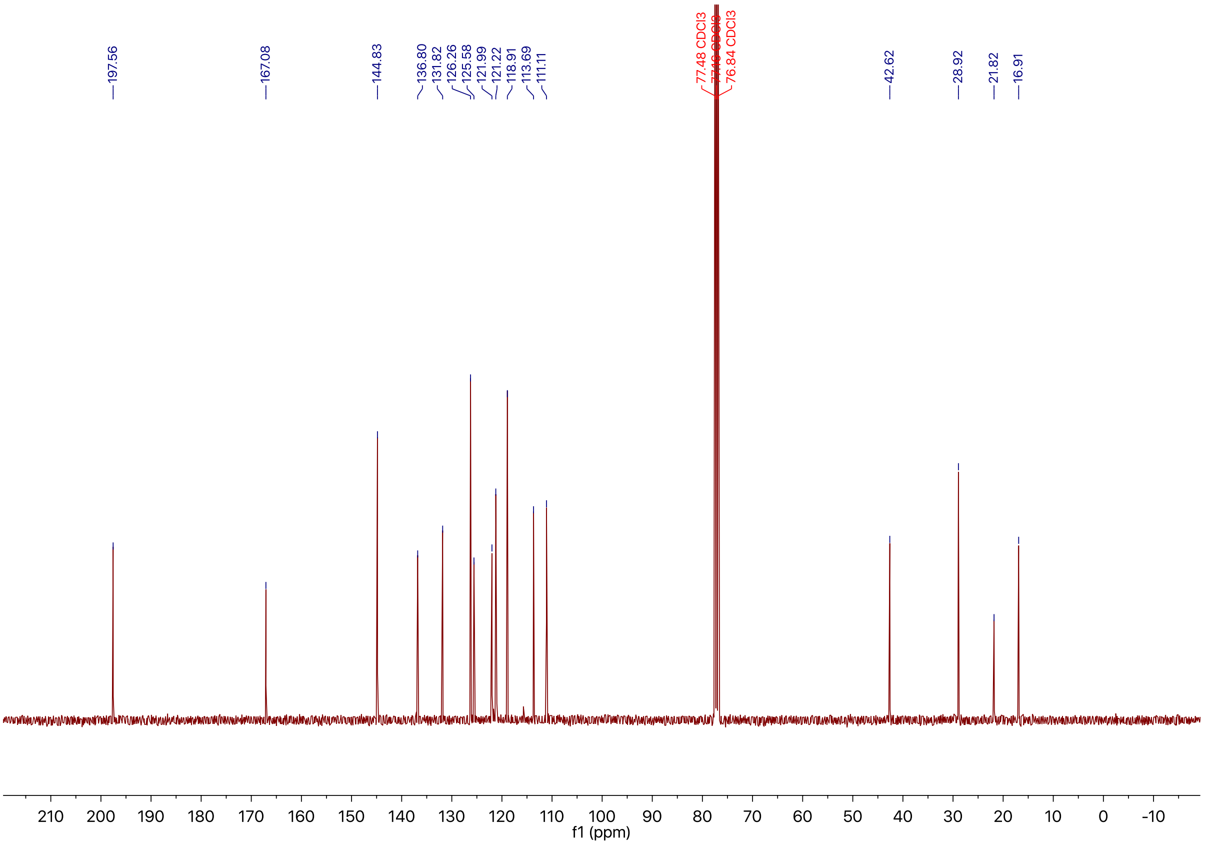
197.56
167.08
144.83
136.80
131.82
126.26
125.58
121.99
121.22
118.91
113.69
111.11
77.48 CDCl3
77.16 CDCl3
76.84 CDCl3
42.62
28.92
21.82
16.91
f1 (ppm)

Racemic [Chiralpak IB column, T = 30 °C, *n*-Hexane/*i*-PrOH = 92:8, 1 mL/min, λ = 300 nm, t_R = 15.375 min and t_R = 19.436 min].

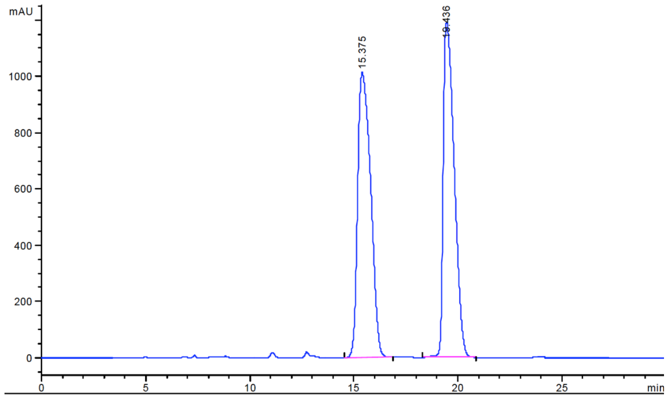

Peak	Retention Time	Rel. Area
	min	%
1	15.375	50.1345
2	19.436	49.8655

Indole [Chiralpak IB column, T = 30 °C, *n*-Hexane/*i*-PrOH = 92:8, 1 mL/min, λ = 300 nm, t_R = 8.736 min].

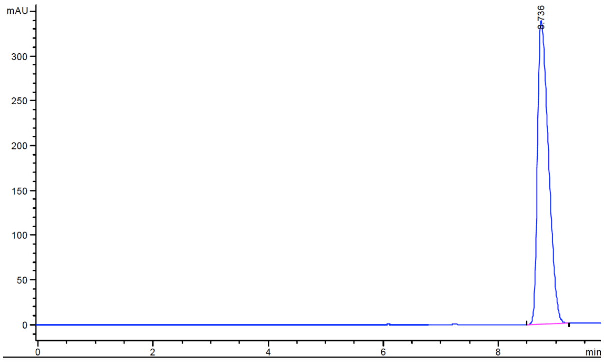

Following the general procedure P. HPLC analysis of the crude residue indicated a ratio indole/**6b** of 4:96 and an enantiomeric excess of (-) 77% [Chiralpak IB column, T = 30 °C, *n*-Hexane/*i*-PrOH = 92:8, 1 mL/min, λ = 300 nm, t_R = 15.632 min and t_R = 19.517 min].

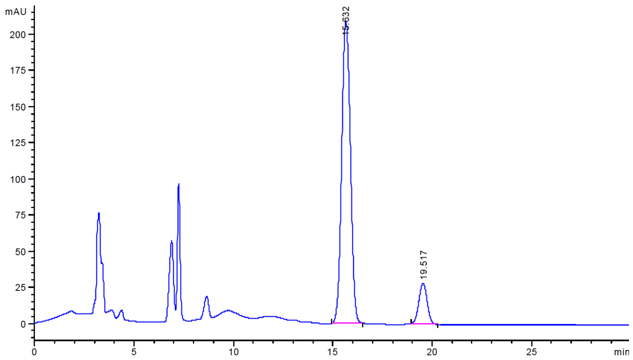

Peak	Retention Time	Rel. Area
	min	%
1	15.632	88.4507
2	19.517	11.5493

Following the general procedure S. HPLC analysis of the crude residue indicated a ratio indole/**6b** of 1:99 and an enantiomeric excess of (+) 36% [Chiralpak IB column, T = 30 °C, *n*-Hexane/*i*-PrOH = 92:8, 1 mL/min, λ = 300 nm, t_R = 15.744 min and t_R = 19.592 min].

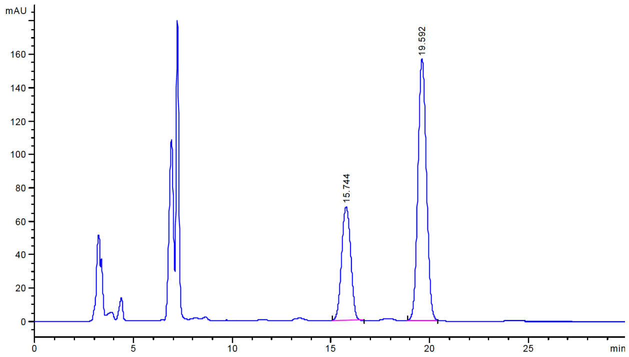

Peak	Retention Time	Rel. Area
	min	%
1	15.744	31.951
2	19.592	68.049

2-Methyl-3-(7-methyl-1*H*-indol-3-yl)-1-(thiazol-2-yl)propan-1-one (6c)

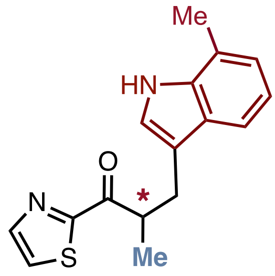

MW (g/mol): 284.38 **Molecular formula:** $C_{16}H_{16}N_2OS$

Synthesised according racemic procedure using **1e**. Isolated as yellow oil.

^{1}H NMR (400 MHz, $CDCl_3$) δ 8.02 (d, *J* = 3.0 Hz, 1H), 7.92 (br s, 1H, -*NH*), 7.63 (d, *J* = 3.0 Hz, 1H), 7.61 (d, *J* = 7.8 Hz, 1H), 7.07 (t, *J* = 7.8 Hz, 1H), 7.02-6.97 (m, 2H), 4.26 (m, 1H), 3.40 (dd, *J* = 14.4, 6.3 Hz, 1H), 2.90 (dd, *J* = 14.4, 7.9 Hz, 1H), 2.46 (s, 3H), 1.31 (d, *J* = 6.9 Hz, 3H).

^{13}C NMR (101 MHz, $CDCl_3$) δ 197.5, 167.1, 144.8, 135.9, 127.3, 126.3, 122.6, 122.4, 120.3, 119.7, 117.0, 114.4, 42.6, 29.0, 17.0, 16.7.

HRMS (ESI): m/z calcd for $C_{16}H_{17}N_2OS$, $[M+H]^+$: 285. 1061, found: 285.1067.

IR (neat): 3411, 1676, 1388, 926, 733 cm^{-1}.

HPLC: Chiralpak IA column, T = 30 °C, *n*-Hexane/*i*-PrOH = 98:2, 1 mL/min, λ = 280 nm.

Entry	General procedure	Sequences	Conversion (%)[a]	ee (%)[a]
**1**	P	**DNA-SerC2/RNA-U**	83	–84
**2**	S	st-DNA	82	+4

[a]Determined by HPLC. The sign before the ee's values is arbitrary evaluated.

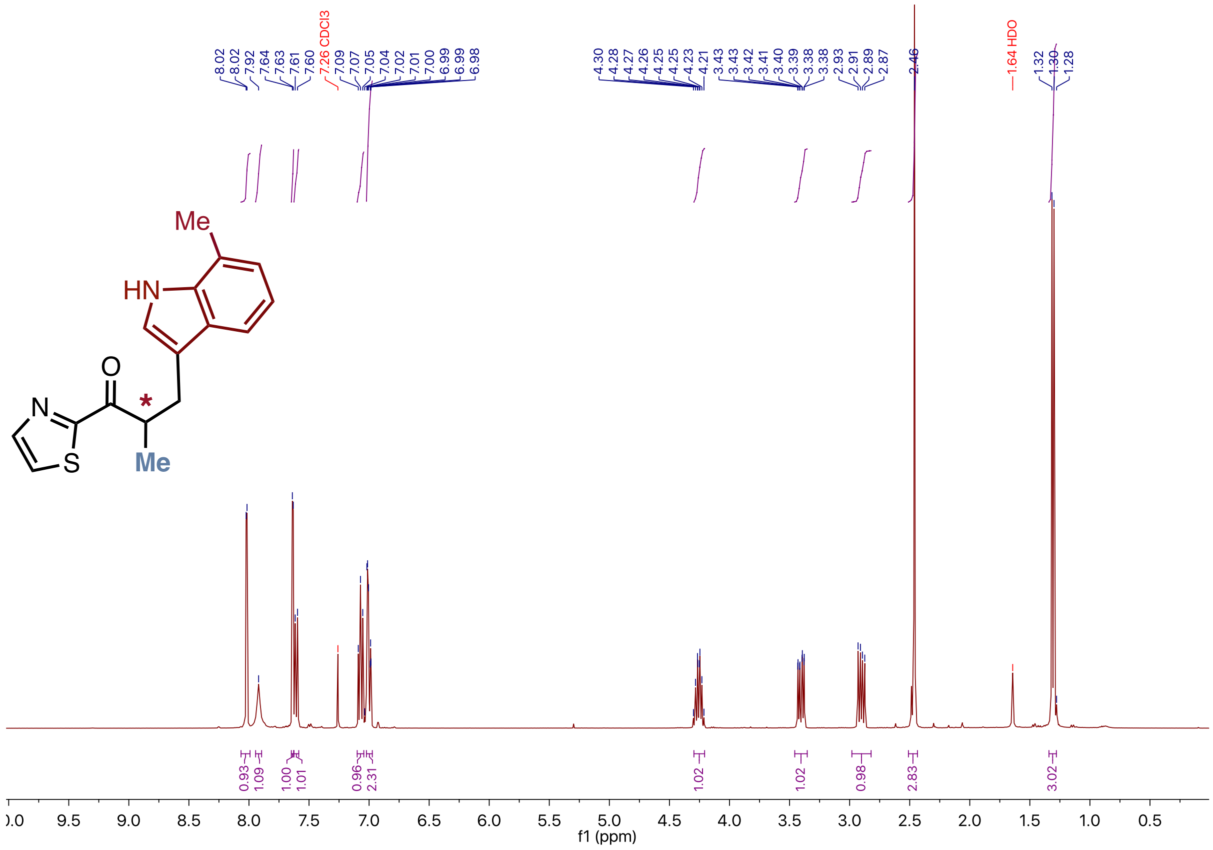

8.02
8.02
7.92
7.64
7.63
7.61
7.60
7.26 CDCl3
7.09
7.07
7.05
7.04
7.02
7.01
7.00
6.99
6.99
6.98
4.30
4.28
4.27
4.26
4.25
4.25
4.23
4.21
3.43
3.43
3.42
3.41
3.40
3.39
3.38
3.38
2.93
2.91
2.89
2.87
2.46
1.64 HDO
1.32
1.30
1.28
Me
HN
O
N
S
*
Me
0.93
1.09
1.00
1.01
0.96
2.31
1.02
1.02
0.98
2.83
3.02
9.5
9.0
8.5
8.0
7.5
7.0
6.5
6.0
5.5
5.0
4.5
4.0
3.5
3.0
2.5
2.0
1.5
1.0
0.5
f1 (ppm)

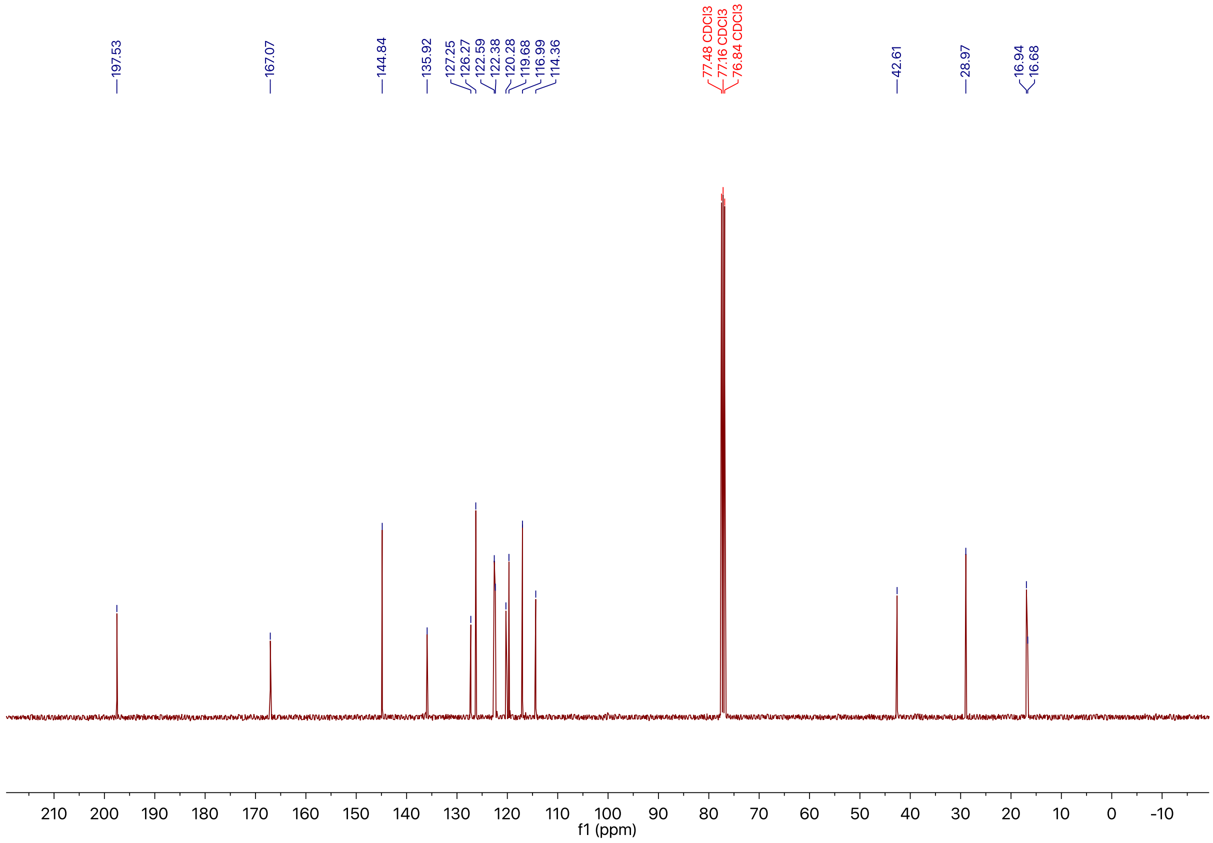

197.53
167.07
144.84
135.92
127.25
126.27
122.59
122.38
120.28
119.68
116.99
114.36
77.48 CDCl3
77.16 CDCl3
76.84 CDCl3
42.61
28.97
16.94
16.68
210
200
190
180
170
160
150
140
130
120
110
100
90
80
70
60
50
40
30
20
10
0
-10
f1 (ppm)

Racemic [Chiralpak IA column, T = 30 °C, *n*-Hexane/*i*-PrOH = 98:2, 1 mL/min, λ = 280 nm, t_R = 56.975 min and t_R = 66.388 min].

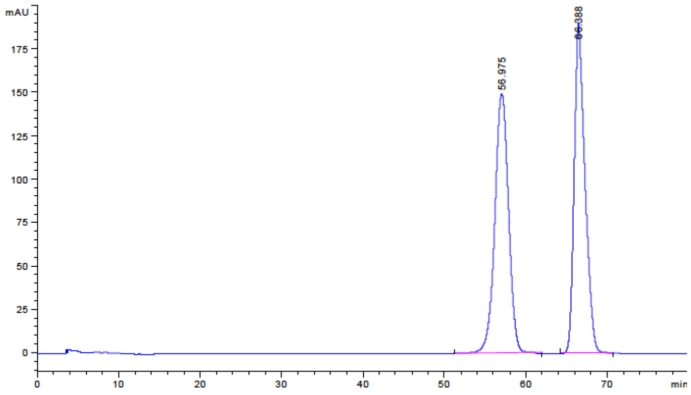

Peak	Retention Time	Rel. Area
	min	%
1	56.975	50.0494
2	66.388	49.9506

Indole [Chiralpak IA column, T = 30 °C, *n*-Hexane/*i*-PrOH = 98:2, 1 mL/min, λ = 280 nm, t_R = 12.066 min].

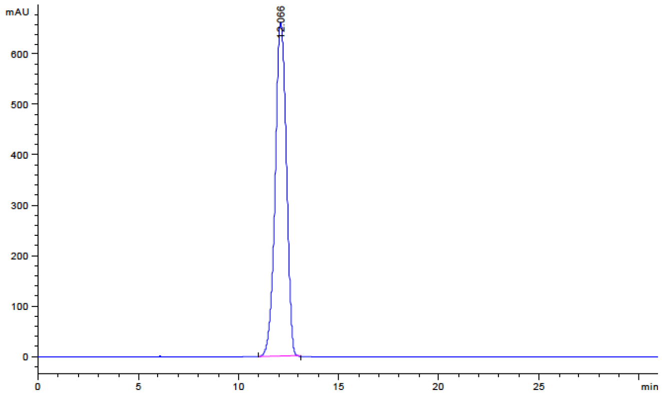

Following the general procedure P. HPLC analysis of the crude residue indicated a ratio indole/**6c** of 6:94 and an enantiomeric excess of (-) 84% [Chiralpak IA column, T = 30 °C, *n*-Hexane/*i*-PrOH = 98:2, 1 mL/min, λ = 280 nm, t_R = 53.181 min and t_R = 65.314 min].

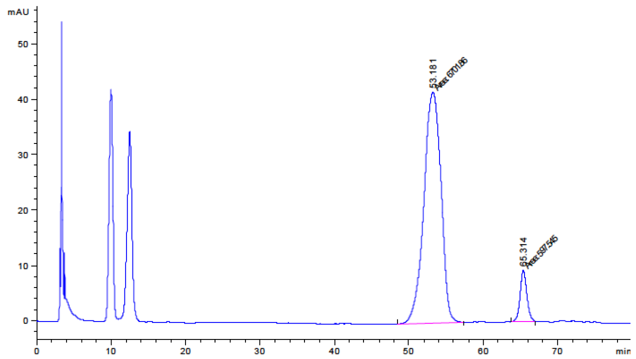

Peak	Retention Time	Rel. Area
	min	%
1	53.181	91.8138
2	65.314	8.1862

Following the general procedure S. HPLC analysis of the crude residue indicated a ratio indole/**6c** of 5:95 and an enantiomeric excess of (+) 4% [Chiralpak IA column, T = 30 °C, *n*-Hexane/*i*-PrOH = 98:2, 1 mL/min, λ = 280 nm, t_R = 54.213 min and t_R = 66.530 min].

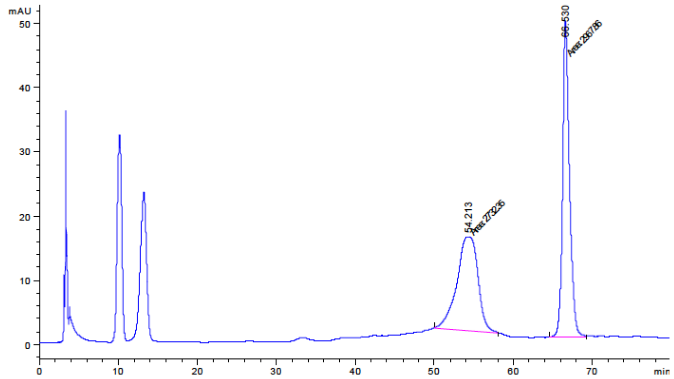

Peak	Retention Time	Rel. Area
	min	%
1	54.213	47.9342
2	66.53	52.0658

3-(5-Methoxy-1*H*-indol-3-yl)-2-methyl-1-(thiazol-2-yl)propan-1-one (6d)

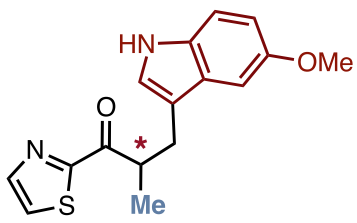

MW (g/mol): 300.38 **Molecular formula:** $C_{16}H_{16}N_2O_2S$

Synthesised according racemic procedure using **1e**. Isolated as yellow oil. The spectroscopic data of the product were identical with those reported in the literature.[11]

^{1}H NMR (400 MHz, CDCl$_3$) δ 8.02 (d, *J* = 3.0 Hz, 1H), 7.88 (br s, 1H, -*NH*), 7.66 (d, *J* = 3.0 Hz, 1H), 7.26 (d, *J* = 2.2 Hz, 1H), 7.22 (d, *J* = 8.8 Hz, 1H), 7.01 (s, 1H), 6.85 (dd, *J* = 8.8, 2.2 Hz, 1H), 4.24 (m, 1H), 3.91 (s, 3H), 3.38 (dd, *J* = 14.4, 6.5 Hz, 1H), 2.84 (dd, *J* = 14.4, 8.1 Hz, 1H), 1.30 (d, *J* = 6.5 Hz, 3H).

^{13}C NMR (101 MHz, CDCl$_3$) δ 197.5, 167.2, 154.1, 144.8, 131.5, 128.1, 126.4, 123.4, 113.7, 112.4, 111.9, 101.1, 56.0, 42.5, 29.2, 16.7.

HPLC: Chiralpak IA column, T = 20 °C, *n*-Hexane/*i*-PrOH = 95:5, 1 mL/min, λ = 280 nm.

Entry	General procedure	Sequences	Conversion (%)[a]	ee (%)[a]
**1**	P	**DNA-SerC2/RNA-U**	70	–80
**2**	S	st-DNA	83	+56

[a]Determined by HPLC. The sign before the ee's values is arbitrary evaluated.

Racemic [Chiralpak IA column, T = 20 °C, *n*-Hexane/*i*-PrOH = 95:5, 1 mL/min, λ = 280 nm, t_R = 38.389 min and t_R = 41.802 min].

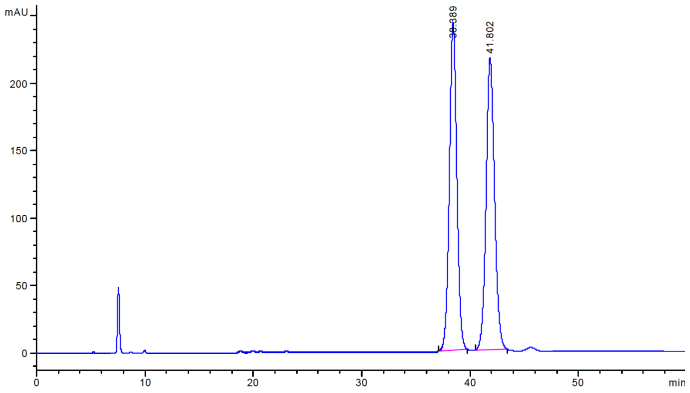

Peak	Retention Time	Rel. Area
	min	%
1	38.389	49.7682
2	41.802	50.2318

Indole [Chiralpak IA column, T = 20 °C, *n*-Hexane/*i*-PrOH = 95:5, 1 mL/min, λ = 280 nm, t_R = 18.965 min].

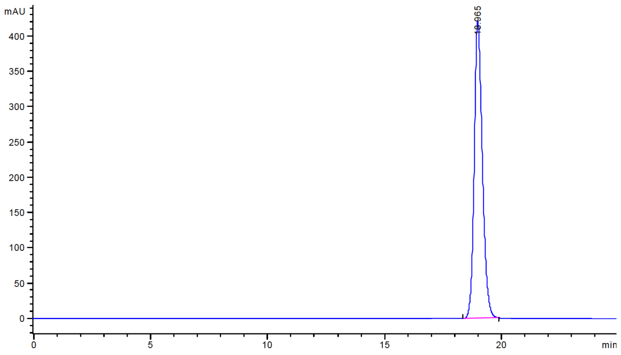

Following the general procedure P. HPLC analysis of the crude residue indicated a ratio indole/**6d** of 30:70 and an enantiomeric excess of (-) 80% [Chiralpak IA column, T = 20 °C, *n*-Hexane/*i*-PrOH = 95:5, 1 mL/min, λ = 280 nm, t_R = 39.681 min and t_R = 44.357 min].

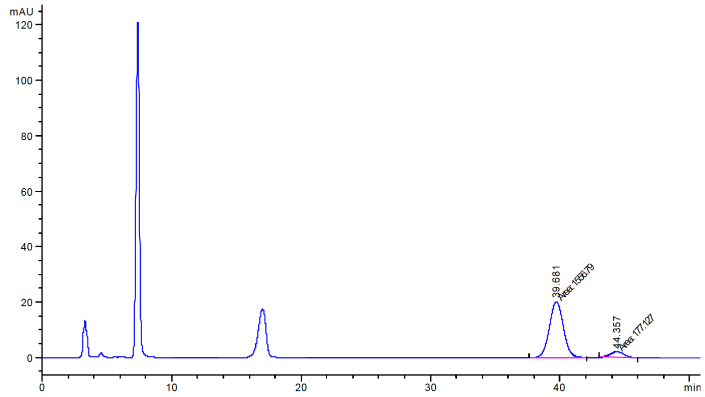

Peak	Retention Time	Rel. Area
	min	%
1	39.681	89.7846
2	44.357	10.2154

Following the general procedure S. HPLC analysis of the crude residue indicated a ratio indole/**6d** of 17:83 and an enantiomeric excess of (+) 56% [Chiralpak IA column, T = 20 °C, *n*-Hexane/*i*-PrOH = 95:5, 1 mL/min, λ = 280 nm, t_R = 38.371 min and t_R = 42.558 min].

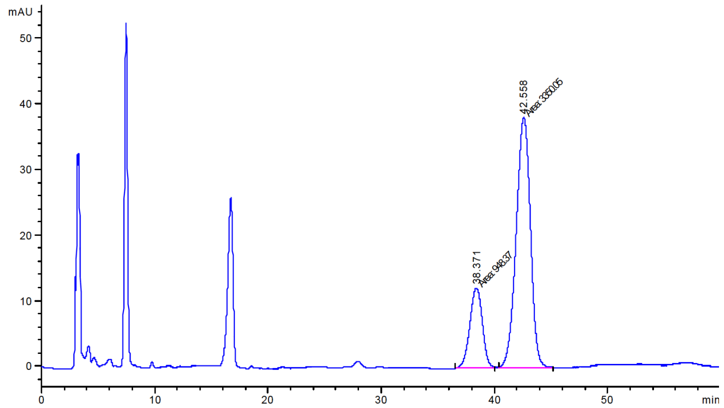

Peak	Retention Time	Rel. Area
	min	%
1	38.371	22.0632
2	42.558	77.9368

3-(5-Fluoro-1*H*-indol-3-yl)-2-methyl-1-(thiazol-2-yl)propan-1-one (6e)

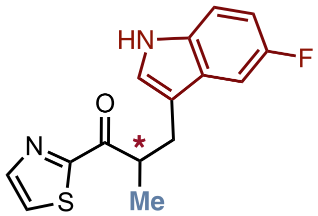

MW (g/mol): 288.07 **Molecular formula:** $C_{15}H_{13}FN_2OS$

Synthesised according racemic procedure using **1e**. Isolated as yellow oil.

^{1}H NMR (400 MHz, $CDCl_3$) δ 8.03 (d, *J* = 3.1 Hz, 1H), 8.01 (s, 1H), 7.65 (d, *J* = 3.1 Hz, 1H), 7.39 (dd, *J* = 9.7, 2.5 Hz, 1H), 7.22 (dd, *J* = 9.7, 4.3 Hz, 1H), 7.04 (d, *J* = 2.4 Hz, 1H), 6.92 (td, *J* = 9.0, 2.5 Hz, 1H), 4.20 (m, 1H), 3.34 (dd, *J* = 14.4, 6.3 Hz, 1H), 2.84 (dd, *J* = 14.4, 7.9 Hz, 1H), 1.29 (d, *J* = 6.9 Hz, 3H).

^{13}C NMR (101 MHz, $CDCl_3$) δ 197.3, 167.0, 157.9 (d, *J* = 234.3 Hz), 144.9, 132.8, 128.1 (d, *J* = 9.8 Hz), 126.4, 124.4, 114.1 (d, *J* = 4.8 Hz), 111.7 (d, *J* = 9.7 Hz), 110.4 (d, *J* = 26.5 Hz), 104.2 (d, *J* = 23.5 Hz), 42.5, 28.9, 16.8.

^{19}F NMR (377 MHz, $CDCl_3$) δ –124.7.

HRMS (ESI): m/z calcd for $C_{15}H_{13}FN_2OSNa$, $[M+Na]^+$: 307.0630, found: 307.0671.

IR (neat): 3410, 1678, 1485, 1389, 936 cm^{-1}.

HPLC: Chiralpak IA column, T = 30 °C, *n*-Hexane/*i*-PrOH = 97:3, 1 mL/min, λ = 280 nm.

Entry	General procedure	Sequences	Conversion (%)[a]	ee (%)[a]
**1**	P	**DNA-SerC2/RNA-U**	73	–76
**2**	S	st-DNA	82	+16

[a]Determined by HPLC. The sign before the ee's values is arbitrary evaluated.

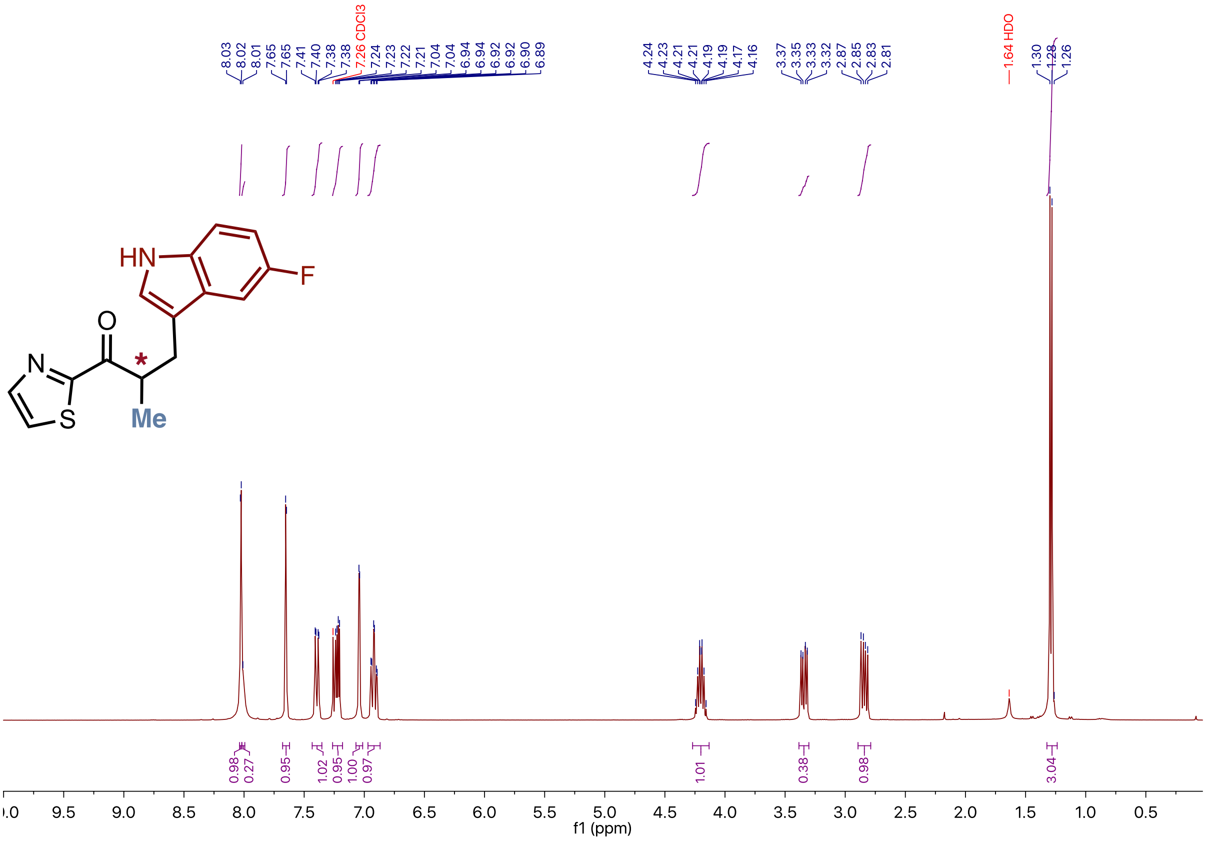

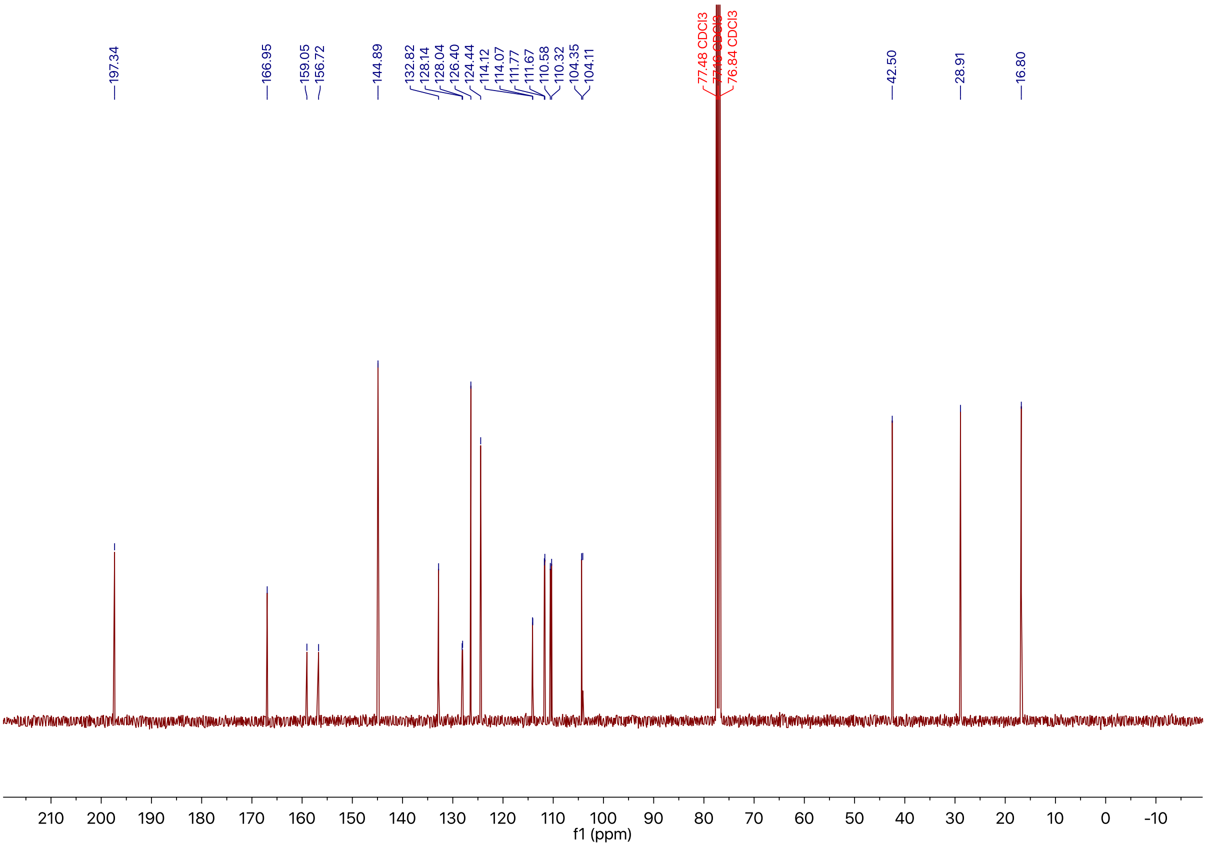

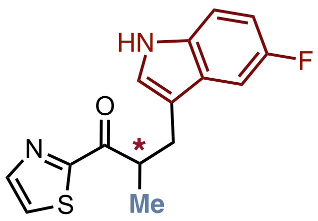
HN
F
O
N
S
Me

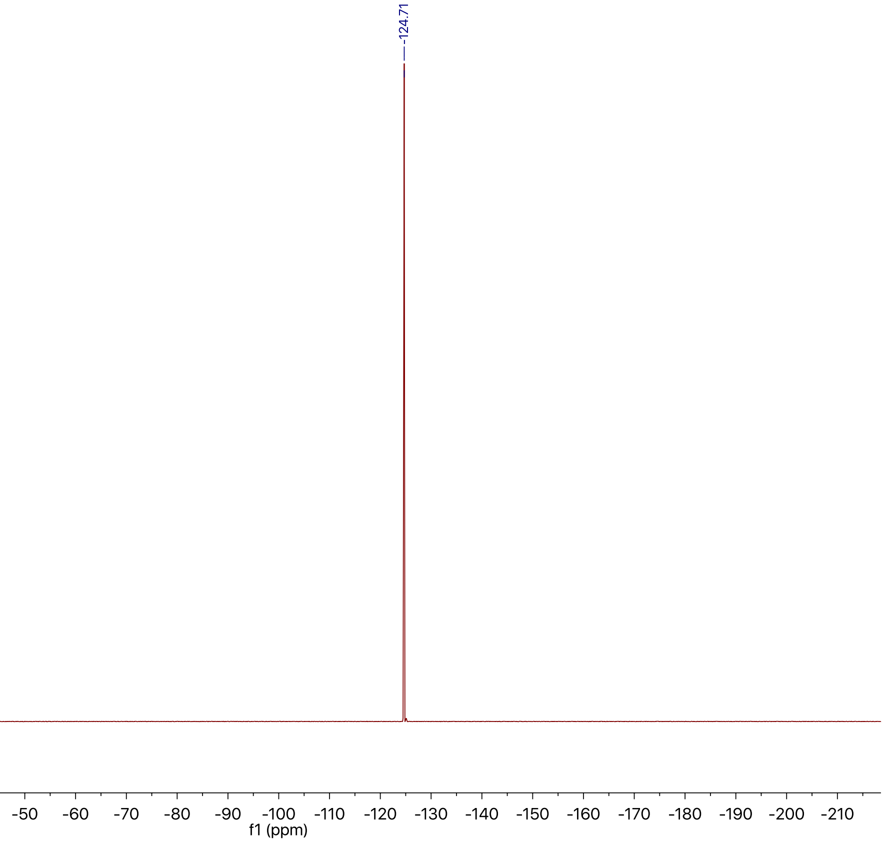
-124.71
10
0
-10
-20
-30
-40
-50
-60
-70
-80
-90
-100
-110
-120
-130
-140
-150
-160
-170
-180
-190
-200
-210
f1 (ppm)

Racemic [Chiralpak IA column, T = 30 °C, *n*-Hexane/*i*-PrOH = 97:3, 1 mL/min, λ = 280 nm, t_R = 45.925 min and t_R = 53.440 min].

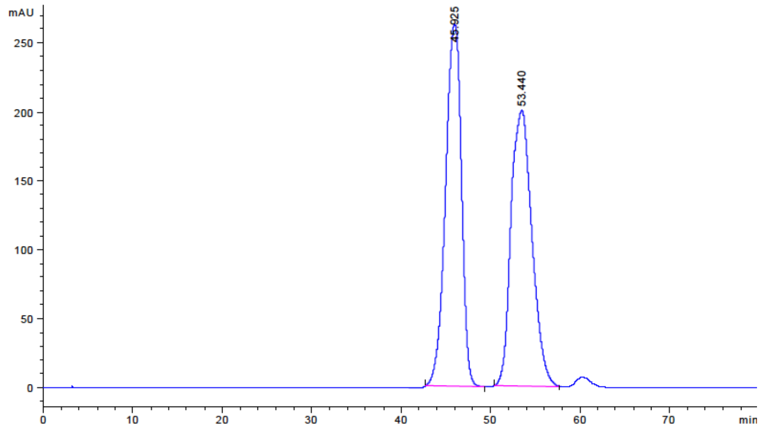

Peak	Retention Time	Rel. Area
	min	%
1	45.925	50.0692
2	53.44	49.9308

Indole [Chiralpak IA column, T = 30 °C, *n*-Hexane/*i*-PrOH = 97:3, 1 mL/min, λ = 280 nm, t_R = 16.861 min].

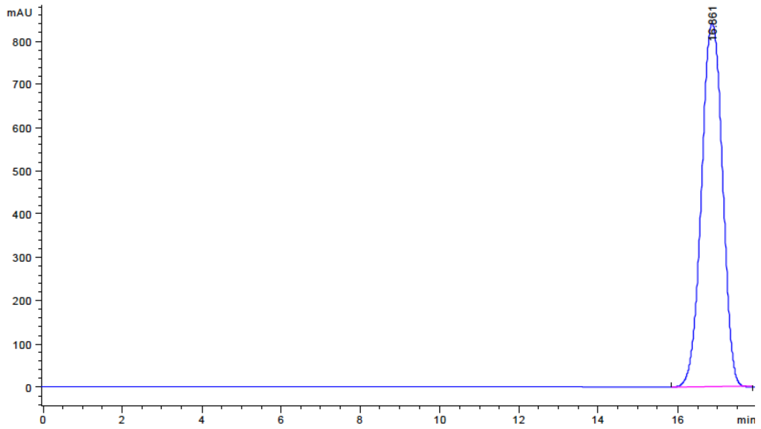

Following the general procedure P. HPLC analysis of the crude residue indicated a ratio indole/**6e** of 27:73 and an enantiomeric excess of (-) 76% [Chiralpak IA column, T = 30 °C, *n*-Hexane/*i*-PrOH = 97:3, 1 mL/min, λ = 280 nm, t_R = 50.125 min and t_R = 61.704 min].

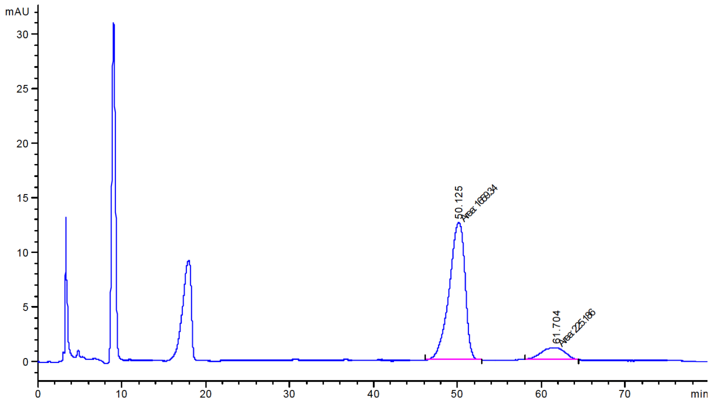

Peak	Retention Time	Rel. Area
	min	%
1	50.125	88.0508
2	61.704	11.9492

Following the general procedure S. HPLC analysis of the crude residue indicated a ratio indole/**6e** of 18:82 and an enantiomeric excess of (+) 16% [Chiralpak IA column, T = 30 °C, *n*-Hexane/*i*-PrOH = 97:3, 1 mL/min, λ = 280 nm, t_R = 50.807 min and t_R = 58.878 min].

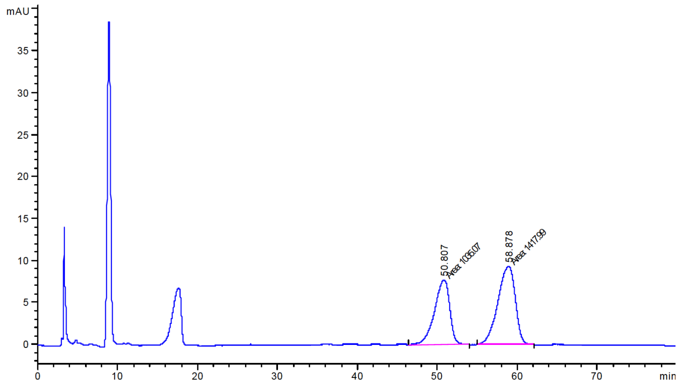

Peak	Retention Time	Rel. Area
	min	%
1	50.807	42.1951
2	58.878	57.8049

3-(1*H*-Indol-3-yl)-2-methyl-1-(thiazol-2-yl)propan-1-one (6f)

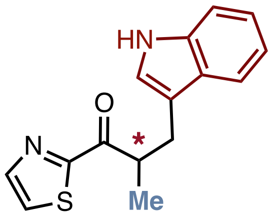

MW (g/mol): 270.35 **Molecular formula:** $C_{15}H_{14}N_2OS$

Synthesised according racemic procedure using **1e**. Isolated as yellow oil. The spectroscopic data of the product were identical with those reported in the literature.[11]

^{1}H NMR (400 MHz, $CDCl_3$) δ 8.01 (d, *J* = 3.0 Hz, 1H), 8.00 (br s, 1H, -*NH*), 7.73 (d, *J* = 7.3 Hz, 1H), 7.64 (d, *J* = 3.0 Hz, 1H), 7.32 (d, *J* = 7.7 Hz, 1H), 7.18 (td, *J* = 7.3, 1.2 Hz, 1H), 7.13 (td, *J* = 7.7, 1.2 Hz, 1H), 7.01 (d, *J* = 2.2 Hz, 1H), 4.25 (m, 1H), 3.40 (dd, *J* = 14.4, 6.3 Hz, 1H), 2.90 (dd, *J* = 14.4, 7.9 Hz, 1H), 1.30 (d, *J* = 6.9 Hz, 3H).

^{13}C NMR (101 MHz, $CDCl_3$) δ 197.5, 167.1, 144.8, 136.3, 127.7, 126.3, 122.6, 122.1, 119.5, 119.3, 113.9, 111.1, 42.6, 28.9, 17.0.

HPLC: Chiralpak IA column, T = 30 °C, *n*-Hexane/*i*-PrOH = 97:3, 1 mL/min, λ = 280 nm.

Entry	General procedure	Sequences	Conversion (%)[a]	ee (%)[a]
**1**	P	**DNA-SerC2/RNA-U**	89	–76
**2**	S	st-DNA	95	+20

[a]Determined by HPLC. The sign before the ee's values is arbitrary evaluated.

Racemic [Chiralpak IA column, T = 30 °C, *n*-Hexane/*i*-PrOH = 97:3, 1 mL/min, λ = 280 nm, t_R = 44.430 min and t_R = 53.322 min].

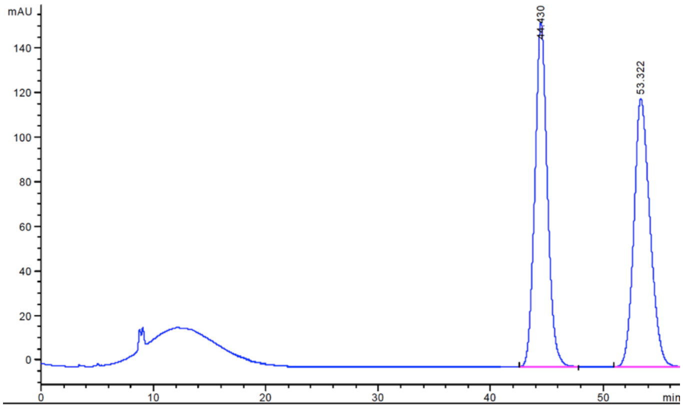

Peak	Retention Time	Rel. Area
	min	%
1	44.430	49.9295
2	53.322	50.0705

Indole [Chiralpak IA column, T = 30 °C, *n*-Hexane/*i*-PrOH = 97:3, 1 mL/min, λ = 280 nm, t_R = 13.494 min].

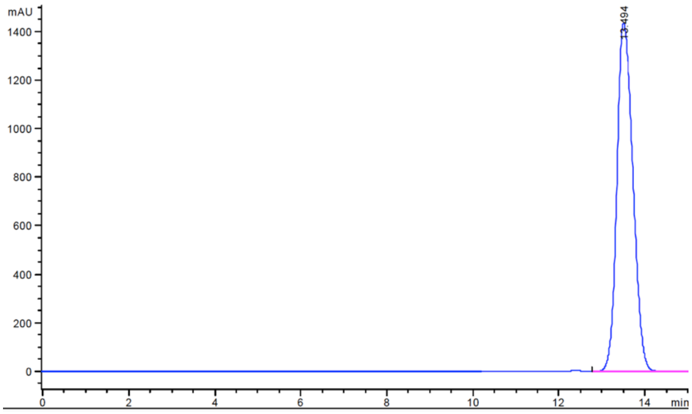

Following the general procedure P. HPLC analysis of the crude residue indicated a ratio indole/**6f** of 11:89 and an enantiomeric excess of (-) 76% [Chiralpak IA column, T = 30 °C, *n*-Hexane/*i*-PrOH = 97:3, 1 mL/min, λ = 280 nm, t_R = 50.066 min and t_R = 57.058 min].

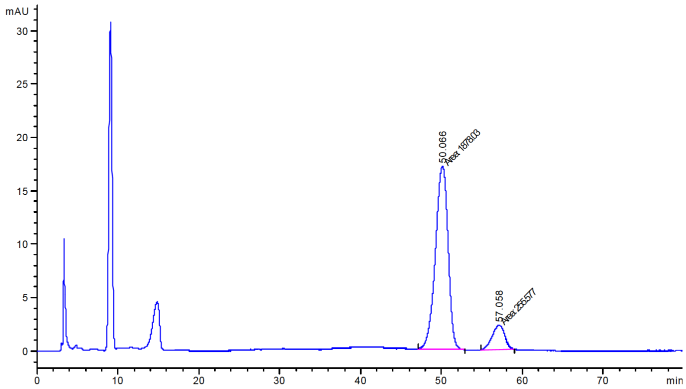

Peak	Retention Time	Rel. Area
	min	%
1	50.066	88.0214
2	57.058	11.9786

Following the general procedure S. HPLC analysis of the crude residue indicated a ratio indole/**6f** of 5:95 and an enantiomeric excess of (+) 20% [Chiralpak IA column, T = 30 °C, *n*-Hexane/*i*-PrOH = 97:3, 1 mL/min, λ = 280 nm, t_R = 51.192 min and t_R = 58.357 min].

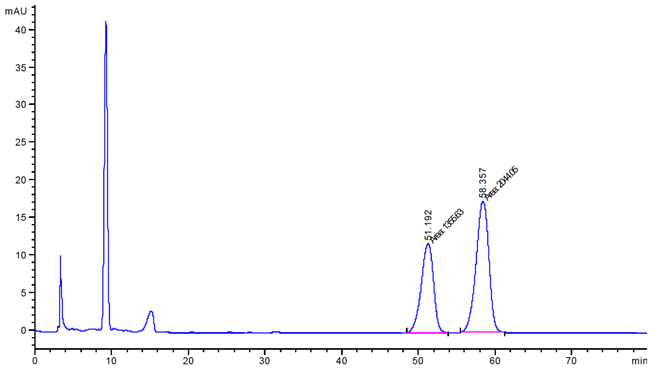

Peak	Retention Time	Rel. Area
	min	%
1	51.192	39.8753
2	58.357	60.1247

2-Methyl-3-(5-morpholino-1*H*-indol-3-yl)-1-(thiazol-2-yl)propan-1-one (6g)

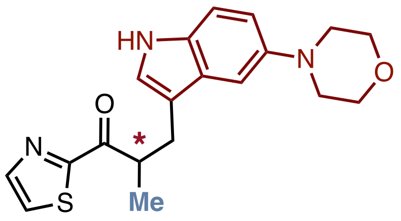

MW (g/mol): 355.14 **Molecular formula:** $C_{19}H_{21}N_3O_2S$

Synthesised according racemic procedure using **1e**. Isolated as yellow oil. The spectroscopic data of the product were identical with those reported in the literature.[11]

^{1}H NMR (400 MHz, CDCl$_3$) δ 8.00 (d, *J* = 3.1 Hz, 1H), 7.82 (br s, 1H, -*NH*), 7.66 (d, *J* = 3.1 Hz, 1H), 7.33 (d, *J* = 2.3 Hz, 1H), 7.24 (d, *J* = 8.8 Hz, 1H), 6.99 (d, *J* = 2.3 Hz, 1H), 6.94 (dd, *J* = 8.8, 2.3 Hz, 1H), 4.22 (m, 1H), 3.93 (t, *J* = 4.7 Hz, 4H), 3.37 (dd, *J* = 14.4, 5.9 Hz, 1H), 3.18 (t, *J* = 4.7 Hz, 4H), 2.82 (dd, *J* = 14.4, 8.2 Hz, 1H), 1.29 (d, *J* = 6.9 Hz, 3H).

^{13}C NMR (101 MHz, CDCl$_3$) δ 197.5, 167.6, 145.8, 144.7, 132.0, 128.2, 126.4, 123.2, 115.2, 113.8, 111.7, 106.3, 67.5 (2C), 52.2 (2C), 42.4, 29.2, 16.6.

HPLC: Chiralpak IA column, T = 30 °C, *n*-Hexane/*i*-PrOH = 90:10, 1 mL/min, λ = 280 nm.

Entry	General procedure	Sequences	Conversion (%)[a]	ee (%)[a]
**1**	P	**DNA-SerC2/RNA-U**	80	–90
**2**	S	st-DNA	>99	+80

[a]Determined by HPLC. The sign before the ee's values is arbitrary evaluated.

Racemic [Chiralpak IA column, T = 30 °C, *n*-Hexane/*i*-PrOH = 90:10, 1 mL/min, λ = 280 nm, t_R = 27.969 min and t_R = 36.836 min].

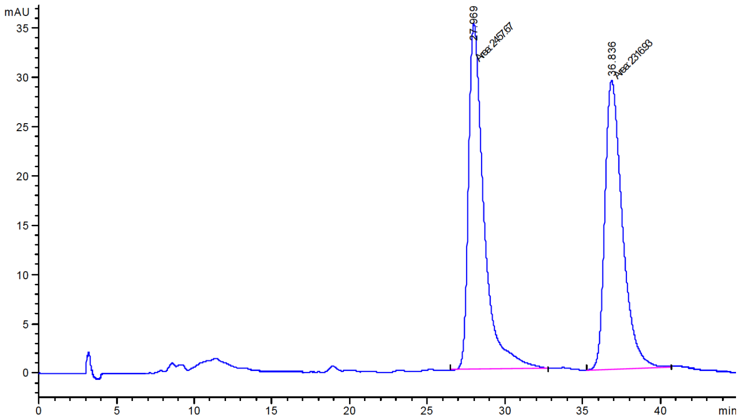

Peak	Retention Time	Rel. Area
	min	%
1	27.969	51.4738
2	36.836	48.5262

Indole [Chiralpak IA column, T = 30 °C, *n*-Hexane/*i*-PrOH = 90:10, 1 mL/min, λ = 280 nm, t_R = 14.237 min].

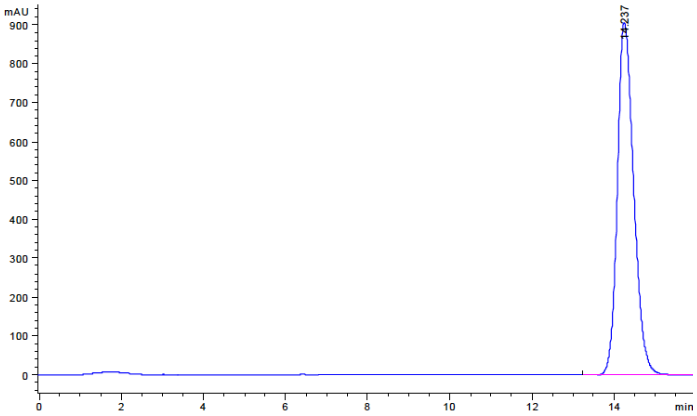

Following the general procedure P. HPLC analysis of the crude residue indicated a ratio indole/**6g** of 9:91 and an enantiomeric excess of (-) 90% [Chiralpak IA column, T = 30 °C, *n*-Hexane/*i*-PrOH = 90:10, 1 mL/min, λ = 280 nm, t_R = 29.708 min and t_R = 34.661 min].

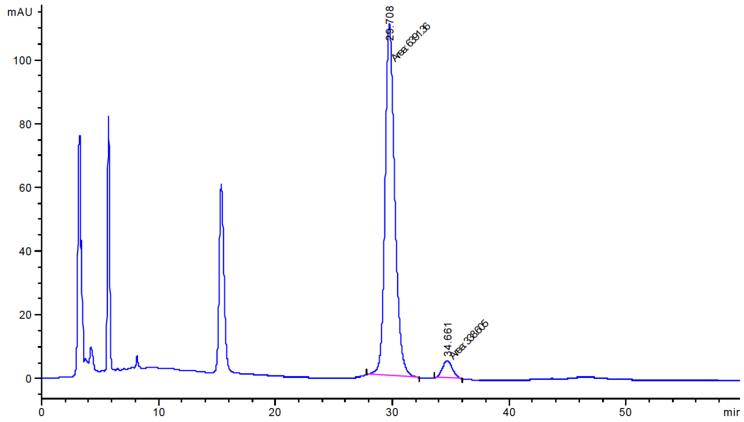

Peak	Retention Time	Rel. Area
	min	%
1	29.708	94.9687
2	34.661	5.0313

Following the general procedure S. HPLC analysis of the crude residue indicated a ratio indole/**6g** of 11:89 and an enantiomeric excess of (+) 80% [Chiralpak IA column, T = 30 °C, *n*-Hexane/*i*-PrOH = 90:10, 1 mL/min, λ = 280 nm, t_R = 30.195 min and t_R = 34.730 min].

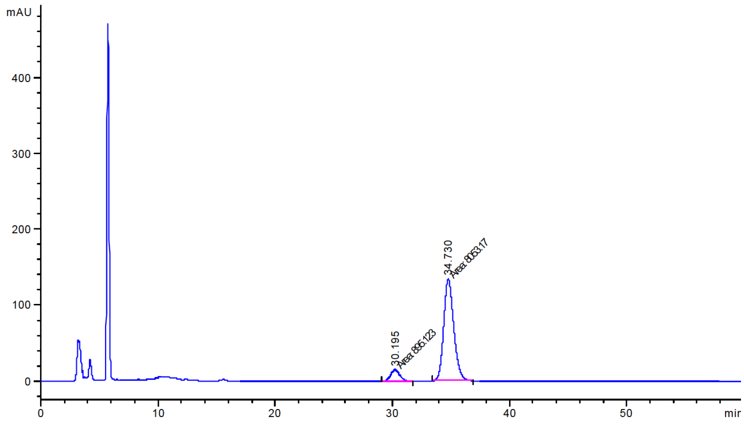

Peak	Retention Time	Rel. Area
	min	%
1	30.195	10.0033
2	34.73	89.9967

2-Methyl-3-(5-(piperidin-1-yl)-1*H*-indol-3-yl)-1-(thiazol-2-yl)propan-1-one (6h)

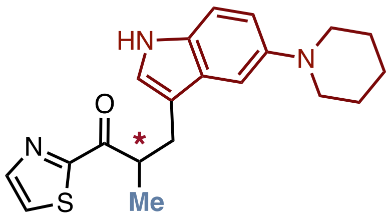

MW (g/mol): 353.16 **Molecular formula:** $C_{20}H_{23}N_3OS$

Synthesised according racemic procedure using **1e**. Isolated as yellow oil. The spectroscopic data of the product were identical with those reported in the literature.[11]

^{1}H NMR (400 MHz, $CDCl_3$) δ 8.01 (d, *J* = 3.0 Hz, 1H), 7.78 (br s, 1H, -*NH*), 7.65 (d, *J* = 3.0 Hz, 1H), 7.36 (d, *J* = 2.4 Hz, 1H), 7.22 (d, *J* = 8.8 Hz, 1H), 6.99 (d, *J* = 8.8 Hz, 1H), 6.97 (d, *J* = 2.3 Hz, 1H), 4.23 (m, 1H), 3.36 (dd, *J* = 14.2, 5.8 Hz, 1H), 3.14 (m, 4H), 2.81 (dd, *J* = 14.2, 8.2 Hz, 1H), 1.83-1.75 (m, 4H), 1.67-1.54 (m, 2H), 1.29 (d, *J* = 6.8 Hz, 3H).

^{13}C NMR (101 MHz, $CDCl_3$) δ 195.7, 165.1, 144.8, 142.6, 134.8, 128.1, 126.3, 122.8, 116.5, 113.5, 111.4, 108.9, 53.7 (2C), 42.5, 29.3, 26.6 (2C), 24.5, 16.6.

HPLC: Chiralpak IB column, T = 30 °C, *n*-Hexane/*i*-PrOH = 92:8, 1 mL/min, λ = 300 nm.

Entry	General procedure	Sequences	Conversion (%)[a]	ee (%)[a]
**1**	P	**DNA-SerC2/RNA-U**	91	–93
**2**	R	st-DNA	89	+70

[a]Determined by HPLC. The sign before the ee's values is arbitrary evaluated.

Racemic [Chiralpak IB column, T = 30 °C, *n*-Hexane/*i*-PrOH = 92:8, 1 mL/min, λ = 300 nm, t_R = 27.286 min and t_R = 34.958 min].

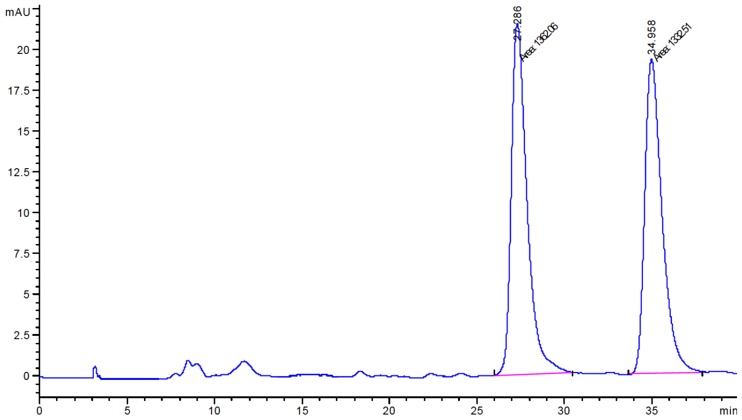

Peak	Retention Time	Rel. Area
	min	%
1	27.286	50.5482
2	34.958	49.4518

Indole [Chiralpak IA column, T = 30 °C, *n*-Hexane/*i*-PrOH = 90:10, 1 mL/min, λ = 300 nm, t_R = 16.386 min].

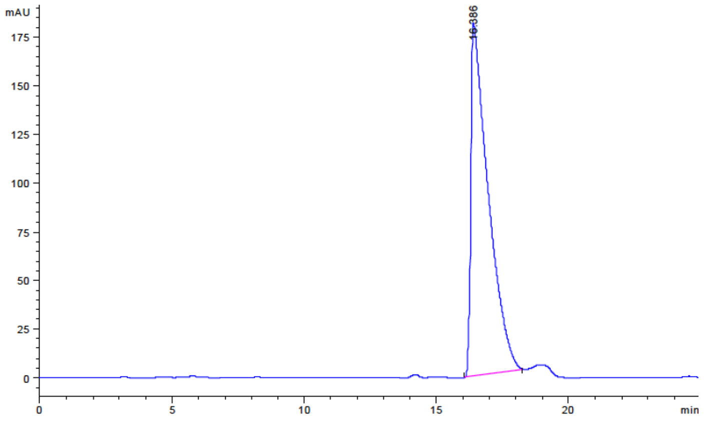

Following the general procedure P. HPLC analysis of the crude residue indicated a ratio indole/**6h** of 9:91 and an enantiomeric excess of (-) 93% [Chiralpak IB column, T = 30 °C, *n*-Hexane/*i*-PrOH = 92:8, 1 mL/min, λ = 300 nm, t_R = 25.791 min and t_R = 31.452 min].

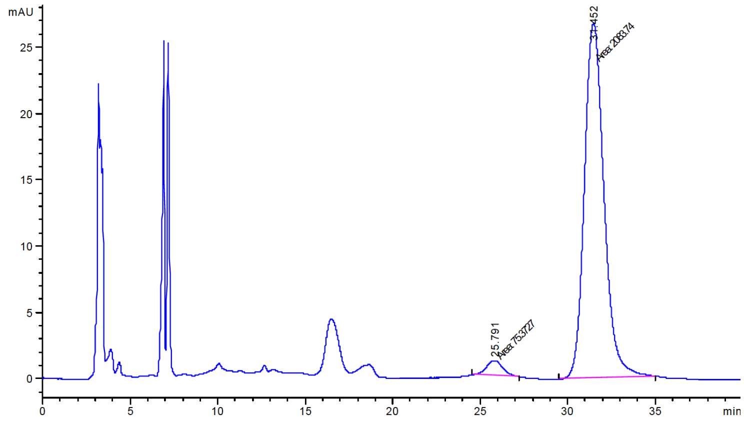

Peak	Retention Time	Rel. Area
	min	%
1	25.791	3.4909
2	31.452	96.5091

Following the general procedure S. HPLC analysis of the crude residue indicated a ratio indole/**6h** of 11:89 and an enantiomeric excess of (+) 70% [Chiralpak IB column, T = 30 °C, *n*-Hexane/*i*-PrOH = 92:8, 1 mL/min, λ = 300 nm, t_R = 25.666 min and t_R = 31.918 min].

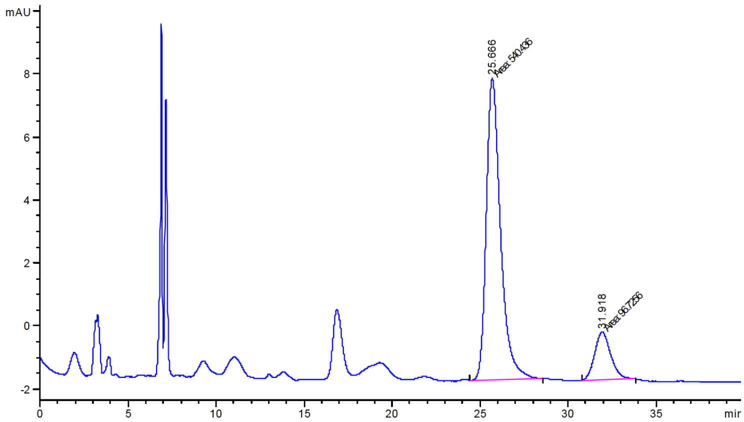

Peak	Retention Time	Rel. Area
	min	%
1	25.666	84.8193
2	31.918	15.1807

2-((5-Methoxy-1*H*-indol-3-yl)methyl)-1-(thiazol-2-yl)butan-1-one (6i)

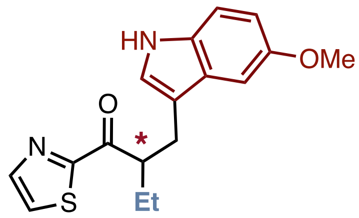

MW (g/mol): 314.40 **Molecular formula:** $C_{17}H_{18}N_2O_2S$

Synthesised according racemic procedure using **1f**. Isolated as yellow oil.

^{1}H NMR (400 MHz, $CDCl_3$) δ 7.99 (d, *J* = 3.0 Hz, 1H), 7.80 (br s, 1H, -*NH*), 7.63 (d, *J* = 3.0 Hz, 1H), 7.23-7.15 (m, 2H), 6.97 (d, *J* = 2.4 Hz, 1H), 6.83 (dd, *J* = 8.7, 2.4 Hz, 1H), 4.17 (m, 1H), 3.90 (s, 3H), 3.29 (dd, *J* = 14.5, 7.0 Hz, 1H), 2.91 (dd, *J* = 14.5, 7.2 Hz, 1H), 1.93 (m, 1H), 1.74 (m, 1H), 0.93 (t, *J* = 7.5 Hz, 3H).

^{13}C NMR (101 MHz, $CDCl_3$) δ 197.4, 167.8, 154.1, 144.8, 131.4, 128.1, 126.3, 123.3, 113.8, 112.4, 111.8, 101.1, 56.0, 49.3, 27.4, 24.9, 11.9.

HRMS (ESI): m/z calcd for $C_{17}H_{19}N_2O_2S$, $[M+H]^+$: 315.1167, found: 315.1190.

IR (neat): 3404, 2963, 1678, 1484, 1391, 1218, 1063, 799 cm^{-1}.

HPLC: Chiralpak IA column, T = 20 °C, *n*-Hexane/*i*-PrOH = 95:5, 1 mL/min, λ = 280 nm.

Entry	General procedure	Sequences	Conversion (%)[a]	ee (%)[a]
**1**	P	**DNA-SerC2/RNA-U**	84	–75
**2**	S	st-DNA	85	+51

[a]Determined by HPLC. The sign before the ee's values is arbitrary evaluated.

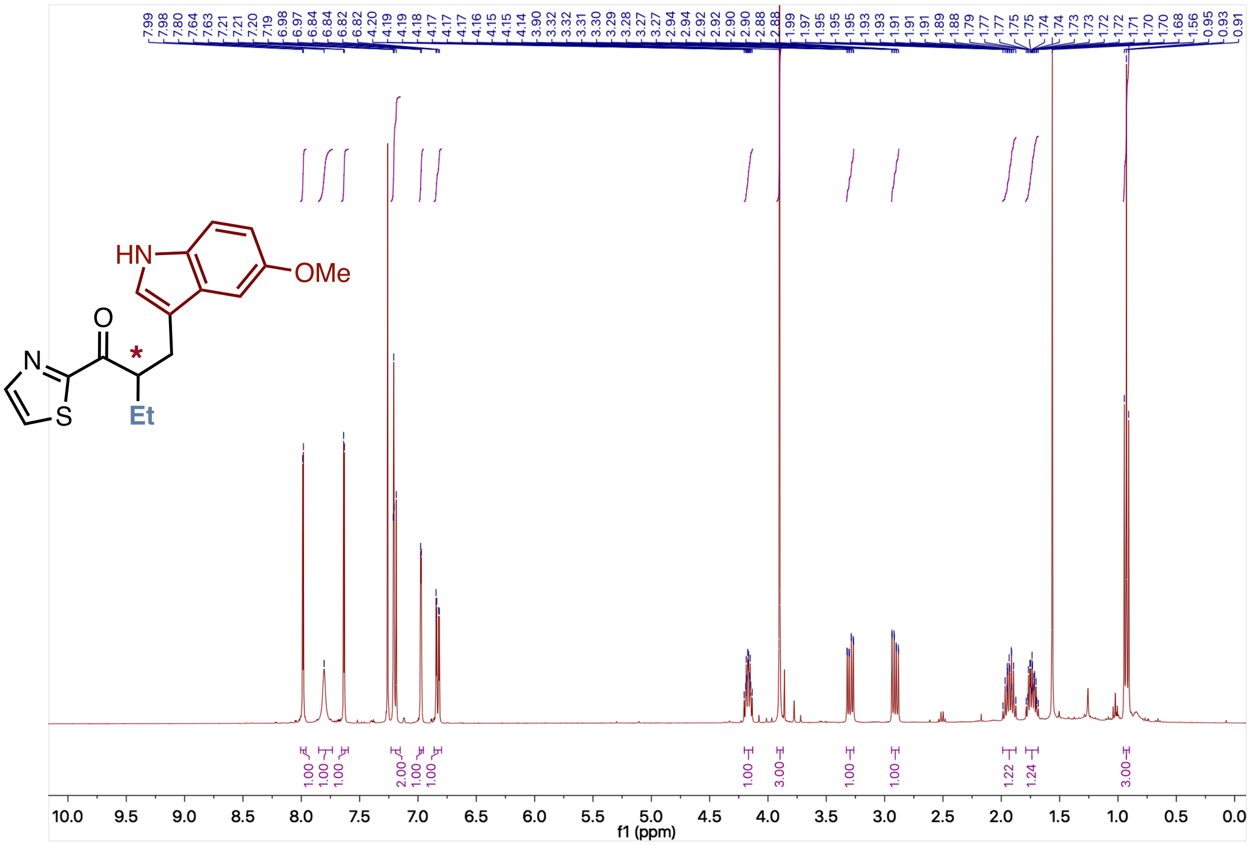
HN
OMe
O
N
S
Et
1.00
1.00
1.00
2.00
1.00
1.00
1.00
3.00
1.00
1.00
1.22
1.24
3.00
10.0 9.5 9.0 8.5 8.0 7.5 7.0 6.5 6.0 5.5 5.0 4.5 4.0 3.5 3.0 2.5 2.0 1.5 1.0 0.5 0.0
f1 (ppm)

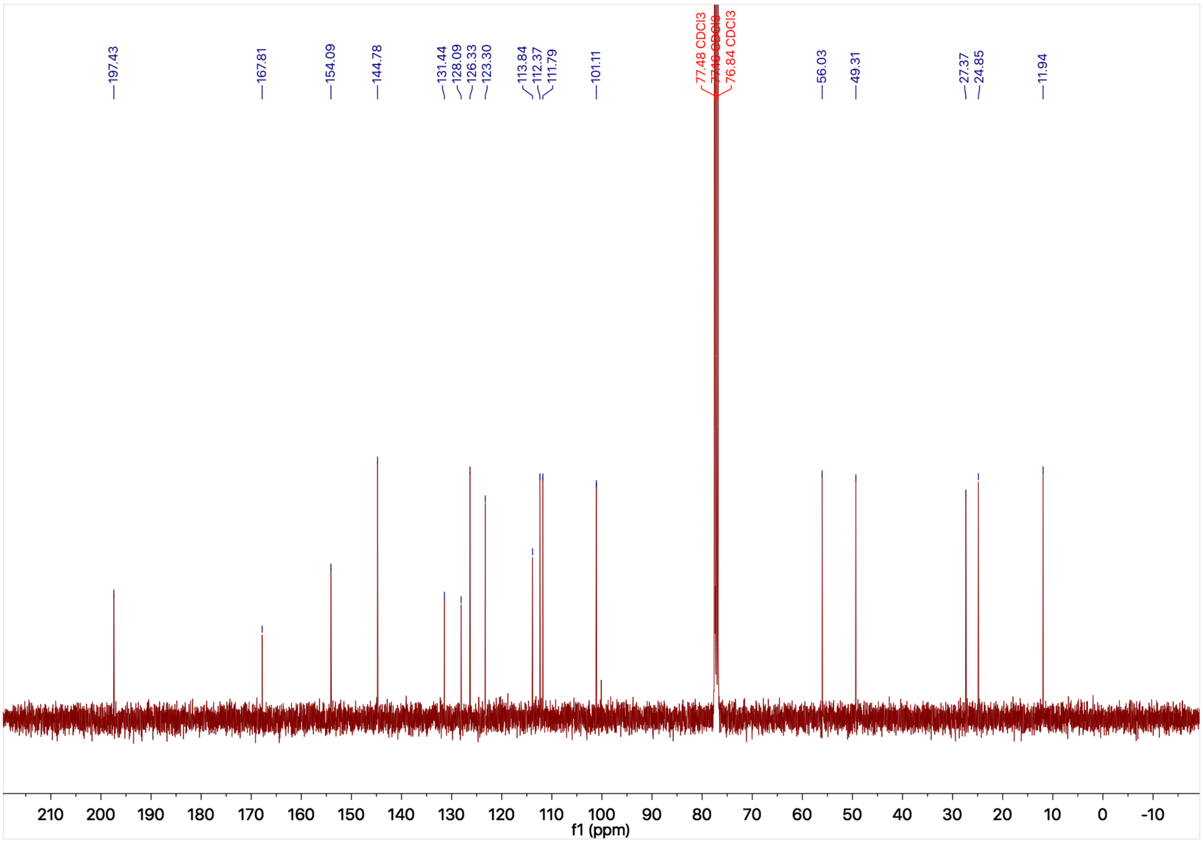
197.43
167.81
154.09
144.78
131.44
128.09
126.33
123.30
113.84
112.37
111.79
101.11
77.48 CDCl3
76.84 CDCl3
56.03
49.31
27.37
24.85
11.94
210 200 190 180 170 160 150 140 130 120 110 100 90 80 70 60 50 40 30 20 10 0 -10
f1 (ppm)

Racemic [Chiralpak IA column, T = 20 °C, *n*-Hexane/*i*-PrOH = 95:5, 1 mL/min, λ = 280 nm, t_R = 34.130 min and t_R = 39.175 min].

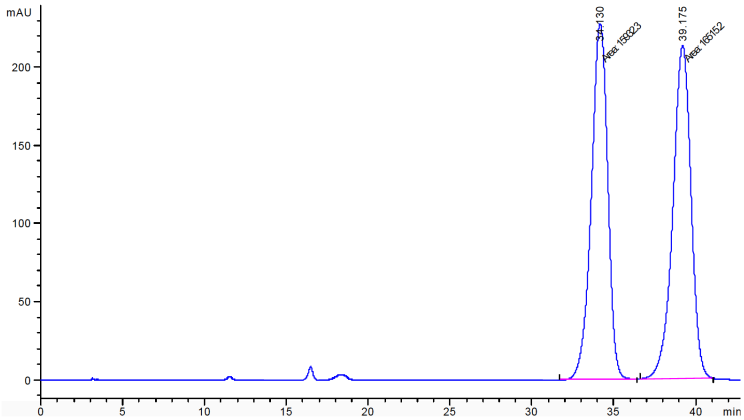

Peak	Retention Time	Rel. Area
	min	%
1	34.13	49.1018
2	39.175	50.8982

Indole [Chiralpak IA column, T = 20 °C, *n*-Hexane/*i*-PrOH = 95:5, 1 mL/min, λ = 280 nm, t_R = 18.965 min].

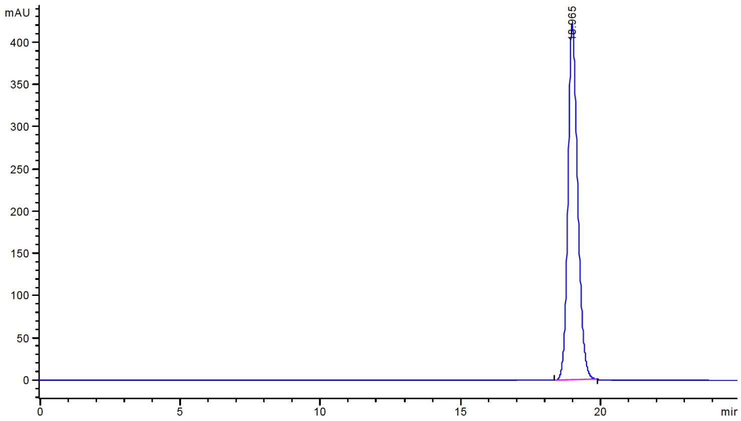

Following the general procedure P. HPLC analysis of the crude residue indicated a ratio indole/**6i** of 16:84 and an enantiomeric excess of (-) 75% [Chiralpak IA column, T = 20 °C, *n*-Hexane/*i*-PrOH = 95:5, 1 mL/min, λ = 280 nm, t_R = 34.066 min and t_R = 39.412 min].

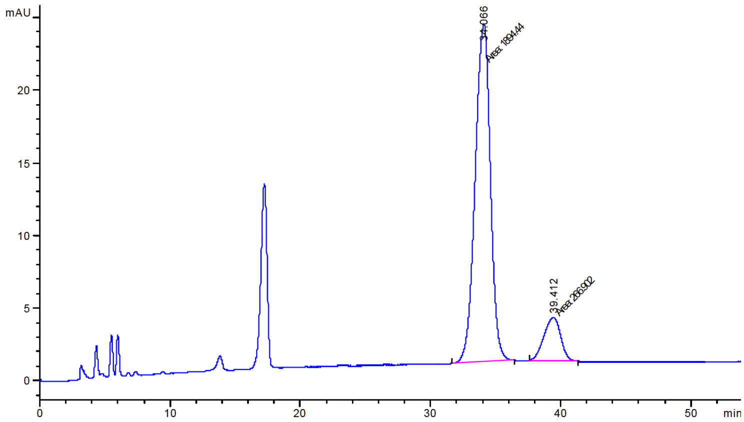

Peak	Retention Time	Rel. Area
	min	%
1	34.066	87.6511
2	39.412	12.3489

Following the general procedure S. HPLC analysis of the crude residue indicated a ratio indole/**6i** of 15:85 and an enantiomeric excess of (+) 51% [Chiralpak IA column, T = 20 °C, *n*-Hexane/*i*-PrOH = 95:5, 1 mL/min, λ = 280 nm, t_R = 34.109 min and t_R = 39.303 min].

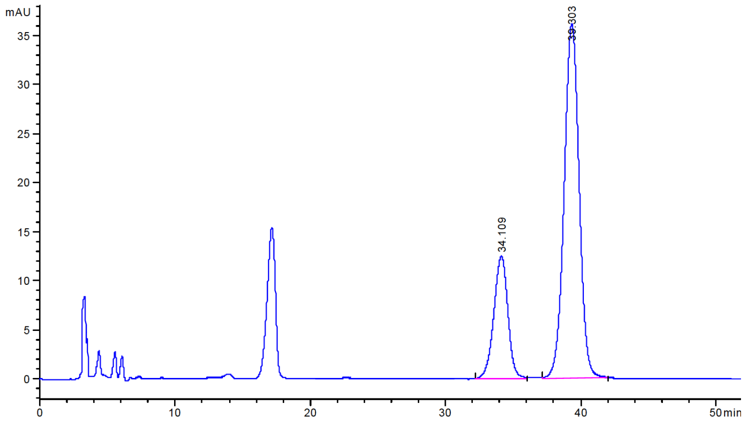

Peak	Retention Time	Rel. Area
	min	%
1	34.109	24.53
2	39.303	75.47

2-((5-Methyl-1*H*-indol-3-yl)methyl)-1-(thiazol-2-yl)butan-1-one (6j)

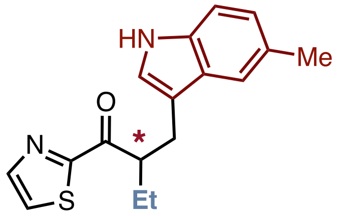

MW (g/mol): 298.40 **Molecular formula:** $C_{17}H_{18}N_2OS$

Synthesised according racemic procedure using **1f**. Isolated as yellow oil.

^{1}H NMR (400 MHz, $CDCl_3$) δ 7.99 (d, *J* = 3.0 Hz, 1H), 7.82 (br s, 1H, -*NH*), 7.62 (d, *J* = 3.0 Hz, 1H), 7.47 (s, 1H), 7.19 (d, *J* = 8.2 Hz, 1H), 6.99 (dd, *J* = 8.3, 1.6 Hz, 1H), 6.94 (d, *J* = 2.4 Hz, 1H), 4.18 (m, 1H), 3.30 (dd, *J* = 14.5, 7.4 Hz, 1H), 2.96 (dd, *J* = 14.5, 6.8 Hz, 1H), 2.47 (s, 3H), 1.93 (m, 1H), 1.76 (m, 1H), 0.94 (t, *J* = 7.4 Hz, 3H).

^{13}C NMR (101 MHz, $CDCl_3$) δ 197.4, 167.8, 144.8, 134.6, 128.6, 128.0, 126.2, 123.6, 122.7, 118.9, 113.5, 110.7, 49.4, 27.0, 25.5, 21.7, 11.9.

HRMS (ESI): m/z calcd for $C_{17}H_{18}N_2OSNa$, $[M+Na]^+$: 321.1038, found: 321.1039.

IR (neat): 3406, 2969, 1682, 1482, 1393, 1231, 797 cm^{-1}.

HPLC: Chiralpak IA column, T = 30 °C, *n*-Hexane/*i*-PrOH = 97:3, 1 mL/min, λ = 280 nm.

Entry	General procedure	Sequences	Conversion (%)[a]	ee (%)[a]
**1**	P	**DNA-SerC2/RNA-U**	95	–77
**2**	S	st-DNA	93	+42

[a]Determined by HPLC. The sign before the ee's values is arbitrary evaluated.

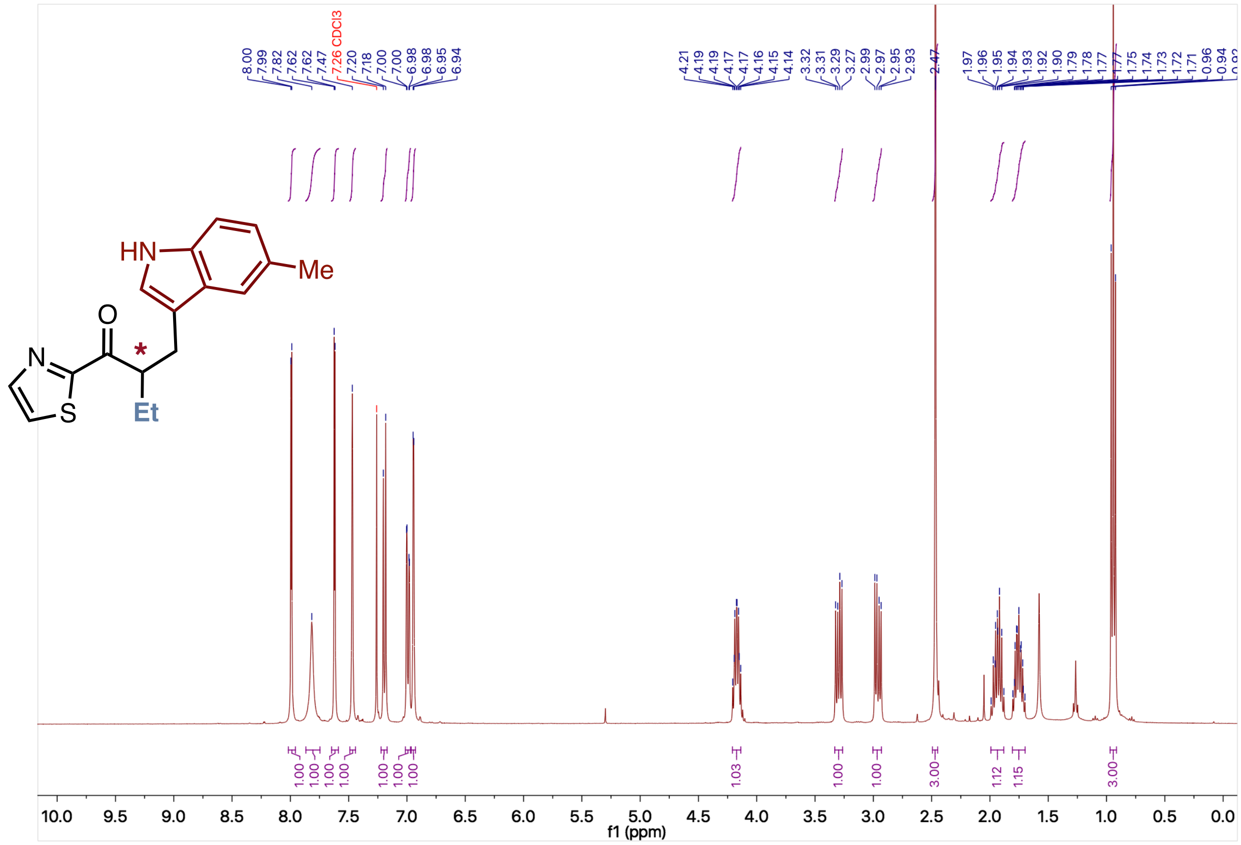

8.00 7.99 7.82 7.62 7.62 7.47 7.26 CDCl3 7.20 7.18 7.00 7.00 6.98 6.95 6.94
4.21 4.19 4.19 4.17 4.17 4.16 4.15 4.14 3.32 3.31 3.29 3.27 2.99 2.97 2.95 2.93 2.47
1.97 1.96 1.95 1.94 1.93 1.92 1.90 1.79 1.78 1.77 1.77 1.75 1.74 1.73 1.72 1.71 0.96 0.94
HN
Me
O
N
S
Et
1.00 1.00 1.00 1.00 1.00 1.00 1.00 1.03 1.00 1.00 3.00 1.12 1.15 3.00
10.0 9.5 9.0 8.5 8.0 7.5 7.0 6.5 6.0 5.5 5.0 4.5 4.0 3.5 3.0 2.5 2.0 1.5 1.0 0.5 0.0
f1 (ppm)

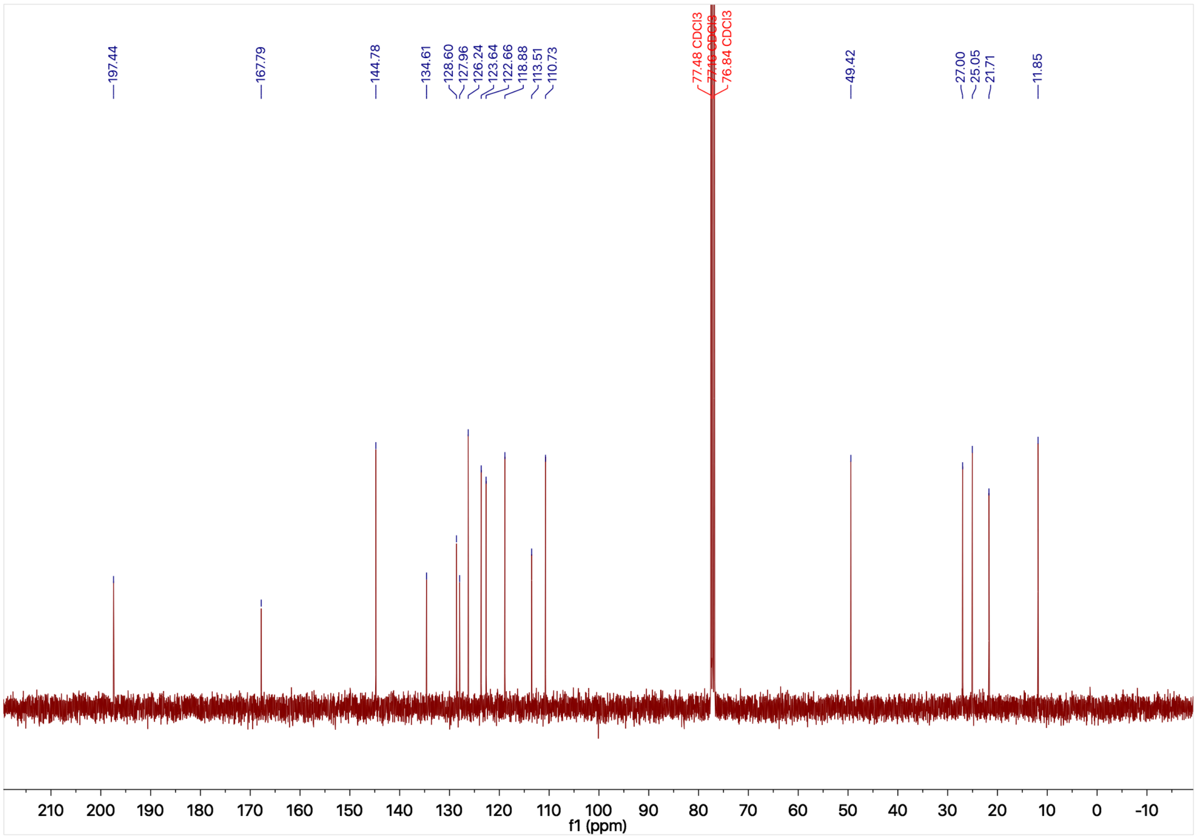

197.44
167.79
144.78
134.61 128.60 127.96 126.24 123.64 122.66 118.88 113.51 110.73
77.48 CDCl3 77.16 CDCl3 76.84 CDCl3
49.42
27.00 25.05 21.71
11.85
210 200 190 180 170 160 150 140 130 120 110 100 90 80 70 60 50 40 30 20 10 0 -10
f1 (ppm)

Racemic [Chiralpak IA column, T = 30 °C, *n*-Hexane/*i*-PrOH = 97:3, 1 mL/min, λ = 280 nm, t_R = 32.625 min and t_R = 35.951 min].

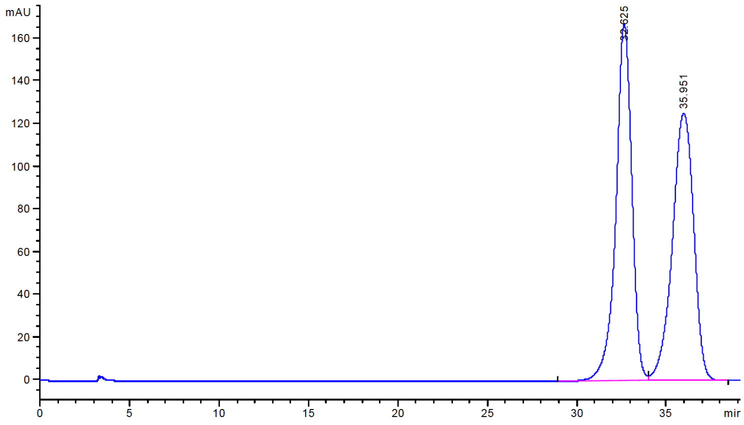

Peak	Retention Time	Rel. Area
	min	%
1	32.625	50.0007
2	35.951	49.9993

Indole [Chiralpak IA column, T = 30 °C, *n*-Hexane/*i*-PrOH = 97:3, 1 mL/min, λ = 280 nm, t_R = 12.395 min].

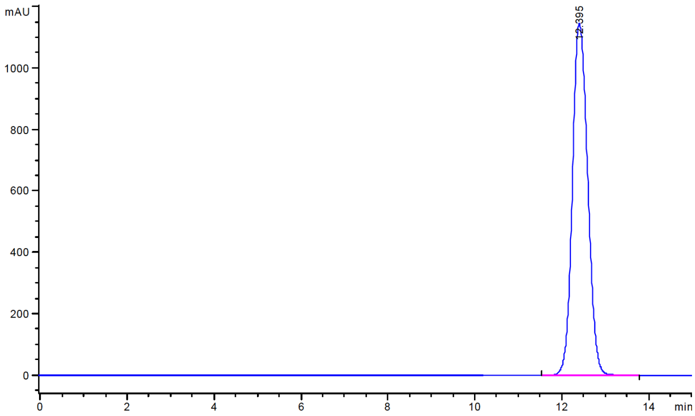

Following the general procedure P. HPLC analysis of the crude residue indicated a ratio indole/**6j** of 5:95 and an enantiomeric excess of (-) 77% [Chiralpak IA column, T = 30 °C, *n*-Hexane/*i*-PrOH = 97:3, 1 mL/min, λ = 280 nm, t_R = 33.297 min and t_R = 36.655 min]. *Presence of an impurity.*

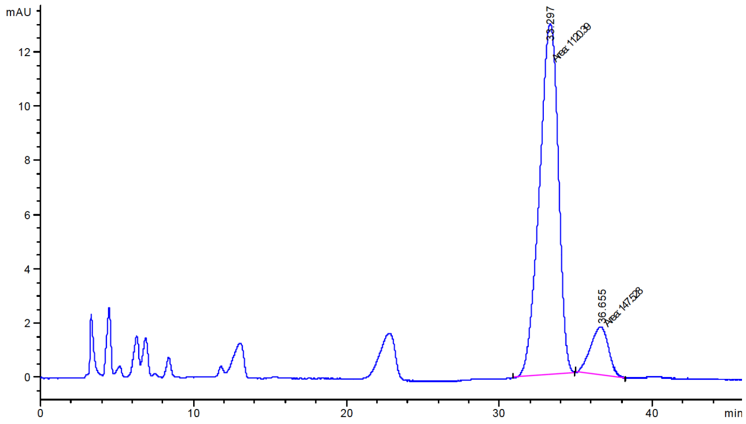

Peak	Retention Time	Rel. Area
	min	%
1	33.297	88.3645
2	36.655	11.6355

Following the general procedure S. HPLC analysis of the crude residue indicated a ratio indole/**6j** of 7:93 and an enantiomeric excess of (+) 42% [Chiralpak IA column, T = 30 °C, *n*-Hexane/*i*-PrOH = 97:3, 1 mL/min, λ = 280 nm, t_R = 32.855 min and t_R = 36.612 min].

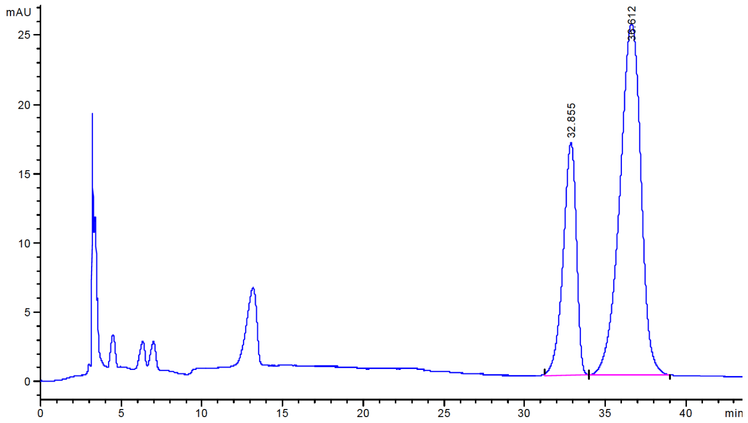

Peak	Retention Time	Rel. Area
	min	%
1	32.855	29.2149
2	36.612	70.7851

2-((7-Methyl-1*H*-indol-3-yl)methyl)-1-(thiazol-2-yl)butan-1-one (6k)

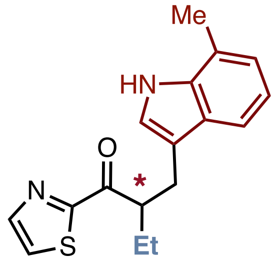

MW (g/mol): 298.40 **Molecular formula:** $C_{17}H_{18}N_2OS$

Synthesised according racemic procedure using **1f**. Isolated as yellow oil.

^{1}H NMR (400 MHz, $CDCl_3$) δ 7.99 (d, *J* = 3.0 Hz, 1H), 7.86 (br s, 1H, -*NH*), 7.62 (d, *J* = 3.0 Hz, 1H), 7.57 (d, *J* = 7.7 Hz, 1H), 7.06 (t, *J* = 7.7 Hz, 1H), 7.00-6.96 (m, 2H), 4.18 (m, 1H), 3.33 (dd, *J* = 14.6, 7.4 Hz, 1H), 2.98 (dd, *J* = 14.6, 6.9 Hz, 1H), 2.45 (s, 3H), 1.93 (m, 1H), 1.75 (m, 1H), 0.94 (t, *J* = 7.5 Hz, 3H).

^{13}C NMR (101 MHz, $CDCl_3$) δ 197.4, 167.8, 144.8, 135.9, 127.3, 126.2, 122.6, 122.3, 120.2, 119.7, 117.0, 114.5, 49.4, 27.1, 25.0, 16.7, 11.9.

HRMS (ESI): m/z calcd for $C_{17}H_{18}N_2OSNa$, $[M+Na]^+$: 321.1038, found: 321.1039.

IR (neat): 3410, 2973, 1676, 1462, 1390, 813 cm^{-1}.

HPLC: Chiralpak IB column, T = 20 °C, *n*-Hexane/*i*-PrOH = 97:3, 1 mL/min, λ = 280 nm.

Entry	General procedure	Sequences	Conversion (%)[a]	ee (%)[a]
**1**	P	**DNA-SerC2/RNA-U**	75	–64
**2**	S	st-DNA	83	+8

[a]Determined by HPLC. The sign before the ee's values is arbitrary evaluated.

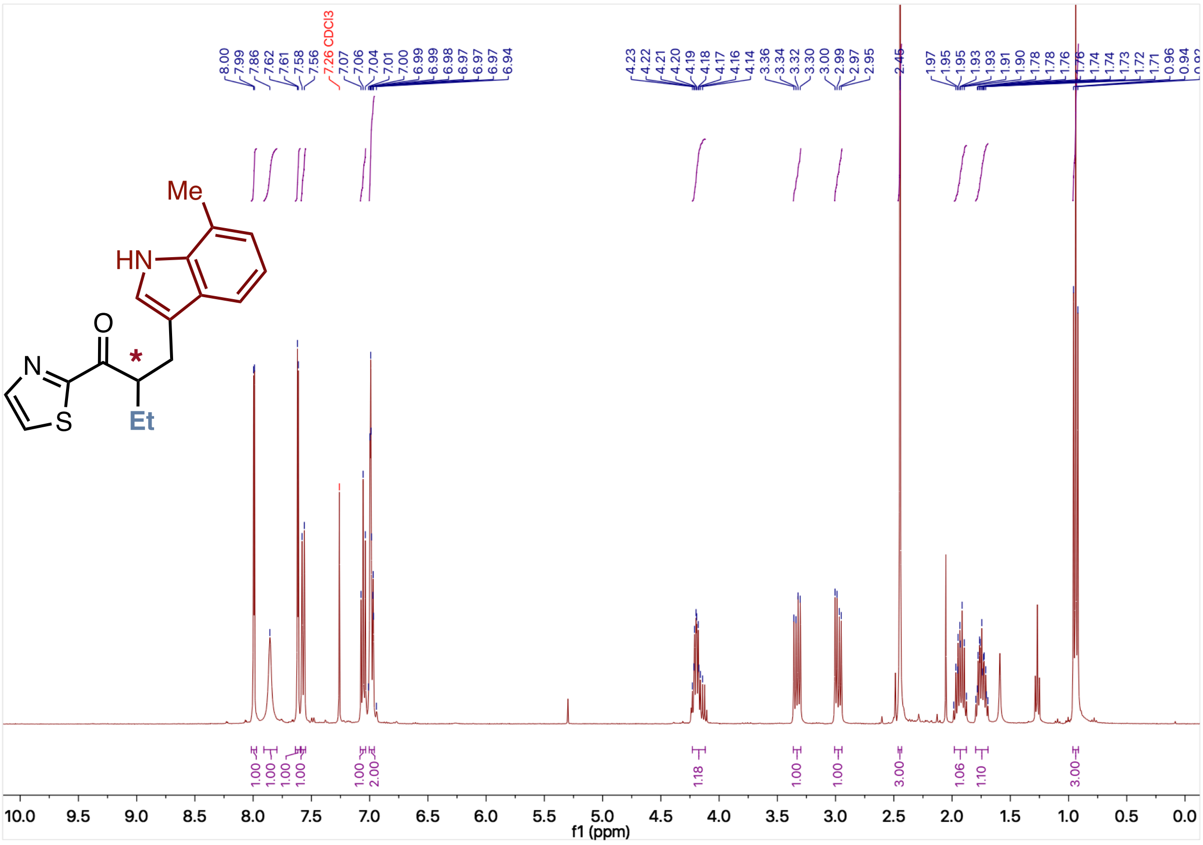
Me
HN
O
N
S
*
Et
8.00 7.99 7.86 7.62 7.61 7.58 7.56
7.26 CDCl3
7.07 7.06 7.04 7.01 7.00 6.99 6.99 6.98 6.97 6.97 6.97 6.94
4.23 4.22 4.21 4.20 4.19 4.18 4.17 4.16 4.14
3.36 3.34 3.32 3.30 3.00 2.99 2.97 2.95
2.45
1.97 1.95 1.95 1.93 1.93 1.91 1.90 1.78 1.78 1.76 1.76 1.74 1.74 1.73 1.72 1.71 0.96 0.94
1.00 1.00 1.00 1.00 1.00 2.00 1.18 1.00 1.00 3.00 1.06 1.10 3.00
10.0 9.5 9.0 8.5 8.0 7.5 7.0 6.5 6.0 5.5 5.0 4.5 4.0 3.5 3.0 2.5 2.0 1.5 1.0 0.5 0.0
f1 (ppm)

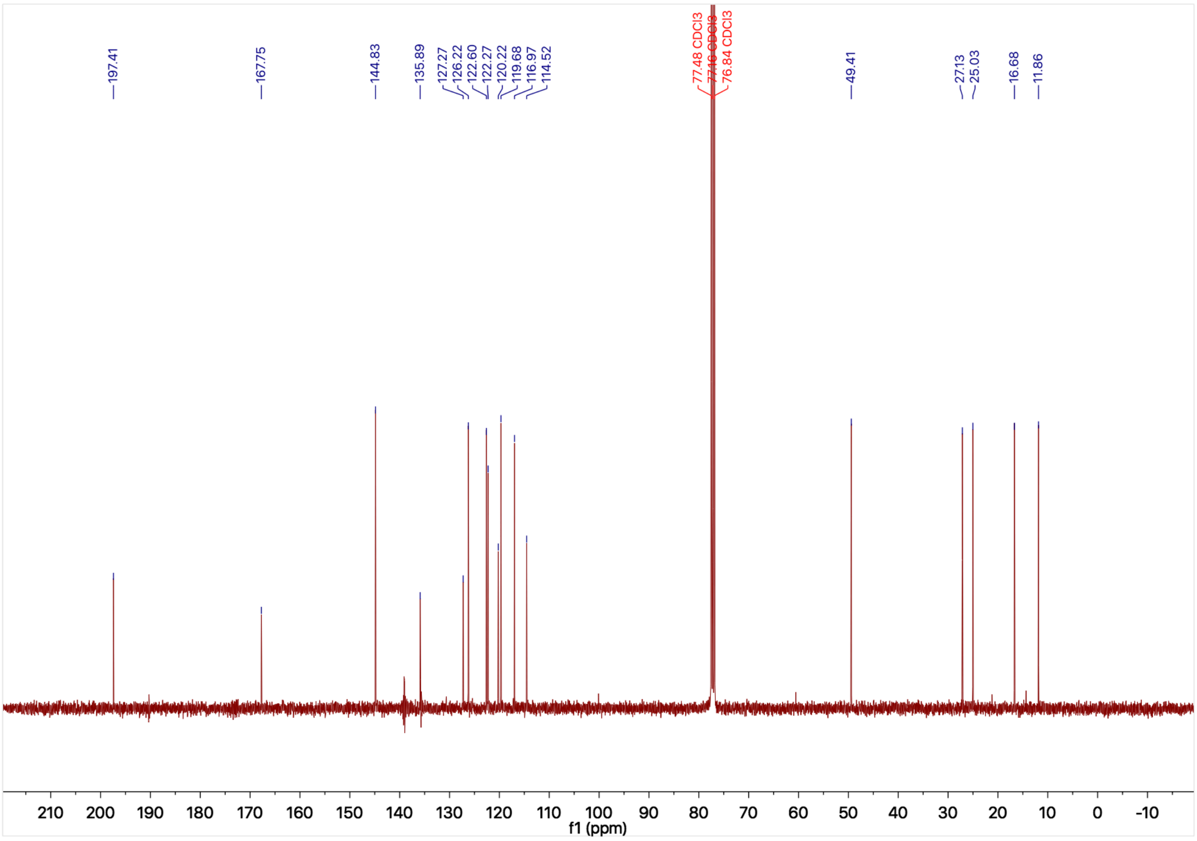
197.41
167.75
144.83
135.89 127.27 126.22 122.60 122.27 120.22 119.68 116.97 114.52
77.48 CDCl3
77.16 CDCl3
76.84 CDCl3
49.41
27.13 25.03
16.68
11.86
210 200 190 180 170 160 150 140 130 120 110 100 90 80 70 60 50 40 30 20 10 0 -10
f1 (ppm)

Racemic [Chiralpak IB column, T = 20 °C, *n*-Hexane/*i*-PrOH = 97:3, 1 mL/min, λ = 280 nm, t_R = 15.098 min and t_R = 40.231 min].

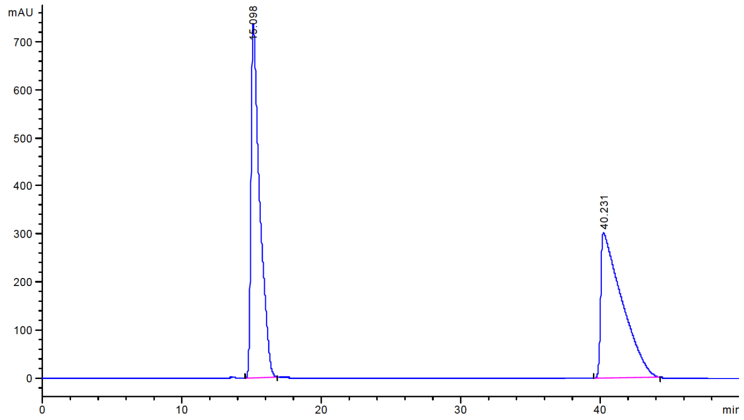

Peak	Retention Time	Rel. Area
	min	%
1	15.098	49.9792
2	40.231	50.0208

Indole [Chiralpak IB column, T = 20 °C, *n*-Hexane/*i*-PrOH = 97:3, 1 mL/min, λ = 280 nm, t_R = 13.034 min].

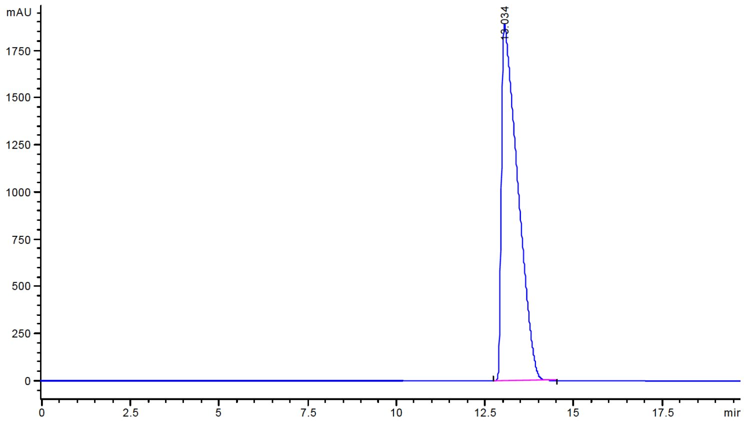

Following the general procedure P. HPLC analysis of the crude residue indicated a ratio indole/**6k** of 25:75 and an enantiomeric excess of (-) 64% [Chiralpak IB column, T = 20 °C, *n*-Hexane/*i*-PrOH = 97:3, 1 mL/min, λ = 280 nm, t_R = 16.140 min and t_R = 42.971 min].

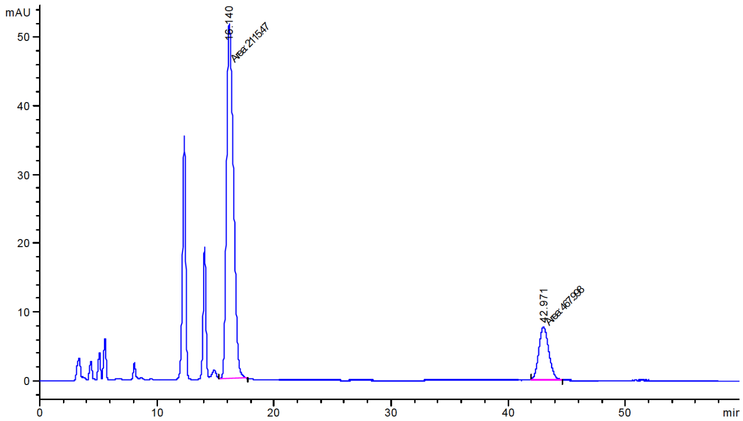

Peak	Retention Time	Rel. Area
	min	%
1	16.14	81.8849
2	42.971	18.1151

Following the general procedure S. HPLC analysis of the crude residue indicated a ratio indole/**6k** of 17:83 and an enantiomeric excess of (+) 8% [Chiralpak IB column, T = 20 °C, *n*-Hexane/*i*-PrOH = 97:3, 1 mL/min, λ = 280 nm, t_R = 15.227 min and t_R = 43.106 min].

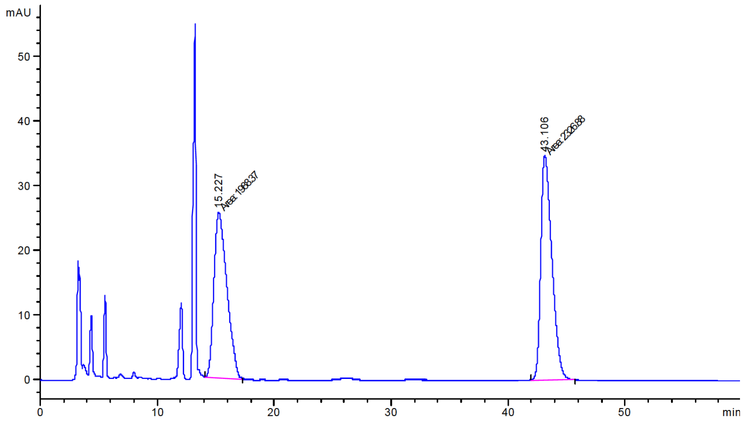

Peak	Retention Time	Rel. Area
	min	%
1	15.227	46.0778
2	43.106	53.9222

V. Computational molecular modelling

Initial models of investigated duplexes were constructed using X-ray crystallography structure of dodecameric DNA-RNA hybrid (PDB code: 4WKJ), DNA dodecamer (PDB code: 1BNA), and RNA dodecamer (PDB code: 6XUS). The nucleotide sequences were adjusted to match sequences shown in Table 2 using UCSF Chimera[17] macromolecular modelling suite. Bipyridine (BiPy) within the leading strand and modified residues on the counter-strand were modelled in Chimera, joined, and subjected to 5,000 cycles of all-atom molecular mechanical energy minimisation in implicit solvent. Partial atomic charges were calculated using AM1-BCC method,[18] and parameters needed for subsequent molecular dynamics simulations were derived using ACPYPE scripts.[19]

To address the stability, intrinsic dynamics and subtle conformational changes within the catalytic sites of all catalysts, modelled duplexes with modifications (DNA/DNA, DNA/RNA, RNA/DNA and RNA/RNA) were subjected to all-atom MD simulations in explicit water. All simulations were carried out in GROMACS 2016.1[20] using the suite with OL15[21] and OL3[22] force fields. A cubic box was centred around each duplex with a 1 nm distance between the nucleotide extreme to the edge, which was immersed in the TIP3P water model.[23] Bonds were constrained using the LINCS[24] algorithm with a 2 fs time step. The electrostatic interactions were calculated using the particle-mesh Ewald (PME) method,[25] with a non-bonded cut-off set at 0.1 nm. All duplexes were energy-minimised using the steepest descent algorithm for 20,000 steps. The minimisation was stopped when the maximum force fell below 1000 kJ/mol/nm using the Verlet cut-off scheme. After the energy minimisation, the models were heated to room temperature (300 K) using NVT ensemble; thermal equilibration was performed for 100 ps with a time step of 2 fs with position restraint applied to the nucleic acid backbone atoms. The temperature coupling was set between the nucleic acid and the non-nucleic acid entities by using a V-rescaling thermostat[26] with a time constant of 0.1 ps and the temperature was set to reach the target with the pressure coupling off. The temperature was set constant at 300 K by using a modified Berendsen thermostat (τ = 0.1 ps).[27] The pressure was kept constant at 1 bar by Parinello-Rahman[28] isotropic coupling (τ = 2.0 ps) to a pressure bath. Subsequently, the 100 ps NPT equilibration was performed with no positional restraints applied to the backbone atoms, and three parallel NPT production run replicas were run for 100 ns.

Analysis of MD production replicas was performed using GROMACS tools, and included RMSD (root-mean-square deviation) calculations to assess the equilibration, convergence and structural stability of the duplexes; RMSF (root-mean-square fluctuation) calculations to assess the flexibility per-residue and subtle conformational changes; solvent-accessible surface area (SASA) to evaluate the size and area of the catalytic site; cluster analysis to evaluate heterogeneity of produced molecular ensembles. For each duplex, all trajectories were concatenated before the cluster analysis.

After the simulations, one reference frame representing the centroid of the RMSD cluster was selected for each duplex. The complexes with the copper ions were modelled in Chimera, with the subsequent addition of the α,β-unsaturated-2-acyl imidazole completing

the coordination site performed in SeeSAR version 11[29] with HYDE scoring function.[30,31] The sampling was set to the maximum (500 poses for the imidazole), and the clash tolerance was set to high. The lowest-energy orientation of the imidazole moiety was selected as a docking receptor for modelling of the second reactant. The 5-methoxyindole was docked using the same protocol as described for α,β-unsaturated-2-acyl imidazole. The best-scored poses were selected for a visual inspection, and the products were modelled in Chimera.

To evaluate the stereoselectivity of the catalysts, we next compared the energetics of reactions leading to different isomers; we calculated the energetics of the best-scoring reactants leading to different products using semi-empirical quantum mechanical calculations. The *R* and *S* stereoisomers modelled from the best-scoring reactant complexes were selected for these evaluations. The energies of both reactant poses were obtained using the SQM/PM6 method[32] with implicit continuum solvation. The SCF was set to converge quadratically with the symmetry of the system being ignored. The calculations were set up using GaussView and were carried out using Gaussian09.[33]

Table S2. Single-point energy calculations of reactants yielding different enantiomers (PM6 Hamiltonian). Binding poses subjected to the calculations were predicted by molecular docking using SeeSAR, with HYDE scoring function.

Highest affinity pose yielding the *R* enantiomer	–915 kcal/mol
Highest affinity pose yielding the *S* enantiomer	–884 kcal/mol
ΔE *R* over *S* product	–31 kcal/mol

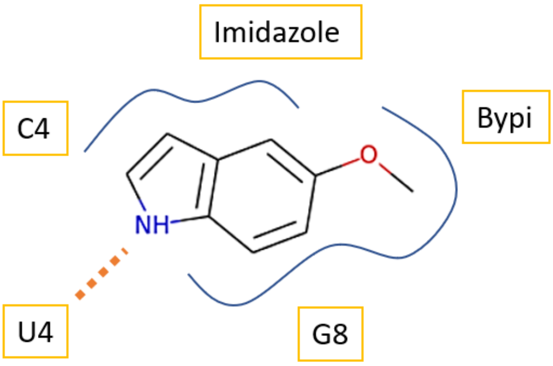

Figure S1. Interactions of the reactant (5-methoxyindole) sampled *via* molecular docking calculations. The dashed line represents a stabilising hydrogen bond, and the blue continuous line represents favourable hydrophobic contacts.

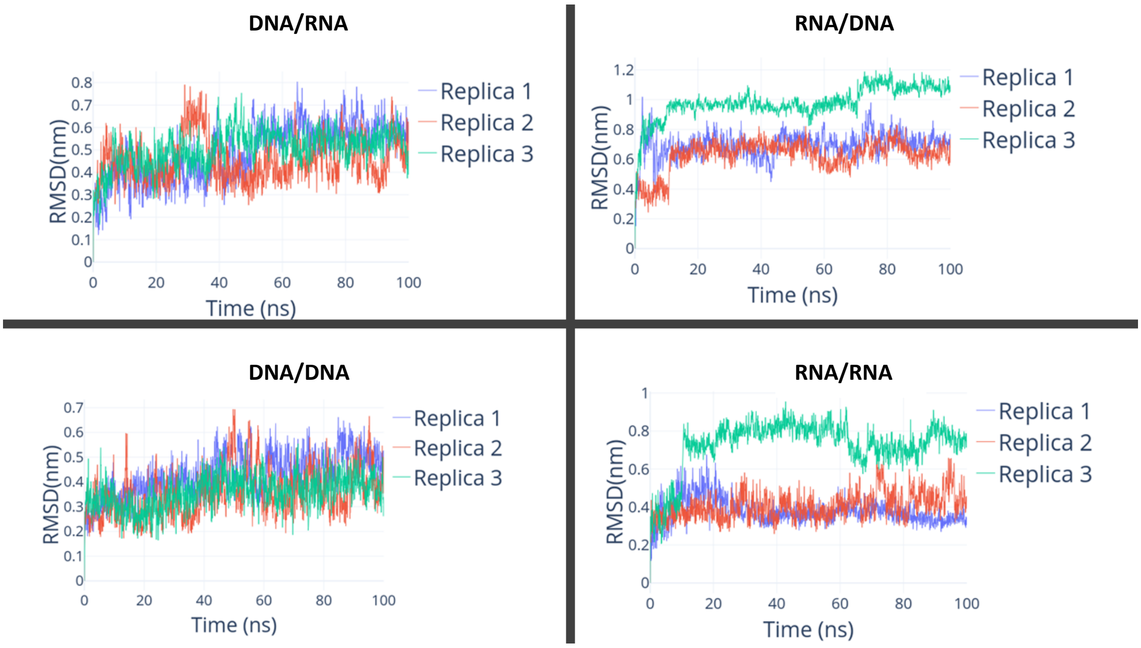

Figure S2. Root-mean-square deviations (RMSD) in nm were calculated for all four investigated catalysts (DNA/DNA, DNA/RNA, RNA/DNA and RNA/RNA), sampled over 100 ns of all-atom MD simulations. Data for all three simulation replicas are shown.

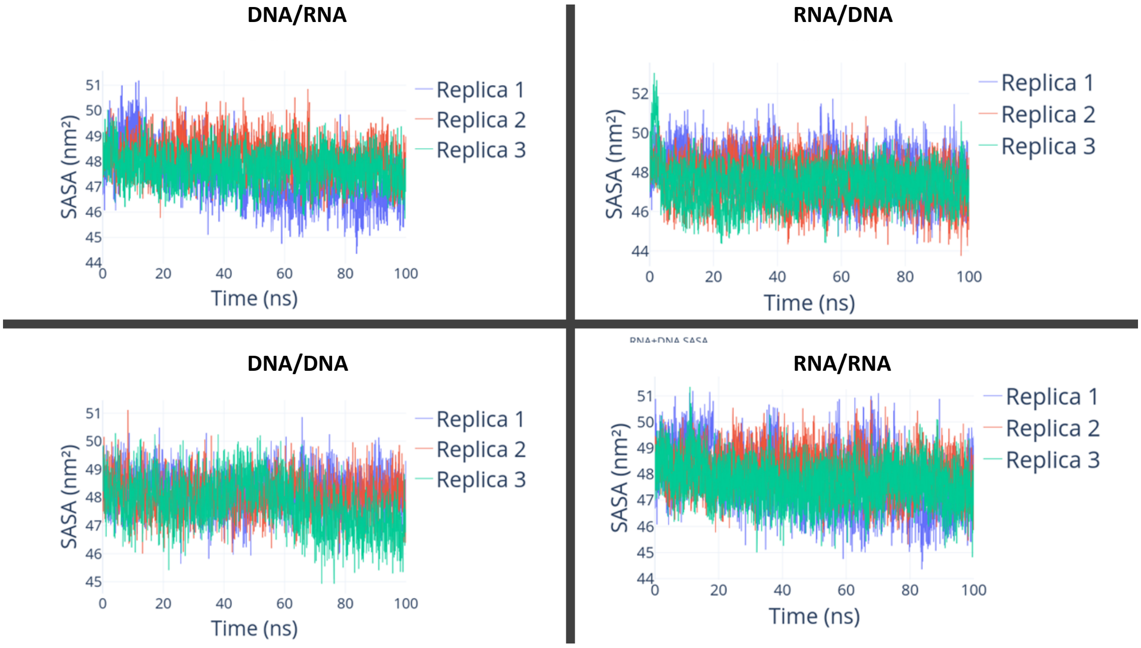

Figure S3. Total solvent-accessible surface area (SASA) in nm^2 calculated for all four investigated catalysts (DNA/DNA, DNA/RNA, RNA/DNA and RNA/RNA), sampled over 100 ns of all-atom MD simulations. Data for all three simulation replicas are shown.

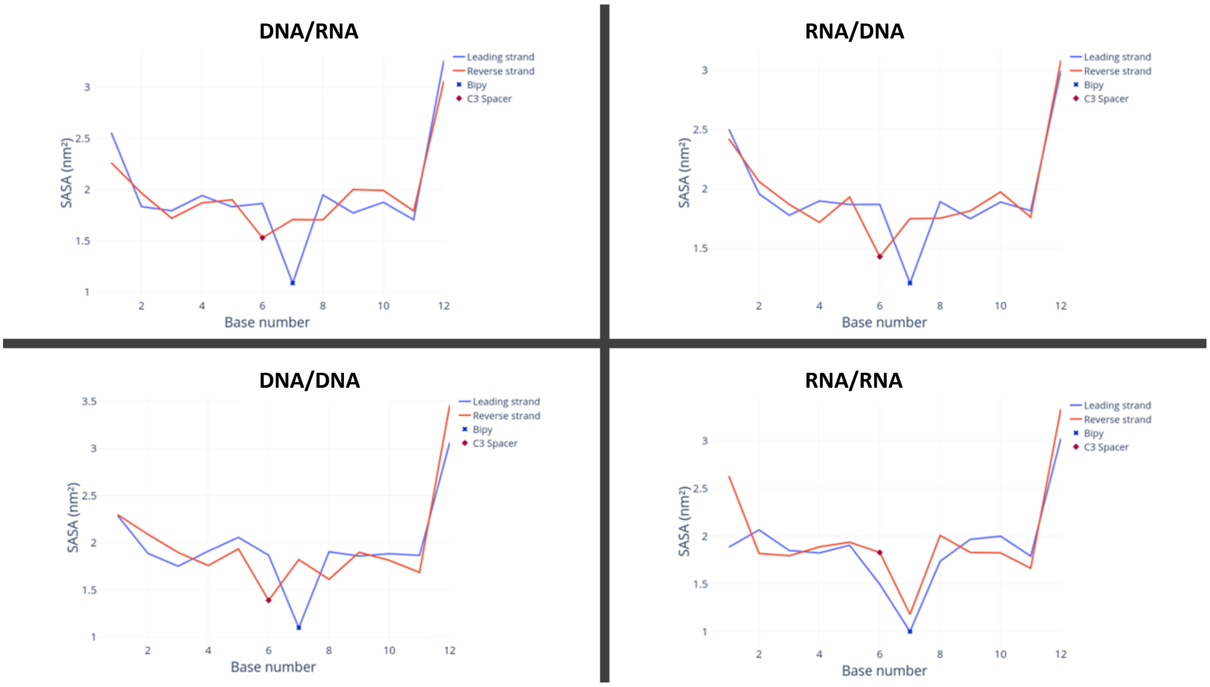

Figure S4. Average solvent-accessible surface area (SASA) in nm^2 was calculated for each base in all four investigated catalysts (DNA/DNA, DNA/RNA, RNA/DNA and RNA/RNA). The averages were taken over 100 ns of all-atom MD simulations.

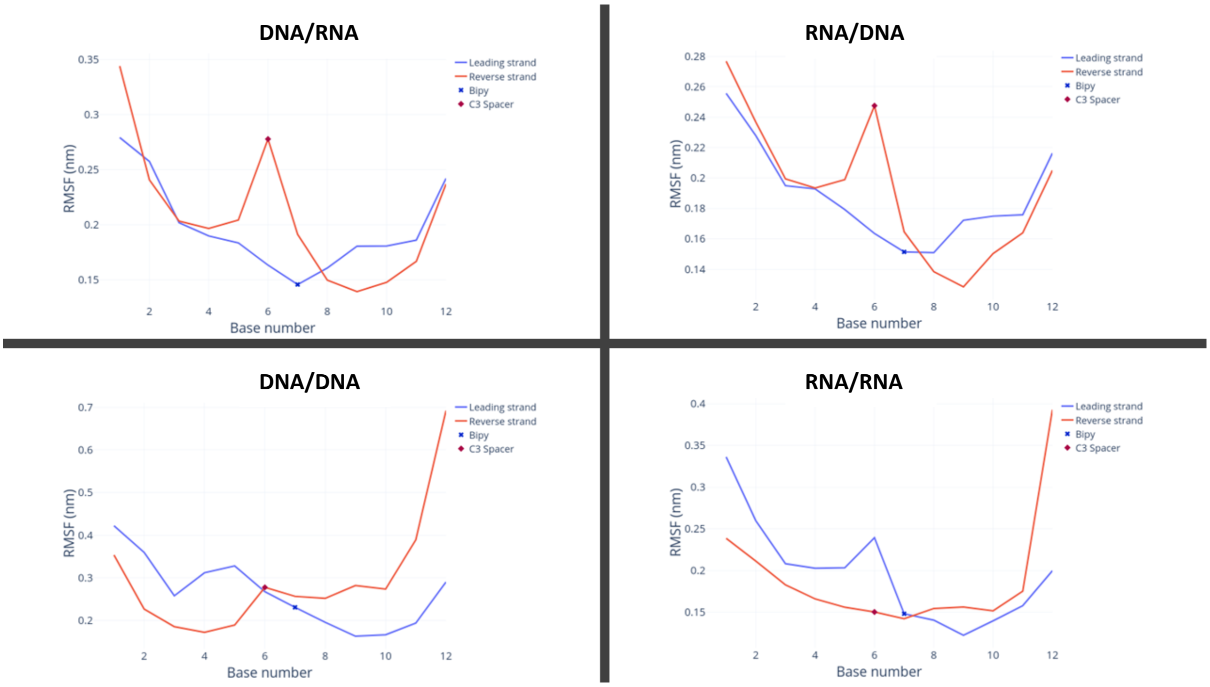

Figure S5. Per-residue root-mean-square fluctuations (RMSF) in nm, calculated for each base in all four investigated catalysts (DNA/DNA, DNA/RNA, RNA/DNA and RNA/RNA).

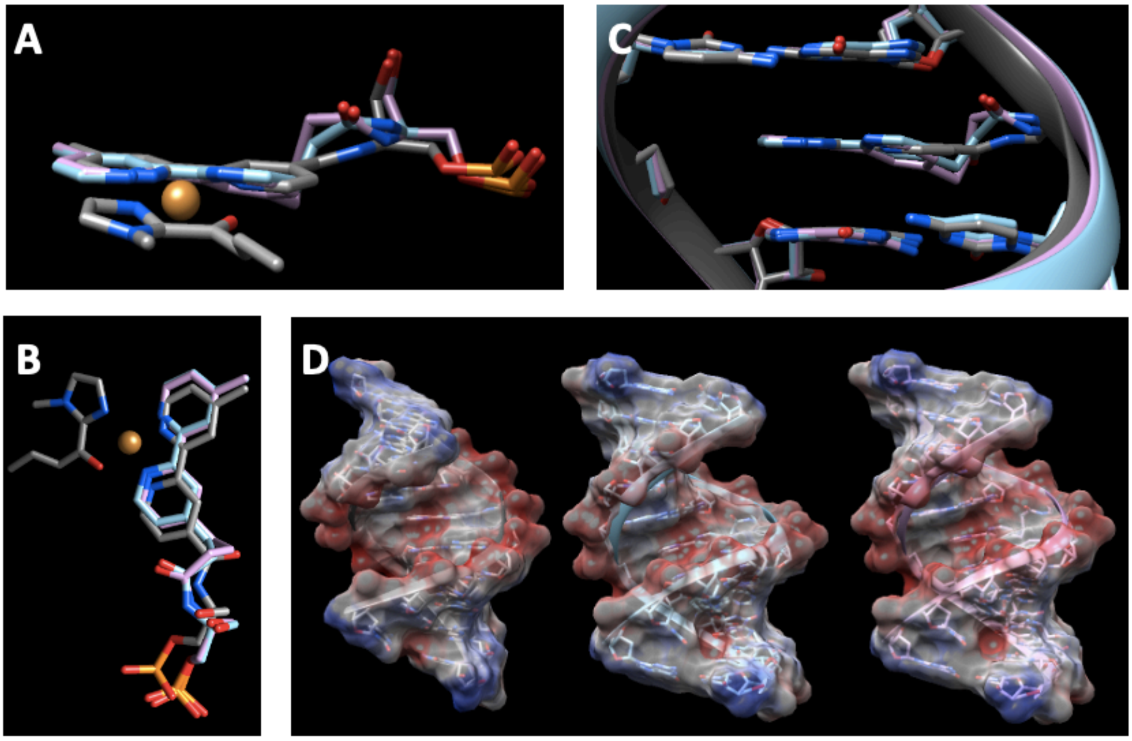

Figure S6. (**A**) and (**B**) Effects of different bipyridine linkers on the planarity of the bipyridine and interactions with copper (orange) and imidazole. $BipyC_0$ (dark grey) is fully planar, whereas $BipyC_2$ (cyan) and $BipyC_3$ (purple) are not. (**C**) The amide bond linking $BipyC_0$ exhibits an *E* conformation, while the corresponding amide bond linking $BipyC_2$ and $BipyC_3$ retain a *Z* conformation. (**D**) Surface of complexes with $BipyC_0$ (left), $BipyC_2$ (middle), and $BipyC_3$ (right), coloured by electrostatic (Coulombic) potential. Colour values: <–25 kcal/mol (red), –10 kcal/mol (white), < 0 kcal/mol (blue). $BipyC_0$ shows a reduced and more localised negatively charged electrostatic potential "patch" in the middle of the binding site compared to $BipyC_2$ and $BipyC_3$. This localised central spot corresponds to the copper binding site.

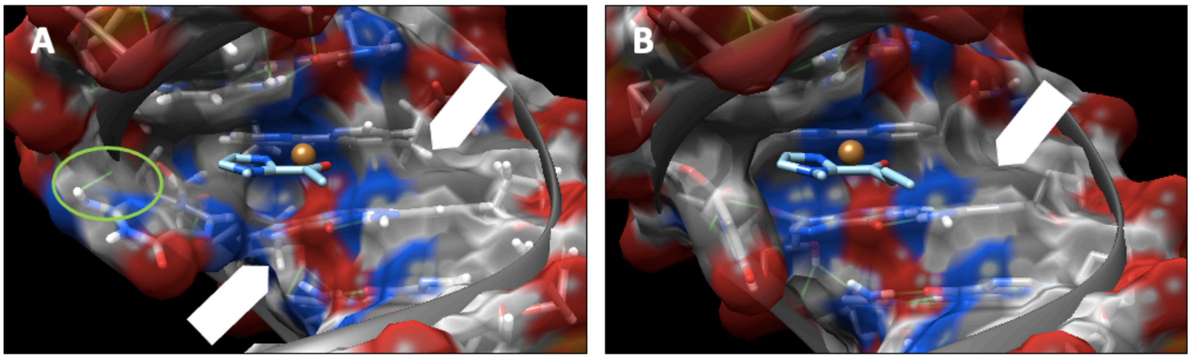

Figure S7. (**A**) Three-dimensional model of entry 7 (Table 3) bound to copper (orange) and imidazole (cyan). The guanine present on the counter-strand is stabilised by an intramolecular hydrogen bond between N2 and OP2 atoms. This bond is circled and highlighted in green. It affects the local conformation of the active site, which allows for the reactant (e.g. 5-methoxyindole) to approach and favour the formation of the *S*-enantiomer of the product. (**B**) In the model of entry 10 (Table 3), the conformation of the uracil in the counter-strand is not stabilised by an intramolecular hydrogen bond between the base and the backbone. This changes the uracil's conformation, allowing the reactant to approach from the "top" (marked by the white arrow), thus favouring the formation of the *R*-enantiomer of the product.

References

[1] Y. Huang, D. R. Dalton, P. J. Carroll. The efficient, enantioselective synthesis of aza sugars from amino acids. 1. The polyhydroxylated pyrrolidines. *J. Org. Chem.* **1997**, *62*, 372–376.

[2] H.-K. Kim, K.-J. J. Park. *Tetrahedron Lett.* **2012**, *53*, 1668–1670.

[3] F. Hövelmann, I. Gaspar, J. Chamiolo, M. Kasper, J. Steffen, A. Ephrussi, O. Seitz. *Chem. Sci.* **2016**, *7*, 128–135.

[4] R. Benhida, M. Devys, J.-L. Fourrey, F. Lecubin, J.-S. Sun. *Tetrahedron Lett.* **1998**, *39*, 6167–6170.

[5] S. I. Antsypovich, T. S. Oretskaya, G. Von Kiedrowski. *Russ. Chem. Bull.* **2005**, *54*, 2671–2681.

[6] Y. Chujo, K. Sada, T. Saegusa. *Polym. J.* **1993**, *25*, 599–608.

[7] W. Nussbaumer, H. Gruber, G. F. Greber. *Monatshefte Für Chem. Chem. Mon.* **1988**, *119*, 1–15.

[8] D. A. Evans, K. R. Fandrick, H.-J. Song. *J. Am. Chem. Soc.* **2005**, *127*, 8942–8943.

[9] M. C. Myers, A. R. Bharadwaj, B. C. Milgram, K. A. Scheidt. *J. Am. Chem. Soc.* **2005**, *127*, 14675–14680.

[10] J. Wang, E. Benedetti, L. Bethge, S. Vonhoff, S. Klussmann, J.-J. Vasseur, J. Cossy, M. Smietana, S. Arseniyadis. *Angew. Chem. Int. Ed.* **2013**, *52*, 11546–11549.

[11] A. García-Fernández, R. P. Megens, L. Villarino, G. Roelfes. *J. Am. Chem. Soc.* **2016**, *138*, 16308–16314.

[12] A. J. Boersma, B. L. Feringa, G. Roelfes. *Angew. Chem. Int. Ed.* **2009**, *48*, 3346–3348.

[13] E. Benedetti, N. Duchemin, L. Bethge, S. Vonhoff, S. Klussmann, J.-J. Vasseur, J. Cossy, M. Smietana, S. Arseniyadis. *Chem. Commun.* **2015**, *51*, 6076–6079.

[14] D. Coquière, B. L. Feringa, G. Roelfes. *Angew. Chem. Int. Ed.* **2007**, *46*, 9308–9311.

[15] Y. Li, C. Wang, G. Jia, S. Lu, C. Li. *Tetrahedron* **2013**, *69*, 6585–6590.

[16] A. J. Boersma, B. L. Feringa, G. Roelfes. *Org. Lett.* **2007**, *9*, 3647–3650.

[17] E. F. Pettersen, T. D. Goddard, C. C. Huang, G. S. Couch, D. M. Greenblatt, E. C. Meng, T. E. Ferrin. UCSF Chimera - A visualization system for exploratory research and analysis. *J. Comput. Chem*. **2004**, *25*, 1605–1612.

[18] A. Jakalian, D. B. Jack, C. I. Bayly. Fast, efficient generation of high-quality atomic charges. AM1-BCC model: II. Parameterization and validation. *J. Comput. Chem*. **2002**, *23*, 1623–1641.

[19] A. W Sousa da Silva, W. F Vranken. ACPYPE - AnteChamber PYthon Parser interfacE. *BMC Research Notes* **2012**, *5*:367.

[20] M. J. Abraham, T. Murtola, R. Schulz, S. Páll, J. C. Smith, B. Hess, E. Lindahl. GROMACS: High performance molecular simulations through multi-level parallelism from laptops to supercomputers. *SoftwareX* **2015**, *1–2*, 19–25.

[21] R. Galindo-Murillo, J. C. Robertson, M. Zgarbovic, J. Sponer, M. Otyepka, P. Jureska, T. E. Cheatham. Assessing the current state of Amber force field modifications for DNA. *J. Chem. Theory Comput*. **2016**, *12*, 4114–4127.

[22] M. Zgarbová, M. Otyepka, J. Sponer, A. Mládek, P. Banáš, T. E. Cheatham, P. Jurečka. Refinement of the Cornell *et al*. nucleic acids force field based on reference quantum chemical calculations of glycosidic torsion profiles. *J. Chem. Theory Comput*. **2011**, *7*, 2886–2902.

[23] W. L. Jorgensen, J. Chandrasekhar, J. D. Madura, R. W. Impey, M. L. Klein. Comparison of simple potential functions for simulating liquid water. *J. Chem. Phys*. **1983**, *79*, 926–935.

[24] B. Hess. P-LINCS: A Parallel Linear Constraint Solver for Molecular Simulation. *J. Chem. Theory Comput*. **2008**, *4*, 116–122.

[25] T. Darden, D. York, L. Pedersen. Particle mesh Ewald: An N•log(N) method for Ewald sums in large systems. *J. Chem. Phys*. **1993**, *98*, 10089–10092.

[26] G. Bussia, D. Donadio, M. Parrinello. Canonical sampling through velocity rescaling. *J. Chem. Phys*. **2007**, *126*, 014101.

[27] G. Bussi, D. Donadio, M. Parrinello. Canonical sampling through velocity rescaling. *J. Chem. Phys*. **2007**, *126*, 014101.

[28] M. Parrinello, A. Rahman. Crystal structure and pair potentials: A molecular-dynamics

study. *Phys. Rev. Lett*. **1980**, *45*, 1196–1199.

[29] https://www.biosolveit.de/SeeSAR/

[30] I. Reulecke, G. Lange, J. Albrecht, R. Klein, M. Rarey. Towards an Integrated Description of Hydrogen Bonding and Dehydration: Decreasing False Positives in Virtual Screening with the HYDE Scoring Function. *ChemMedChem* **2008**, *3*, 885–897.

[31] N. Schneider, G. Lange, S. Hindle, R. Klein, M. Rarey. A consistent description of HYdrogen bond and DEhydration energies in protein-ligand complexes: methods behind the HYDE scoring function. *J. Comput Aided Mol Des*. **2013**, *27*, 15–29.

[32] J. J. P. Stewart. Optimization of parameters for semiempirical methods V: Modification of NDDO approximations and application to 70 elements. *Journal of Molecular Modeling* **2007**, *13*, 1173–1213.

[33] https://gaussian.com/g09citation/
